# *QuBiLS*-*MAS*, open source multi-platform software for atom- and bond-based topological (2D) and chiral (2.5D) algebraic molecular descriptors computations

**DOI:** 10.1186/s13321-017-0211-5

**Published:** 2017-06-07

**Authors:** José R. Valdés-Martiní, Yovani Marrero-Ponce, César R. García-Jacas, Karina Martinez-Mayorga, Stephen J. Barigye, Yasser Silveira Vaz d‘Almeida, Hai Pham-The, Facundo Pérez-Giménez, Carlos A. Morell

**Affiliations:** 1 StreelBridge Laboratories, SteelBridge Consulting Technology Solutions, Miami, FL USA; 2 Universidad San Francisco de Quito (USFQ), Grupo de Medicina Molecular y Traslacional (MeM&T), Colegio de Ciencias de la Salud (COCSA), Escuela de Medicina, Edificio de Especialidades Médicas, Quito, Ecuador; 3 Universidad San Francisco de Quito (USFQ), Instituto de Simulación Computacional (ISC-USFQ), Diego de Robles y vía Interoceánica, 170157 Quito, Pichincha Ecuador; 4 Computer-Aided Molecular “Biosilico” Discovery and Bioinformatics Research International Network (CAMD-BIR IN), Cumbayá, Quito, Ecuador; 5 Grupo de Investigación Ambiental (GIA), Fundación Universitaria Tecnológico de Comfenalco, Facultad de Ingenierías, Programa de Ingeniería de Procesos, Cartagena de Indias, Bolívar Colombia; 6 Unidad de Investigación de Diseño de Fármacos y Conectividad Molecular, Departamento de Química Física, Facultad de Farmacia, Universitat de València, Valencia, Spain; 7 Instituto de Química, Universidad Nacional Autónoma de México (UNAM), Ciudad de México, México; 8 Escuela de Sistemas y Computación, Pontificia Universidad Católica del Ecuador Sede Esmeraldas (PUCESE), Esmeraldas, Ecuador; 9 Grupo de Investigación de Bioinformática, Universidad de las Ciencias Informáticas (UCI), Havana, Cuba; 10 Facultad de Medicina, Universidad de Las Américas, Quito, Pichincha Ecuador; 11 YByte - Soluções Informáticas, Lda, Avenida da Independência, São Tomé, Sao Tome and Principe; 12 Department of Pharmaceutical Chemistry, Hanoi University of Pharmacy, 13-15 Le Thanh Tong, Hoan Kiem, Hanoi, Vietnam; 13 Laboratorio de Inteligencia Artificial, Centro de Estudios de Informática (CEI), Facultad de Matemática, Física y Computación, Universidad Central “Marta Abreu” de Las Villas, Santa Clara, Villa Clara Cuba

**Keywords:** ToMoCoMD-CARDD, QuBiLS-MAS, Linear, Bilinear and quadratic indices, Atom/bond-based molecular descriptor, Non-stochastic, Simple stochastic, Double stochastic, Mutual probability matrices, QSAR, Free and open source software

## Abstract

**Background:**

In previous reports, Marrero-Ponce et al. proposed algebraic formalisms for characterizing topological (2D) and chiral (2.5D) molecular features through atom- and bond-based ToMoCoMD-CARDD (acronym for Topological Molecular Computational Design-Computer Aided Rational Drug Design) molecular descriptors. These MDs codify molecular information based on the bilinear, quadratic and linear algebraic forms and the *graph*-*theoretical electronic*-*density and edge*-*adjacency matrices* in order to consider atom- and bond-based relations, respectively. These MDs have been successfully applied in the screening of chemical compounds of different therapeutic applications ranging from antimalarials, antibacterials, tyrosinase inhibitors and so on. To compute these MDs, a computational program with the same name was initially developed. However, this *in house* software barely offered the functionalities required in contemporary molecular modeling tasks, in addition to the inherent limitations that made its usability impractical. Therefore, the present manuscript introduces the QuBiLS-MAS (acronym for Quadratic, Bilinear and N-Linear mapS based on graph-theoretic electronic-density Matrices and Atomic weightingS) software designed to compute topological (0–2.5D) molecular descriptors based on bilinear, quadratic and linear algebraic forms for atom- and bond-based relations.

**Results:**

The QuBiLS-MAS module was designed as standalone software, in which extensions and generalizations of the former ToMoCoMD-CARDD 2D-algebraic indices are implemented, considering the following aspects: (a) two new matrix normalization approaches based on double-stochastic and mutual probability formalisms; (b) topological constraints (cut-offs) to take into account particular inter-atomic relations; (c) six additional atomic properties to be used as weighting schemes in the calculation of the molecular vectors; (d) four new local-fragments to consider molecular regions of interest; (e) number of lone-pair electrons in chemical structure defined by diagonal coefficients in matrix representations; and (f) several aggregation operators (*invariants*) applied over atom/bond-level descriptors in order to compute global indices. This software permits the parallel computation of the indices, contains a batch processing module and data curation functionalities. This program was developed in Java v1.7 using the Chemistry Development Kit library (version 1.4.19). The QuBiLS-MAS software consists of two components: a *desktop interface* (GUI) and an *API library* allowing for the easy integration of the latter in chemoinformatics applications. The relevance of the novel extensions and generalizations implemented in this software is demonstrated through three studies. Firstly, a comparative Shannon’s entropy based variability study for the proposed QuBiLS-MAS and the DRAGON indices demonstrates superior performance for the former. A principal component analysis reveals that the QuBiLS-MAS approach captures chemical information orthogonal to that codified by the DRAGON descriptors. Lastly, a QSAR study for the binding affinity to the corticosteroid-binding globulin using Cramer’s steroid dataset is carried out.

**Conclusions:**

From these analyses, it is revealed that the QuBiLS-MAS approach for atom-pair relations yields similar-to-superior performance with regard to other QSAR methodologies reported in the literature. Therefore, the QuBiLS-MAS approach constitutes a useful tool for the diversity analysis of chemical compound datasets and high-throughput screening of structure–activity data.

**Electronic supplementary material:**

The online version of this article (doi:10.1186/s13321-017-0211-5) contains supplementary material, which is available to authorized users.

**Graphical abstract:**
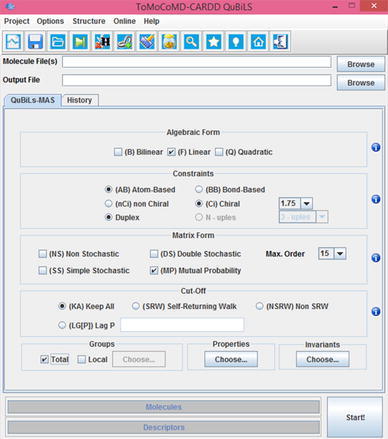
.

## Background

The codification of chemical information using mathematical–computational methods to accelerate small-molecule drug discovery constitutes one of the fundamental tasks of mathematical chemistry [1, 2]. In recent years, the number and diversity of molecular features, also known as molecular descriptors (MDs), has significantly increased and corresponding educational and commercial computational implementations developed [3–9]. The absence of an ultimate universal chemical descriptor emphasizes the need of defining alternative methods to codify relevant and orthogonal chemical information.

In previous reports, Marrero-Ponce et al. proposed algebraic formalisms for characterizing topological (2D) and chiral (2.5D) molecular features through atom- and bond-based ToMoCoMD-CARDD (acronym for Topological Molecular Computational Design-Computer Aided Rational Drug Design) molecular descriptors [10–13]. These MDs codify molecular information based on the bilinear, quadratic and linear algebraic forms and the *graph*-*theoretical electronic*-*density and edge*-*adjacency matrices* in order to consider atom- and bond-based relations, respectively. The ToMoCOMD-CARDD MDs have been successfully applied in the screening of chemical compounds of different therapeutic applications ranging from antimalarials [14], trichomonacidals [15, 16], antitrypanosomals [17], paramphistomicides [18], antibacterials [19], tyrosinase inhibitors [20, 21] and others [22, 23]. To compute these descriptors, a computational program with the same name was developed. However, this software barely offered the functionalities required in contemporary molecular modeling tasks, in addition to the inherent limitations that made its usability impractical, for instance: (a) it did not support standard input formats (i.e. MDL MOL/SDF files) and the only input method for the chemical structures entailed the sketching of molecular pseudographs using a built-in manual drawing mode; (b) parameter configurations could not be exported or saved for posterior experiments; (c) no option for batch processing of descriptors was offered; (d) lacked the distributed computing functionality which permits the correct utilization of current multi-core architectures; (e) could not be used as a standalone library thus preventing the its integration in other applications; and (f) presented ambiguities in the labeling of the descriptors’ names in the output file.

In addition, in several mathematical procedures employed to compute MDs (e.g. GT-STAF [24, 25], DIVATI [26] and QuBiLS-MIDAS [27–30]), the molecules are not analyzed as a whole, that is, these are partitioned in order to univocally characterize each atom independently. In this way, several mathematical operators (also known as *aggregation operators*) may be applied over the atom-level indices to compute different global/local MDs. The use of several aggregation operators is based on the idea that the most suitable global definition of a system may not necessarily be additive. In fact, it is reported in the literature that operators other than the sum could yield better correlations with determined chemical properties [24–28]. In this sense, in the present report strategies are defined to generalize the procedure of obtaining global or local QuBiLS-MAS (acronym for Quadratic, Bilinear and N-Linear mapS based on graph-theoretic electronic-density Matrices and Atomic weightingS) indices using the so-called aggregation operators. Moreover, several new atom-based properties, chemical local-fragments (e.g. terminal methyl groups), distance-based cut-offs (for the analysis of the most important non-covalent or covalent interactions) and probabilistic transformations of the matrix representations are introduced. Furthermore, initiatives to deal with the computational and practical limitations inherent to the original ToMoCoMD-CARDD program were carried out, with the ultimate goal of improving its applicability in present-day cheminformatics tasks.

## Theoretical scaffold: past and present

### Brief history of algebraic maps-based indices

The algebraic forms-based topological MDs (0–2.5D) are divided into three main families: *quadratic, bilinear* and *linear* indices [12, 31, 32]. They are distinguished in atom-based [33] and bond-based indices [10] depending on whether they are derived from the atom-based or bond-based matrix, respectively. The main diagonal elements for the atom-based matrix [denominated as non-stochastic (NS) when it doesn’t involve any normalization procedure] describe the presence of loops on graph vertices, which are used to characterize atoms in conjugated systems having more than one canonical structure [31, 34]. Thus, the elements for the *k*th *non*-*stochastic pseudograph*-*theoretic electronic*-*density matrix* $({\boldsymbol{\mathcal{M}}}_{ns}^{k} )$ are labeled as ${}^{k}m_{ij}$ and defined as follows:

1 $${}^{k}m_{ij} = \left\{ {\begin{array}{*{20}ll} {P_{ij} } \hfill & if\,i\,\ne\,j\,{\bigwedge }\,\exists\,e_{ij}{:}\;e_{ij} \in \varvec{E} \hfill \\ {L_{ij} } \hfill & if\,i = j \,{\bigwedge }\,\exists\,e_{ij}{:}\;e_{ij} \in \varvec{E} \hfill \\ 0 \hfill & {otherwise} \hfill \\ \end{array} } \right.$$

where, $i$ and $j$ represent two vertices (atoms) of the molecular pseudograph G, *k* is the matrix power, $\varvec{E}$ is the set of edges of G, $P_{ij}$ is the number of edges $(e_{ij} )$ between the atoms $i$ and $j$ (e.g. $P_{ij} = 3$ for a triple covalent bond between *i* and *j*), and $L_{ij}$ is the number of loops in $v_{i}$ [12, 13, 31, 33, 35, 36]. Likewise, the coefficients corresponding to the bond-based matrix, ${\boldsymbol{\mathcal{E}}}_{ns}^{\varvec{k}} ,$ may be defined. In this way, the entries $e_{vw}$ belonging to ${\boldsymbol{\mathcal{E}}}_{ns}^{\varvec{k}}$ are equal to 1 if the edge $v$ shares a common vertex with the edge $w$ [37, 38]. Moreover, the NS matrix may be normalized by means of the *simple stochastic* (SS) procedure [10], yielding matrices whose row or column coefficients are non-negative real numbers which sum up to 1. This mathematical procedure has been explained in detail elsewhere [13, 18, 39]. Let us take a simple example of the *isonicotinic acid* structure, and consider its corresponding labeled molecular pseudograph and atom-based matrix [31]. Table 1 shows the non-stochastic (NS) matrix for the *isonicotinic acid* structure for *k* = 0, 1, 2.

**Table 1:**
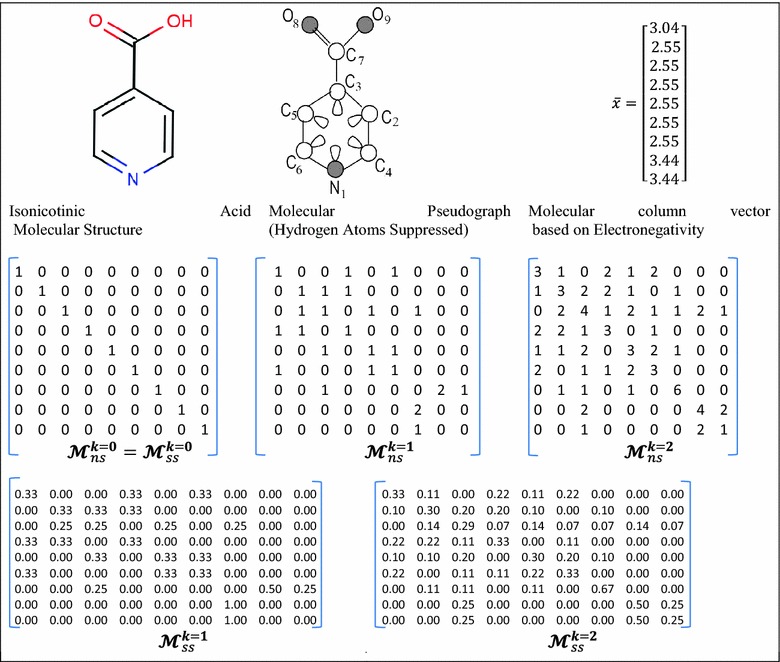
The molecular structure and the atom adjacency stochastic (ss) and non-stochastic (ns) matrices for the *k* = 0, 1, 2 corresponding to the *Isonicotinic Acid*

To compute the algebraic form-based indices, the molecular vector concept is employed, which uses atom-based properties as weighting schemes. Thus, atomic properties (e.g. mass, polarizability, electronegativity according to Pauling’s scale and Van der Waals volume) may be considered [11, 12]. In this way, the molecular structures may be represented as vectors. For instance, the *Isonicotinic Acid* molecule may be represented by the molecular vector $\bar{x} = \left[ {x_{N1} ,x_{C2} ,x_{C3} ,x_{C4} ,x_{C5} ,x_{C6} ,x_{C7} ,x_{O8} ,x_{O9} } \right]$, where $\bar{x} \in {\mathbb{R}}^{9}$ (i.e. considering an H-atoms suppressed molecular graph). Table 1 shows the Pauling electronegativity-based molecular vector for *Isonicotinic acid*. The weighting scheme for the bond-based molecular vector is built with values computed from the properties corresponding to the atoms that each bond connects [10, 13, 20, 40]:

2 $$w_{ij} = \frac{{w_{i} }}{{\delta_{i} }} + \frac{{w_{j} }}{{\delta_{j} }}$$

where, $w_{ij}$ constitutes the weighting scheme computed for the edge $e_{ij} ,\,w_{i}$ and $w_{j}$ are the atomic weights (e.g. electronegativity) for atoms *i* and *j* forming the considered bond $(e_{ij} )$, $\delta_{i}$ and $\delta_{j}$ are the corresponding vertex degrees which also account for bond multiplicity. Moreover, in order to codify information on the 3D structure of the molecule, a *trigonometric 3D*-*chirality correction factor* is applied to the molecular vectors aforementioned, which has been comprehensively explained in several reports [40–42].

From the previous molecular vectors and matrix formalisms, the algebraic calculation of the NS and SS total (whole-molecule) bilinear indices may be condensed in the following equations, for atom- (see Eq. 3) and bond-based (see Eq. 4) indices, respectively:

3 $${}^{ns, ss}b^{k} \left( {\bar{x},\bar{y}} \right) = \mathop \sum \limits_{i = 1}^{n} \mathop \sum \limits_{j = 1}^{n} m_{ij}^{k} x^{i} y^{j} = \left( {\bar{x}} \right)^{T} \times {\boldsymbol{\mathcal{M}}}_{ns, ss}^{\varvec{k}} \times \bar{y}\quad \forall k = 1,2, \ldots ,15$$

4 $${}_{e}^{ns,ss} b^{k} \left( {\bar{x},\bar{y}} \right) = \mathop \sum \limits_{i = 1}^{m} \mathop \sum \limits_{j = 1}^{m} e_{ij}^{k} x^{i} y^{j} = \left( {\bar{x}} \right)^{T} \times {\boldsymbol{\mathcal{E}}}_{ns, ss}^{\varvec{k}} \times \bar{y}\quad \forall k = 1,2, \ldots ,15$$

where, *n* (or *m*) is the number of atoms (or bonds) in the molecule, *k* = 1, 2, …15 is the matrix power, $m_{ij}^{k}$ (or $e_{ij}^{k}$) represents the elements of the ${\boldsymbol{\mathcal{M}}}_{{\varvec{ns},\varvec{ss}}}^{\varvec{k}}$ (or ${\boldsymbol{\mathcal{E}}}_{{\varvec{ns},\varvec{ss}}}^{\varvec{k}}$) non-stochastic (*ns*) and simple stochastic (*ss*) matrices, and $x^{i}$ and $y^{j}$ are the elements of the $\bar{x}$ and $\bar{y}$ atom-based (or bond-based) property vectors. On one hand, when the vectors $\bar{x}$ and $\bar{y}$ encode the same atomic property (i.e. $\bar{x} = \bar{y}$), the Eqs. 3 and 4 define the NS and SS total atom-based and bond-based quadratic indices, respectively. On the other hand, if $\bar{x}$ is a vector with all entries equal to 1 and $\bar{y}$ an atom/bond-based property vector, then the Eqs. 3 and 4 define the NS and SS total atom-based and bond-based linear indices, respectively.

In addition, local-fragment (group or atom-type) quadratic, bilinear and linear atom/bond-based indices can be defined to characterize a predetermined molecular fragment (*F*) instead of the whole molecule (total indices). These are computed using the *k*th local-fragment matrix ${}_{F}{\boldsymbol{\mathcal{M}}}^{k}$ $({}_{F}{\boldsymbol{\mathcal{E}}}^{k} )$, which is computed from the corresponding *k*th total matrix ${\boldsymbol{\mathcal{M}}}^{k}$ (${\boldsymbol{\mathcal{E}}}^{\varvec{k}}$) considering only those vertices (or edges) belonging to the selected molecular fragment. These fragments *F* may be heteroatoms (X), halogens (G) and H-bond donors (N or O atoms sharing a bond with an H-atom, labeled as D) [10, 34, 36]. Thus, NS and SS local-fragment atom/bond-based bilinear, quadratic and linear indices can be computed using the ${}_{F}{\boldsymbol{\mathcal{M}}}^{k}$ and $_{F} {\boldsymbol{\mathcal{E}}}^{k}$ local-fragment matrices instead of the corresponding total matrices in the Eqs. 3 and 4.

It is important to remark that for each partitioning of a molecule into Z molecular exclusive fragments, there will be Z local-fragment matrices. In this case, if a molecule is partitioned into Z molecular fragments, then the original *k*th power of matrix ${\boldsymbol{\mathcal{M}}}_{{\varvec{ns},\varvec{ss}}}^{k}$ (or ${\boldsymbol{\mathcal{E}}}_{{\varvec{ns},\varvec{ss}}}^{k}$) is exactly the sum of the *k*th power of the local-fragment matrices. Consequently, the total algebraic form-based indices are the sum of the exclusive contributions of the respective local-fragment algebraic form-based indices, as long as there is not overlap among the fragments. Therefore, taking into consideration the previous elements, the next sections address in detail the improvements related with the mathematical definition corresponding to the 2D algebraic indices introduced by Marrero-Ponce et al. [10, 31, 32, 43, 44].

### The QuBiLS-MAS MDs: new definitions, generalization and extension of algebraic indices

As previously explained, up to date, the 2D atom/bond-based algebraic indices have been computed as whole-molecule (total) indices or from specific chemical groups (local indices), where the simplest fragment could be the atom itself, known as a LOcal Vertex Invariant (LOVI) and in case of a bond as LOcal Edge Invariant (LOEI). In this manuscript the LOVEIs term is adopted to refer both LOVIs and LOEIs of a molecule, and is denoted as ${\boldsymbol{\mathcal{L}}}$. Therefore, if a molecule is comprised of *n* atoms or *m* bonds then the *kth total bilinear, quadratic and linear* indices for each atom “*a*” (known as *total atom*-*level index*) or each bond “*e*” (known as *total bond*-*level index*) may be computed as two-linear algebraic forms (maps) in ${\mathbb{R}}^{n}$, in a canonical basis set, and whose values are components (entries) of the vector ${\boldsymbol{\mathcal{L}}}$ denoted as ${\boldsymbol{\mathcal{L}}}_{a}$ and ${\boldsymbol{\mathcal{L}}}_{e}$ for atom- and bond-level indices, respectively. In this way, the kth total atom-level and bond-level bilinear indices are mathematically defined as follows, respectively:

5 $${{}_{b}}{\boldsymbol{\mathcal{L}}}_{a} = b^{a,k} \left({\bar{x},\bar{y}} \right) = \mathop \sum \limits_{i = 1}^{n} \mathop \sum \limits_{j = 1}^{n} \fancyscript{m}_{ij}^{a,k} x^{i} y^{j} = \left({\bar{x}} \right)^{T} \times {\boldsymbol{\mathcal{M}}}^{a,k} \times \bar{y}\quad \forall a = 1,2, \ldots,n$$

6 $${}_{b}{\boldsymbol{\mathcal{L}}}_{e} = b^{e,k} \left( {\bar{x},\bar{y}} \right) = \mathop \sum \limits_{i = 1}^{m} \mathop \sum \limits_{j = 1}^{m} {\text{e}}_{ij}^{e,k} x^{i} y^{j} = \left( {\bar{x}} \right)^{T} \times {\boldsymbol{\mathcal{E}}}^{e,k} \times \bar{y}\quad \forall e = 1,2, \ldots ,m$$

where *x* ^1^, …, *x* ^*n*(*m*)^ and *y* ^1^, …, *y* ^*n*(*m*)^ are the coordinates or components of the molecular vectors $\bar{x}$ and $\bar{y}$ [45]. To compute these molecular vectors the following atomic properties have been selected: (1) atomic mass, (2) the Van der Waals volume, (3) the atomic polarizability, (4) atomic electronegativity according to Pauling scale, (5) atomic Ghose–Crippen LogP, (6) atomic Gasteiger–Marsili charge, (7) atomic polar surface area, (8) atomic refractivity, (9) atomic hardness and (10) atomic softness. These properties are calculated using the CDK library [9]. Note that when $\bar{x} = \bar{y}$ atom- and bond-level quadratic indices are obtained [i.e. ${}_{q}{\boldsymbol{\mathcal{L}}}_{a} = q^{a,k} \left( {\bar{x},\bar{x}} \right)$ and ${}_{q}{\boldsymbol{\mathcal{L}}}_{e} = q^{e,k} \left( {\bar{x},\bar{x}} \right)$], while if all coefficients of $\bar{x}$ are equal to 1 then linear indices for atoms (or bonds) may be obtained [i.e. ${}_{f}{\boldsymbol{\mathcal{L}}}_{a} = f^{a,k} \left( {\bar{u},\bar{y}} \right)$ and ${}_{f}{\boldsymbol{\mathcal{L}}}_{e} = f^{e,k} \left( {\bar{u},\bar{y}} \right)$].

The coefficients ${\fancyscript{m}}$_*ij*_^*a*,*k*^ (see Eq. 5) are the elements corresponding to the kth NS (or SS) total atom-level pseudograph-theoretic electronic-density matrix [NS(SS)-GEDM] ${\boldsymbol{\mathcal{M}}}^{a,k}$ for atom “*a*”, while the entries $e_{ij}^{e,k}$ (see Eq. 6) belonging to kth NS (or SS) total bond-level edge-adjacency matrix [NS(SS)-EAM] ${\boldsymbol{\mathcal{E}}}^{e,k}$ for bond “*e*”. These atom/bond-level coefficients are obtained from the entries ${\fancyscript{m}}$_*ij*_^*k*^ of the ${\boldsymbol{\mathcal{M}}}^{k}$ total matrix and $e_{ij}^{k}$ of the ${\boldsymbol{\mathcal{E}}}^{k}$ total matrix, respectively, using the described procedure to compute local-fragment matrices but considering the fragment *F* as an atom “a” or bond “e” of the molecule. Moreover, the diagonal coefficients ${\fancyscript{m}}$_*ii*_^1^ could have two distinct values in order to achieve greater discrimination of molecular structures: (1) aromatic ring sensibility for setting up aromatic atoms hooked on full aromatic rings instead of mapping individual atom loops as shown in the molecular pseudograph of the Table 1, and/or (2) the number of lone-pairs for each atom. The $e_{ii}^{1}$ entries are always zero.

It is important to highlight that as an extension of the former ToMoCoMD 2D-MDs several local-fragments have been aggregated: H-bond acceptors (A), carbon atoms in aliphatic chains (C), H-bond donors (D), halogens (G), terminal methyl groups (M), carbon atoms in an aromatic portion (P) and heteroatoms (X). Thus, from these local-fragments the kth NS (or SS) local-fragment atom-level pseudograph-theoretic electronic-density matrices $_{F} {\boldsymbol{\mathcal{M}}}^{a,k}$ for atom “a” and the kth NS (or SS) local-fragment bond-level edge-adjacency matrices $_{F} {\boldsymbol{\mathcal{E}}}^{e,k}$ for bond “e”, may be computed. Consequently, local-fragment atom- and bond-level bilinear, quadratic and linear indices are determined from the Eqs. 5 and 6 using $_{F} {\boldsymbol{\mathcal{M}}}^{a,k}$ and $_{F} {\boldsymbol{\mathcal{E}}}^{a,k}$ as matrix forms, respectively. Note that the coefficients $_{F} \fancyscript{m}_{ij}^{a,k} \in\,{_{F} {\boldsymbol{\mathcal{M}}}^{a,k}}$ and $_{F} e_{ij}^{e,k} \in\,{_{F} {\boldsymbol{\mathcal{E}}}^{e,k}}$ are calculated from the elements $_{F} \fancyscript{m}_{ij}^{k} \in\,{_{F} {\boldsymbol{\mathcal{M}}}^{k}}$ and $_{F} e_{ij}^{k} \in\,{_{F} {\boldsymbol{\mathcal{E}}}^{k}} ,$ respectively.

In addition, two normalization procedures are introduced as novel extensions. The atom-based simple stochastic scheme defined in the original ToMoCoMD 2D-MDs [18, 39, 43] describes changes in the electron distribution over time throughout the molecular backbone. This SS matrix is not symmetrical and the probability for atom *i* to interact with atom *j* is different from the probability for the atom *j* to interact with the atom *i*. Therefore, with the aim of balancing the probabilities in both senses a double-stochastic (DS) matrix is employed, that is, a matrix with real non-negatives entries whose column and row sums are equal to one. In this way, the kth total (or local-fragment) DS graph-theoretical electronic-density (DS-GEDM, $_{(F)} {\boldsymbol{\mathcal{M}}}_{{\varvec{ds}}}^{k}$) and edge-adjacency (DS-EAM, $_{(F)} {\boldsymbol{\mathcal{E}}}_{{\varvec{ds}}}^{k}$) matrix approaches can be calculated from the corresponding ${\boldsymbol{\mathcal{M}}}_{ns}^{\varvec{k}}$ and ${\boldsymbol{\mathcal{E}}}_{ns}^{\varvec{k}}$ matrices, respectively, using the Sinkhorn–Knopp algorithm [46]. Additionally, the kth total (or local-fragment) mutual probability (MP) graph-theoretical electronic-density matrix (MP-GEDM, $_{(F)} {\boldsymbol{\mathcal{M}}}_{mp}^{k}$) and edge-adjacency matrix (MP-EAM, $_{(F)} {\boldsymbol{\mathcal{E}}}_{{\varvec{mp}}}^{k}$) are introduced. The mutual probability matrices are obtained dividing each entry between the total sum of their elements, in this way, symmetrical matrices where the total sum is equal to 1 are obtained. The Scheme 1 shows the steps followed in the computation of the NS-, SS-, DS- and MP-GEDMs, while Tables 2 and 3 illustrate the calculation of these matrices with and without taking in consideration the lone-pair electrons.

**Schema 1:**
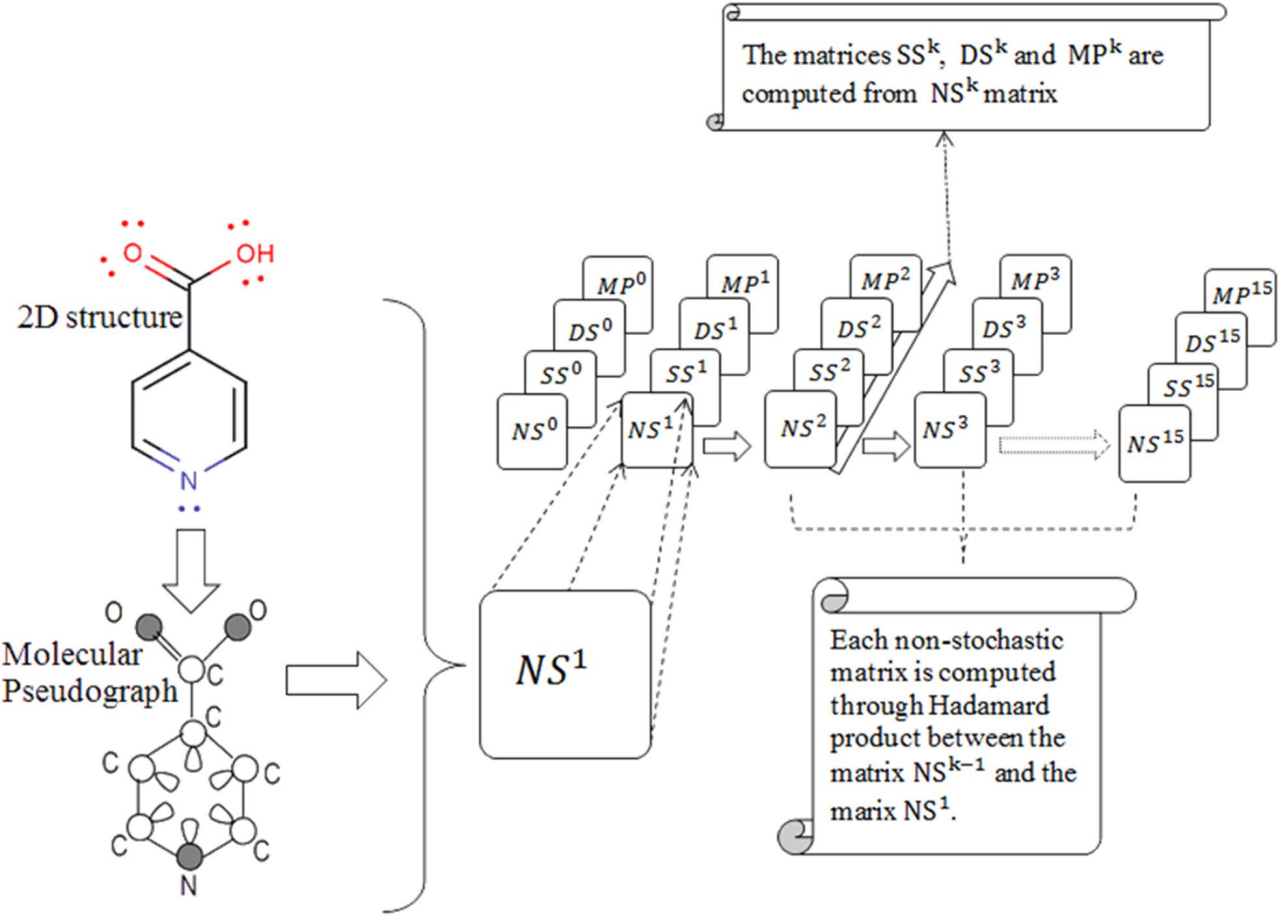
The stages involved in the computation of the NS-, SS-, DS-, and MP-pseudograph-theoretical electronic-density matrices

**Table 2:**
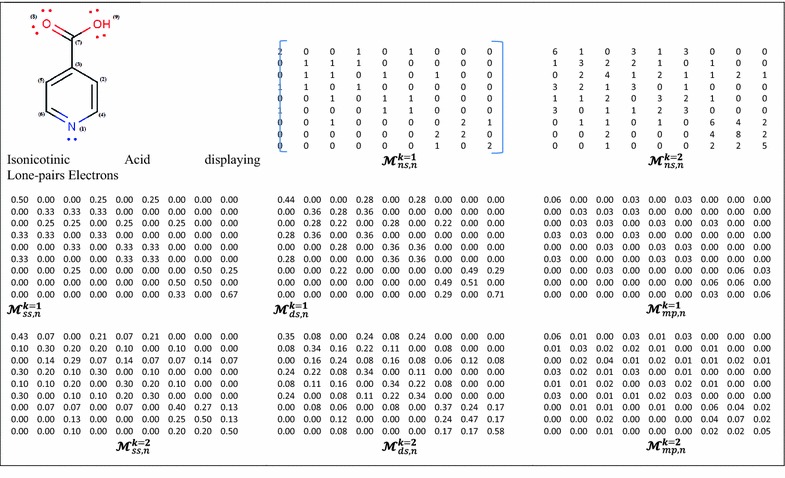
The molecular structure considering lone-pair electrons (*n*) for the first and second powers of the molecular pseudograph’s atom adjacency mutual probability (*mp*), non- (*ns*), double (*ds*)- and stochastic (*ss*) matrices for *Isonicotinic Acid*

**Table 3:**
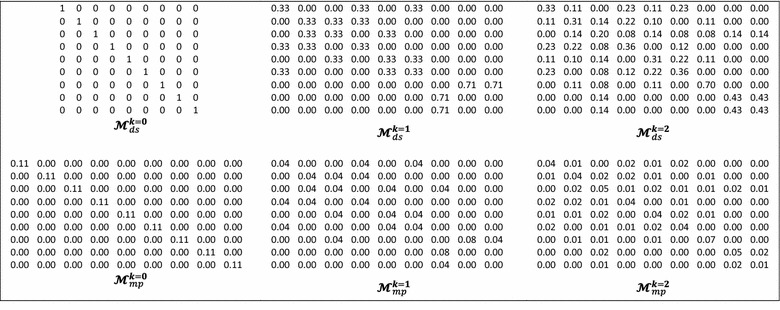
The zero, first and second powers of the molecular pseudograph’s atom adjacency double stochastic and mutual probability matrices for *Isonicotinic Acid*

Lastly, in order to obtain the global kth total (or local-fragment) bilinear, quadratic and linear indices from the corresponding atom-level (${\boldsymbol{\mathcal{L}}}_{a}$) or bond-level (${\boldsymbol{\mathcal{L}}}_{e}$) definitions, the summation operator is used. The global indices obtained using this operator over components of vector ${\boldsymbol{\mathcal{L}}}$ coincide with those indices calculated through the original procedure vector–matrix–vector detailed in Eqs. 3 and 4. Note that the summation operator is equivalent to the Manhattan norm applied to elements of the vector ${\boldsymbol{\mathcal{L}}}$ relative to the origin, which is in turn a specific case of Minkowski norm when *p* = 1. Motivated by this understanding, a generalization in which different *p* values are used, i.e. *p* = 2 and 3, where the former (p = 2) is the Euclidean norm (see Additional file 1: Figure SI1 for geometrical interpretation) was introduced. Additionally, other operators (see Additional file 1: Table SI2) applicable to the vector of LOVEIs were applied with the aim of generalizing the use of the linear combination to obtain global indices. It has been demonstrated in several reports [24–28] that better correlations for bioactivities may be attained when operators other than the sum are employed.

#### Neighborhood topological constraints in the graph-theoretical electronic-density and edge-adjacency matrix

The $_{(F)} {\boldsymbol{\mathcal{M}}}^{k}$ and $_{(F)} {\boldsymbol{\mathcal{E}}}^{k}$ matrices contain information on the connectivity for all atoms and bonds that constitute a molecule, respectively. However, some biological properties do not depend on the chemical structure as a whole but rather on interactions at particular topological distances, for example, short-, middle- and large-range contacts. Thus, with the aim of considering interactions that satisfy specific topological criteria, three graph-theoretical constraints (*cut*-*offs*) are introduced: (1) keeping only the diagonal elements of the matrix, denoted as “Self-Returning Walks” (SRW), (2) keeping only the off-diagonal elements of the matrix, denoted as “Non-Self-Returning Walks” (NSRW), and (3) keeping only the elements within a given interval, based on the topological distance for a *path cut*-*off*, denoted as *Lag p*.

The application of these cut-offs over the matrices $_{(F)} {\boldsymbol{\mathcal{M}}}^{k}$ and $_{(F)} {\boldsymbol{\mathcal{E}}}^{k}$ yields the following representations: the Self-Returning Walks matrices (i.e. ${}_{{\left( \varvec{F} \right)}}^{srw} {\boldsymbol{\mathcal{M}}}^{k}$ and ${}_{{\left( \varvec{F} \right)}}^{srw} {\boldsymbol{\mathcal{E}}}^{k}$), the non-Self-Returning Walks matrices (i.e. ${}_{{\left( \varvec{F} \right)}}^{nsrw} {\boldsymbol{\mathcal{M}}}_{{}}^{k}$ and $\varvec{ }{}_{{\left( \varvec{F} \right)}}^{nsrw} {\boldsymbol{\mathcal{E}}}^{k}$), and the topological path cut-off matrices (i.e. ${}_{{\left( \varvec{F} \right)}}^{p} {\boldsymbol{\mathcal{M}}}^{k}$ and ${}_{{\varvec{ }\left( \varvec{F} \right)}}^{p} {\boldsymbol{\mathcal{E}}}^{k}$), respectively. The coefficients ${}_{{\left( \varvec{F} \right)}}^{\varvec{p}} m^{1}$ and ${}_{{\left( \varvec{F} \right)}}^{\varvec{p}} e^{1}$ belonging to these last matrices, respectively, are defined as follows:

7 $${}_{{\left(\varvec{F} \right)}}^{\varvec{p}} \fancyscript{m}^{k} \left[{{}_{{\left(\varvec{F} \right)}}^{\varvec{p}} e^{k}} \right] = \left\{{\begin{array}{*{20}l} {_{{(\varvec{F})}} \fancyscript{m}^{k} \left[{_{{(\varvec{F})}} e^{k}} \right]} \hfill &\quad if\,p_{min}\,\le\,p_{ij}\,\le\,p_{max} \hfill \\ 0 \hfill &\quad {otherwise} \hfill \\ \end{array}} \right.$$

where, $p_{ij}$ is a user-defined topological distance threshold, and *min* and *max* are the minimum and maximum cut-off values (rank). Table 4 shows an illustrative example where three topological constraints are calculated for an atom-level matrix. A custom *cut*-*off* allows to distinguish the interaction types, for example, when a topological graph-theoretical cut-off is applied, then atomic indices could be calculated for atoms separated by 1 step (covalent interactions) or for those atoms separated by more than 1 step ($p \ge 2$). The present approach could be viewed as a threshold that generalizes the use of *lag p* in 2D-Moreau–Broto autocorrelations [1]. Likewise, these matrices based on cut-offs may be employed to determine the corresponding atom-level and bond-level representations to be used in the calculation of QuBiLS-MAS 2D-MDs. In Scheme 2, a complete workflow to compute the QuBiLS-MAS indices is represented.

**Table 4:**
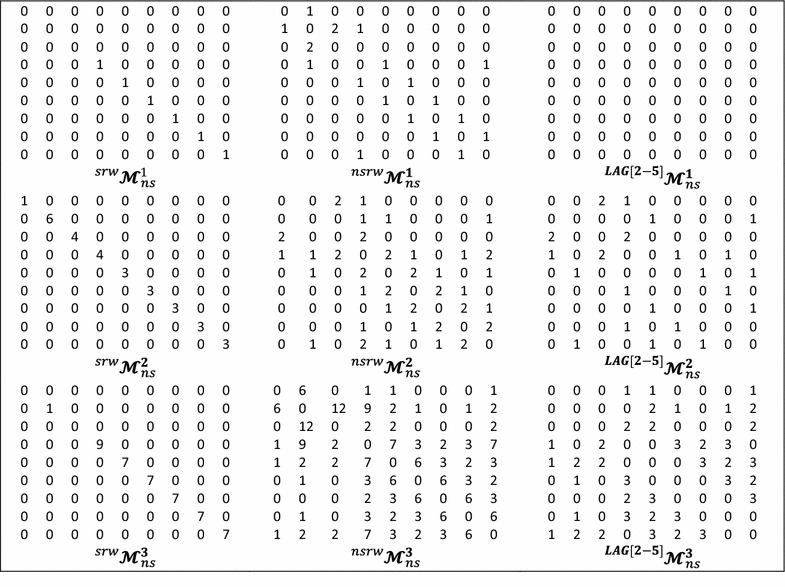
First, second and third order NS—matrices for *Isonicotinic Acid*, obtained by applying three types of topological constraints (*cut*-*off*): Self-Returning Walks (SRW), Non-Self-Returning Walks (NSRW) and a topological *path cut*-*off* distance from 2 to 5 (LAG [2–5])

**Schema 2:**
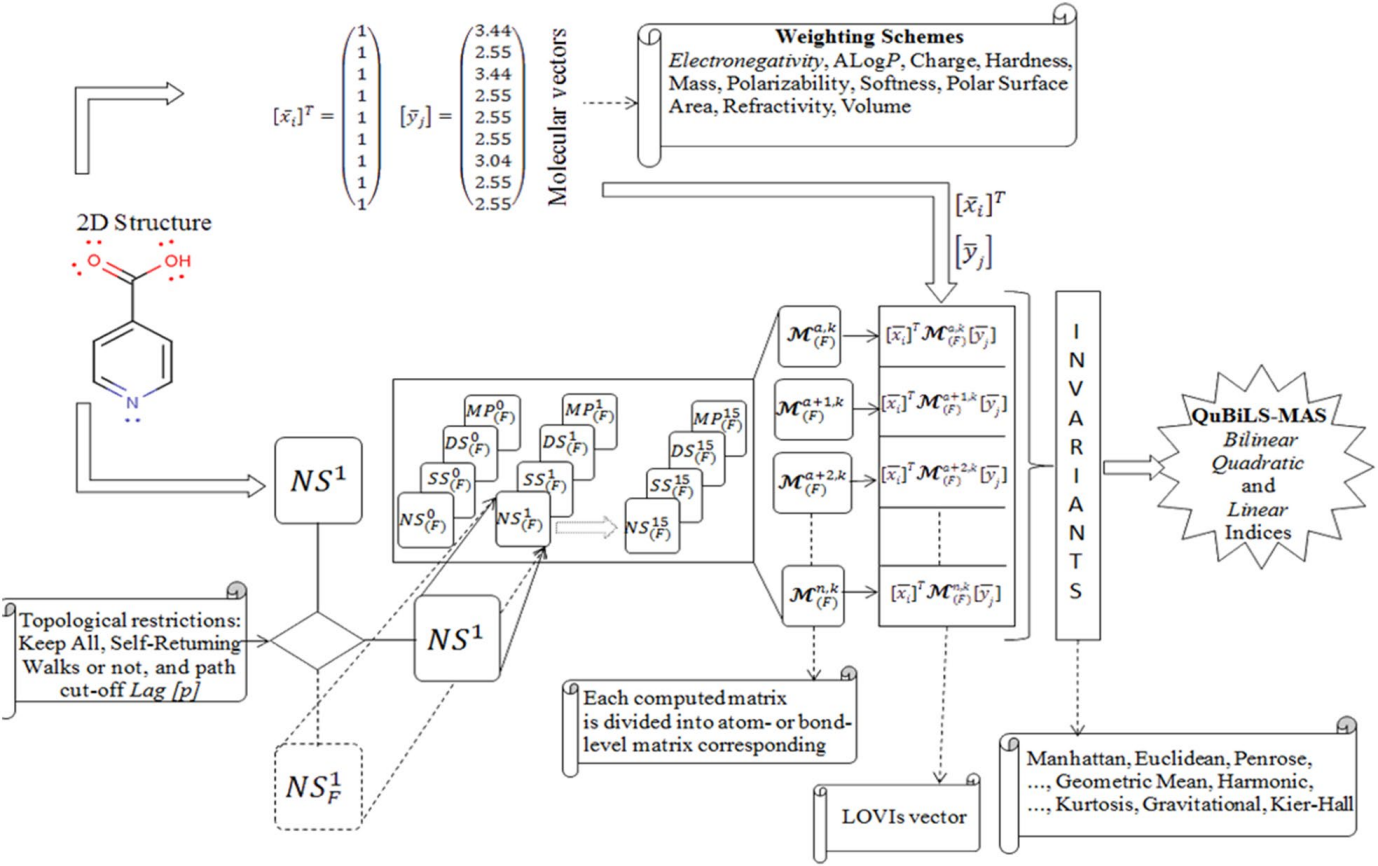
Workflow followed in the computation of the ToMoCoMD-CARDD QuBiLS-MAS MDs

## The QuBiLS-MAS module

The QuBiLS-MAS module was designed as standalone software, with the extensions and generalizations discussed in “The QuBiLS-MAS MDs: new definitions, generalization and extension of algebraic indices” section. This software was developed in Java v1.8 and the Chemistry Development Kit (CDK) library (version 1.4.19) [9] was used in the manipulation of the chemical structures, as well as in determining the atom- and fragment-based chemical properties involved in the calculation process. The QuBiLS-MAS software is comprised of a *front*-*end* and *back*-*end*. The *front*-*end* is composed of a desktop and command-line user interface, while the *back*-*end* is developed as an Abstract Programming Interface (API) to enable its use as an independent Java library in the development of other cheminformatics applications or in the implementation of other user-friendly interfaces either graphical or command-line based. With these two components, independence between the software presentation layer and the processing logic implemented in the *back*-*end* is achieved and thus, any modification in the latter does not provoke changes in the *front*-*end* (GUI), and vice versa.

### Back-end: the QuBiLS-MAS molecular descriptors library-computational complexity of algorithms

All the requests performed by the users through the GUI are processed by the QuBiLS-MAS library. This component is structured in packages according to the goals of the functionalities (see Additional file 1: Figure SI3 for UML diagram). The main package is *tomocomd.cardd.qubils* which contains the packages *descriptors*, *matrices*, *metrics* and *workers* that encapsulate the main concepts utilized in the definition of the QuBiLS-MAS MDs. The *descriptors* package includes the classes related to the calculation of the *total and local*-*fragment bilinear*, *quadratic* and *linear algebraic maps*. The *matrices* package contains the objects responsible for building the *pseudograph*-*theoretic electronic*-*density matrix* and the *edge*-*adjacency matrix* corresponding to atom- and bond-based representations, respectively. Additionally, the simple-stochastic, double-stochastic and mutual probability normalization strategies, as well as the topological constraints (*cut*-*offs*) are defined in this package. The *tools* package includes classes for the identification of the local-fragments, as well as the considered aggregation operators. Lastly, the *workers* package comprises the classes for the configuration and control of the algebraic MDs calculation process.

The algorithms responsible for performing the multiplication based on bilinear, quadratic and linear algebraic forms constitute the principal procedures to compute the QuBiLS-MAS indices. This procedure consists of a loop that iterates for each atom of the molecule to determine the corresponding atom- or bond-level matrix. Next the atom/bond-level matrices are multiplied by the corresponding property vectors in order to obtain the atom/bond-level indices. The corresponding sequential implementations have a computational complexity of $O(n^{3} ).$ Nonetheless, when the atom/bond-level matrices are computed according to the mentioned procedure, it is noted that the only entries with values different from zero correspond to the atom with respect to which the atom/bond-level matrix is built. Therefore, instead of iterating for each atom in order to build the atom/bond-level matrix used posteriorly to determine the corresponding index, it is more suitable to compute the atom/bond-level indices at the same time as the original matrix is analyzed. Taking this into account, the algorithms have been optimized to an inferior polynomial order, achieving a complexity of $O(n^{2} )$ in the computation of the atom/bond-based contributions for the QuBiLS-MAS indices.

### Graphic user interface of the QuBiLS-MAS software

**Fig. 1:**
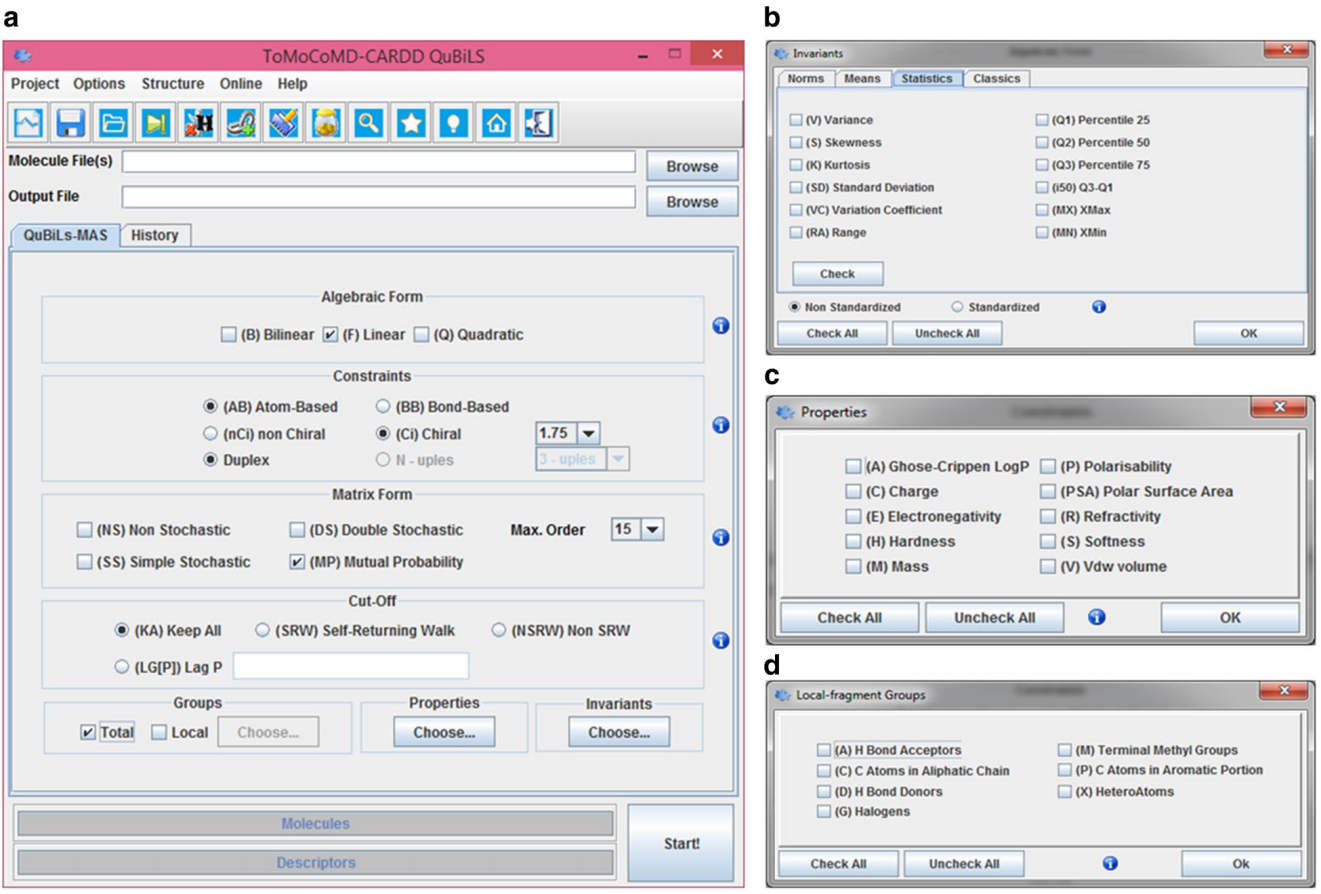
Main graphic user interface for QuBiLS-MAS software (**a**) and dialog windows to configure the following parameters: invariants or aggregation operators (**b**), atom properties (**c**) and local-fragment chemical groups (**d**)

To facilitate the calculation of the QuBiLS-MAS MDs, a friendly Desktop GUI was developed in order to provide a simple and intuitive way to configure the different parameters used, such as: algebraic forms, matrix approaches, atomic properties, topological cut-offs and so on. Figure 1 shows the main GUI and the dialog windows designed to configure some of these parameters. These configuration sections allow the users to personalize the *bilinear, quadratic and linear indices* according to their necessities and thus predefined MDs are not calculated.

In the “Algebraic Form” panel, the specific algebraic maps to be used in the computation of the MDs are chosen according to the selected option in the “Constraints” panel, which could be a*tom*-*based* or *bond*-*based*. Also, chirality detection may be configured in the “Constraints” panel. The matrix normalization formalisms (MP, NS, SS, and DS) used in the algebraic forms are configured in the “Matrix Form” panel, as well as the maximum order (*k* value) to which the coefficients of the matrices are raised. In the “Cut-Off” panel the option to “keep all” (KA) atomic interactions is selected by default, but other options [i.e. “Self-Returning Walks” (SRW), “Non-Self-Returning Walks” (NSRW) and/or the value-rank(s) of threshold *p*] may be considered to take into account only the non-covalent interactions according to the established criterion. The “Local-Fragments” *panel* contains the options to configure the seven chemical groups (or atom-types) that may be employed to compute either the total or local-fragment indices. Likewise, in the “Properties” panel the atomic properties used to setup different weighting schemes are chosen. Finally, the mathematical operators used to compute the global total or local indices from the atomic contributions are selected in the “Invariants” panel.

It is important to highlight that the selected options to compute the descriptors can be exported into an XML configuration file, called the *project file*, which can be used to calculate the same QuBiLS-MAS indices for other datasets when the software is run again. Another important feature is that the software can be executed on computer clusters using a command-line interface, which uses the *project files* to obtain the configuration of the indices to be computed. Also, the QuBiLS-MAS software has incorporated the “On/Off H-Atoms” option to consider (or not) the H-atoms during the calculation, the “On/Off Lone-Pair Electron” option to consider (or not) the number of lone-pairs for heteroatoms and the “Show Debug Report” option to track the algebraic processes that take place during the calculation (see Additional file 1: SI4).

The supported input file format for the chemical structures to be analyzed is the MDL MOL/SDF format and these are sequentially read in order to employ suitable memory allocation according to the size of the molecule. Moreover, the path of the output file may be specified where the values of the computed MDs are saved. To this end, the QuBiLS-MAS software supports the following output file formats: CSV, ARFF, and TXT (space- and tab-separated ASCII format) which are easily interpretable in popular statistical and/or machine learning software.

The calculation procedure is monitored in real time through the main interface and controlled with the interactive mode of the GUI. Indeed, more than one *project file* can be calculated over different datasets. This is a feature implemented in the QuBiLS-MAS software encapsulated into a batch processing module, which is useful for carrying out high-throughput and routine MD calculations. This module is designed to manage the configuration of up to eight independent tasks (see Additional file 1: SI5), where each task consists of one or several datasets for which one or several *projects files* previously saved with the QuBiLS-MAS GUI may be computed. Finally, a module for chemical structure curation tasks was incorporated, taking into account Tropsha’s guidelines [47]. Table 5 shows a comparison between the old [48] ToMoCOMD software and the present one (QuBiLS-MAS module), highlighting the numerous functionalities incorporated. Table 6 compares the characteristics for common molecular descriptor calculating software and including the QuBiLS-MAS program, specifying the respective strengths and weaknesses.

**Table 5:** Comparison between the old software (TOMOCOMD) and the new one proposed in this report (QuBiLS-MAS)

Features	Computer program	
	TOMOCOMD	QuBiLS-MAS
*Description level*		
Theoretical		
Algebraic form maps	3 (quadratic, bilinear and linear)	
Atom and Bond level	Yes	Yes
Matrices	2 (NS, SS)	4 (NS, SS, DS, MP)
Atom Weightings	4 (M, V, P, E)	10 (M, V, P, E, A, C, PSA, R, H, S)
Local-fragments	3 (D, G, X)	7 (A, C, D, G, M, P, X)
Chirality	YES, ${\mathfrak{c}}$ = ±1	YES, extended to ${\mathfrak{c}}$ = ±0.25 to ±3 with a 0.25 step
Lone-pair electrons	–	Yes
Topological constraints	–	Yes, three cut-off types (SRW, NSRW, Lag P)
H-atoms consideration	–	Yes, permits inclusion or removal
Invariants or aggregation operators	–	Yes, 21 aggregation operators classified in four major groups
Computational		
Open source	–	Yes, under LGPL
Availability	Shareware	Freeware
Programming language	Borland Delphi	Java
Clear Object-oriented source code design	–	Yes
Canonical namespace packages structure	–	Yes, under *com.tomocomd.qublis.*
Target operating system(OS)	Microsoft Windows	Platform-independent
Graphical user interface	Yes	Yes
Command line	–	Yes
Portable MDs library	–	Yes, as pre-compiled Java *JAR* file
Supported input format	In-house file format	mol/sdf MDL
Output format	Text File (TSV)	Text File (TSV, SSV, CSV), Weka (ARFF)
Structure curation and cleaning	–	Yes, available under *Structure* menu item (with 10 check/cleaning tasks, H-atoms handling, and function for chemical formats conversion)
Built-in example data	–	Yes, six chemical datasets
Unique MD header	–	Yes, identifying the codification scheme
Batch Processing mode	–	Yes
Parallelized computing	–	Yes, using the Fork/Join framework
Configurable projects	–	Yes
Import/export configuration	–	Yes, using a XML file format
Calculation progress	–	Yes, for descriptors and molecules
Real-time memory monitor	–	Yes, with garbage collection option when desired
Events logging	–	Yes, accessible through the *History* Tab
Calculation report	–	Yes
Runtime help accessibility	–	Yes
User owner’s manual	–	Yes
Online webpage	–	Yes http://www.tomocomd.com/qubils

*Matrices* Non-stochastic (NS), simple stochastic (SS), double stochastic (DS) and mutual probability (MP). *Atom weightings* (*atomic properties*) (1) atomic mass (M), (2) the Van der Waals volume (V), (3) the atomic polarizability (P), (4) atomic electronegativity according to Pauling scale (E), (5) atomic Ghose–Crippen LogP (A), (6) atomic Gasteiger–Marsili charge (C), (7) atomic polar surface area (PSA), (8) atomic refractivity (R), (9) atomic hardness (H), and (10) atomic softness (S). *Local-fragments* (*atom-type and/or group-type*) H-bond acceptors (A), carbon atoms in aliphatic chains (C), H-bond donors (D), halogens (G), terminal methyl groups (M), carbon atoms in an aromatic portion (P) and heteroatoms (X). *Chirality* trigonometric 3D-chirality correction factor (${\mathfrak{c}}$). *Topological constraints* (*cut-offs*) (1) keeping only the diagonal elements of the matrix, denoted as “Self-Returning Walks” (SRW), (2) keeping only the offdiagonal elements of the matrix, denoted as “Non-Self-Returning Walks” (NSRW), and (3) keeping only the elements within a given interval, based on the topological distance for a path cut-off, denoted as Lag p

**Table 6:** Main features of commonly used tools for molecular descriptors (MDs) calculations

Software	Number of types of MDs	Configuration of MDs parameters	Advantages	Disadvantages	Additional remarks and online reference
QuBiLS-MAS v1.0	2080 (linear, quadratic and bilinear)	1. Atom- or Bond-Based	1. Computes MDs based on algebraic maps	1. Only accepts MDL files (MOL or SDF) as input formats	1. Uses CDK to read molecular files and calculate atomic properties
		2. Atomic properties	2. 10 atom weighting schemes		2. Requires Java JRE 1.7 or above http://www.tomocomd.com
		3. Local-fragments	3. Graphic user-friendly interface and command-line interface		
		4. Matrix approaches	4. Platform-independency		
		5. Aggregation operators	5. Supports any organic molecules		
		6. Add (or remove) hydrogen atoms	6. Free download and support		
		7. Consider lone-pair electrons	7. Batch mode processing		
			8. Data cleaning module		
			9. Parallel processing		
PaDEL-Descriptor v2.0	43	None	1. Graphic user interface	1. One functionality for data cleaning tasks (remove salts)	1. Uses CDK to read molecular files and calculate most of the descriptors and fingerprints
			2. Fully cross-platform	2. No MDs batch processing	2. Employs Java Web Start technology
			3. Command line interface		
			4. Free and Open Source		
			5. Accepts multiple file formats (>90 formats)		
			6. Parallel processing		
DRAGON v6.0	29	1. Predefined atom weighting schemes	1. Graphic user-friendly interface	1. Only Windows and Linux platforms	Academic permanent license: 900 euros (to be installed on 3 PCs)
		2. Selection of single molecular descriptors included in the different blocks	2. Command line interface	2. No parallel processing	http://www.talete.mi.it/products/dragon_description.htm
			3. Batch mode processing	3. No data cleaning functionalities	
			4. Supports any organic molecules	4. Does not allow selection of local-fragments	
			5. Accepts the formats: MDL, Sybyl, HyperChem, Macromodel, Smiles, CML and HyperChem	5. Commercial cost	
CDK Descriptor Calculator v1.3.9	48	1. Add (or remove) hydrogen atom	1. Graphic user interface	1. Only accepts MDL files (MOL or SDF) as input formats	Use CDK library and requires JRE 1.6
			2. Command line execution	2. No data cleaning functionalities	http://www.rguha.net/code/java/cdkdesc.html
			3. Fully cross-platform	3. Does not allow selection of local-fragments	
			4. Free software	4. Does not allow selection of atom weighting schemes	
			5. Batch mode processing		
BlueDesc	36	None	1. Free and Open Source	1. No graphic user interface	Use CDK and JOELib2 library and requires Java JRE 1.6
			2. Fully cross-platform	2. Only accepts MDL files (MOL or SDF) as input formats	http://www.ra.cs.uni-tuebingen.de/software/bluedesc/welcome_e.html
				3. No parallel processing	
				4. No data cleaning functionalities	
				5. Does not allow selection of local-fragments	
				6. Does not allow selection of atom weighting schemes	
Model	98	None	1. Web-based graphic user interface	1. No parallel processing	Use of MODEL for commercial purposes is not allowed
			2. Accepts the formats: PDB, MDL, MOL2,COR	2. No data cleaning tasks	http://jing.cz3.nus.edu.sg/cgi-bin/model/model.cgi
				3. Does not allow selection of local-fragments	
				4. Does not allow selection of atom weighting schemes	
				5. For academic purposes only	
Mol2	20	None	1. Command line interface	1. No graphic user interface	http://www.fda.gov/ScienceResearch/BioinformaticsTools/Mold2/ucm144528.htm
			2. Free of charge download request	2. Only Windows platform	
				3. Only accepts SDfile format	
				4. No parallel processing	
				5. No data cleaning functionalities	
				6. Does not allow selection of local-fragments	
				7. Does not allow selection of atom weighting schemes	
MOE	–	None	1. Graphic user interface	1. Only accepts SDfile format	http://www.chemcomp.com/MOE-Cheminformatics_and_QSAR.htm
			2. Command line interface	2. No parallel processing	
			3. Data cleaning tasks	3. Does not allow selection of local-fragment	
			4. Fully cross-platform	4. Does not allow selection of atom weighting schemes	
VolSurf	22	None	1. Graphic user interface	1. Commercial	http://www.moldiscovery.com/soft_volsurf.php
			2. Command line interface	2. Only Linux platform	
			3. Accepts several formats: MDL SDF, Sybyl, Mol2, Multi Mol2, GRID kout	3. Only compute 2D MDs	
				4. No parallel processing	
				5. Does not allow selection of local-fragment	
				6. Does not allow selection of atom weighting schemes	
Adriana. Code	5	None	1. Graphic user interface	1. Commercial	A demo version is available on request free of charge
			2. Command line interface	2. Only Windows and Linux platforms	http://www.molecular-networks.com/products/adrianacode
			3. Batch mode processing	3. No parallel processing	
			4. Accepts any organic molecule	4. No data cleaning functionalities	
			5. Several input and output formats	5. Does not allow selection of local-fragment	
				6. Does not allow selection of atom weighting schemes	
CODESSA PRO	8	None	1. Graphic user interface	1. Commercial	http://www.codessa-pro.com/
				2. Only for Windows platform	
				3. No parallel processing	
				4. No batch mode processing	
				5. Does not allow selection of local-fragment	
				6. Does not allow selection of atom weighting schemes	
PowerMV	–	None	1. Graphic user interface	1. Only for Windows platform	Requires Microsoft.Net 1.1 or above
				2. No parallel processing	http://nisla05.niss.org/PowerMV
				3. No batch mode processing	
				4. Does not allow selection of local-fragment	
				5. Does not allow selection of atom weighting schemes	
Molconn-Z v4.10	79		Multi-platform SGI Irix, Linux, Solaris, Mac OS-X and Windows. 12 months free Support	No GUI, Commercial	Minimum price US$750 for a Single Educational Node/User license
					http://www.edusoft-lc.com/molconn
Pre ADMET Descriptor	34		GUI, Free web-based Limited application and Commercial PC version. Maintenance and Upgrade free of charge	Commercial. Runs on Windows. Only accepts MDL files (MOL or SDF) as input formats	Requires Microsoft.NET Framework 2.0 and minimum price is US$1 000 for 1 year Academic license
					http://preadmet.bmdrc.org
Toxicity Estimation Software Tool (T.E.S.T.) v4.1	13 (628)		GUI, Open source and multi-platform	Platform specific distributions. Only accepts MOL or SMILES as input formats	Based on CDK library. Requires Java JRE 1.6
					http://www.epa.gov/ordntrnt/ORD/NRMRL/std/qsar/qsar.html
ADAPT	27		Non-Commercial	Runs on Unix. Heavy-atom limitations up to 255 atoms. Only accepts MOL as input formats	Written in Fortran and is installed on a DEC alpha workstation
					http://research.chem.psu.edu/pcjgroup/adapt.html
ChemAxon Calculator Plugins *v5.11*	12	27	Free for non-commercial, freely accessible web pages	s	http://www.chemaxon.com/marvin/help/calculations/calculator-plugins.html
			GUI, Batch execution from command line		
			Multi-platform Windows, HP, MacOS X, Solaris and Linux		
JOELib2		40	Free, Open Source, Redistributable. Multi-platform		http://www.ra.cs.uni-tuebingen.de/software/joelib/introduction.html
*TOPS*-*MODE &* MODes Lab		Several (mainly edge-based) topological indices	GUI	Runs on Windows	http://www.modeslab.com/
			Non-Commercial	No Batch execution	

## Assessment of the performance of the QuBiLS-MAS descriptors

### Information content analysis based on Shannon’s entropy

Shannon’s entropy (SE) quantifies the information content codified by molecular indices, according to the principle that variables that effectively discriminate all molecules in a dataset possess high entropy values, while redundant variables have low entropy values. To perform this study, the Spectrum dataset (http://www.msdiscovery.com/spectrum.html) comprised by 1963 structures was used. The highest SE for this dataset is equal to 10.93 bits (log_2_N, where N is the number of compounds). In the following subsections the novel QuBiLS-MAS 2D-MDs are analyzed taking into account the proposed internal generalizations, as well as with respect to well-known MDs computed by other software. For this study, the IMMAN software was used [49].

#### Comparative variability analysis according to the matrix formalisms

**Fig. 2:**
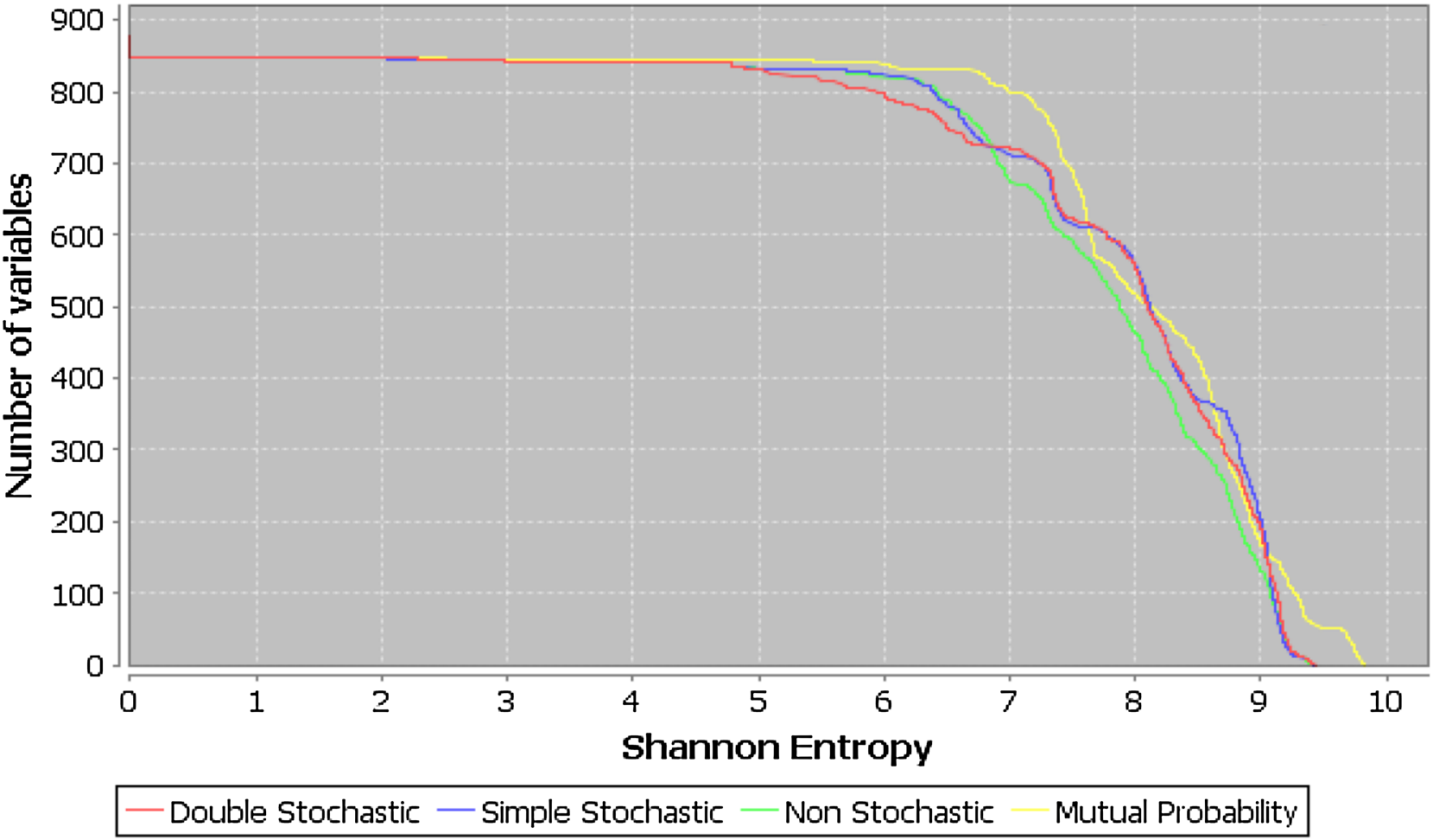
In-house comparison of Shannon’s entropy distribution for the QuBiLS-MAS 2D-Indices considering the non-stochastic, simple stochastic, double-stochastic and mutual probability matrix formalisms

The four matrix schemes defined in the present report are analyzed. To this end, 880 MDs are calculated for each matrix. Figure 2 shows similar entropy distributions for the non-, double- and simple-stochastic matrix approaches, while the best behavior is obtained with the mutual probability approach. The superior performance of the mutual probability formalism with respect to the other three matrix transformations justifies the theoretical contribution of this scheme in the computation of the QuBiLS-MAS 2D-MDs.

#### Analysis of variability according to the aggregation operators

**Fig. 3:**
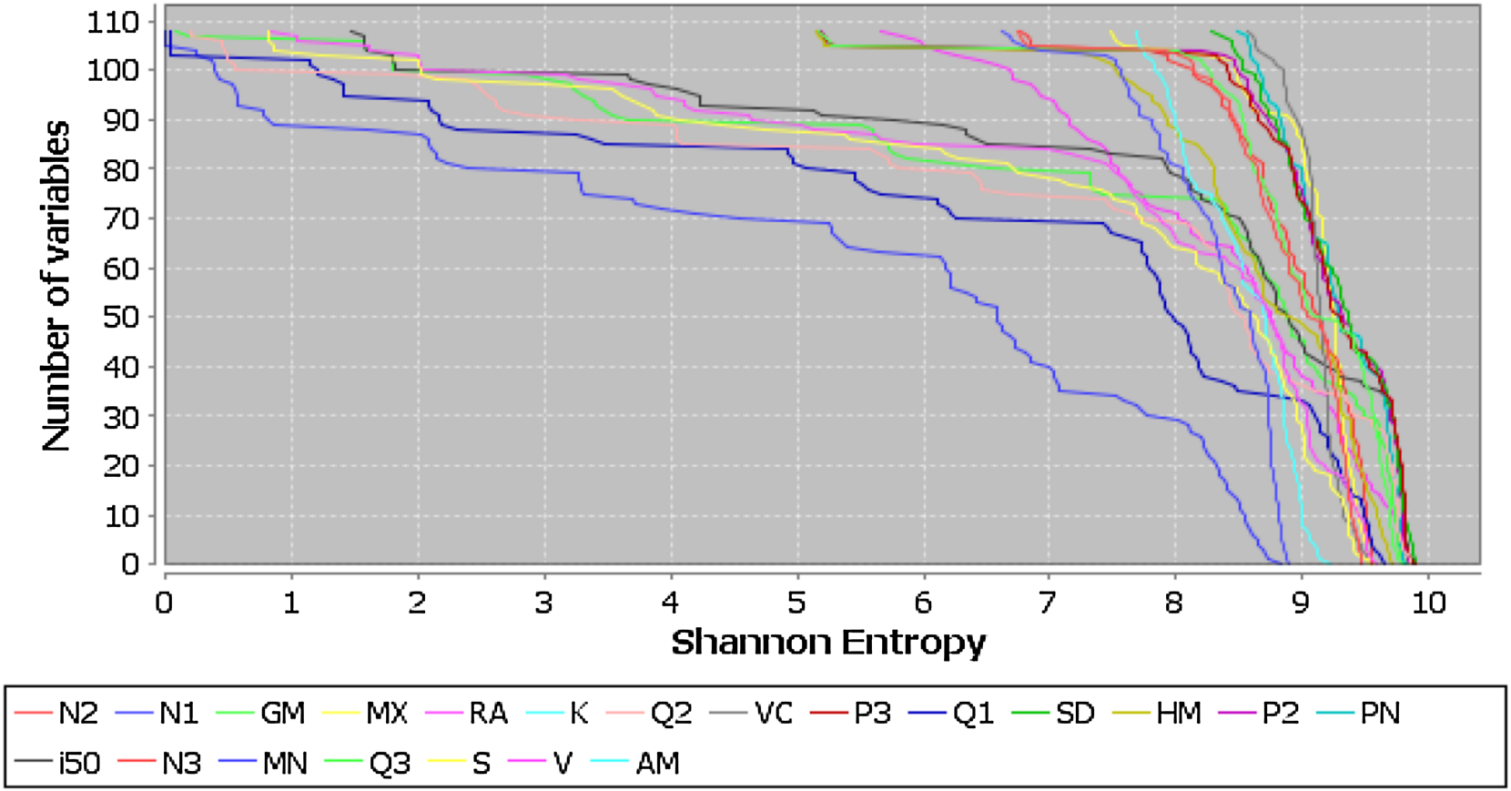
In-house comparison of Shannon’s entropy distribution for the QuBiLS-MAS 2D-Indices considering the norms, the statistical operators of central tendency and the operators for dispersion and form

The aim of this section is to evaluate the variability of the QuBiLS-MAS 2D-indices according to the mathematical operators used over the vector of LOVEIs. In this study, the aggregation operators classified as norms, means and statistical invariants are compared. To this end, 110 atom-based linear indices for each operator were calculated and the results are shown Fig. 3. As it can be noted, the best results are achieved by the Potential Mean, Quadratic Mean and Standard Deviation operators with 71, 67, 66 and 65% of the total variables having entropy values greater than 9.0 bits (82% of the maximum entropy), respectively. Moreover, the indices based on the Manhattan (sum of LOVEIs) and Minimum operators present the worst performance, while the remaining distributions have similar behavior. This result suggests that the generalization of the linear combination of LOVIEs to consider other aggregation operators yields variables with greater information content, and thus, it should contribute to a greater modeling capacity for the QuBiLS-MAS MDs.

#### Variability analysis of QuBiLS-MAS 2D-indices versus DRAGON descriptor families

The purpose of this analysis is to compare the entropy of the QuBiLS-MAS 2D-MDs with the DRAGON descriptor families. To perform this study some DRAGON descriptor-blocks were clustered into bigger families: (1) *0D_others* for molecular properties, constitutional and charge descriptors, (2) *1D*-*fragment* for functional group counts and atom-centered fragments, (3) *2D*-*conn_autocorr_inf* for 2D autocorrelations, connectivity and information indices, (4) *2D*-*edge_walk* for edge adjacency indices, walk and path counts, (5) *2D*-*eigenvalues* for Burden eigenvalues, topological charge and eigenvalues-based indices, and (6) *3D*-*Randic_geometrical* for Randic molecular profiles and geometrical descriptors. The remaining DRAGON families were kept with the same denominations. The maximum number of descriptors considered for each family is 91, which corresponds to the *0D_others* family that has the least number of MDs.

**Fig. 4:**
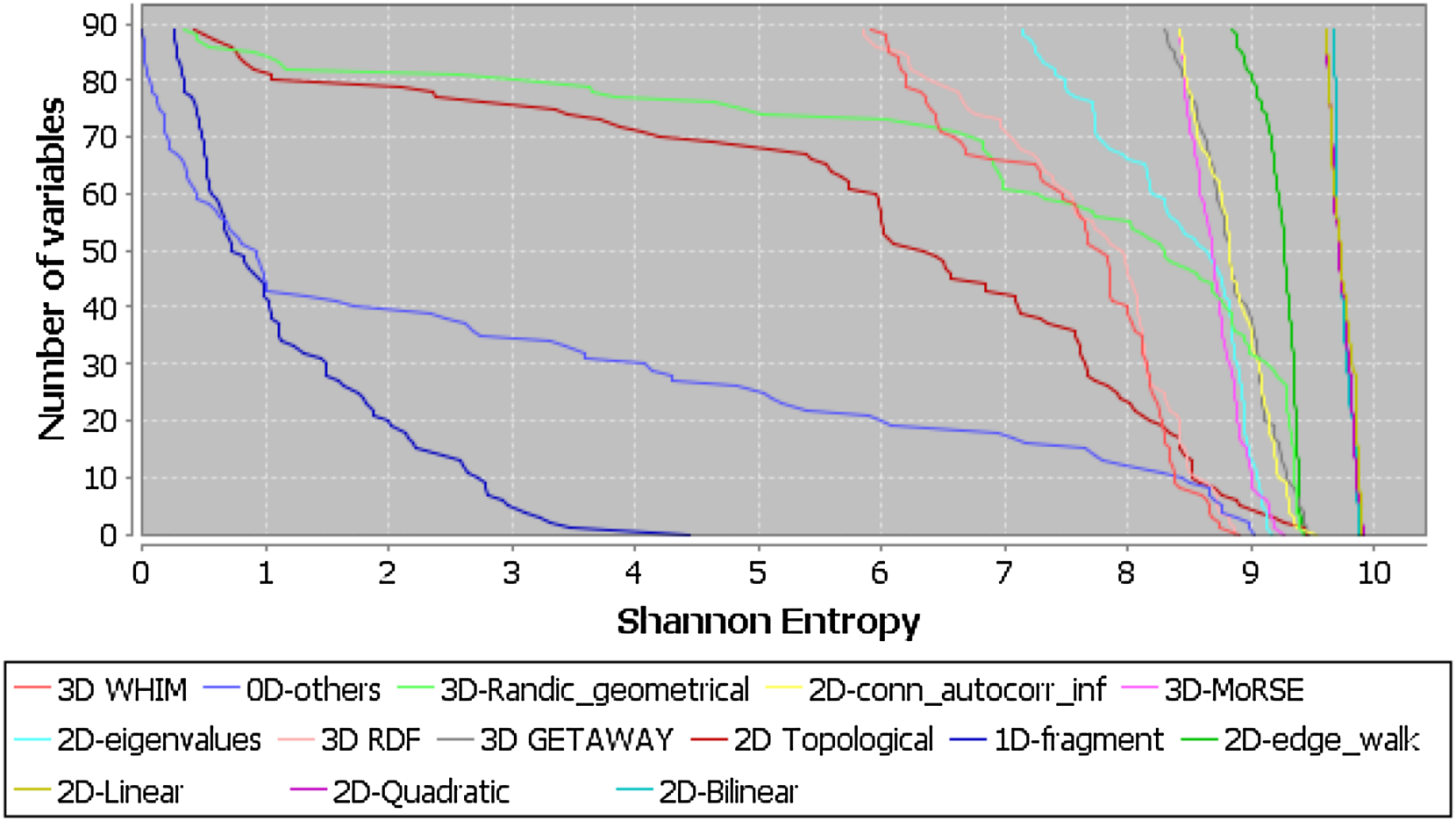
Shannon’s entropy distribution for DRAGON MDs families versus bilinear, linear and quadratic QuBiLS-MAS 2D-Indices

As it can be observed in Fig. 4, the QuBiLS-MAS 2D-MDs show the best overall performance with all the considered indices presenting entropy values above 9.55 bits (87% of the maximum entropy). As for the DRAGON MD families, the *2D*-*edge_walk*, 3D-GETAWAY and *2D*-*conn_autocorr_inf* indices show the best behavior with 63, 21 and 15 variables presenting SE values greater than 8.70 bits (80% of the maximum entropy), respectively, although all these distributions are inferior to the one corresponding to the QuBiLS-MAS 2D-indices. This is a promising result bearing in mind that the DRAGON MD families are obtained from a diverse range of theoretical and practical considerations, encompassing over 30 years of research.

#### Variability comparison for QuBiLS-MAS 2D-indices with respect to other descriptor computing software

**Fig. 5:**
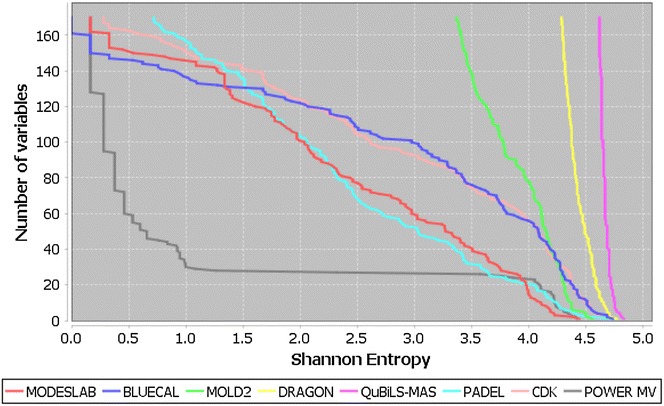
Shannon’s entropy distribution for QuBiLS-MAS topological indices and other descriptors computed by well-known software used in cheminformatics studies

The variability distribution of the QuBiLS-MAS MDs was computed and compared to MDs calculated with other programs used in cheminformatics tasks, such as: DRAGON [3], MOLD2 [4], PADEL [7], _ENREF_70 CDK Descriptor Calculator [9], MODESLAB [50], BLUECAL [51] and POWER MV [52]. To this end, the DRAGON’s example data comprising 42 structurally diverse chemicals was used. The cut-off number of variables for this study was 170 MDs, determined by the BLUECAL software as it possesses the least number of indices. As it can be observed in Fig. 5, the QuBiLS-MAS topological indices achieve superior performance than other software considered, with the former presenting all its values above 4.62 bits [86% of the maximum entropy ($log_{2} 41 = 5.35$)], while the indices of the remaining approaches practically have all their indices inferior to this threshold. The high entropy distribution obtained for the QuBiLS-MAS topological indices demonstrates the relevance of these MDs, in the sense that they are sensitive to progressive structural modifications and should therefore be valuable in different cheminformatics tasks.

### Linear independence of the QuBiLS-MAS algebraic descriptors

In this section, the possible orthogonality of the QuBiLS-MAS 2D-Indices with respect to the DRAGON 0D-2D MDs is examined, using the Principal Component Analysis (PCA) [53, 54]. The PCA is a mathematical technique that converts several correlated variables into a reduced number of non-correlated variables, called principal components. The extracted components have the following features: (1) the first component will explain the highest possible variance of all determined components, (2) the successive components will explain the variance that the previous components did not explain, and (3) variables loaded in each component are linearly independent to the ones loaded in the remaining components. For all the studies performed in this section, the curated Spectrum Collection dataset (1963 molecules) was employed.

To perform this analysis, two sets of descriptors were calculated using QuBiLS-MAS MDs and the DRAGON (824 MDs) software, respectively, with the latter comprising of the following families: *0D*-*others* (B01 Constitutional, B19 Charge and B20 Molecular Properties) with 91 indices, *1D*-*fragment* (B17 Functional Groups Counts and B18 Atom-centered Fragments) with 274 indices, *2D*-*conn_autocorr_inf* (B04 Connectivity, B05 Information and B06 2D-AutoCorrelations) with 176 indices, *2D*-*edge_walk* (B03 Walk-Path Counts and B07 Edge Adjacency) with 154 indices, *2D*-*eigenvalues* (B08 Burden, B10 Eigenvalue-based and B09 Topological Charge) with 129 indices, and finally the B02 2D Topological with 119 indices.

In this analysis, 12 principal components were selected, which explain approximately 74.60% of the cumulative variance (see Additional file 1: SI6 and Additional file 1: SI7). As it can be observed, Factors 1 (27.83%), 2 (13.06%), 8 (2.47%) and 9 (1.99%) exhibit strong loadings for some QuBiLS-MAS indices and some 0D–2D descriptors of the DRAGON software. On the other hand, exclusive loadings are obtained for the QuBiLS-MAS descriptors in the Factors 3 (8.6%), 4 (6.26%), 5 (3.86%), 6 (3.51%), 7 (2.71%), 11 (1.42%) and 12 (1.20%), explaining 27% of the total variance. Factor 10 (1.62%) is important for some 0–2D DRAGON MDs as these are exclusively loaded in this factor, and these indices include: TI2 (B02 2D Topological), PW2 (B02 2D Topological), RBF (0D–others) and EEig01r (2D-edge_walk) [for details on these descriptors, see Additional file 1: SI8]. On the whole, much of the information codified by the 0D-2D DRAGON MDs is equally captured by the QuBiLS-MAS indices, considering that negligible variance (1.62%) is explained by the factor exclusive for the former (F10). Moreover, the numerous factors (i.e. F3, F4, F5, F6, F7, F11 and F12) exclusive for the QuBiLS-MAS MDs suggest that orthogonal information is codified and thus demonstrating the theoretical contribution of the generalization schemes adopted in this framework.

### QSAR modeling of the binding affinity to corticosteroid binding globulin (CBG) of Cramer’s steroid dataset

In what follows, the predictive ability of the QuBiLS-MAS approach is assessed. To accomplish this objective, QSAR models for predicting the “binding affinity to the corticosteroid-binding globulin (CBG) of the popular Cramer’s steroid database” (see Additional file 1: SI9 for names and CGB values of compounds) were built. This dataset has been used as a “benchmark” to evaluate the quality of novel procedures. A total of 1455 variables were computed for each algebraic form (quadratic, bilinear and linear maps). The prediction models were built using Multiple Linear Regression (MLR) as the fitting method, coupled with the Genetic Algorithm (GA) as variable subset selection strategy and the statistical parameter Q_loo_^2^ (“leave-one-out” *cross validation*) as the fitness function. Throughout the study, regression models of 2–6 variables were developed and the best model in each case retained for posterior validation. The GA was setup with the following configurations: population size—100, crossover/mutation rate—0.7, selection operator was fixed at 60 and the number of iterations—500,000. In addition, the *tabu list* option was configured to remove those MDs with correlation equal or greater than 0.95. The MLR-GA based model building was performed using the MobyDigs [55] computer program. The best models built were also assessed with the bootstrapping [56] $(Q_{boot}^{2} )$ and Y-scrambling [57] $(a (Q^{2} ))$ validation methods in order to assess the predictive power and the possible chance correlation with respect to the activity modeled.

#### Examination of matrix formalisms

**Fig. 6:**
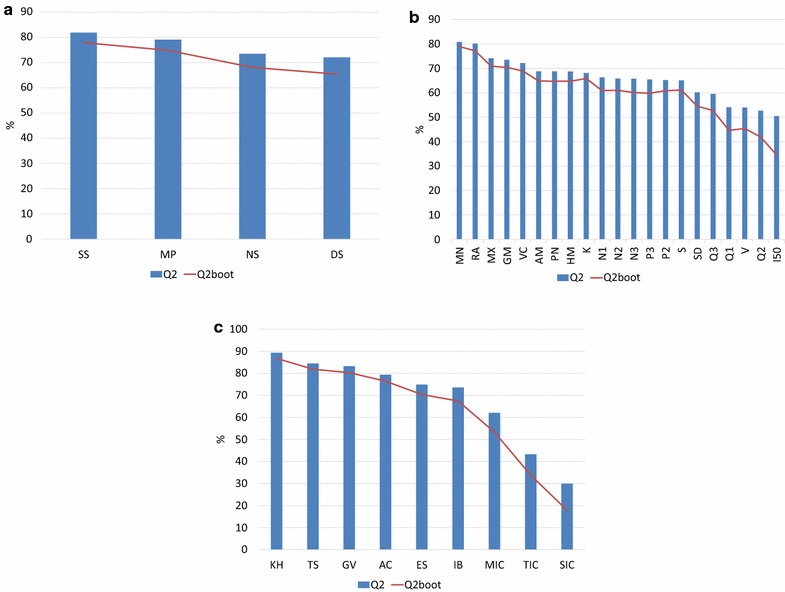
Comparison of the performance of some inner features of the QuBiLS-MAS software in QSAR modeling: **a** the matrix formalisms, **b** the aggregation operators and **c** the classical algorithms

In order to assess the performance of the NS, SS, DS and MP matrix-based approaches in QSAR modeling, 46 variables for each formalism were calculated. Figure 6a shows the statistical parameters achieved in this experiment, where the SS approach (Q_loo_^2^ = 81.85%, Q_boot_^2^ = 77.89%) presents the best behavior, followed by MP (Q_loo_^2^ = 79.05%, Q_boot_^2^ = 74.85%). The indices based on NS (Q_loo_^2^ = 73.48%, Q_boot_^2^ = 68.09%) and DS (Q_loo_^2^ = 72.01%, Q_boot_^2^ = 65.4%) matrices present a much lower performance. This result is in agreement with the variability analysis, where the highest entropy indices involved the SS and MP matrix formalisms.

#### Analysis of the aggregation operators

The following study evaluates the predictive power of the aggregation operators proposed as a generalization scheme for the linear combination of LOVEIs as method for obtaining global (or local) indices. As it can be observed in Fig. 6b, all Q_loo_^2^ values are superior to 50%, with the best performances corresponding to the statistical operators, followed by the mean operators and lastly by the norms. Regarding the evaluation of the operators classified as “classical algorithms” (Fig. 6c) it is observed that Kier–Hall (KH), Total Sum (TS), Gravitational (GV) and Autocorrelation (AC) algorithms yield comparable to superior performance with respect to the remaining operators. It may therefore be concluded that the incorporation of the aforementioned generalization scheme improves the performance of the QuBiLS-MAS indices in modeling tasks and thus demonstrating its practical contribution.

#### The QuBiLS-MAS MDs versus literature reports

To evaluate the earnest contribution of the QuBiLs-MAS approach, it is necessary to assess its performance in correlation studies with determined molecular properties and compare the results with the existing methods. Different QSAR models for predicting the binding affinity to CBG of the 31 structures of Cramer’s steroid database (1–31 or also 1–30 with compound 31 as outlier) have been reported in the literature, which will be compared here with the models obtained using the QuBiLs-MAS 2D-MDs. In this experiment, the best 3–5 variable models were selected according to the quality of the statistical parameters Q_loo_^2^ and Q_boot_^2^. Table 7 shows the best regression models and their corresponding statistical parameters, based on the QuBiLs-MAS 2D-indices. Comparisons with other QSAR methodologies reported in the literature are presented in Table 8 according to the Q_loo_^2^ statistic.

**Table 7:** Statistical parameters for the best models for 2–6 variables for the physicochemical property log K, considering the 31 structures as the training set

Size	*R* ^2^	*Q*_loo_^2^	*Q*_boot_^2^	a (*Q* ^2^)	F	Models	Equations
2	0.778	0.734	0.738	−0.208	49.16	log K = 1.596 (±0.885) + 3.809 (±0.582)	(19)
						TS[1]_MX_B_AB_nCi_2_SS12_T_KA_a-h − 0.118 (±0.011)	
						KH[1]_MX_F_AB_nCi_2_MP2_T_KA_h	
3	0.863	0.826	0.820	−0.259	57.14	log K = −32.132 (±3.841) − 75.624 (±9.789)	(20)
						TS[1]_RA_F_AB_nCi_2_MP2_T_KA_h + 135.484 (±13.179	
						TS[4]_PN_Q_AB_nCi_2_MP0_T_KA_h + 1782.101 (±257.835)	
						KH[2]_PN_B_AB_nCi_2_SS8_T_KA_v-h	
4	0.915	0.887	0.879	−0.324	70.59	log K = −66.472 (±6.939) − 0.223 ± 0.021)	(21)
						AC[2]_MX_B_AB_nCi_2_SS7_T_KA_r-h + 0.407 (±0.089)	
						TS[5]_HM_B_AB_nCi_2_SS8_T_KA_v-h + 131.848 (±10.928)	
						TS[4]_PN_Q_AB_nCi_2_MP0_T_KA_h + 3323.451 (±355.509)	
						KH[2]_PN_B_AB_nCi_2_SS8_T_KA_v-h	
5	0.932	0.902	0.890	−0.376	68.53	log K = −70.522 (±6.342) − 0.246 (±0.020)	(22)
						AC[2]_MX_B_AB_nCi_2_SS7_T_KA_r-h + 0.422 (±0.081)	
						TS[5]_HM_B_AB_nCi_2_SS8_T_KA_v-h + 144.507 (±9.991)	
						TS[4]_PN_Q_AB_nCi_2_MP0_T_KA_h + 4616.536 (±15.439)	
						GV[2]_MX_Q_AB_nCi_2_MP3_X_KA_h + 3536.215 (±324.863)	
						KH[2]_PN_B_AB_nCi_2_SS8_T_KA_v-h	
6	0.942 (0.960)^a^	0.914 (0.937)^a^	0.898 (0.925)^a^	−0.414 (−0.465)^a^	65.26 (91.74)^a^	log K = −81.005 (±6.216) − 0.233 (±0.020)	(23)
						AC[2]_MX_B_AB_nCi_2_SS7_T_KA_r-h − 39,144.250 (±4.757)	
						AC[2]_MN_B_AB_nCi_2_MP2_A_KA_c-h + 0.572 (±17.485)	
						TS[5]_HM_B_AB_nCi_2_SS8_T_KA_v-h + 120.683 (±1.681)	
						TS[4]_PN_Q_AB_nCi_2_MP0_T_KA_h + 0.804 (±0.354)	
						TS[6]_HM_Q_AB_nCi_2_SS0_A_KA_h + 3979.089 (±310.376)	
						KH[2]_PN_B_AB_nCi_2_SS8_T_KA_v-h	

^a^Compound 31 excluded, taken as outlier, is not taken into account in the training set

**Table 8:** Comparison of Q_loo_^2^ statistics of *n*D-QSAR methods for the property log K (CGB)^†^ for 31 (or 30)

*n*D-QSAR method	PCs/var.	Statistical method	${\text{Q}}^{2}$_loo_	Equations/references
*31/30 Steroids (all dataset)*				
Combined electrostatic and shape similarity matrix	6	Genetic NN	0.941	[59]
QuBiLS-MAS^c^	6	MLR and GA	*0.937*	Equation 23
QuBiLS-MAS	6	MLR and GA	*0.914*	Equation 23
Hodking SM	6	Genetic NN	0.903	[59]
QuBiLS-MAS	5	MLR and GA	*0.902*	Equation 22
QuBiLS-MAS	4	MLR and GA	*0.887*	Equation 21
Fragment QS-SM	4	PLS	0.886	[60]
MEDV-13	5	MLR and GA	0.882	[61]
MiDSASA—“template”	2 “compounds”	–	0.88	[62]
SOM^a^	3	–	R^2^ 0.85	[63]
Tuned-QSAR	6	MLR and PCA	0.842	[64]
Autocorrelation vector 30	–	–	0.84	[65]
CoMMA	3	PLS	0.828	[66]
QuBiLS-MAS	3	MLR and GA	*0.826*	Equation 20
Similarity Indices (ESP MC matrix 30)	1	PLS	0.820	[65]
SOMFA/esp + ALPHA	–	SOR	0.82	[67]
Combined electrostatic and shape similarity matrix	6	MLR and GA	0.819	[59]
EEVA	4	PLS	0.81	[68]
SOM-4D-QSAR	4	SOM neural network	0.80	[69]
Charges and Properties from MEPS-AM1	5	MLR	0.80	[70]
HE State/E-State^a,b^	3	–	0.80	[71]
E-State^a,b^	3	–	0.79	[71]
CoSA	3 “Bins”	PLS	0.78	[72]
QSAR/E-State	3 “atoms”	–	0.78	[73]
TQSI	4	MLR	0.775	[64]
EVA	5	PLS	0.77	[74]
CoMSA	1	PLS	0.76	[75]
MQSM	5	MLR and PCA	0.759	[64]
EVA + ALPHA	–	SOR	0.75	[67]
GRIND	–	PLS	0.75	[76]
SEAL	3	PLS	0.748	[77]
SOMFA/esp	6	PLS	0.74	[67]
CoSCoSA^a^	3	–	0.74	[78]
CoSASA	3 “atoms”	PLS	0.73	[72]
E-State and kappa shape index	4	MLR	0.72	[79]
TARIS	2	–	0.71	[80]
MQSM	3	MLR	0.705	[64]
Combined electrostatic and shape similarity matrix	5	PLS	0.70	[59]
SAMFA-RF	–	RF	0.69	[81]
SAMFA-PLS	4–5	PLS	0.69	[81]
4D-QSAR	2	PLS	0.69	[69]
CoMMA (ab initio)	6	PLS	0.689	[82]
QSAR^a^	3	–	0.68	[83]
SOM-4D-QSAR	4	SOM Neural Network	0.68	[69]
Wagener’s (AMSP Method)	–	k-NN and FNN	0.630	[84]
SAMFA-SVM	–	SVM	0.60	[81]
ALPHA	2	PLS	0.57	[67]

Italic values indicate the results of QuBiLS-MAS approach

^a^When it is applicable, specifies the number of components (PCs)

^b^1.0 A models

^c^Compound 31 excluded, taken as outlier, is not taken into account in the training set

^†^Logarithm of the binding affinity to the corticosteroid-binding globulin (CBG)

In general, when the 31 steroids are taken into account as training set, the models based on QuBiLS-MAS indices yield comparable-to-superior performance relative to other methods reported in the literature according to the Q_loo_^2^ statistic. Up to now, the best model reported has been the one based on the “Combined Electrostatic and Shape Similarity Matrix” (Q_loo_^2^ = 0.941, *var* = 6), which is an alignment- and grid-based method known to be computationally expensive. Additionally, this model employs the Genetic Neural Network (GNN) as the fitting method, which generally yields more robust and better optimized models compared to other linear methods. Even then, comparable performance is obtained with QuBiLs-MAS models [(Q_loo_^2^ = 0.937 (compound 31 excluded), *var* = 6), (Q_loo_^2^ = 0.914 (compound 31 included), *var* = 6)] based on the MLR-GA, which is a much simpler technique than GNN. Therefore, based on the results obtained in this study, it can be claimed that the QuBiLs-MAS MDs proposed offer a considerable advantage over well-known traditional methodologies.

## Conclusions

The QuBiLs-MAS approach for atom-pair relations, in its diverse generalizations and extensions, seems to renew the prospect of achieving 2D-QSAR models with good predictive power. Inspired by the “No Free Lunch” theorem [58], which postulates that there is no unique best alternative for tackling optimization problems, the different extensions constitute an innovative undertaking to suitably characterize the different phenomena that affect the molecular configuration and intermolecular interactions, and thus affecting their biological activity. Variability and Principal Component analyses of the QuBiLs-MAS indices demonstrated that the proposed generalizations yield indices with superior variability compared to other indices defined in the literature and capture chemical information not codified by the DRAGON MD families. Also, it was demonstrated that suitable gains are obtained in the predictive ability of the QSAR models with the QuBiLs-MAS approach. Therefore, the QuBiLs-MAS 2D-indices constitute a relevant tool for the diversity analysis of compound datasets and high-throughput screening of structure–activity data.

## Futures outlooks

Future tasks include the development of a version of the QuBiLs-MAS module to compute molecular indices on a distributed computing system for high-throughput calculation, as well as, a version to use the Graphical Processing Units (GPU) present in several personal computers nowadays. Moreover, various (dis-)similarity multi-metrics to consider relations for more than two atoms (multi-linear forms) are to be introduced, in addition to a new set of multi-metrics based cut-offs.

## Additional file

**Additional file 1.** The mathematical definitions of the norms, means and statistical invariants as generalizations of the linear combination of LOVIs as global (and/or local) MDs aggregation operator, as well as classical algorithms which generalize the first three groups are presented as **Figure SI1**-**Table S12**. The UML diagram (**Figure SI3**), a debug report file content (**Figure SI4**), a batch process manager dialog window (**Figure SI5**) are also listed. Some results of the factor analysis by the principal component method are shown as **Table SI6**-**Table SI8**, and finally, the names of structures for Cramer’s steroid database and their corresponding values for the binding affinity to the corticosteroid-binding globulin (CBG) is in **Table SI9**.
