# Supplementary material for: QuBiLS-MAS, open source multi-platform software for atom- and bond-based topological (2D) and chiral (2.5D) algebraic molecular descriptors computations
Source: J Cheminform. 2017 Jun 7;9:35. doi: 10.1186/s13321-017-0211-5 (PMC5462671; doi:10.1186/s13321-017-0211-5)
Supplement: Supplementary file 1 — Additional file 1. The mathematical definitions of the norms, means and statistical invariants as generalizations of the linear combination of LOVIs as global (and/or local) MDs aggregation operator, as well as classical algorithms which generalize the first three groups are presented as Figure SI1-Table S12. The UML diagram (Figure SI3), a debug report file content (Figure SI4), a batch process manager dialog window (Figure SI5) are also listed. Some results of the factor analysis by the principal component method are shown as Table SI6-Table SI8, and finally, the names of structures for Cramer’s steroid database and their corresponding values for the binding affinity to the corticosteroid-binding globulin (CBG) is in Table SI9. [file 13321_2017_211_MOESM1_ESM.docx]

**Supplementary Information**

***QuBiLS-MAS*, Open Source Multi-Platform Software for Atom- and Bond-based 2D and 2.5D Algebraic Molecular Descriptors Computations**

**José R. Valdés-Martiní, Yovani Marrero-Ponce,(^
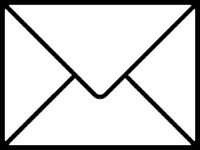
^) César R. García-Jacas, Karina Martinez-Mayorga, Stephen J. Barigye, Yasser S. Vaz d‘ Almeida, Hai Pham-The, Facundo Pérez-Giménez and Carlos A. Morell.**

## Table of Contents

[Table of Contents 1](#_Toc483911795)

[S1. Cartesian representation of the n-dimensional vector of LOVEIs; the blue line represents Minkowski’s first norm, also known as Manhattan norm or city-block distance to the origin of coordinates, and the red line Minkowski’s second norm, formally known as the Euclidean norm. 2](#_Toc483911796)

[S2. Norms, Means and Statistical Invariants as Generalizations of the Linear Combination of LOVIs as Global (and/or Local) MDs Aggregation Operator, as well as Classical Algorithms which generalize the first three groups. 3](#_Toc483911797)

[S3. UML diagram, showing the essential inheritance hierarchy within the QuBiLS-MAS MDL and the basic dependencies from CDK. 7](#_Toc483911798)

[S4. Debug Report file content for an *atom-based bilinear algebraic form* descriptor calculated to the *Isonicotinic Acid* structure. Considering electronegativity and mass as weighting schemes (properties) 8](#_Toc483911799)

[S5. Batch Process Manager dialog window, where the *Isonicotinic Acid* is loaded as input file, a project file is loaded and verified and output file format is set to ARFF 9](#_Toc483911800)

[S6. Table with the eigenvalues and percentages of the explained variance by the computed components regarding QuBiLS-MAS versus DRAGON 2D. 10](#_Toc483911801)

[S7. Factor Loadings using the Principal Components Method according the QuBiLS-MAS versus Dragon 2D 11](#_Toc483911802)

[S8. Details for the 0-2D Dragon MDs merely explained in a exclusive way into Factor 10. 97](#_Toc483911803)

[S9. Names of structures for Cramer’s steroid database and their corresponding values for the binding affinity to the corticosteroid-binding globulin (CBG). 98](#_Toc483911804)

## S1. Cartesian representation of the n-dimensional vector of LOVEIs; the blue line represents Minkowski’s first norm, also known as Manhattan norm or city-block distance to the origin of coordinates, and the red line Minkowski’s second norm, formally known as the Euclidean norm.


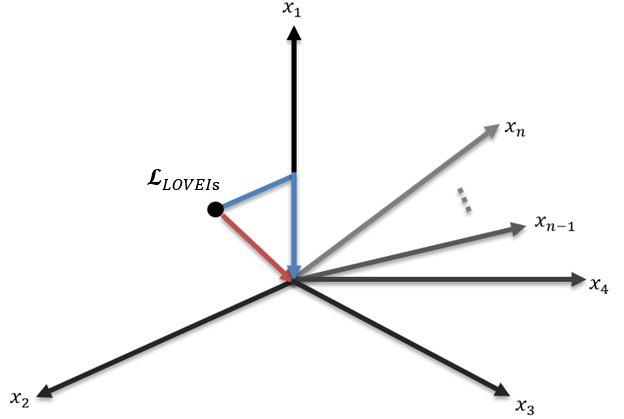


## S2. Norms, Means and Statistical Invariants as Generalizations of the Linear Combination of LOVIs as Global (and/or Local) MDs Aggregation Operator, as well as Classical Algorithms which generalize the first three groups.

| **No.** | **Group^a^** | **Name** | **Label** | **Formula^b^** |
| --- | --- | --- | --- | --- |
| 1 | **Norms**  (Metrics) | Minkowski norm (p = 1), also known as Manhattan norm | N1 |  |
| 2 |  | Minkowski norm (p = 2), also known as Euclidean norm | N2 |  |
| 3 |  | Minkowski norm (p = 3) | N3 |  |
| 4 |  | Penrose size | PN |  |
| 5 | **Means**  (firsts statistical moments) | Geometric Mean | GM |  |
| 6 |  | Arithmetic Mean  (Power mean of degree β = 1) | AM |  |
| 7 |  | Quadratic Mean  (Power mean of degree β = 2) | P2 |  |
| 8 |  | Power mean of degree β = 3 | P3 |  |
| 9 |  | Harmonic Mean  (Power mean of degree β = -1) | HM |  |
| 10 | **Statistical** (highest statistical moments): | Variance | V |  |
| 11 |  | Skewness | S |   M, arithmetic mean  SD, standard deviation |
| 12 |  | Kurtosis | K | M, arithmetic mean  SD, standard deviation |
| 13 |  | Standard Deviation | SD |  |
| 14 |  | Variation Coefficient | VC | $\text{VC}=\frac{SD}{M}$ |
| 15 |  | Range | RA |  |
| 16 |  | Percentile 25 | Q1 | $P 25= \left[ \frac{N}{4}+ \frac{1}{2} \right]$  N, L_a_ number |
| 17 |  | Percentile 50 | Q2 | $P 50= \left[ \frac{N}{2}+ \frac{1}{2} \right]$  N, L_a_ number |
| 18 |  | Percentile 75 | Q3 | $P 75= \left[ \frac{3N}{4}+ \frac{1}{2} \right]$  N, L_a_ number |
| 19 |  | Inter-quartile Range | I50 | $\text{I50}=Q3-Q1$ |
| 20 |  | Maximum value | MX | MX = *L_a_* max |
| 21 |  | Minimum value | MN | MN = *L_a_* min |
| 22 | **Classical Algorithms** | Autocorrelation | AC*^k^* | $\begin{matrix} AC_{k}=\sum_{i=1}^{n} \sum_{j\geq1}^{n} L_{i}\times L_{j}\bullet(\delta(d_{ij},k)) \\ k=1,2,..7 \end{matrix}$ |
| 23 |  | Gravitational | GV*^k^* | $\begin{matrix} GV_{k}=\frac{1}{n}\sum_{i=1}^{n} \sum_{j=1}^{n} \frac{L_{i}L_{j}}{{}^{k}d_{ij}}\bullet\delta(d_{ij},k)) \\ k=1,2,..7 \end{matrix}$ |
| 24 |  | Total sum at lag k | TS*^k^* | $\begin{matrix} TS_{k}=\sum_{i=1}^{n} \sum_{j=1}^{n} L_{ij}\bullet\delta(d_{ij},k))\text{ } \\ k=1,2,\ldots,7 \end{matrix}$ |
| 25 |  | Kier-Hall connectivity | KH*^m^* | ${}^{m}KH_{t}=\sum_{i=1}^{K} (\prod_{i=1}^{n_{k}} L_{i},w)_{k}^{\lambda}$  *where, K is the number of sub-graphs, n_k_ is the number of atoms in a fragment, λ is equal to ½, m and t are the sub-graph order and type, respectively* |
| 26 |  | Mean Information Content | MIC | $MIC=-\sum_{i=1}^{A} \frac{N_{g}}{N_{o}}\cdot\log_{2}\frac{N_{g}}{N_{o}}$  *where, Ng is the number of atoms with the same LOVI value. N_o_ is the number of atoms in a molecule* |
| 27 |  | Total Information Content | TIC |  |
| 28 |  | Standardized Information Content | SIC | $SIC=\frac{IT}{N_{0}\cdot\log_{2}N_{0}}$ |
| 29 |  | Electrotopological state (E-state index) | ES | $S_{i}=I_{i}+\Delta I_{i}=I_{i}+\sum_{j=1}^{n} \frac{I_{i}-I_{j}}{(d_{ij}+1)^{2}}$*where, I_i_ is the intrinsic state of the i^th^ atom and ΔI_i_ is the field effect on the ith atom calculated as perturbation of the I_i_ of i^th^ atom by all other atoms in the molecule, d_ij_ is the topological distance between the i^th^ and the j^th^ atoms, and n is the number of atoms. The exponent k is 2.* |
| 30 |  | Ivanciuc-Balaban Type-Indices | IB | $J_{k}=\frac{n^{2}\cdot B}{n+C+1}\sum_{i=1}^{n-1} \sum_{j=i+1}^{n} a_{ij}\left[L_{i}\times L_{j}\right]^{-\frac{1}{2}}$  *where, the summation goes over all pairs of atoms but only pairs of adjacent atoms are accounted for by means of the elements a_ij_ of the adjacency matrix. The n, B, and C are the number of atoms, bonds, and rings (cyclomatic number), respectively.* |

## S3. UML diagram, showing the essential inheritance hierarchy within the QuBiLS-MAS MDL and the basic dependencies from CDK.


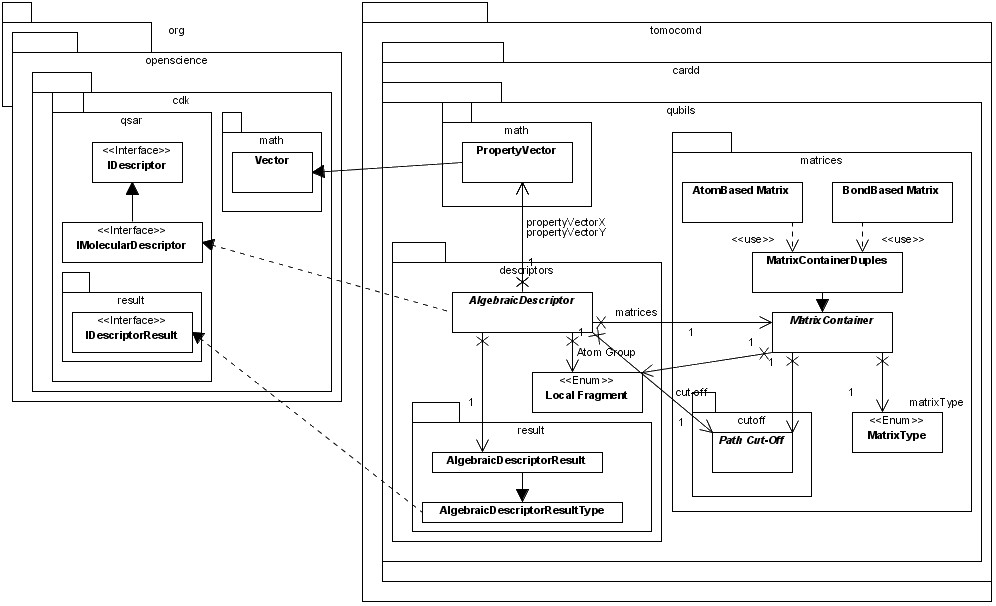


## S4. Debug Report file content for an *atom-based bilinear algebraic form* descriptor calculated to the *Isonicotinic Acid* structure. Considering electronegativity and mass as weighting schemes (properties)

| Property Vector X  [ O1; C2; O3; C4; C5; C6; N7; C8; C9]  [ 1.33; 1.0; 1.33; 1.0; 1.0; 1.0; 1.16; 1.0; 1.0 ]  Property Vector Y  [ O1; C2; O3; C4; C5; C6; N7; C8; C9]  [ 3.44; 2.55; 3.44; 2.55; 2.55; 2.55; 3.04; 2.55; 2.55 ]  Matrix order 0  [ O1; C2; O3; C4; C5; C6; N7; C8; C9 ]  O1[ 1.0; 0.0; 0.0; 0.0; 0.0; 0.0; 0.0; 0.0; 0.0 ]  C2[ 0.0; 1.0; 0.0; 0.0; 0.0; 0.0; 0.0; 0.0; 0.0 ]  O3[ 0.0; 0.0; 1.0; 0.0; 0.0; 0.0; 0.0; 0.0; 0.0 ]  C4[ 0.0; 0.0; 0.0; 1.0; 0.0; 0.0; 0.0; 0.0; 0.0 ]  C5[ 0.0; 0.0; 0.0; 0.0; 1.0; 0.0; 0.0; 0.0; 0.0 ]  C6[ 0.0; 0.0; 0.0; 0.0; 0.0; 1.0; 0.0; 0.0; 0.0 ]  N7[ 0.0; 0.0; 0.0; 0.0; 0.0; 0.0; 1.0; 0.0; 0.0 ]  C8[ 0.0; 0.0; 0.0; 0.0; 0.0; 0.0; 0.0; 1.0; 0.0 ]  C9[ 0.0; 0.0; 0.0; 0.0; 0.0; 0.0; 0.0; 0.0; 1.0 ]  LOVIS Vector order 0  [ O1; C2; O3; C4; C5; C6; N7; C8; C9]  [ 4.582396401336023; 2.55; 4.582396401336023; 2.55; 2.55; 2.55; 3.5451930768685536; 2.55; 2.55 ]  Matrix order 1  [ O1; C2; O3; C4; C5; C6; N7; C8; C9 ]  O1[ 0.0; 1.0; 0.0; 0.0; 0.0; 0.0; 0.0; 0.0; 0.0 ]  C2[ 1.0; 0.0; 2.0; 1.0; 0.0; 0.0; 0.0; 0.0; 0.0 ]  O3[ 0.0; 2.0; 0.0; 0.0; 0.0; 0.0; 0.0; 0.0; 0.0 ]  C4[ 0.0; 1.0; 0.0; 1.0; 1.0; 0.0; 0.0; 0.0; 1.0 ]  C5[ 0.0; 0.0; 0.0; 1.0; 1.0; 1.0; 0.0; 0.0; 0.0 ]  C6[ 0.0; 0.0; 0.0; 0.0; 1.0; 1.0; 1.0; 0.0; 0.0 ]  N7[ 0.0; 0.0; 0.0; 0.0; 0.0; 1.0; 1.0; 1.0; 0.0 ]  C8[ 0.0; 0.0; 0.0; 0.0; 0.0; 0.0; 1.0; 1.0; 1.0 ]  C9[ 0.0; 0.0; 0.0; 1.0; 0.0; 0.0; 0.0; 1.0; 1.0 ]  LOVIS Vector order 1  [ O1; C2; O3; C4; C5; C6; N7; C8; C9]  [ 3.39; 12.87; 6.791; 10.2; 7.64; 8.14; 9.49; 8.14; 7.64 ]  Matrix order 2  [ O1; C2; O3; C4; C5; C6; N7; C8; C9 ]  O1[ 1.0; 0.0; 2.0; 1.0; 0.0; 0.0; 0.0; 0.0; 0.0 ]  C2[ 0.0; 6.0; 0.0; 1.0; 1.0; 0.0; 0.0; 0.0; 1.0 ]  O3[ 2.0; 0.0; 4.0; 2.0; 0.0; 0.0; 0.0; 0.0; 0.0 ]  C4[ 1.0; 1.0; 2.0; 4.0; 2.0; 1.0; 0.0; 1.0; 2.0 ]  C5[ 0.0; 1.0; 0.0; 2.0; 3.0; 2.0; 1.0; 0.0; 1.0 ]  C6[ 0.0; 0.0; 0.0; 1.0; 2.0; 3.0; 2.0; 1.0; 0.0 ]  N7[ 0.0; 0.0; 0.0; 0.0; 1.0; 2.0; 3.0; 2.0; 1.0 ]  C8[ 0.0; 0.0; 0.0; 1.0; 0.0; 1.0; 2.0; 3.0; 2.0 ]  C9[ 0.0; 1.0; 0.0; 2.0; 1.0; 0.0; 1.0; 2.0; 3.0 ]  LOVIS Vector order 2  [ O1; C2; O3; C4; C5; C6; N7; C8; C9]  [ 17.14; 22.95; 34.28; 38.37; 25.99; 23.93; 28.47; 23.93; 25.98 ] |
| --- |

## S5. Batch Process Manager dialog window, where the *Isonicotinic Acid* is loaded as input file, a project file is loaded and verified and output file format is set to ARFF


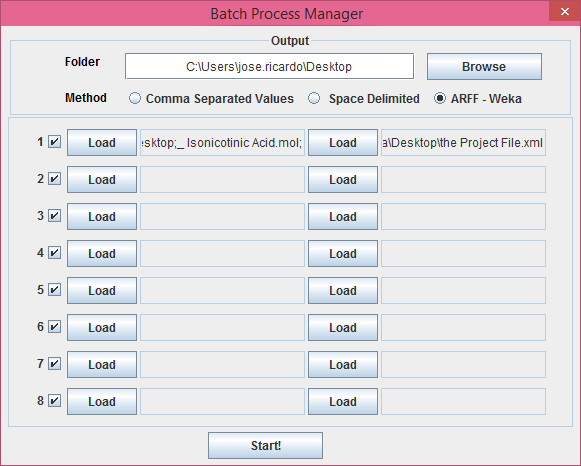


## S6. Table with the eigenvalues and percentages of the explained variance by the computed components regarding QuBiLS-MAS versus DRAGON 2D.

| Factor | Eigenvalue | % Total Variance | Cumulative Eigenvalue | Cumulative % |
| --- | --- | --- | --- | --- |
| 1 | 445.4048 | 27.83780 | 445.405 | 27.83780 |
| 2 | 209.5491 | 13.09682 | 654.954 | 40.93462 |
| 3 | 137.6652 | 8.60407 | 792.619 | 49.53870 |
| 4 | 100.0436 | 6.25272 | 892.663 | 55.79142 |
| 5 | 61.8280 | 3.86425 | 954.491 | 59.65567 |
| 6 | 56.1862 | 3.51163 | 1010.677 | 63.16731 |
| 7 | 43.4614 | 2.71634 | 1054.138 | 65.88364 |
| 8 | 39.5535 | 2.47209 | 1093.692 | 68.35574 |
| 9 | 31.8823 | 1.99264 | 1125.574 | 70.34838 |
| 10 | 26.0086 | 1.62554 | 1151.583 | 71.97392 |
| 11 | 22.7846 | 1.42404 | 1174.367 | 73.39796 |
| 12 | 19.2692 | 1.20433 | 1193.637 | 74.60228 |

## S7. Factor Loadings using the Principal Components Method according the QuBiLS-MAS versus Dragon 2D

| **MDs** | **Factor 1** | **Factor 2** | **Factor3** | **Factor 4** | **Factor 5** | **Factor 6** | **Factor 7** | **Factor 8** | **Factor 9** | **Factor 10** | **Factor 11** | **Factor 12** |
| --- | --- | --- | --- | --- | --- | --- | --- | --- | --- | --- | --- | --- |
| PN_B_AB_nCi_2_SS0_T_KA_a-v_MAS | 0.1282 | 0.4412 | 0.47413 | 0.0922 | 0.15079 | 0.18853 | 0.23433 | 0.02822 | 0.60968 | 0.01008 | 0.05341 | 0.07810 |
| PN_B_AB_nCi_2_SS1_T_KA_a-v_MAS | 0.1186 | 0.4686 | 0.40450 | 0.0742 | 0.14489 | 0.10199 | 0.24803 | 0.04626 | 0.65486 | 0.02853 | 0.02378 | 0.09478 |
| PN_B_AB_nCi_2_SS2_T_KA_a-v_MAS | 0.1229 | 0.4621 | 0.47406 | 0.0869 | 0.14963 | 0.19370 | 0.23698 | 0.02699 | 0.59282 | 0.01267 | 0.07555 | 0.08527 |
| PN_B_AB_nCi_2_SS3_T_KA_a-v_MAS | 0.1167 | 0.4717 | 0.43458 | 0.0837 | 0.16452 | 0.14554 | 0.24634 | 0.03603 | 0.62327 | 0.01430 | 0.04793 | 0.09379 |
| PN_B_AB_nCi_2_SS4_T_KA_a-v_MAS | 0.1195 | 0.4730 | 0.46680 | 0.0851 | 0.15115 | 0.19039 | 0.23536 | 0.02840 | 0.59316 | 0.01583 | 0.08156 | 0.08455 |
| PN_B_AB_nCi_2_SS5_T_KA_a-v_MAS | 0.1167 | 0.4738 | 0.44323 | 0.0874 | 0.17252 | 0.16567 | 0.24371 | 0.03236 | 0.60762 | 0.01067 | 0.05875 | 0.09184 |
| PN_B_AB_nCi_2_SS6_T_KA_a-v_MAS | 0.1181 | 0.4783 | 0.46364 | 0.0849 | 0.15474 | 0.19094 | 0.23410 | 0.02889 | 0.59090 | 0.01757 | 0.08465 | 0.08430 |
| P2_B_AB_nCi_2_SS0_T_KA_a-v_MAS | 0.0765 | 0.3083 | 0.70011 | 0.1516 | -0.09355 | 0.16281 | 0.00204 | 0.00045 | 0.51431 | 0.01754 | 0.03096 | 0.06403 |
| P2_B_AB_nCi_2_SS1_T_KA_a-v_MAS | 0.1085 | 0.4161 | 0.52959 | 0.0847 | -0.00765 | 0.04556 | 0.11000 | 0.03116 | 0.64365 | 0.03372 | 0.00464 | 0.07464 |
| P2_B_AB_nCi_2_SS2_T_KA_a-v_MAS | 0.1037 | 0.3896 | 0.62532 | 0.1237 | -0.02641 | 0.21540 | 0.07218 | 0.00130 | 0.55630 | -0.01142 | 0.06463 | 0.05572 |
| P2_B_AB_nCi_2_SS3_T_KA_a-v_MAS | 0.1091 | 0.4424 | 0.51688 | 0.1013 | 0.04769 | 0.12426 | 0.12895 | 0.02527 | 0.64522 | -0.00201 | 0.03243 | 0.08548 |
| P2_B_AB_nCi_2_SS4_T_KA_a-v_MAS | 0.1025 | 0.4321 | 0.57401 | 0.1021 | -0.01106 | 0.19218 | 0.08950 | 0.01428 | 0.59678 | 0.00128 | 0.06748 | 0.05860 |
| P2_B_AB_nCi_2_SS5_T_KA_a-v_MAS | 0.1098 | 0.4504 | 0.51576 | 0.1035 | 0.07230 | 0.15315 | 0.13283 | 0.02568 | 0.63102 | 0.00246 | 0.04447 | 0.08393 |
| P2_B_AB_nCi_2_SS6_T_KA_a-v_MAS | 0.0992 | 0.4446 | 0.55617 | 0.0931 | 0.00678 | 0.17934 | 0.10090 | 0.02243 | 0.60629 | 0.01367 | 0.07177 | 0.05906 |
| P3_B_AB_nCi_2_SS0_T_KA_a-v_MAS | -0.1268 | -0.1911 | -0.63343 | -0.2837 | -0.08059 | -0.24947 | -0.20330 | 0.01504 | -0.35842 | 0.04033 | 0.01756 | 0.06620 |
| P3_B_AB_nCi_2_SS1_T_KA_a-v_MAS | -0.1331 | -0.3556 | -0.54822 | -0.1328 | -0.03415 | -0.07733 | -0.16961 | -0.02211 | -0.56338 | -0.00523 | 0.03099 | -0.00898 |
| P3_B_AB_nCi_2_SS2_T_KA_a-v_MAS | -0.1335 | -0.3062 | -0.61745 | -0.2239 | -0.04827 | -0.28174 | -0.16601 | 0.01430 | -0.43629 | 0.06445 | -0.03909 | 0.02319 |
| P3_B_AB_nCi_2_SS3_T_KA_a-v_MAS | -0.1252 | -0.3792 | -0.51463 | -0.1648 | -0.08857 | -0.16249 | -0.20299 | -0.01735 | -0.58187 | 0.06170 | -0.00078 | -0.01561 |
| P3_B_AB_nCi_2_SS4_T_KA_a-v_MAS | -0.1259 | -0.3692 | -0.57337 | -0.1858 | -0.03365 | -0.23983 | -0.16484 | -0.00385 | -0.51296 | 0.04995 | -0.04330 | 0.00922 |
| P3_B_AB_nCi_2_SS5_T_KA_a-v_MAS | -0.1240 | -0.3856 | -0.50979 | -0.1725 | -0.11887 | -0.18649 | -0.21141 | -0.02077 | -0.57156 | 0.05455 | -0.01703 | -0.01828 |
| P3_B_AB_nCi_2_SS6_T_KA_a-v_MAS | -0.1194 | -0.3843 | -0.55717 | -0.1696 | -0.04291 | -0.21390 | -0.17448 | -0.01452 | -0.53714 | 0.03511 | -0.04630 | 0.00905 |
| S_B_AB_nCi_2_SS0_T_KA_a-v_MAS | -0.0402 | 0.2604 | -0.12853 | -0.4215 | -0.26934 | -0.20155 | -0.23375 | -0.02341 | 0.41109 | -0.03178 | 0.00709 | 0.25946 |
| S_B_AB_nCi_2_SS1_T_KA_a-v_MAS | -0.1053 | -0.0017 | -0.35270 | -0.2078 | -0.14039 | -0.15498 | -0.15649 | -0.04826 | 0.16715 | -0.05964 | -0.01305 | 0.17391 |
| S_B_AB_nCi_2_SS2_T_KA_a-v_MAS | -0.0273 | 0.2434 | -0.10587 | -0.4021 | -0.18516 | -0.30532 | -0.16228 | -0.01067 | 0.46884 | 0.06410 | -0.02634 | 0.19677 |
| S_B_AB_nCi_2_SS3_T_KA_a-v_MAS | 0.0026 | 0.1430 | -0.18198 | -0.2804 | -0.08993 | -0.29628 | -0.17063 | -0.05747 | 0.31662 | 0.13822 | -0.04536 | 0.20313 |
| S_B_AB_nCi_2_SS4_T_KA_a-v_MAS | -0.0178 | 0.2276 | -0.10112 | -0.3890 | -0.10788 | -0.28849 | -0.15709 | -0.02475 | 0.43246 | 0.09788 | -0.02999 | 0.18907 |
| S_B_AB_nCi_2_SS5_T_KA_a-v_MAS | 0.0256 | 0.2017 | -0.09010 | -0.3144 | -0.07157 | -0.30875 | -0.18239 | -0.06072 | 0.36398 | 0.14127 | -0.05688 | 0.18008 |
| S_B_AB_nCi_2_SS6_T_KA_a-v_MAS | -0.0033 | 0.2557 | -0.08714 | -0.3763 | -0.05553 | -0.22945 | -0.16947 | -0.03943 | 0.40568 | 0.10092 | -0.03171 | 0.19033 |
| SD_B_AB_nCi_2_SS0_T_KA_a-v_MAS | -0.0255 | -0.0348 | 0.73447 | 0.2003 | -0.31821 | 0.10473 | -0.26208 | -0.04136 | 0.15361 | -0.00999 | -0.02845 | -0.01175 |
| SD_B_AB_nCi_2_SS1_T_KA_a-v_MAS | 0.0592 | 0.1283 | 0.59918 | 0.1070 | -0.25896 | -0.05600 | -0.15050 | -0.01436 | 0.37663 | 0.00449 | -0.06006 | -0.03615 |
| SD_B_AB_nCi_2_SS2_T_KA_a-v_MAS | 0.0210 | -0.0163 | 0.67386 | 0.1930 | -0.32750 | 0.21293 | -0.26530 | -0.06032 | 0.17291 | -0.11079 | 0.00125 | -0.05681 |
| SD_B_AB_nCi_2_SS3_T_KA_a-v_MAS | 0.0417 | 0.0748 | 0.55695 | 0.1678 | -0.27838 | 0.04906 | -0.23245 | -0.01964 | 0.39485 | -0.12512 | -0.06040 | -0.03144 |
| SD_B_AB_nCi_2_SS4_T_KA_a-v_MAS | 0.0206 | 0.0303 | 0.60104 | 0.1580 | -0.37472 | 0.15356 | -0.30020 | -0.03450 | 0.26446 | -0.10177 | -0.01872 | -0.06541 |
| SD_B_AB_nCi_2_SS5_T_KA_a-v_MAS | 0.0371 | 0.0339 | 0.55098 | 0.1865 | -0.26721 | 0.08688 | -0.28354 | -0.00992 | 0.36626 | -0.11976 | -0.07006 | -0.04500 |
| SD_B_AB_nCi_2_SS6_T_KA_a-v_MAS | 0.0099 | 0.0235 | 0.57198 | 0.1354 | -0.37776 | 0.09794 | -0.31346 | -0.00979 | 0.28835 | -0.07036 | -0.02746 | -0.07865 |
| PN_B_AB_nCi_2_SS0_T_KA_h-a_MAS | -0.2489 | 0.2576 | 0.56142 | 0.0829 | 0.12402 | 0.23294 | 0.23313 | 0.01231 | 0.42145 | -0.13633 | -0.10409 | 0.12681 |
| PN_B_AB_nCi_2_SS1_T_KA_h-a_MAS | -0.2234 | 0.2891 | 0.44702 | 0.0685 | 0.13251 | 0.26240 | 0.23236 | 0.01292 | 0.50591 | -0.16766 | -0.11398 | 0.14660 |
| PN_B_AB_nCi_2_SS2_T_KA_h-a_MAS | -0.2441 | 0.2772 | 0.50471 | 0.0678 | 0.12960 | 0.22908 | 0.23961 | 0.02334 | 0.48369 | -0.14726 | -0.13800 | 0.14246 |
| PN_B_AB_nCi_2_SS3_T_KA_h-a_MAS | -0.2244 | 0.2944 | 0.44308 | 0.0620 | 0.13120 | 0.23723 | 0.24615 | 0.02340 | 0.52607 | -0.14390 | -0.14565 | 0.15467 |
| PN_B_AB_nCi_2_SS4_T_KA_h-a_MAS | -0.2379 | 0.2902 | 0.47162 | 0.0614 | 0.13636 | 0.21238 | 0.24412 | 0.03143 | 0.51291 | -0.14084 | -0.16029 | 0.15541 |
| PN_B_AB_nCi_2_SS5_T_KA_h-a_MAS | -0.2227 | 0.2944 | 0.44039 | 0.0572 | 0.13828 | 0.22390 | 0.25019 | 0.02753 | 0.53184 | -0.13105 | -0.15982 | 0.15788 |
| PN_B_AB_nCi_2_SS6_T_KA_h-a_MAS | -0.2369 | 0.2902 | 0.45813 | 0.0569 | 0.14227 | 0.20341 | 0.24642 | 0.03580 | 0.52252 | -0.13355 | -0.17081 | 0.15909 |
| P2_B_AB_nCi_2_SS0_T_KA_h-a_MAS | -0.3276 | 0.1020 | 0.68245 | 0.1633 | -0.03788 | 0.23104 | 0.05835 | -0.03803 | 0.21028 | -0.13539 | -0.19472 | 0.07741 |
| P2_B_AB_nCi_2_SS1_T_KA_h-a_MAS | -0.2742 | 0.2104 | 0.53720 | 0.1072 | 0.01828 | 0.27345 | 0.13781 | -0.01459 | 0.43265 | -0.20668 | -0.15837 | 0.11699 |
| P2_B_AB_nCi_2_SS2_T_KA_h-a_MAS | -0.3050 | 0.1837 | 0.61146 | 0.1160 | -0.01375 | 0.26526 | 0.09754 | -0.01955 | 0.35773 | -0.19146 | -0.20306 | 0.09129 |
| P2_B_AB_nCi_2_SS3_T_KA_h-a_MAS | -0.2737 | 0.2522 | 0.49469 | 0.0883 | 0.02570 | 0.24388 | 0.15255 | 0.00652 | 0.49810 | -0.19173 | -0.18491 | 0.15239 |
| P2_B_AB_nCi_2_SS4_T_KA_h-a_MAS | -0.2982 | 0.2371 | 0.54183 | 0.0844 | -0.00322 | 0.22488 | 0.11864 | 0.00765 | 0.45818 | -0.18326 | -0.22682 | 0.12467 |
| P2_B_AB_nCi_2_SS5_T_KA_h-a_MAS | -0.2732 | 0.2572 | 0.48376 | 0.0738 | 0.04503 | 0.22671 | 0.16081 | 0.01811 | 0.51080 | -0.16792 | -0.20106 | 0.16260 |
| P2_B_AB_nCi_2_SS6_T_KA_h-a_MAS | -0.2992 | 0.2441 | 0.51420 | 0.0678 | 0.01768 | 0.19977 | 0.13385 | 0.02474 | 0.48779 | -0.16632 | -0.23412 | 0.13874 |
| P3_B_AB_nCi_2_SS0_T_KA_h-a_MAS | 0.2106 | -0.0776 | -0.64973 | -0.3169 | -0.11029 | -0.21555 | -0.19569 | 0.03152 | -0.11241 | 0.13491 | 0.12131 | -0.01396 |
| P3_B_AB_nCi_2_SS1_T_KA_h-a_MAS | 0.2530 | -0.1783 | -0.55645 | -0.1597 | -0.02385 | -0.28672 | -0.16409 | 0.01981 | -0.36451 | 0.21202 | 0.16276 | -0.06488 |
| P3_B_AB_nCi_2_SS2_T_KA_h-a_MAS | 0.2278 | -0.1561 | -0.61211 | -0.2318 | -0.10187 | -0.25529 | -0.17731 | 0.01223 | -0.23923 | 0.19357 | 0.12188 | -0.06171 |
| P3_B_AB_nCi_2_SS3_T_KA_h-a_MAS | 0.2496 | -0.2209 | -0.49867 | -0.1473 | -0.03223 | -0.26621 | -0.19845 | 0.00176 | -0.44791 | 0.22260 | 0.19523 | -0.08824 |
| P3_B_AB_nCi_2_SS4_T_KA_h-a_MAS | 0.2410 | -0.2185 | -0.55364 | -0.1860 | -0.08005 | -0.21927 | -0.18609 | -0.01305 | -0.37018 | 0.19658 | 0.16837 | -0.09159 |
| P3_B_AB_nCi_2_SS5_T_KA_h-a_MAS | 0.2516 | -0.2247 | -0.48705 | -0.1310 | -0.05708 | -0.24869 | -0.21194 | -0.01196 | -0.46382 | 0.19863 | 0.21191 | -0.09958 |
| S_B_AB_nCi_2_SS0_T_KA_h-a_MAS | -0.1479 | 0.0977 | -0.20566 | -0.6194 | -0.24423 | -0.14547 | -0.23982 | -0.04575 | 0.24937 | -0.06310 | -0.05915 | 0.17197 |
| S_B_AB_nCi_2_SS1_T_KA_h-a_MAS | -0.1095 | -0.0664 | -0.30425 | -0.3281 | -0.13897 | -0.23387 | -0.13585 | -0.06219 | 0.21781 | -0.03066 | -0.03269 | 0.16536 |
| S_B_AB_nCi_2_SS2_T_KA_h-a_MAS | -0.1343 | 0.0671 | -0.19605 | -0.5106 | -0.26695 | -0.21666 | -0.18984 | -0.03231 | 0.32852 | -0.01960 | -0.06882 | 0.16308 |
| S_B_AB_nCi_2_SS3_T_KA_h-a_MAS | -0.0176 | 0.0186 | -0.14897 | -0.3356 | -0.12446 | -0.30141 | -0.20898 | -0.07147 | 0.26010 | 0.09259 | -0.03739 | 0.17867 |
| S_B_AB_nCi_2_SS4_T_KA_h-a_MAS | -0.0971 | 0.0321 | -0.17599 | -0.4723 | -0.25916 | -0.21319 | -0.21691 | -0.04553 | 0.30566 | -0.00175 | -0.04381 | 0.17034 |
| S_B_AB_nCi_2_SS5_T_KA_h-a_MAS | 0.0136 | 0.0725 | -0.09242 | -0.3478 | -0.16932 | -0.31741 | -0.25477 | -0.06628 | 0.32648 | 0.07214 | -0.03575 | 0.17584 |
| S_B_AB_nCi_2_SS6_T_KA_h-a_MAS | -0.0689 | 0.0431 | -0.15134 | -0.4521 | -0.24721 | -0.18707 | -0.24436 | -0.05398 | 0.29784 | 0.00428 | -0.02362 | 0.17670 |
| SD_B_AB_nCi_2_SS0_T_KA_h-a_MAS | -0.3392 | -0.0471 | 0.67658 | 0.2153 | -0.13175 | 0.18883 | -0.06877 | -0.06642 | -0.00820 | -0.11611 | -0.23086 | 0.01843 |
| SD_B_AB_nCi_2_SS1_T_KA_h-a_MAS | -0.2897 | 0.0068 | 0.57060 | 0.1684 | -0.16165 | 0.24495 | -0.03500 | -0.06988 | 0.17543 | -0.23780 | -0.20320 | -0.00370 |
| SD_B_AB_nCi_2_SS2_T_KA_h-a_MAS | -0.3113 | -0.0230 | 0.62584 | 0.1863 | -0.17951 | 0.25138 | -0.10928 | -0.07260 | 0.04060 | -0.22358 | -0.23088 | -0.01346 |
| SD_B_AB_nCi_2_SS3_T_KA_h-a_MAS | -0.3168 | 0.0259 | 0.48625 | 0.1671 | -0.24344 | 0.21731 | -0.10896 | -0.04499 | 0.22692 | -0.30381 | -0.24436 | 0.05969 |
| SD_B_AB_nCi_2_SS5_T_KA_h-a_MAS | -0.3356 | -0.0038 | 0.46994 | 0.1499 | -0.24840 | 0.19673 | -0.14550 | -0.02086 | 0.20983 | -0.28826 | -0.28108 | 0.08161 |
| HM_B_AB_nCi_2_SS1_T_KA_h-v_MAS | -0.5592 | 0.1308 | 0.11054 | -0.0011 | 0.14176 | 0.14552 | 0.06526 | -0.05368 | -0.02978 | -0.22384 | -0.35182 | 0.32328 |
| HM_B_AB_nCi_2_SS3_T_KA_h-v_MAS | -0.5922 | 0.1217 | 0.11455 | -0.0218 | 0.11711 | 0.09670 | 0.06435 | -0.03519 | 0.01336 | -0.18947 | -0.36315 | 0.31822 |
| HM_B_AB_nCi_2_SS5_T_KA_h-v_MAS | -0.5934 | 0.1183 | 0.11295 | -0.0261 | 0.11011 | 0.06135 | 0.07293 | -0.02971 | 0.02809 | -0.17877 | -0.37556 | 0.32179 |
| S_B_AB_nCi_2_SS0_T_KA_h-v_MAS | 0.0561 | 0.0256 | 0.38429 | 0.4846 | 0.04989 | -0.03540 | -0.01676 | 0.01259 | -0.05450 | 0.04059 | -0.05889 | -0.08180 |
| S_B_AB_nCi_2_SS1_T_KA_h-v_MAS | 0.1297 | 0.1566 | 0.42954 | 0.1048 | -0.08746 | -0.03728 | -0.06046 | 0.04683 | 0.25536 | 0.03232 | -0.25852 | -0.09259 |
| S_B_AB_nCi_2_SS2_T_KA_h-v_MAS | 0.2551 | 0.1581 | 0.27507 | 0.2158 | -0.15780 | -0.09395 | -0.00558 | 0.02321 | 0.27524 | -0.04784 | -0.13726 | -0.00164 |
| S_B_AB_nCi_2_SS3_T_KA_h-v_MAS | 0.1567 | 0.0589 | 0.39750 | 0.0905 | -0.12159 | -0.10684 | 0.01227 | 0.07040 | 0.40512 | 0.03135 | -0.31958 | -0.15287 |
| S_B_AB_nCi_2_SS4_T_KA_h-v_MAS | 0.2310 | 0.0501 | 0.33443 | 0.0886 | -0.19431 | -0.12285 | -0.00999 | 0.06517 | 0.41314 | 0.04611 | -0.15780 | -0.08951 |
| S_B_AB_nCi_2_SS5_T_KA_h-v_MAS | 0.1246 | 0.0184 | 0.36571 | 0.0429 | -0.15898 | -0.14527 | 0.03228 | 0.08894 | 0.51271 | 0.06649 | -0.28640 | -0.18748 |
| S_B_AB_nCi_2_SS6_T_KA_h-v_MAS | 0.1863 | 0.0231 | 0.31693 | 0.0326 | -0.19904 | -0.14229 | -0.00796 | 0.04927 | 0.50169 | 0.09395 | -0.12166 | -0.10014 |
| SD_B_AB_nCi_2_SS0_T_KA_h-v_MAS | -0.2617 | -0.0133 | 0.58654 | 0.2092 | -0.07341 | 0.09245 | 0.06436 | -0.07417 | 0.04224 | -0.10910 | -0.11861 | 0.05426 |
| SD_B_AB_nCi_2_SS1_T_KA_h-v_MAS | -0.4289 | -0.0247 | 0.35739 | -0.0671 | -0.05657 | -0.19108 | 0.08589 | 0.04481 | 0.21877 | -0.04928 | -0.34962 | -0.15989 |
| SD_B_AB_nCi_2_SS3_T_KA_h-v_MAS | -0.3675 | -0.0510 | 0.32084 | -0.0394 | 0.01115 | -0.18749 | 0.13243 | 0.04867 | 0.18324 | -0.12130 | -0.39130 | -0.24239 |
| VC_B_AB_nCi_2_SS0_T_KA_h-v_MAS | 0.2014 | -0.0890 | 0.56575 | 0.2954 | -0.18463 | 0.06057 | 0.01332 | -0.03825 | 0.05953 | 0.04770 | 0.14671 | -0.11411 |
| VC_B_AB_nCi_2_SS1_T_KA_h-v_MAS | 0.0454 | -0.1511 | 0.30598 | -0.0785 | -0.19092 | -0.34524 | 0.02632 | 0.13927 | 0.27808 | 0.16788 | -0.08367 | -0.45271 |
| VC_B_AB_nCi_2_SS2_T_KA_h-v_MAS | 0.3992 | -0.1473 | 0.31492 | 0.1499 | 0.02318 | -0.01774 | 0.02033 | 0.04665 | 0.06871 | 0.02965 | 0.15074 | -0.34374 |
| VC_B_AB_nCi_2_SS3_T_KA_h-v_MAS | 0.1050 | -0.1808 | 0.27455 | -0.0317 | -0.09781 | -0.30565 | 0.09466 | 0.13584 | 0.20879 | 0.04811 | -0.14595 | -0.54979 |
| VC_B_AB_nCi_2_SS4_T_KA_h-v_MAS | 0.3169 | -0.1957 | 0.20414 | 0.0566 | 0.15326 | -0.00658 | -0.03253 | 0.10542 | 0.05806 | 0.08573 | 0.08274 | -0.46245 |
| VC_B_AB_nCi_2_SS5_T_KA_h-v_MAS | 0.0555 | -0.1973 | 0.27897 | -0.0362 | -0.04304 | -0.18594 | 0.06122 | 0.13951 | 0.19614 | 0.01800 | -0.12723 | -0.59589 |
| VC_B_AB_nCi_2_SS6_T_KA_h-v_MAS | 0.2189 | -0.1811 | 0.19491 | 0.0221 | 0.16028 | 0.07826 | -0.07600 | 0.08653 | 0.08111 | 0.04882 | 0.06917 | -0.49978 |
| S_B_AB_nCi_2_MP0_T_KA_a-v_MAS | -0.0402 | 0.2604 | -0.12853 | -0.4215 | -0.26934 | -0.20155 | -0.23375 | -0.02341 | 0.41109 | -0.03178 | 0.00709 | 0.25946 |
| S_B_AB_nCi_2_MP0_T_KA_h-a_MAS | -0.1479 | 0.0977 | -0.20566 | -0.6194 | -0.24423 | -0.14547 | -0.23982 | -0.04575 | 0.24937 | -0.06310 | -0.05915 | 0.17197 |
| S_B_AB_nCi_2_MP0_T_KA_h-v_MAS | 0.0561 | 0.0256 | 0.38429 | 0.4846 | 0.04989 | -0.03540 | -0.01676 | 0.01259 | -0.05450 | 0.04059 | -0.05889 | -0.08180 |
| S_B_AB_nCi_2_MP1_T_KA_h-v_MAS | 0.0423 | -0.0766 | 0.23835 | 0.0327 | -0.25326 | -0.32637 | 0.08436 | 0.05225 | 0.49397 | 0.09379 | -0.34891 | -0.27079 |
| S_B_AB_nCi_2_MP2_T_KA_h-v_MAS | 0.1971 | -0.0408 | 0.28730 | 0.0704 | -0.23506 | -0.08930 | 0.01178 | -0.02776 | 0.53365 | -0.02534 | -0.05418 | -0.20099 |
| S_B_AB_nCi_2_MP3_T_KA_h-v_MAS | 0.0436 | -0.0126 | 0.17398 | 0.0474 | -0.37638 | -0.17413 | 0.10866 | 0.08335 | 0.67056 | 0.00359 | -0.08323 | -0.26277 |
| S_B_AB_nCi_2_MP4_T_KA_h-v_MAS | 0.3065 | 0.0169 | 0.16162 | 0.0612 | -0.18526 | -0.10249 | 0.01363 | -0.00415 | 0.54979 | 0.04327 | -0.07520 | -0.18709 |
| S_B_AB_nCi_2_MP5_T_KA_h-v_MAS | 0.0958 | 0.0013 | 0.12459 | 0.0489 | -0.38198 | -0.16444 | 0.12494 | 0.10471 | 0.63693 | 0.00420 | -0.04201 | -0.26937 |
| S_B_AB_nCi_2_MP6_T_KA_h-v_MAS | 0.3764 | 0.0278 | 0.11628 | 0.0619 | -0.17419 | -0.11608 | 0.03152 | 0.03119 | 0.52239 | 0.07426 | -0.06973 | -0.17443 |
| VC_B_AB_nCi_2_MP0_T_KA_h-v_MAS | 0.2014 | -0.0890 | 0.56575 | 0.2954 | -0.18463 | 0.06057 | 0.01332 | -0.03825 | 0.05953 | 0.04770 | 0.14671 | -0.11411 |
| VC_B_AB_nCi_2_MP1_T_KA_h-v_MAS | -0.1355 | -0.4097 | 0.18581 | -0.0841 | -0.14862 | -0.33448 | 0.07355 | 0.14006 | 0.26928 | 0.17198 | -0.20168 | -0.58503 |
| VC_B_AB_nCi_2_MP2_T_KA_h-v_MAS | 0.1631 | -0.3430 | 0.17948 | 0.0629 | 0.22185 | 0.21073 | -0.12304 | -0.00012 | 0.01830 | -0.01904 | 0.19549 | -0.53474 |
| VC_B_AB_nCi_2_MP3_T_KA_h-v_MAS | -0.0749 | -0.3001 | 0.25011 | 0.0567 | -0.24256 | -0.04184 | 0.05137 | 0.06556 | 0.35453 | -0.12396 | -0.04552 | -0.64580 |
| VC_B_AB_nCi_2_MP4_T_KA_h-v_MAS | 0.2121 | -0.1429 | 0.18677 | 0.1454 | 0.06642 | 0.29662 | -0.13074 | -0.05055 | 0.18208 | -0.23774 | 0.15720 | -0.44534 |
| VC_B_AB_nCi_2_MP5_T_KA_h-v_MAS | 0.0329 | -0.1542 | 0.19687 | 0.1165 | -0.24004 | 0.07751 | 0.02542 | 0.05111 | 0.40886 | -0.26040 | 0.02024 | -0.55370 |
| VC_B_AB_nCi_2_MP6_T_KA_h-v_MAS | 0.2768 | -0.0476 | 0.14152 | 0.1687 | 0.01519 | 0.26734 | -0.10174 | -0.02972 | 0.26168 | -0.28783 | 0.10854 | -0.37522 |
| PN_B_AB_nCi_2_SS1_A_KA_a-v_MAS | -0.1255 | -0.3363 | 0.18550 | 0.0017 | -0.26998 | 0.64194 | 0.09664 | 0.06688 | 0.17701 | 0.00129 | 0.13718 | 0.07881 |
| PN_B_AB_nCi_2_SS2_A_KA_a-v_MAS | -0.1789 | -0.4234 | -0.03958 | -0.0579 | -0.32916 | 0.29291 | 0.07205 | 0.15208 | 0.39063 | 0.07502 | 0.00341 | 0.13188 |
| PN_B_AB_nCi_2_SS3_A_KA_a-v_MAS | -0.1301 | -0.3731 | 0.13332 | -0.0282 | -0.36848 | 0.53570 | 0.12503 | 0.10610 | 0.30546 | 0.05600 | 0.09332 | 0.11858 |
| PN_B_AB_nCi_2_SS4_A_KA_a-v_MAS | -0.1559 | -0.4505 | 0.00666 | -0.0401 | -0.33088 | 0.32132 | 0.12428 | 0.14599 | 0.41144 | 0.05239 | -0.00541 | 0.12951 |
| PN_B_AB_nCi_2_SS5_A_KA_a-v_MAS | -0.1311 | -0.3912 | 0.11209 | -0.0413 | -0.39971 | 0.45661 | 0.13939 | 0.11942 | 0.36924 | 0.06621 | 0.05915 | 0.13480 |
| PN_B_AB_nCi_2_SS6_A_KA_a-v_MAS | -0.1454 | -0.4570 | 0.02619 | -0.0363 | -0.34074 | 0.31764 | 0.14026 | 0.14223 | 0.42173 | 0.04290 | -0.01528 | 0.13424 |
| P2_B_AB_nCi_2_SS0_A_KA_a-v_MAS | -0.1095 | -0.0469 | -0.07166 | -0.0873 | -0.56293 | 0.26551 | -0.29211 | -0.01906 | 0.04557 | 0.04310 | -0.06856 | 0.20847 |
| P2_B_AB_nCi_2_SS1_A_KA_a-v_MAS | -0.0758 | 0.0765 | 0.22689 | 0.0857 | -0.38789 | 0.72630 | -0.03141 | -0.01996 | -0.00596 | -0.04430 | 0.09357 | 0.07785 |
| P2_B_AB_nCi_2_SS2_A_KA_a-v_MAS | -0.1223 | -0.0608 | 0.01233 | -0.0709 | -0.58713 | 0.32262 | -0.07214 | 0.00263 | 0.27992 | 0.00132 | -0.15686 | 0.08756 |
| P2_B_AB_nCi_2_SS3_A_KA_a-v_MAS | -0.0963 | -0.0433 | 0.17266 | 0.0147 | -0.55421 | 0.63256 | -0.02656 | 0.01955 | 0.21644 | -0.01788 | 0.02588 | 0.12138 |
| P2_B_AB_nCi_2_SS4_A_KA_a-v_MAS | -0.1301 | -0.1779 | 0.02932 | -0.0633 | -0.58616 | 0.32765 | -0.04766 | 0.02649 | 0.34119 | -0.02483 | -0.16755 | 0.08270 |
| P2_B_AB_nCi_2_SS5_A_KA_a-v_MAS | -0.1054 | -0.1548 | 0.13837 | -0.0256 | -0.61046 | 0.52461 | -0.02896 | 0.04065 | 0.30807 | -0.00007 | -0.02120 | 0.13963 |
| P2_B_AB_nCi_2_SS6_A_KA_a-v_MAS | -0.1316 | -0.2484 | 0.03999 | -0.0634 | -0.58854 | 0.31581 | -0.03791 | 0.03730 | 0.36392 | -0.02749 | -0.16873 | 0.09848 |
| P3_B_AB_nCi_2_SS0_A_KA_a-v_MAS | 0.0557 | 0.0676 | 0.00100 | -0.0588 | 0.07000 | -0.22936 | -0.68680 | -0.05421 | -0.18769 | 0.01981 | 0.01868 | 0.07747 |
| P3_B_AB_nCi_2_SS1_A_KA_a-v_MAS | 0.0137 | -0.0837 | -0.23560 | -0.1752 | 0.11077 | -0.62155 | -0.34219 | -0.02812 | -0.04022 | 0.07639 | -0.14035 | -0.02344 |
| P3_B_AB_nCi_2_SS2_A_KA_a-v_MAS | 0.0606 | 0.0474 | -0.05282 | -0.0482 | 0.18211 | -0.31192 | -0.57545 | -0.04700 | -0.29701 | 0.05460 | 0.08905 | 0.05829 |
| P3_B_AB_nCi_2_SS3_A_KA_a-v_MAS | 0.0163 | -0.0476 | -0.18200 | -0.1438 | 0.16214 | -0.55380 | -0.44908 | -0.05132 | -0.21308 | 0.07722 | -0.08317 | -0.02525 |
| P3_B_AB_nCi_2_SS4_A_KA_a-v_MAS | 0.0584 | 0.0986 | -0.06499 | -0.0566 | 0.25658 | -0.34442 | -0.52331 | -0.04150 | -0.34282 | 0.08966 | 0.11255 | 0.03779 |
| P3_B_AB_nCi_2_SS5_A_KA_a-v_MAS | 0.0218 | 0.0106 | -0.15910 | -0.1187 | 0.18076 | -0.46934 | -0.49178 | -0.06760 | -0.28862 | 0.06458 | -0.05436 | -0.03460 |
| P3_B_AB_nCi_2_SS6_A_KA_a-v_MAS | 0.0542 | 0.1376 | -0.07574 | -0.0606 | 0.27567 | -0.33872 | -0.51274 | -0.04442 | -0.36361 | 0.09787 | 0.11916 | 0.02477 |
| S_B_AB_nCi_2_SS0_A_KA_a-v_MAS | -0.0408 | -0.0298 | -0.05982 | -0.0208 | -0.17516 | 0.01470 | -0.80120 | -0.04451 | -0.04693 | -0.04217 | -0.04080 | 0.00248 |
| S_B_AB_nCi_2_SS3_A_KA_a-v_MAS | -0.1109 | -0.4849 | -0.13738 | -0.1100 | -0.22534 | 0.02217 | -0.59280 | 0.00953 | -0.00359 | -0.00905 | -0.05817 | -0.05519 |
| S_B_AB_nCi_2_SS5_A_KA_a-v_MAS | -0.0627 | -0.4752 | -0.10507 | -0.0791 | -0.23613 | 0.01511 | -0.62965 | 0.00599 | 0.00364 | 0.00617 | -0.07678 | -0.03490 |
| SD_B_AB_nCi_2_SS0_A_KA_a-v_MAS | -0.1170 | -0.2061 | -0.10441 | -0.0998 | -0.60124 | 0.19424 | -0.32213 | -0.03590 | 0.01446 | 0.03609 | -0.17172 | 0.10390 |
| SD_B_AB_nCi_2_SS1_A_KA_a-v_MAS | -0.0566 | -0.1049 | 0.21519 | 0.0787 | -0.46802 | 0.65895 | -0.11998 | -0.07348 | -0.09294 | -0.04087 | -0.02455 | 0.00551 |
| SD_B_AB_nCi_2_SS2_A_KA_a-v_MAS | -0.0985 | -0.1662 | -0.02930 | -0.0742 | -0.58136 | 0.25388 | -0.19984 | -0.05581 | 0.12506 | -0.00514 | -0.18573 | 0.03346 |
| SD_B_AB_nCi_2_SS3_A_KA_a-v_MAS | -0.0546 | -0.0807 | 0.14637 | 0.0312 | -0.59450 | 0.59044 | -0.16304 | -0.06669 | 0.02558 | -0.01731 | -0.03500 | 0.08761 |
| SD_B_AB_nCi_2_SS4_A_KA_a-v_MAS | -0.0893 | -0.1771 | 0.00606 | -0.0562 | -0.59840 | 0.27430 | -0.14784 | -0.05961 | 0.16050 | -0.01822 | -0.18955 | 0.04979 |
| SD_B_AB_nCi_2_SS5_A_KA_a-v_MAS | -0.0510 | -0.0938 | 0.11411 | 0.0022 | -0.65109 | 0.49555 | -0.15670 | -0.05691 | 0.11502 | -0.00394 | -0.06890 | 0.12653 |
| SD_B_AB_nCi_2_SS6_A_KA_a-v_MAS | -0.0829 | -0.1854 | 0.02385 | -0.0479 | -0.61402 | 0.27182 | -0.13080 | -0.05530 | 0.18630 | -0.02241 | -0.19398 | 0.07365 |
| P2_B_AB_nCi_2_SS0_A_KA_h-a_MAS | -0.2823 | -0.0371 | -0.09190 | -0.1319 | -0.40961 | 0.33120 | -0.15040 | 0.03211 | 0.18101 | 0.03325 | -0.19709 | 0.08044 |
| P2_B_AB_nCi_2_SS1_A_KA_h-a_MAS | -0.2168 | 0.0219 | 0.32932 | 0.0995 | -0.09757 | 0.68152 | 0.06784 | -0.00089 | -0.06427 | -0.09118 | -0.04116 | 0.02043 |
| P2_B_AB_nCi_2_SS2_A_KA_h-a_MAS | -0.3000 | -0.0375 | 0.02975 | -0.0969 | -0.39650 | 0.24010 | 0.03953 | 0.03727 | 0.34545 | -0.01767 | -0.37850 | -0.01680 |
| P2_B_AB_nCi_2_SS4_A_KA_h-a_MAS | -0.3156 | -0.1579 | 0.07081 | -0.0750 | -0.39687 | 0.22432 | 0.05170 | 0.05562 | 0.39342 | -0.05711 | -0.40514 | -0.00077 |
| P3_B_AB_nCi_2_SS0_A_KA_h-a_MAS | 0.1503 | 0.0102 | -0.00196 | -0.0300 | -0.10987 | -0.18910 | -0.61875 | -0.12756 | -0.22287 | -0.02083 | -0.03585 | 0.00902 |
| P3_B_AB_nCi_2_SS1_A_KA_h-a_MAS | 0.1485 | -0.0109 | -0.33301 | -0.1505 | 0.02188 | -0.65701 | -0.20934 | -0.00681 | 0.04723 | 0.09222 | 0.01314 | 0.02005 |
| P3_B_AB_nCi_2_SS2_A_KA_h-a_MAS | 0.1731 | -0.0317 | -0.08319 | -0.0259 | -0.03269 | -0.16126 | -0.46986 | -0.10711 | -0.29396 | -0.00613 | 0.08891 | -0.05319 |
| S_B_AB_nCi_2_SS0_A_KA_h-a_MAS | -0.0586 | -0.0618 | -0.06754 | -0.0194 | -0.22003 | 0.05437 | -0.78696 | -0.06470 | -0.07205 | -0.06560 | -0.04146 | 0.01323 |
| S_B_AB_nCi_2_SS1_A_KA_h-a_MAS | -0.2472 | -0.4234 | -0.16933 | -0.1822 | -0.19229 | -0.11301 | -0.40215 | -0.06420 | -0.00359 | -0.05153 | -0.05419 | 0.00658 |
| S_B_AB_nCi_2_SS3_A_KA_h-a_MAS | -0.1364 | -0.5337 | -0.17269 | -0.1571 | -0.22782 | -0.04627 | -0.48449 | -0.02900 | -0.03251 | -0.02129 | -0.03877 | -0.00315 |
| S_B_AB_nCi_2_SS5_A_KA_h-a_MAS | -0.0794 | -0.5683 | -0.14953 | -0.1072 | -0.22051 | -0.04321 | -0.49444 | -0.02688 | -0.03293 | -0.00290 | -0.04617 | 0.00661 |
| SD_B_AB_nCi_2_SS0_A_KA_h-a_MAS | -0.2568 | -0.2199 | -0.13442 | -0.1325 | -0.46261 | 0.25402 | -0.19472 | 0.00414 | 0.12583 | 0.02380 | -0.27139 | -0.00854 |
| SD_B_AB_nCi_2_SS1_A_KA_h-a_MAS | -0.1853 | -0.1596 | 0.31138 | 0.1084 | -0.17858 | 0.66175 | 0.00306 | -0.04368 | -0.12494 | -0.07107 | -0.10586 | -0.04804 |
| SD_B_AB_nCi_2_SS3_A_KA_h-a_MAS | -0.2484 | -0.1496 | 0.26880 | 0.0587 | -0.37174 | 0.64095 | -0.05856 | -0.02934 | 0.05580 | -0.03923 | -0.19352 | 0.02374 |
| SD_B_AB_nCi_2_SS5_A_KA_h-a_MAS | -0.2607 | -0.1511 | 0.22914 | 0.0177 | -0.46688 | 0.49994 | -0.06188 | -0.01421 | 0.20889 | -0.02020 | -0.26787 | 0.06574 |
| PN_B_AB_nCi_2_SS0_A_KA_h-v_MAS | -0.4492 | -0.5872 | -0.19570 | -0.1527 | -0.26466 | 0.08476 | -0.07720 | 0.07428 | 0.07797 | 0.01824 | -0.37492 | -0.14610 |
| PN_B_AB_nCi_2_SS2_A_KA_h-v_MAS | -0.4367 | -0.5818 | -0.14030 | -0.1135 | -0.31928 | 0.07391 | -0.02243 | 0.07008 | 0.13673 | -0.00314 | -0.43211 | -0.13501 |
| PN_B_AB_nCi_2_SS4_A_KA_h-v_MAS | -0.4284 | -0.5850 | -0.10923 | -0.1011 | -0.34161 | 0.06109 | -0.00521 | 0.06890 | 0.15666 | -0.01106 | -0.43688 | -0.11843 |
| PN_B_AB_nCi_2_SS6_A_KA_h-v_MAS | -0.4245 | -0.5846 | -0.09379 | -0.0969 | -0.34957 | 0.05169 | 0.00311 | 0.06960 | 0.17299 | -0.01403 | -0.43263 | -0.10692 |
| P2_B_AB_nCi_2_SS0_A_KA_h-v_MAS | -0.5929 | 0.0475 | -0.07360 | -0.1312 | 0.00142 | 0.08535 | -0.04121 | 0.01536 | 0.01482 | 0.00765 | -0.52374 | 0.06994 |
| P2_B_AB_nCi_2_SS2_A_KA_h-v_MAS | -0.4962 | -0.1103 | -0.01602 | -0.0873 | -0.27223 | -0.05251 | 0.12002 | 0.03927 | 0.19503 | -0.04830 | -0.69152 | -0.05144 |
| P2_B_AB_nCi_2_SS4_A_KA_h-v_MAS | -0.4593 | -0.2791 | -0.01774 | -0.0769 | -0.33763 | -0.03978 | 0.08123 | 0.06528 | 0.26187 | -0.06495 | -0.62761 | -0.05487 |
| P2_B_AB_nCi_2_SS6_A_KA_h-v_MAS | -0.4474 | -0.3548 | -0.01431 | -0.0801 | -0.34645 | -0.03773 | 0.07053 | 0.07146 | 0.27370 | -0.06334 | -0.57744 | -0.04737 |
| P3_B_AB_nCi_2_SS0_A_KA_h-v_MAS | -0.5738 | 0.0385 | -0.07771 | -0.1324 | -0.01388 | 0.08740 | -0.05607 | 0.01216 | 0.02111 | 0.01291 | -0.52899 | 0.06377 |
| P3_B_AB_nCi_2_SS2_A_KA_h-v_MAS | -0.4920 | -0.0798 | -0.01394 | -0.0790 | -0.25075 | -0.03897 | 0.10875 | 0.02421 | 0.18203 | -0.04063 | -0.72200 | -0.03914 |
| P3_B_AB_nCi_2_SS4_A_KA_h-v_MAS | -0.4535 | -0.2188 | -0.00588 | -0.0642 | -0.31936 | -0.04476 | 0.08767 | 0.04509 | 0.25359 | -0.06001 | -0.67809 | -0.03702 |
| P3_B_AB_nCi_2_SS6_A_KA_h-v_MAS | -0.4392 | -0.2867 | 0.00056 | -0.0664 | -0.33298 | -0.04709 | 0.08026 | 0.05261 | 0.28024 | -0.06300 | -0.62903 | -0.02736 |
| HM_B_AB_nCi_2_SS0_A_KA_h-v_MAS | -0.6358 | 0.0695 | -0.06019 | -0.1178 | 0.04046 | 0.08055 | 0.01127 | 0.02769 | -0.00907 | -0.01962 | -0.49465 | 0.08143 |
| S_B_AB_nCi_2_SS3_A_KA_h-v_MAS | 0.1291 | 0.8322 | 0.08526 | 0.0514 | 0.13563 | 0.07454 | 0.02738 | -0.03149 | 0.02624 | -0.00124 | 0.10935 | 0.05296 |
| S_B_AB_nCi_2_SS5_A_KA_h-v_MAS | 0.1069 | 0.8243 | 0.08572 | 0.0235 | 0.16682 | 0.10721 | 0.02059 | -0.02119 | 0.03449 | 0.02061 | 0.13579 | 0.06054 |
| SD_B_AB_nCi_2_SS0_A_KA_h-v_MAS | -0.5549 | -0.2863 | -0.11655 | -0.1209 | -0.07892 | 0.09062 | -0.09397 | 0.00490 | -0.02444 | -0.00144 | -0.48197 | 0.05190 |
| SD_B_AB_nCi_2_SS1_A_KA_h-v_MAS | -0.3944 | -0.0532 | 0.10709 | 0.0360 | -0.24439 | 0.33053 | 0.04628 | -0.07604 | -0.12132 | -0.06385 | -0.54549 | -0.00827 |
| SD_B_AB_nCi_2_SS2_A_KA_h-v_MAS | -0.4927 | -0.2762 | -0.06287 | -0.0676 | -0.25680 | 0.02189 | 0.03923 | -0.00660 | 0.09772 | -0.03442 | -0.69410 | -0.00069 |
| SD_B_AB_nCi_2_SS4_A_KA_h-v_MAS | -0.4346 | -0.2903 | -0.02207 | -0.0386 | -0.31416 | -0.02366 | 0.08201 | -0.01218 | 0.13969 | -0.04666 | -0.69218 | 0.01447 |
| SD_B_AB_nCi_2_SS6_A_KA_h-v_MAS | -0.4089 | -0.2872 | 0.00530 | -0.0284 | -0.33082 | -0.03812 | 0.08893 | -0.00418 | 0.20440 | -0.05968 | -0.66451 | 0.03267 |
| VC_B_AB_nCi_2_SS0_A_KA_h-v_MAS | 0.0545 | 0.9179 | 0.13641 | 0.0319 | 0.18715 | 0.02919 | 0.08449 | -0.01775 | 0.04181 | 0.03528 | 0.05639 | 0.07184 |
| VC_B_AB_nCi_2_SS1_A_KA_h-v_MAS | 0.0308 | 0.9245 | 0.10161 | 0.0195 | 0.12566 | -0.11107 | 0.12863 | -0.01094 | 0.04526 | 0.03564 | -0.06219 | -0.01453 |
| VC_B_AB_nCi_2_SS2_A_KA_h-v_MAS | 0.0467 | 0.9245 | 0.11791 | 0.0320 | 0.12774 | -0.00282 | 0.12397 | -0.01635 | 0.02406 | 0.02030 | -0.07655 | 0.02932 |
| VC_B_AB_nCi_2_SS3_A_KA_h-v_MAS | 0.0674 | 0.9403 | 0.07784 | 0.0347 | 0.12586 | -0.00828 | 0.08586 | -0.01126 | 0.02843 | 0.00090 | -0.02948 | 0.02561 |
| VC_B_AB_nCi_2_SS4_A_KA_h-v_MAS | 0.0655 | 0.9235 | 0.10509 | 0.0460 | 0.09463 | -0.02290 | 0.13867 | -0.02597 | 0.03985 | 0.02805 | -0.11872 | 0.03292 |
| VC_B_AB_nCi_2_SS5_A_KA_h-v_MAS | 0.0774 | 0.9328 | 0.08801 | 0.0466 | 0.14648 | 0.02223 | 0.08490 | -0.01406 | 0.03758 | -0.00737 | -0.01163 | 0.05087 |
| VC_B_AB_nCi_2_SS6_A_KA_h-v_MAS | 0.0749 | 0.9206 | 0.11109 | 0.0502 | 0.08289 | -0.03067 | 0.13773 | -0.02232 | 0.07765 | 0.02603 | -0.12515 | 0.03875 |
| P2_B_AB_nCi_2_MP0_A_KA_a-v_MAS | -0.4783 | -0.0956 | -0.02667 | -0.1415 | -0.47215 | 0.24456 | -0.23384 | 0.03058 | 0.06682 | 0.12639 | -0.11096 | 0.20283 |
| S_B_AB_nCi_2_MP0_A_KA_a-v_MAS | -0.0408 | -0.0298 | -0.05982 | -0.0208 | -0.17516 | 0.01470 | -0.80120 | -0.04451 | -0.04693 | -0.04217 | -0.04080 | 0.00248 |
| S_B_AB_nCi_2_MP1_A_KA_a-v_MAS | -0.0517 | -0.4084 | -0.15589 | -0.0945 | -0.23768 | 0.10062 | -0.53537 | 0.02537 | 0.09061 | 0.05303 | -0.04969 | 0.07984 |
| S_B_AB_nCi_2_MP2_A_KA_a-v_MAS | -0.0746 | -0.2708 | -0.07416 | -0.0550 | -0.02969 | 0.11129 | -0.75177 | 0.02331 | 0.00120 | 0.02197 | 0.11596 | -0.03513 |
| S_B_AB_nCi_2_MP3_A_KA_a-v_MAS | -0.0345 | -0.3150 | -0.03131 | -0.0531 | 0.02048 | 0.12758 | -0.70578 | 0.01418 | -0.00331 | 0.08559 | -0.01084 | -0.00435 |
| S_B_AB_nCi_2_MP4_A_KA_a-v_MAS | -0.0756 | -0.2396 | -0.02687 | -0.0592 | 0.03156 | 0.08950 | -0.74649 | 0.01107 | -0.04022 | 0.05458 | 0.11518 | -0.04808 |
| S_B_AB_nCi_2_MP5_A_KA_a-v_MAS | -0.0492 | -0.2143 | -0.00653 | -0.0687 | 0.03405 | 0.07140 | -0.72266 | -0.01095 | -0.01835 | 0.10137 | -0.01653 | -0.00027 |
| S_B_AB_nCi_2_MP6_A_KA_a-v_MAS | -0.0691 | -0.1858 | 0.00348 | -0.0647 | 0.04900 | 0.04936 | -0.73941 | -0.00771 | -0.04825 | 0.09126 | 0.07448 | -0.04044 |
| S_B_AB_nCi_2_MP0_A_KA_h-a_MAS | -0.0586 | -0.0618 | -0.06754 | -0.0194 | -0.22003 | 0.05437 | -0.78696 | -0.06470 | -0.07205 | -0.06560 | -0.04146 | 0.01323 |
| S_B_AB_nCi_2_MP1_A_KA_h-a_MAS | -0.0978 | -0.4291 | -0.16227 | -0.0862 | -0.32608 | 0.02933 | -0.31079 | -0.03066 | 0.01897 | -0.02881 | -0.08773 | 0.18076 |
| S_B_AB_nCi_2_MP2_A_KA_h-a_MAS | -0.1344 | -0.3191 | -0.08945 | -0.0674 | -0.02638 | 0.16106 | -0.65288 | 0.01839 | -0.03533 | -0.04631 | 0.18232 | -0.02626 |
| S_B_AB_nCi_2_MP3_A_KA_h-a_MAS | -0.0752 | -0.4141 | -0.06373 | -0.0654 | 0.03470 | 0.15340 | -0.54820 | -0.01646 | -0.03950 | 0.02084 | -0.00048 | 0.08763 |
| S_B_AB_nCi_2_MP4_A_KA_h-a_MAS | -0.1406 | -0.2839 | -0.03145 | -0.0682 | 0.08621 | 0.16193 | -0.63832 | 0.01003 | -0.10122 | -0.01792 | 0.22948 | -0.04112 |
| S_B_AB_nCi_2_MP5_A_KA_h-a_MAS | -0.0934 | -0.3026 | 0.00108 | -0.0665 | 0.15352 | 0.16546 | -0.60188 | -0.03057 | -0.08382 | 0.04162 | 0.05804 | 0.06271 |
| S_B_AB_nCi_2_MP6_A_KA_h-a_MAS | -0.1286 | -0.2366 | 0.00885 | -0.0682 | 0.13508 | 0.13287 | -0.66442 | -0.00949 | -0.12542 | 0.02767 | 0.19502 | -0.03264 |
| S_B_AB_nCi_2_MP1_A_KA_h-v_MAS | 0.1494 | 0.5807 | 0.00432 | 0.0405 | -0.00310 | -0.27354 | 0.06923 | -0.00887 | 0.02310 | 0.06379 | -0.30504 | -0.21300 |
| S_B_AB_nCi_2_MP2_A_KA_h-v_MAS | 0.1595 | 0.7776 | 0.05044 | 0.0637 | 0.12659 | 0.04814 | 0.02735 | -0.03929 | 0.06390 | 0.03115 | -0.07576 | 0.01824 |
| S_B_AB_nCi_2_MP3_A_KA_h-v_MAS | 0.1178 | 0.6649 | 0.00255 | 0.0589 | -0.12098 | -0.14220 | 0.08266 | 0.00002 | 0.14926 | -0.00736 | -0.17784 | -0.25686 |
| S_B_AB_nCi_2_MP4_A_KA_h-v_MAS | 0.1435 | 0.8129 | -0.03547 | 0.0606 | 0.01193 | 0.13551 | -0.00470 | 0.01590 | 0.18795 | -0.00841 | -0.01933 | 0.01979 |
| S_B_AB_nCi_2_MP5_A_KA_h-v_MAS | 0.0960 | 0.6546 | -0.07507 | 0.0541 | -0.32053 | -0.01389 | 0.03243 | 0.04802 | 0.24467 | -0.04338 | -0.06596 | -0.18036 |
| S_B_AB_nCi_2_MP6_A_KA_h-v_MAS | 0.1519 | 0.7480 | -0.09925 | 0.0636 | -0.07300 | 0.16231 | -0.02387 | 0.04297 | 0.18680 | -0.01859 | 0.01587 | 0.05054 |
| VC_B_AB_nCi_2_MP0_A_KA_h-v_MAS | 0.0545 | 0.9179 | 0.13641 | 0.0319 | 0.18715 | 0.02919 | 0.08449 | -0.01775 | 0.04181 | 0.03528 | 0.05639 | 0.07184 |
| VC_B_AB_nCi_2_MP1_A_KA_h-v_MAS | -0.0043 | 0.8224 | 0.02223 | -0.0314 | 0.07399 | -0.33539 | 0.16932 | 0.01122 | 0.11515 | 0.05742 | -0.19806 | -0.16669 |
| VC_B_AB_nCi_2_MP2_A_KA_h-v_MAS | 0.0692 | 0.9013 | 0.08897 | 0.0438 | -0.03176 | -0.01220 | 0.12389 | -0.02316 | 0.10771 | -0.00201 | -0.16208 | 0.02734 |
| VC_B_AB_nCi_2_MP3_A_KA_h-v_MAS | 0.0424 | 0.8205 | 0.04138 | 0.0279 | -0.17275 | -0.19218 | 0.15993 | 0.00397 | 0.19546 | -0.02463 | -0.24244 | -0.15780 |
| VC_B_AB_nCi_2_MP4_A_KA_h-v_MAS | 0.1024 | 0.8509 | 0.06447 | 0.0602 | -0.12948 | 0.02822 | 0.13458 | -0.02574 | 0.18491 | -0.01773 | -0.17560 | 0.01618 |
| VC_B_AB_nCi_2_MP5_A_KA_h-v_MAS | 0.0685 | 0.7939 | 0.05220 | 0.0646 | -0.24032 | -0.05417 | 0.12527 | 0.01707 | 0.26299 | -0.08333 | -0.16882 | -0.13047 |
| VC_B_AB_nCi_2_MP6_A_KA_h-v_MAS | 0.1327 | 0.8058 | 0.06175 | 0.0793 | -0.17400 | 0.07998 | 0.11212 | -0.00637 | 0.26005 | -0.04723 | -0.14630 | 0.00817 |
| PN_B_AB_nCi_2_SS0_M_KA_a-v_MAS | 0.0458 | 0.1562 | 0.91253 | 0.0744 | 0.04203 | 0.01552 | 0.07905 | 0.02782 | 0.09669 | 0.12033 | 0.03554 | -0.06417 |
| PN_B_AB_nCi_2_SS1_M_KA_a-v_MAS | 0.0475 | 0.2121 | 0.86052 | 0.0635 | 0.02718 | -0.08411 | 0.08094 | 0.04147 | 0.19996 | 0.11056 | 0.02007 | -0.04171 |
| PN_B_AB_nCi_2_SS2_M_KA_a-v_MAS | 0.0327 | 0.1815 | 0.91550 | 0.0572 | 0.03766 | 0.05376 | 0.07281 | 0.02737 | 0.09726 | 0.09006 | 0.05401 | -0.05723 |
| P2_B_AB_nCi_2_SS2_M_KA_a-v_MAS | 0.1409 | 0.1174 | 0.65813 | 0.5983 | 0.01044 | 0.19226 | 0.01920 | -0.01616 | -0.04978 | -0.06678 | 0.05857 | -0.04524 |
| P2_B_AB_nCi_2_SS4_M_KA_a-v_MAS | 0.1080 | 0.2485 | 0.69429 | 0.4564 | -0.04435 | 0.13561 | 0.00541 | 0.01755 | 0.11666 | -0.07267 | 0.01793 | -0.02571 |
| P3_B_AB_nCi_2_SS2_M_KA_a-v_MAS | -0.1504 | -0.1176 | -0.66113 | -0.6150 | -0.01776 | -0.17843 | -0.03100 | 0.00872 | 0.04541 | 0.03946 | -0.05259 | 0.04936 |
| P3_B_AB_nCi_2_SS4_M_KA_a-v_MAS | -0.1243 | -0.2412 | -0.69359 | -0.5059 | 0.02911 | -0.12668 | -0.02150 | -0.02211 | -0.10686 | 0.04458 | -0.01660 | 0.03179 |
| S_B_AB_nCi_2_SS2_M_KA_a-v_MAS | -0.1869 | -0.0365 | -0.15619 | -0.9505 | -0.01441 | -0.01580 | -0.03330 | -0.01441 | 0.02666 | -0.02397 | -0.00221 | -0.00803 |
| S_B_AB_nCi_2_SS4_M_KA_a-v_MAS | -0.1784 | -0.0504 | -0.07251 | -0.9657 | 0.00164 | -0.01786 | -0.02810 | -0.01340 | 0.00958 | 0.01234 | -0.00103 | -0.02150 |
| S_B_AB_nCi_2_SS6_M_KA_a-v_MAS | -0.1658 | -0.0546 | -0.02629 | -0.9704 | 0.00410 | -0.01398 | -0.02700 | -0.01810 | -0.00374 | 0.03419 | -0.00098 | -0.02508 |
| SD_B_AB_nCi_2_SS0_M_KA_a-v_MAS | 0.1402 | 0.1337 | 0.80370 | 0.4659 | 0.06177 | 0.04178 | 0.06230 | -0.00399 | 0.03202 | 0.12313 | 0.01422 | -0.06512 |
| SD_B_AB_nCi_2_SS1_M_KA_a-v_MAS | 0.1549 | 0.2098 | 0.73127 | 0.4315 | 0.02722 | -0.06107 | 0.05986 | 0.00508 | 0.15508 | 0.08662 | -0.01527 | -0.05067 |
| SD_B_AB_nCi_2_SS2_M_KA_a-v_MAS | 0.1181 | 0.1459 | 0.82681 | 0.4265 | 0.04303 | 0.13474 | 0.04148 | -0.00864 | 0.00386 | 0.03266 | 0.04521 | -0.06742 |
| SD_B_AB_nCi_2_SS4_M_KA_a-v_MAS | 0.1118 | 0.2294 | 0.82695 | 0.3770 | 0.01971 | 0.09459 | 0.04095 | 0.01157 | 0.10006 | 0.04929 | 0.02107 | -0.05286 |
| SD_B_AB_nCi_2_SS6_M_KA_a-v_MAS | 0.1133 | 0.2507 | 0.81567 | 0.3528 | 0.02455 | 0.06775 | 0.04722 | 0.01877 | 0.13639 | 0.07459 | 0.00853 | -0.04619 |
| VC_B_AB_nCi_2_SS6_M_KA_a-v_MAS | -0.1901 | -0.0784 | -0.13169 | -0.9440 | 0.00849 | -0.00716 | -0.01783 | -0.02673 | -0.04348 | 0.04650 | -0.00094 | -0.01261 |
| P2_B_AB_nCi_2_SS2_M_KA_h-a_MAS | -0.0650 | 0.0785 | 0.61370 | 0.5587 | 0.03634 | 0.21403 | 0.03734 | -0.05782 | -0.06932 | -0.18633 | -0.07330 | 0.00362 |
| P3_B_AB_nCi_2_SS2_M_KA_h-a_MAS | 0.0594 | -0.0789 | -0.62070 | -0.5646 | -0.04245 | -0.19999 | -0.05344 | 0.04821 | 0.06617 | 0.16116 | 0.08015 | 0.00428 |
| S_B_AB_nCi_2_SS2_M_KA_h-a_MAS | -0.2294 | -0.0556 | -0.19150 | -0.9239 | -0.02553 | -0.01387 | -0.03443 | -0.03407 | 0.00314 | -0.04886 | 0.00267 | -0.00243 |
| S_B_AB_nCi_2_SS4_M_KA_h-a_MAS | -0.2150 | -0.0663 | -0.12025 | -0.9509 | -0.00890 | -0.01465 | -0.03026 | -0.02964 | -0.01023 | -0.01085 | 0.00310 | -0.00954 |
| S_B_AB_nCi_2_SS6_M_KA_h-a_MAS | -0.1967 | -0.0726 | -0.07610 | -0.9603 | -0.00341 | -0.01204 | -0.02874 | -0.03258 | -0.02094 | 0.01504 | -0.00035 | -0.00999 |
| VC_B_AB_nCi_2_SS6_M_KA_h-a_MAS | -0.1976 | -0.0964 | -0.14764 | -0.9434 | 0.00824 | -0.01634 | -0.01398 | -0.02498 | -0.04011 | 0.04431 | -0.00407 | -0.01187 |
| S_B_AB_nCi_2_SS0_M_KA_h-v_MAS | 0.2212 | 0.0753 | 0.18754 | 0.9423 | 0.02731 | -0.00789 | 0.03102 | 0.02218 | 0.02646 | 0.04418 | -0.00015 | 0.00859 |
| S_B_AB_nCi_2_SS2_M_KA_h-v_MAS | 0.2289 | 0.0768 | 0.18208 | 0.9400 | 0.03384 | 0.00263 | 0.02816 | 0.02120 | 0.01944 | 0.03968 | -0.00117 | 0.00186 |
| S_B_AB_nCi_2_SS4_M_KA_h-v_MAS | 0.2146 | 0.0783 | 0.11023 | 0.9570 | 0.01703 | 0.00673 | 0.02409 | 0.02124 | 0.02305 | 0.00430 | -0.00056 | 0.01085 |
| S_B_AB_nCi_2_SS6_M_KA_h-v_MAS | 0.1974 | 0.0762 | 0.06924 | 0.9626 | 0.00975 | 0.00732 | 0.02251 | 0.02796 | 0.02403 | -0.01826 | 0.00228 | 0.01249 |
| VC_B_AB_nCi_2_SS0_M_KA_h-v_MAS | 0.2135 | 0.0553 | 0.10602 | 0.9597 | 0.01288 | 0.00832 | 0.02551 | 0.01960 | 0.00505 | 0.00654 | 0.01008 | 0.02141 |
| VC_B_AB_nCi_2_SS1_M_KA_h-v_MAS | 0.2204 | 0.0679 | 0.08977 | 0.9539 | 0.00165 | 0.02574 | 0.01871 | 0.02124 | -0.00850 | -0.00770 | 0.00389 | 0.01696 |
| VC_B_AB_nCi_2_SS2_M_KA_h-v_MAS | 0.2038 | 0.0476 | 0.12203 | 0.9521 | 0.01394 | 0.04861 | 0.01993 | 0.01148 | -0.02577 | -0.03929 | 0.02028 | 0.01202 |
| VC_B_AB_nCi_2_SS3_M_KA_h-v_MAS | 0.2011 | 0.0678 | 0.07237 | 0.9595 | -0.01084 | 0.02384 | 0.01709 | 0.03025 | -0.00257 | -0.04133 | 0.00620 | 0.02076 |
| VC_B_AB_nCi_2_SS4_M_KA_h-v_MAS | 0.2027 | 0.0816 | 0.13707 | 0.9526 | 0.00015 | 0.03369 | 0.01238 | 0.01770 | 0.01275 | -0.04825 | 0.01069 | 0.01374 |
| VC_B_AB_nCi_2_SS5_M_KA_h-v_MAS | 0.2036 | 0.0640 | 0.09178 | 0.9594 | 0.00398 | 0.02826 | 0.01859 | 0.02563 | -0.01609 | -0.03087 | 0.01167 | 0.01839 |
| VC_B_AB_nCi_2_SS6_M_KA_h-v_MAS | 0.2050 | 0.0979 | 0.14095 | 0.9477 | -0.00502 | 0.02344 | 0.00913 | 0.02119 | 0.03279 | -0.04498 | 0.00399 | 0.01605 |
| S_B_AB_nCi_2_MP1_M_KA_a-v_MAS | -0.1796 | -0.0155 | -0.12774 | -0.9265 | -0.02477 | -0.04885 | -0.04213 | -0.00483 | 0.07748 | -0.06191 | 0.00179 | -0.00250 |
| S_B_AB_nCi_2_MP2_M_KA_a-v_MAS | -0.1813 | -0.0177 | -0.13745 | -0.9371 | -0.00417 | -0.02808 | -0.03718 | -0.01447 | 0.03979 | -0.01134 | -0.00977 | -0.01919 |
| S_B_AB_nCi_2_MP3_M_KA_a-v_MAS | -0.2068 | -0.0187 | -0.11467 | -0.9245 | -0.00976 | -0.03687 | -0.04540 | -0.02096 | 0.05106 | -0.04591 | 0.00798 | -0.00191 |
| S_B_AB_nCi_2_MP4_M_KA_a-v_MAS | -0.1971 | -0.0166 | -0.11205 | -0.9224 | 0.00838 | -0.03196 | -0.03904 | -0.02598 | 0.03084 | 0.00217 | -0.00068 | -0.01885 |
| S_B_AB_nCi_2_MP5_M_KA_a-v_MAS | -0.2278 | -0.0301 | -0.11597 | -0.9081 | 0.00703 | -0.03457 | -0.04414 | -0.04086 | 0.02666 | -0.02149 | 0.00897 | -0.00174 |
| S_B_AB_nCi_2_MP6_M_KA_a-v_MAS | -0.2210 | -0.0258 | -0.11775 | -0.9001 | 0.01104 | -0.03682 | -0.04070 | -0.04726 | 0.01538 | 0.00513 | 0.00462 | -0.01330 |
| S_B_AB_nCi_2_MP1_M_KA_h-a_MAS | -0.2086 | -0.0198 | -0.14419 | -0.9032 | -0.02792 | -0.05662 | -0.04505 | -0.01460 | 0.08175 | -0.07599 | 0.01347 | 0.00381 |
| S_B_AB_nCi_2_MP2_M_KA_h-a_MAS | -0.2051 | -0.0272 | -0.15592 | -0.9172 | -0.01045 | -0.01884 | -0.04196 | -0.03148 | 0.02875 | -0.03363 | -0.00238 | -0.01283 |
| S_B_AB_nCi_2_MP3_M_KA_h-a_MAS | -0.2158 | 0.0033 | -0.08977 | -0.9077 | -0.01574 | -0.04546 | -0.05079 | -0.02304 | 0.07937 | -0.07006 | 0.01574 | -0.00258 |
| S_B_AB_nCi_2_MP4_M_KA_h-a_MAS | -0.2015 | -0.0097 | -0.11701 | -0.9142 | 0.00673 | -0.02514 | -0.04400 | -0.03283 | 0.04025 | -0.00859 | 0.00560 | -0.01514 |
| S_B_AB_nCi_2_MP5_M_KA_h-a_MAS | -0.2431 | -0.0131 | -0.09393 | -0.8925 | -0.00764 | -0.04104 | -0.05243 | -0.03447 | 0.05101 | -0.05508 | 0.02306 | 0.00362 |
| S_B_AB_nCi_2_MP6_M_KA_h-a_MAS | -0.2135 | -0.0153 | -0.11274 | -0.8988 | 0.00924 | -0.03279 | -0.04556 | -0.04575 | 0.02865 | 0.00379 | 0.00702 | -0.01195 |
| S_B_AB_nCi_2_MP0_M_KA_h-v_MAS | 0.2212 | 0.0753 | 0.18754 | 0.9423 | 0.02731 | -0.00789 | 0.03102 | 0.02218 | 0.02646 | 0.04418 | -0.00015 | 0.00859 |
| S_B_AB_nCi_2_MP2_M_KA_h-v_MAS | 0.2226 | 0.0768 | 0.17439 | 0.9356 | 0.03505 | -0.01737 | 0.03124 | 0.02435 | 0.02563 | 0.04197 | -0.01463 | -0.00254 |
| S_B_AB_nCi_2_MP4_M_KA_h-v_MAS | 0.2156 | 0.0719 | 0.09390 | 0.9371 | -0.00417 | -0.01976 | 0.02679 | 0.03040 | 0.02832 | 0.01214 | -0.02044 | -0.00088 |
| S_B_AB_nCi_2_MP6_M_KA_h-v_MAS | 0.2211 | 0.0815 | 0.06388 | 0.9222 | -0.01517 | -0.02056 | 0.02391 | 0.04811 | 0.04969 | -0.00273 | -0.02307 | -0.00322 |
| VC_B_AB_nCi_2_MP0_M_KA_h-v_MAS | 0.2135 | 0.0553 | 0.10602 | 0.9597 | 0.01288 | 0.00832 | 0.02551 | 0.01960 | 0.00505 | 0.00654 | 0.01008 | 0.02141 |
| VC_B_AB_nCi_2_MP1_M_KA_h-v_MAS | 0.2206 | 0.0789 | 0.12236 | 0.9453 | 0.00979 | 0.00933 | 0.02282 | 0.02176 | -0.00346 | -0.00127 | 0.00193 | 0.01098 |
| VC_B_AB_nCi_2_MP2_M_KA_h-v_MAS | 0.2078 | 0.0570 | 0.10329 | 0.9510 | 0.00650 | 0.02487 | 0.01987 | 0.02214 | -0.00007 | -0.04612 | 0.00419 | 0.00969 |
| VC_B_AB_nCi_2_MP3_M_KA_h-v_MAS | 0.2190 | 0.0777 | 0.08912 | 0.9388 | -0.01568 | -0.00616 | 0.02294 | 0.04342 | 0.02864 | -0.02830 | -0.02043 | 0.01230 |
| VC_B_AB_nCi_2_MP4_M_KA_h-v_MAS | 0.2145 | 0.0824 | 0.12402 | 0.9339 | -0.01496 | -0.01066 | 0.01414 | 0.04109 | 0.05539 | -0.03876 | -0.01736 | 0.00453 |
| VC_B_AB_nCi_2_MP5_M_KA_h-v_MAS | 0.2413 | 0.0784 | 0.11587 | 0.9281 | -0.01290 | -0.01739 | 0.02395 | 0.04841 | 0.04823 | -0.00864 | -0.02340 | 0.00330 |
| VC_B_AB_nCi_2_MP6_M_KA_h-v_MAS | 0.2307 | 0.0919 | 0.13898 | 0.9162 | -0.02124 | -0.03148 | 0.01625 | 0.05065 | 0.08915 | -0.02387 | -0.02735 | 0.00134 |
| PN_B_AB_nCi_2_SS1_X_KA_a-v_MAS | -0.1542 | -0.3338 | 0.17368 | 0.0000 | -0.34383 | 0.56041 | 0.12020 | 0.07547 | 0.16826 | -0.01084 | 0.17828 | 0.01712 |
| PN_B_AB_nCi_2_SS2_X_KA_a-v_MAS | -0.2211 | -0.3867 | -0.02147 | -0.0752 | -0.42506 | 0.18368 | 0.09356 | 0.15768 | 0.34268 | 0.06325 | 0.12670 | 0.04862 |
| PN_B_AB_nCi_2_SS3_X_KA_a-v_MAS | -0.1636 | -0.3567 | 0.12564 | -0.0380 | -0.46022 | 0.43611 | 0.15640 | 0.10970 | 0.28460 | 0.04282 | 0.15653 | 0.04071 |
| PN_B_AB_nCi_2_SS4_X_KA_a-v_MAS | -0.2040 | -0.4027 | 0.04182 | -0.0606 | -0.46531 | 0.18833 | 0.14597 | 0.14664 | 0.36797 | 0.02978 | 0.14371 | 0.00280 |
| PN_B_AB_nCi_2_SS6_X_KA_a-v_MAS | -0.1953 | -0.4004 | 0.06652 | -0.0567 | -0.49042 | 0.17097 | 0.15641 | 0.13971 | 0.38136 | 0.01205 | 0.14970 | -0.00830 |
| P2_B_AB_nCi_2_SS0_X_KA_a-v_MAS | -0.1634 | -0.0158 | -0.02196 | -0.0624 | -0.66964 | 0.10986 | -0.37454 | 0.00109 | 0.01847 | -0.00572 | 0.06455 | 0.19243 |
| P2_B_AB_nCi_2_SS1_X_KA_a-v_MAS | -0.1115 | 0.0678 | 0.25111 | 0.0832 | -0.50599 | 0.60850 | -0.02477 | -0.01619 | -0.01296 | -0.07234 | 0.18664 | 0.03405 |
| P2_B_AB_nCi_2_SS2_X_KA_a-v_MAS | -0.1890 | -0.0626 | 0.06802 | -0.0629 | -0.74513 | 0.14879 | -0.11584 | 0.02639 | 0.24259 | -0.03487 | -0.00199 | 0.02631 |
| P2_B_AB_nCi_2_SS3_X_KA_a-v_MAS | -0.1438 | -0.0550 | 0.18328 | 0.0126 | -0.70107 | 0.47950 | -0.01071 | 0.02867 | 0.19121 | -0.04046 | 0.12407 | 0.04825 |
| P2_B_AB_nCi_2_SS4_X_KA_a-v_MAS | -0.1983 | -0.1627 | 0.09798 | -0.0593 | -0.76510 | 0.12863 | -0.07028 | 0.04470 | 0.28369 | -0.05814 | 0.02902 | -0.03682 |
| P2_B_AB_nCi_2_SS5_X_KA_a-v_MAS | -0.1540 | -0.1498 | 0.14535 | -0.0239 | -0.76178 | 0.35717 | -0.01174 | 0.04592 | 0.26301 | -0.02332 | 0.09389 | 0.04413 |
| P2_B_AB_nCi_2_SS6_X_KA_a-v_MAS | -0.1994 | -0.2147 | 0.11097 | -0.0602 | -0.77067 | 0.10730 | -0.05307 | 0.05244 | 0.29946 | -0.06286 | 0.04707 | -0.04651 |
| P3_B_AB_nCi_2_SS0_X_KA_a-v_MAS | -0.0168 | 0.0392 | 0.05750 | -0.0133 | -0.14308 | -0.30532 | -0.71644 | -0.02699 | -0.16533 | -0.01691 | 0.07606 | 0.14243 |
| P3_B_AB_nCi_2_SS1_X_KA_a-v_MAS | -0.0152 | -0.0768 | -0.16533 | -0.1693 | 0.08764 | -0.61963 | -0.40326 | -0.01789 | -0.02407 | 0.04572 | -0.07275 | 0.01143 |
| P3_B_AB_nCi_2_SS2_X_KA_a-v_MAS | 0.0103 | 0.0365 | -0.02819 | -0.0165 | 0.17256 | -0.32230 | -0.61531 | -0.02173 | -0.23759 | 0.01696 | 0.07298 | 0.19036 |
| P3_B_AB_nCi_2_SS3_X_KA_a-v_MAS | -0.0061 | -0.0380 | -0.14007 | -0.1378 | 0.22167 | -0.52378 | -0.50072 | -0.03300 | -0.18316 | 0.06558 | -0.06686 | 0.05090 |
| P3_B_AB_nCi_2_SS4_X_KA_a-v_MAS | 0.0328 | 0.0873 | -0.09277 | -0.0409 | 0.36763 | -0.26846 | -0.51762 | -0.01791 | -0.28950 | 0.08616 | 0.00483 | 0.22902 |
| P3_B_AB_nCi_2_SS5_X_KA_a-v_MAS | 0.0076 | 0.0137 | -0.14217 | -0.1098 | 0.29317 | -0.40986 | -0.52596 | -0.04302 | -0.26086 | 0.07399 | -0.08349 | 0.06819 |
| P3_B_AB_nCi_2_SS6_X_KA_a-v_MAS | 0.0429 | 0.1177 | -0.11795 | -0.0465 | 0.41442 | -0.23929 | -0.49278 | -0.02338 | -0.31150 | 0.10706 | -0.01437 | 0.22541 |
| S_B_AB_nCi_2_SS0_X_KA_a-v_MAS | -0.0419 | -0.0466 | -0.03414 | 0.0122 | -0.29197 | -0.05395 | -0.79377 | -0.03047 | -0.05419 | -0.07297 | -0.01908 | 0.04373 |
| S_B_AB_nCi_2_SS2_X_KA_a-v_MAS | -0.0477 | -0.2623 | -0.08154 | -0.0126 | -0.25209 | -0.04588 | -0.76862 | 0.00842 | -0.00086 | -0.02759 | -0.00141 | 0.04911 |
| S_B_AB_nCi_2_SS3_X_KA_a-v_MAS | -0.1195 | -0.4567 | -0.09947 | -0.1075 | -0.26463 | -0.03474 | -0.64777 | 0.01670 | -0.00382 | -0.03391 | -0.00807 | -0.03045 |
| S_B_AB_nCi_2_SS4_X_KA_a-v_MAS | -0.0474 | -0.3525 | -0.08350 | -0.0120 | -0.20183 | -0.04128 | -0.74549 | 0.01634 | -0.00758 | -0.01159 | -0.02816 | 0.05398 |
| S_B_AB_nCi_2_SS5_X_KA_a-v_MAS | -0.0716 | -0.4533 | -0.07794 | -0.0761 | -0.26016 | -0.03507 | -0.68599 | 0.01867 | -0.00315 | -0.01219 | -0.04285 | -0.00451 |
| S_B_AB_nCi_2_SS6_X_KA_a-v_MAS | -0.0365 | -0.3708 | -0.07086 | -0.0146 | -0.18653 | -0.05616 | -0.73914 | 0.01671 | 0.00106 | -0.00419 | -0.04975 | 0.06235 |
| SD_B_AB_nCi_2_SS0_X_KA_a-v_MAS | -0.1852 | -0.1769 | -0.03777 | -0.0700 | -0.73641 | 0.02045 | -0.37239 | -0.00459 | -0.00258 | -0.00514 | -0.02813 | 0.07508 |
| SD_B_AB_nCi_2_SS1_X_KA_a-v_MAS | -0.1069 | -0.1001 | 0.25168 | 0.0737 | -0.61775 | 0.47942 | -0.13732 | -0.06372 | -0.09224 | -0.07307 | 0.10131 | -0.03250 |
| SD_B_AB_nCi_2_SS2_X_KA_a-v_MAS | -0.1779 | -0.1552 | 0.04239 | -0.0600 | -0.75726 | 0.05768 | -0.22971 | -0.01954 | 0.09674 | -0.04642 | -0.00542 | -0.03808 |
| SD_B_AB_nCi_2_SS3_X_KA_a-v_MAS | -0.1188 | -0.0787 | 0.17192 | 0.0351 | -0.76329 | 0.38903 | -0.15520 | -0.04942 | 0.00599 | -0.04714 | 0.09359 | 0.00867 |
| SD_B_AB_nCi_2_SS4_X_KA_a-v_MAS | -0.1736 | -0.1579 | 0.09049 | -0.0470 | -0.77738 | 0.06355 | -0.16625 | -0.01969 | 0.12475 | -0.05715 | 0.01807 | -0.06662 |
| SD_B_AB_nCi_2_SS5_X_KA_a-v_MAS | -0.1181 | -0.0922 | 0.13178 | 0.0101 | -0.81790 | 0.29328 | -0.13616 | -0.03762 | 0.07981 | -0.03174 | 0.06115 | 0.02128 |
| SD_B_AB_nCi_2_SS6_X_KA_a-v_MAS | -0.1702 | -0.1604 | 0.10692 | -0.0407 | -0.79136 | 0.05455 | -0.14056 | -0.01375 | 0.14718 | -0.06151 | 0.02666 | -0.06832 |
| P2_B_AB_nCi_2_SS0_X_KA_h-a_MAS | -0.3182 | -0.0285 | -0.07435 | -0.1315 | -0.44329 | 0.28524 | -0.19667 | 0.04306 | 0.18324 | 0.01864 | -0.15147 | 0.10078 |
| P2_B_AB_nCi_2_SS1_X_KA_h-a_MAS | -0.2283 | 0.0155 | 0.33334 | 0.0998 | -0.12202 | 0.65633 | 0.08275 | 0.00008 | -0.07083 | -0.09540 | -0.01917 | 0.01128 |
| P2_B_AB_nCi_2_SS2_X_KA_h-a_MAS | -0.3302 | -0.0421 | 0.04711 | -0.0982 | -0.42852 | 0.19532 | 0.01062 | 0.04867 | 0.34630 | -0.02262 | -0.34496 | -0.00110 |
| P2_B_AB_nCi_2_SS4_X_KA_h-a_MAS | -0.3471 | -0.1625 | 0.08951 | -0.0773 | -0.45438 | 0.17031 | 0.02967 | 0.06538 | 0.38885 | -0.06440 | -0.35577 | -0.00355 |
| P3_B_AB_nCi_2_SS0_X_KA_h-a_MAS | 0.0833 | 0.0148 | 0.02065 | -0.0276 | -0.18390 | -0.23822 | -0.66062 | -0.10732 | -0.21558 | -0.02900 | 0.00803 | 0.05469 |
| P3_B_AB_nCi_2_SS1_X_KA_h-a_MAS | 0.1285 | -0.0089 | -0.31281 | -0.1502 | 0.02487 | -0.66524 | -0.24576 | 0.00194 | 0.05392 | 0.08396 | 0.02739 | 0.04774 |
| P3_B_AB_nCi_2_SS2_X_KA_h-a_MAS | 0.1073 | -0.0291 | -0.05774 | -0.0275 | -0.10479 | -0.21247 | -0.50077 | -0.08660 | -0.28094 | -0.01741 | 0.12716 | -0.01678 |
| S_B_AB_nCi_2_SS0_X_KA_h-a_MAS | -0.0721 | -0.0574 | -0.04154 | -0.0044 | -0.29764 | -0.00469 | -0.81034 | -0.05926 | -0.07837 | -0.08688 | -0.00857 | 0.04269 |
| S_B_AB_nCi_2_SS1_X_KA_h-a_MAS | -0.2591 | -0.4004 | -0.12904 | -0.1746 | -0.24326 | -0.16480 | -0.44605 | -0.05828 | -0.00064 | -0.07333 | -0.00172 | 0.01946 |
| S_B_AB_nCi_2_SS2_X_KA_h-a_MAS | -0.1080 | -0.3122 | -0.08797 | -0.0273 | -0.33835 | -0.08756 | -0.69247 | -0.02473 | -0.05153 | -0.06681 | 0.00159 | 0.02825 |
| S_B_AB_nCi_2_SS3_X_KA_h-a_MAS | -0.1523 | -0.5151 | -0.14431 | -0.1488 | -0.27990 | -0.09876 | -0.51235 | -0.02049 | -0.03232 | -0.04228 | 0.00866 | 0.01306 |
| S_B_AB_nCi_2_SS4_X_KA_h-a_MAS | -0.1157 | -0.4483 | -0.09828 | -0.0280 | -0.31268 | -0.08747 | -0.60913 | -0.00967 | -0.06247 | -0.05763 | 0.00558 | 0.00815 |
| S_B_AB_nCi_2_SS5_X_KA_h-a_MAS | -0.0965 | -0.5569 | -0.12890 | -0.0998 | -0.27170 | -0.09419 | -0.52537 | -0.01713 | -0.03405 | -0.02104 | -0.00672 | 0.02319 |
| S_B_AB_nCi_2_SS6_X_KA_h-a_MAS | -0.1074 | -0.4909 | -0.09453 | -0.0362 | -0.30097 | -0.09350 | -0.57948 | -0.00265 | -0.06141 | -0.04746 | 0.00194 | 0.00561 |
| SD_B_AB_nCi_2_SS0_X_KA_h-a_MAS | -0.3080 | -0.2176 | -0.10601 | -0.1288 | -0.52373 | 0.18828 | -0.23269 | 0.02616 | 0.12720 | 0.01179 | -0.22259 | 0.01097 |
| SD_B_AB_nCi_2_SS1_X_KA_h-a_MAS | -0.2125 | -0.1628 | 0.32349 | 0.1073 | -0.24474 | 0.60938 | -0.01033 | -0.03603 | -0.12869 | -0.08031 | -0.06827 | -0.04577 |
| SD_B_AB_nCi_2_SS3_X_KA_h-a_MAS | -0.2843 | -0.1535 | 0.27842 | 0.0616 | -0.46629 | 0.56049 | -0.07161 | -0.01854 | 0.04328 | -0.04823 | -0.14235 | 0.01260 |
| SD_B_AB_nCi_2_SS5_X_KA_h-a_MAS | -0.2959 | -0.1560 | 0.23307 | 0.0226 | -0.56093 | 0.41480 | -0.07204 | -0.00183 | 0.18975 | -0.02837 | -0.21617 | 0.04718 |
| PN_B_AB_nCi_2_SS0_X_KA_h-v_MAS | -0.5004 | -0.5582 | -0.15178 | -0.1447 | -0.38646 | -0.01304 | -0.11395 | 0.10111 | 0.07429 | 0.00369 | -0.27963 | -0.13672 |
| PN_B_AB_nCi_2_SS2_X_KA_h-v_MAS | -0.4839 | -0.5472 | -0.09206 | -0.1059 | -0.44173 | -0.02826 | -0.05809 | 0.09600 | 0.12946 | -0.02016 | -0.31964 | -0.13473 |
| PN_B_AB_nCi_2_SS3_X_KA_h-v_MAS | -0.4610 | -0.5677 | -0.01773 | -0.0878 | -0.48691 | 0.07164 | -0.05634 | 0.08095 | 0.10574 | -0.01901 | -0.25015 | -0.09956 |
| PN_B_AB_nCi_2_SS4_X_KA_h-v_MAS | -0.4734 | -0.5456 | -0.06000 | -0.0942 | -0.46719 | -0.04409 | -0.04068 | 0.09363 | 0.14758 | -0.02957 | -0.31360 | -0.12424 |
| PN_B_AB_nCi_2_SS5_X_KA_h-v_MAS | -0.4584 | -0.5616 | -0.02458 | -0.0904 | -0.49789 | 0.02471 | -0.04905 | 0.08534 | 0.13474 | -0.01866 | -0.25480 | -0.09448 |
| PN_B_AB_nCi_2_SS6_X_KA_h-v_MAS | -0.4683 | -0.5433 | -0.04554 | -0.0902 | -0.47661 | -0.05395 | -0.03164 | 0.09347 | 0.16223 | -0.03321 | -0.30578 | -0.11602 |
| P2_B_AB_nCi_2_SS0_X_KA_h-v_MAS | -0.6484 | 0.0452 | -0.04373 | -0.1342 | -0.03557 | 0.01924 | -0.08997 | 0.03618 | 0.02271 | -0.00946 | -0.45873 | 0.12511 |
| P2_B_AB_nCi_2_SS2_X_KA_h-v_MAS | -0.5396 | -0.1220 | 0.01757 | -0.0855 | -0.32230 | -0.12136 | 0.07434 | 0.06364 | 0.19833 | -0.05668 | -0.61885 | -0.00930 |
| P2_B_AB_nCi_2_SS4_X_KA_h-v_MAS | -0.4975 | -0.2798 | 0.01183 | -0.0740 | -0.41872 | -0.12313 | 0.04125 | 0.08543 | 0.25074 | -0.07630 | -0.53195 | -0.03534 |
| P2_B_AB_nCi_2_SS6_X_KA_h-v_MAS | -0.4833 | -0.3471 | 0.01655 | -0.0758 | -0.43716 | -0.12231 | 0.03030 | 0.08901 | 0.25894 | -0.07686 | -0.47514 | -0.03580 |
| P3_B_AB_nCi_2_SS0_X_KA_h-v_MAS | -0.6302 | 0.0369 | -0.04669 | -0.1363 | -0.05638 | 0.01881 | -0.10762 | 0.03306 | 0.02876 | -0.00375 | -0.46078 | 0.12003 |
| P3_B_AB_nCi_2_SS1_X_KA_h-v_MAS | -0.5029 | -0.0423 | 0.11758 | -0.0005 | -0.44752 | 0.12762 | 0.08534 | -0.00789 | -0.05957 | -0.10024 | -0.41074 | -0.08736 |
| P3_B_AB_nCi_2_SS2_X_KA_h-v_MAS | -0.5391 | -0.0914 | 0.02073 | -0.0783 | -0.30263 | -0.11157 | 0.06338 | 0.04972 | 0.18632 | -0.05147 | -0.64559 | 0.00492 |
| P3_B_AB_nCi_2_SS4_X_KA_h-v_MAS | -0.4955 | -0.2230 | 0.02537 | -0.0633 | -0.39585 | -0.12655 | 0.04693 | 0.06737 | 0.24633 | -0.07316 | -0.58278 | -0.01308 |
| P3_B_AB_nCi_2_SS6_X_KA_h-v_MAS | -0.4787 | -0.2842 | 0.03177 | -0.0643 | -0.41777 | -0.12855 | 0.03997 | 0.07238 | 0.26910 | -0.07765 | -0.52849 | -0.01192 |
| HM_B_AB_nCi_2_SS0_X_KA_h-v_MAS | -0.6858 | 0.0648 | -0.03497 | -0.1190 | 0.01682 | 0.02044 | -0.02648 | 0.04702 | -0.00186 | -0.03833 | -0.43741 | 0.12966 |
| S_B_AB_nCi_2_SS3_X_KA_h-v_MAS | 0.1227 | 0.8219 | 0.08832 | 0.0437 | 0.04703 | 0.02791 | 0.10499 | -0.00322 | 0.02579 | -0.01374 | 0.16332 | -0.06044 |
| S_B_AB_nCi_2_SS5_X_KA_h-v_MAS | 0.1012 | 0.8269 | 0.09119 | 0.0100 | 0.06833 | 0.06098 | 0.10229 | 0.00293 | 0.03289 | 0.00417 | 0.19787 | -0.05530 |
| SD_B_AB_nCi_2_SS0_X_KA_h-v_MAS | -0.6014 | -0.2705 | -0.08719 | -0.1230 | -0.11756 | 0.02615 | -0.15723 | 0.01939 | -0.02213 | -0.02374 | -0.40891 | 0.12122 |
| SD_B_AB_nCi_2_SS1_X_KA_h-v_MAS | -0.4071 | -0.0518 | 0.11233 | 0.0290 | -0.46420 | 0.14147 | 0.08693 | -0.05829 | -0.12893 | -0.09019 | -0.33008 | -0.13248 |
| SD_B_AB_nCi_2_SS2_X_KA_h-v_MAS | -0.5428 | -0.2682 | -0.02197 | -0.0687 | -0.33977 | -0.06694 | -0.00840 | 0.01929 | 0.09757 | -0.05522 | -0.58553 | 0.03901 |
| SD_B_AB_nCi_2_SS3_X_KA_h-v_MAS | -0.4376 | -0.1636 | 0.03578 | -0.0126 | -0.55190 | 0.09107 | -0.00488 | -0.01400 | -0.00314 | -0.11637 | -0.28631 | -0.04676 |
| SD_B_AB_nCi_2_SS4_X_KA_h-v_MAS | -0.4785 | -0.2809 | 0.02169 | -0.0381 | -0.38247 | -0.10037 | 0.03331 | 0.01181 | 0.14159 | -0.06836 | -0.59044 | 0.05116 |
| SD_B_AB_nCi_2_SS5_X_KA_h-v_MAS | -0.4278 | -0.2079 | 0.04969 | -0.0104 | -0.54550 | 0.03488 | 0.00987 | -0.00376 | 0.09105 | -0.12339 | -0.28823 | -0.00291 |
| SD_B_AB_nCi_2_SS6_X_KA_h-v_MAS | -0.4493 | -0.2769 | 0.04328 | -0.0278 | -0.39460 | -0.11002 | 0.04481 | 0.01833 | 0.20406 | -0.08041 | -0.56755 | 0.06336 |
| VC_B_AB_nCi_2_SS0_X_KA_h-v_MAS | 0.0640 | 0.8918 | 0.12635 | 0.0252 | 0.28622 | 0.04633 | 0.11262 | -0.01557 | 0.05630 | 0.04452 | 0.02570 | 0.10579 |
| VC_B_AB_nCi_2_SS1_X_KA_h-v_MAS | 0.0397 | 0.9102 | 0.09460 | 0.0148 | 0.14867 | -0.12039 | 0.17769 | -0.00871 | 0.04965 | 0.03876 | -0.05590 | -0.02204 |
| VC_B_AB_nCi_2_SS2_X_KA_h-v_MAS | 0.0474 | 0.9028 | 0.11607 | 0.0234 | 0.20516 | -0.00379 | 0.15361 | -0.01088 | 0.04195 | 0.02664 | -0.09341 | 0.06140 |
| VC_B_AB_nCi_2_SS3_X_KA_h-v_MAS | 0.0678 | 0.9342 | 0.07126 | 0.0279 | 0.13416 | -0.03161 | 0.14131 | -0.00446 | 0.03425 | -0.00186 | -0.01135 | 0.00691 |
| VC_B_AB_nCi_2_SS4_X_KA_h-v_MAS | 0.0673 | 0.8997 | 0.10658 | 0.0377 | 0.18420 | -0.01929 | 0.16450 | -0.01917 | 0.06114 | 0.03481 | -0.14068 | 0.07015 |
| VC_B_AB_nCi_2_SS5_X_KA_h-v_MAS | 0.0785 | 0.9292 | 0.08052 | 0.0406 | 0.15626 | -0.00418 | 0.14312 | -0.00665 | 0.04490 | -0.01007 | 0.00483 | 0.03057 |
| VC_B_AB_nCi_2_SS6_X_KA_h-v_MAS | 0.0780 | 0.8966 | 0.10902 | 0.0428 | 0.17564 | -0.02456 | 0.16460 | -0.01565 | 0.09852 | 0.03405 | -0.14993 | 0.07616 |
| S_B_AB_nCi_2_MP0_X_KA_a-v_MAS | -0.0419 | -0.0466 | -0.03414 | 0.0122 | -0.29197 | -0.05395 | -0.79377 | -0.03047 | -0.05419 | -0.07297 | -0.01908 | 0.04373 |
| S_B_AB_nCi_2_MP1_X_KA_a-v_MAS | -0.0658 | -0.3731 | -0.08745 | -0.0700 | -0.28120 | 0.03402 | -0.60984 | 0.01874 | 0.09871 | 0.01550 | 0.00433 | 0.09665 |
| S_B_AB_nCi_2_MP2_X_KA_a-v_MAS | -0.0741 | -0.2253 | -0.02770 | -0.0127 | -0.10886 | 0.07523 | -0.78930 | 0.02370 | -0.00857 | -0.01408 | 0.13289 | 0.02740 |
| S_B_AB_nCi_2_MP3_X_KA_a-v_MAS | -0.0403 | -0.2648 | 0.02544 | -0.0256 | -0.02607 | 0.09934 | -0.76833 | 0.01080 | 0.00814 | 0.04365 | 0.03905 | 0.02332 |
| S_B_AB_nCi_2_MP4_X_KA_a-v_MAS | -0.0765 | -0.2018 | 0.01044 | -0.0242 | -0.02922 | 0.07394 | -0.78785 | 0.00931 | -0.04223 | 0.02147 | 0.12954 | 0.01599 |
| S_B_AB_nCi_2_MP5_X_KA_a-v_MAS | -0.0546 | -0.1762 | 0.04114 | -0.0376 | -0.00691 | 0.05598 | -0.77172 | -0.00719 | -0.00475 | 0.06272 | 0.02716 | 0.02882 |
| S_B_AB_nCi_2_MP6_X_KA_a-v_MAS | -0.0729 | -0.1594 | 0.03611 | -0.0362 | -0.00146 | 0.04002 | -0.77880 | -0.00796 | -0.04680 | 0.05920 | 0.09076 | 0.02093 |
| S_B_AB_nCi_2_MP0_X_KA_h-a_MAS | -0.0721 | -0.0574 | -0.04154 | -0.0044 | -0.29764 | -0.00469 | -0.81034 | -0.05926 | -0.07837 | -0.08688 | -0.00857 | 0.04269 |
| S_B_AB_nCi_2_MP1_X_KA_h-a_MAS | -0.1218 | -0.4022 | -0.10589 | -0.0811 | -0.29852 | 0.00192 | -0.39552 | -0.02486 | 0.04719 | -0.04606 | -0.06169 | 0.20793 |
| S_B_AB_nCi_2_MP2_X_KA_h-a_MAS | -0.1363 | -0.2621 | -0.05941 | -0.0327 | -0.14963 | 0.11021 | -0.69219 | 0.02081 | -0.06125 | -0.07675 | 0.23151 | -0.00753 |
| S_B_AB_nCi_2_MP3_X_KA_h-a_MAS | -0.0863 | -0.3469 | -0.00700 | -0.0531 | 0.05987 | 0.14789 | -0.62238 | -0.01185 | -0.01260 | -0.00412 | 0.03257 | 0.12829 |
| S_B_AB_nCi_2_MP4_X_KA_h-a_MAS | -0.1386 | -0.2327 | -0.01013 | -0.0319 | -0.03620 | 0.12315 | -0.68002 | 0.01248 | -0.12212 | -0.05045 | 0.26938 | -0.01748 |
| S_B_AB_nCi_2_MP5_X_KA_h-a_MAS | -0.0972 | -0.2404 | 0.04696 | -0.0457 | 0.16087 | 0.16587 | -0.65630 | -0.02063 | -0.05472 | 0.01397 | 0.08188 | 0.10531 |
| S_B_AB_nCi_2_MP6_X_KA_h-a_MAS | -0.1308 | -0.1961 | 0.02811 | -0.0353 | 0.02220 | 0.10012 | -0.69965 | -0.00781 | -0.14115 | -0.00551 | 0.23171 | -0.00516 |
| S_B_AB_nCi_2_MP1_X_KA_h-v_MAS | 0.1660 | 0.5493 | -0.01326 | 0.0529 | -0.08565 | -0.30421 | 0.13091 | -0.01727 | -0.00566 | 0.04546 | -0.24245 | -0.23622 |
| S_B_AB_nCi_2_MP2_X_KA_h-v_MAS | 0.1670 | 0.7567 | 0.05212 | 0.0562 | 0.22018 | 0.06637 | 0.03446 | -0.03543 | 0.07868 | 0.02793 | -0.08444 | 0.05248 |
| S_B_AB_nCi_2_MP3_X_KA_h-v_MAS | 0.1157 | 0.6155 | -0.01870 | 0.0567 | -0.19057 | -0.17279 | 0.14008 | 0.00514 | 0.12740 | -0.02952 | -0.13616 | -0.25961 |
| S_B_AB_nCi_2_MP4_X_KA_h-v_MAS | 0.1469 | 0.8053 | -0.04713 | 0.0537 | 0.03958 | 0.12225 | 0.01780 | 0.01915 | 0.18908 | -0.01516 | -0.00854 | 0.03282 |
| S_B_AB_nCi_2_MP5_X_KA_h-v_MAS | 0.0823 | 0.6060 | -0.08798 | 0.0512 | -0.38678 | -0.05561 | 0.08390 | 0.05314 | 0.21234 | -0.06409 | -0.02740 | -0.18603 |
| S_B_AB_nCi_2_MP6_X_KA_h-v_MAS | 0.1561 | 0.7429 | -0.11666 | 0.0587 | -0.08584 | 0.12803 | 0.01099 | 0.04052 | 0.17630 | -0.02833 | 0.04013 | 0.04836 |
| VC_B_AB_nCi_2_MP0_X_KA_h-v_MAS | 0.0640 | 0.8918 | 0.12635 | 0.0252 | 0.28622 | 0.04633 | 0.11262 | -0.01557 | 0.05630 | 0.04452 | 0.02570 | 0.10579 |
| VC_B_AB_nCi_2_MP1_X_KA_h-v_MAS | 0.0051 | 0.7946 | 0.01176 | -0.0275 | 0.01816 | -0.36254 | 0.22914 | 0.01064 | 0.10049 | 0.05139 | -0.15675 | -0.19543 |
| VC_B_AB_nCi_2_MP2_X_KA_h-v_MAS | 0.0618 | 0.8933 | 0.09852 | 0.0360 | 0.00871 | -0.03725 | 0.14192 | -0.01500 | 0.12299 | -0.00854 | -0.15270 | 0.04941 |
| VC_B_AB_nCi_2_MP3_X_KA_h-v_MAS | 0.0446 | 0.7560 | 0.02136 | 0.0310 | -0.27920 | -0.24754 | 0.21816 | 0.00936 | 0.15618 | -0.04949 | -0.14772 | -0.19732 |
| VC_B_AB_nCi_2_MP4_X_KA_h-v_MAS | 0.0970 | 0.8489 | 0.07482 | 0.0557 | -0.09960 | -0.00048 | 0.14402 | -0.01694 | 0.19750 | -0.03200 | -0.15497 | 0.03861 |
| VC_B_AB_nCi_2_MP5_X_KA_h-v_MAS | 0.0677 | 0.7182 | 0.02198 | 0.0670 | -0.36350 | -0.13564 | 0.18759 | 0.02382 | 0.20484 | -0.10887 | -0.06355 | -0.17943 |
| VC_B_AB_nCi_2_MP6_X_KA_h-v_MAS | 0.1302 | 0.8061 | 0.06269 | 0.0783 | -0.16215 | 0.04424 | 0.12564 | 0.00112 | 0.26333 | -0.06405 | -0.11754 | 0.02487 |
| PN_Q_AB_nCi_2_SS0_T_KA_a_MAS | 0.0271 | 0.3060 | 0.69379 | 0.0584 | -0.13465 | 0.17877 | 0.03532 | 0.00772 | 0.54294 | 0.01936 | 0.02700 | 0.05852 |
| PN_Q_AB_nCi_2_SS1_T_KA_a_MAS | 0.0323 | 0.3423 | 0.21885 | 0.0326 | -0.04874 | 0.20915 | 0.18729 | 0.03271 | 0.70540 | -0.07470 | 0.05932 | 0.09937 |
| PN_Q_AB_nCi_2_SS2_T_KA_a_MAS | 0.0481 | 0.3798 | 0.53944 | 0.0398 | -0.05209 | 0.25611 | 0.11037 | 0.02030 | 0.62560 | -0.04612 | 0.06230 | 0.06138 |
| PN_Q_AB_nCi_2_SS3_T_KA_a_MAS | 0.0551 | 0.4056 | 0.24870 | 0.0313 | 0.02084 | 0.21913 | 0.19373 | 0.04357 | 0.70882 | -0.04040 | 0.05537 | 0.11865 |
| PN_Q_AB_nCi_2_SS4_T_KA_a_MAS | 0.0549 | 0.4262 | 0.41943 | 0.0240 | -0.00675 | 0.22304 | 0.14020 | 0.04341 | 0.67351 | -0.02852 | 0.04582 | 0.08726 |
| PN_Q_AB_nCi_2_SS5_T_KA_a_MAS | 0.0673 | 0.4176 | 0.25242 | 0.0282 | 0.06181 | 0.22005 | 0.19693 | 0.05307 | 0.69613 | -0.01241 | 0.05734 | 0.12704 |
| PN_Q_AB_nCi_2_SS6_T_KA_a_MAS | 0.0542 | 0.4328 | 0.37028 | 0.0139 | 0.02956 | 0.19610 | 0.15829 | 0.06051 | 0.68069 | -0.00171 | 0.04041 | 0.10032 |
| P2_Q_AB_nCi_2_SS0_T_KA_a_MAS | 0.0776 | 0.1792 | 0.81577 | 0.2283 | -0.08008 | 0.24053 | 0.03110 | -0.01902 | 0.28648 | 0.02694 | 0.01077 | -0.03140 |
| P2_Q_AB_nCi_2_SS1_T_KA_a_MAS | 0.0215 | 0.2615 | 0.27295 | 0.0654 | -0.23784 | 0.24632 | 0.08332 | -0.00913 | 0.63615 | -0.14514 | -0.00609 | 0.05427 |
| P2_Q_AB_nCi_2_SS2_T_KA_a_MAS | 0.0789 | 0.3011 | 0.66668 | 0.1505 | -0.07955 | 0.36583 | 0.05078 | -0.01532 | 0.44100 | -0.09482 | 0.05640 | 0.00274 |
| P2_Q_AB_nCi_2_SS3_T_KA_a_MAS | 0.0481 | 0.3516 | 0.30036 | 0.0596 | -0.13731 | 0.26505 | 0.10663 | 0.00915 | 0.68720 | -0.12634 | 0.01402 | 0.08323 |
| P2_Q_AB_nCi_2_SS4_T_KA_a_MAS | 0.0785 | 0.3978 | 0.50248 | 0.0881 | -0.07160 | 0.30086 | 0.06738 | 0.01784 | 0.58736 | -0.08217 | 0.02998 | 0.05980 |
| P2_Q_AB_nCi_2_SS5_T_KA_a_MAS | 0.0596 | 0.3786 | 0.30947 | 0.0513 | -0.07551 | 0.26179 | 0.12081 | 0.02796 | 0.69793 | -0.09101 | 0.03080 | 0.10344 |
| P2_Q_AB_nCi_2_SS6_T_KA_a_MAS | 0.0710 | 0.4126 | 0.44221 | 0.0597 | -0.04189 | 0.24906 | 0.08875 | 0.04205 | 0.63619 | -0.05100 | 0.02551 | 0.08340 |
| P3_Q_AB_nCi_2_SS0_T_KA_a_MAS | 0.1090 | 0.1215 | 0.80187 | 0.3358 | -0.05146 | 0.26071 | 0.02934 | -0.02337 | 0.15787 | 0.03835 | 0.00351 | -0.06446 |
| P3_Q_AB_nCi_2_SS1_T_KA_a_MAS | 0.0718 | 0.2731 | 0.25390 | 0.1102 | -0.01327 | 0.23379 | 0.15900 | 0.02415 | 0.47221 | -0.12353 | 0.10586 | 0.13229 |
| P3_Q_AB_nCi_2_SS2_T_KA_a_MAS | 0.1064 | 0.2562 | 0.68423 | 0.2214 | -0.07392 | 0.41289 | 0.02788 | -0.02406 | 0.32156 | -0.10609 | 0.05153 | -0.03114 |
| P3_Q_AB_nCi_2_SS3_T_KA_a_MAS | 0.0928 | 0.3484 | 0.29154 | 0.1124 | 0.01787 | 0.26598 | 0.15222 | 0.02826 | 0.55668 | -0.12710 | 0.08641 | 0.14402 |
| P3_Q_AB_nCi_2_SS4_T_KA_a_MAS | 0.1055 | 0.3798 | 0.51817 | 0.1315 | -0.08215 | 0.34299 | 0.04021 | 0.00826 | 0.51365 | -0.10597 | 0.02170 | 0.03147 |
| P3_Q_AB_nCi_2_SS5_T_KA_a_MAS | 0.1095 | 0.3764 | 0.28960 | 0.1030 | 0.05682 | 0.27694 | 0.15597 | 0.03639 | 0.58603 | -0.09848 | 0.07158 | 0.14725 |
| P3_Q_AB_nCi_2_SS6_T_KA_a_MAS | 0.0973 | 0.4009 | 0.45496 | 0.0938 | -0.05321 | 0.28359 | 0.06594 | 0.03362 | 0.58419 | -0.07681 | 0.01610 | 0.05561 |
| S_Q_AB_nCi_2_SS0_T_KA_a_MAS | 0.1054 | -0.2223 | -0.18648 | 0.4309 | 0.08427 | 0.07587 | -0.00745 | 0.03244 | -0.49983 | 0.04425 | -0.02529 | -0.17038 |
| S_Q_AB_nCi_2_SS1_T_KA_a_MAS | 0.1553 | -0.0305 | 0.15374 | 0.1489 | 0.11648 | 0.29223 | 0.05923 | 0.00191 | -0.27088 | -0.08191 | 0.05802 | -0.05694 |
| S_Q_AB_nCi_2_SS2_T_KA_a_MAS | 0.1073 | -0.2392 | -0.04443 | 0.3778 | 0.04396 | 0.15853 | -0.06210 | 0.01881 | -0.56770 | -0.00164 | -0.04611 | -0.20763 |
| S_Q_AB_nCi_2_SS3_T_KA_a_MAS | 0.1321 | -0.0210 | 0.16211 | 0.2028 | 0.10835 | 0.33164 | 0.00333 | 0.01881 | -0.29641 | -0.12636 | 0.01499 | -0.10782 |
| S_Q_AB_nCi_2_SS4_T_KA_a_MAS | 0.1186 | -0.2031 | 0.03732 | 0.3443 | 0.02405 | 0.19970 | -0.08274 | 0.00286 | -0.52551 | -0.06988 | -0.05651 | -0.24413 |
| S_Q_AB_nCi_2_SS5_T_KA_a_MAS | 0.1246 | -0.0341 | 0.15287 | 0.2327 | 0.13710 | 0.34101 | -0.00316 | 0.02690 | -0.31571 | -0.14474 | 0.00171 | -0.13627 |
| SD_Q_AB_nCi_2_SS0_T_KA_a_MAS | 0.0877 | 0.0576 | 0.81603 | 0.3182 | -0.03435 | 0.25810 | 0.01478 | -0.03616 | 0.07054 | 0.03587 | -0.01185 | -0.10234 |
| SD_Q_AB_nCi_2_SS1_T_KA_a_MAS | 0.0042 | 0.1410 | 0.28369 | 0.0887 | -0.34888 | 0.25465 | -0.00896 | -0.04428 | 0.46326 | -0.19061 | -0.05434 | 0.00066 |
| SD_Q_AB_nCi_2_SS2_T_KA_a_MAS | 0.0839 | 0.1641 | 0.71132 | 0.2347 | -0.10176 | 0.42463 | -0.02367 | -0.04809 | 0.20097 | -0.13234 | 0.03666 | -0.06431 |
| SD_Q_AB_nCi_2_SS3_T_KA_a_MAS | 0.0251 | 0.2164 | 0.32080 | 0.0935 | -0.28543 | 0.29644 | -0.00468 | -0.03031 | 0.54117 | -0.22040 | -0.03232 | 0.03212 |
| SD_Q_AB_nCi_2_SS4_T_KA_a_MAS | 0.0862 | 0.2966 | 0.55579 | 0.1570 | -0.13987 | 0.35946 | -0.03203 | -0.01500 | 0.41226 | -0.14505 | 0.00219 | 0.00986 |
| SD_Q_AB_nCi_2_SS5_T_KA_a_MAS | 0.0367 | 0.2496 | 0.34897 | 0.0878 | -0.23312 | 0.30455 | 0.00531 | -0.00886 | 0.58115 | -0.20088 | -0.01112 | 0.06019 |
| SD_Q_AB_nCi_2_SS6_T_KA_a_MAS | 0.0779 | 0.3196 | 0.50295 | 0.1200 | -0.12997 | 0.29436 | -0.01836 | 0.01259 | 0.50262 | -0.12242 | -0.00447 | 0.04129 |
| VC_Q_AB_nCi_2_SS0_T_KA_a_MAS | 0.0312 | -0.3034 | 0.03880 | 0.4053 | 0.15124 | 0.06358 | 0.03018 | -0.02101 | -0.64889 | -0.00511 | -0.04303 | -0.22855 |
| VC_Q_AB_nCi_2_SS2_T_KA_a_MAS | -0.0318 | -0.3288 | 0.08857 | 0.2973 | -0.05551 | 0.09263 | -0.15577 | -0.04988 | -0.64771 | -0.04016 | -0.04432 | -0.14485 |
| S_Q_AB_nCi_2_SS0_T_KA_h_MAS | 0.3201 | 0.2789 | -0.00902 | 0.1709 | 0.04989 | 0.04529 | -0.05908 | 0.00022 | 0.06589 | -0.01950 | -0.14446 | -0.00132 |
| S_Q_AB_nCi_2_SS1_T_KA_h_MAS | 0.2417 | 0.2056 | 0.07874 | 0.1048 | 0.08913 | -0.04783 | -0.04953 | 0.01106 | 0.07573 | -0.10470 | -0.39749 | -0.19966 |
| S_Q_AB_nCi_2_SS2_T_KA_h_MAS | 0.1744 | 0.1366 | 0.00642 | 0.1606 | 0.12504 | 0.01953 | -0.08081 | -0.02780 | 0.11394 | 0.01970 | -0.35403 | -0.15882 |
| S_Q_AB_nCi_2_SS3_T_KA_h_MAS | 0.1248 | 0.1489 | 0.07375 | 0.0901 | -0.00875 | -0.05296 | -0.05045 | 0.01445 | 0.17653 | -0.05371 | -0.45867 | -0.20337 |
| S_Q_AB_nCi_2_SS4_T_KA_h_MAS | 0.1124 | 0.1137 | -0.02402 | 0.1270 | 0.05139 | -0.06850 | -0.05923 | -0.02611 | 0.18171 | 0.03564 | -0.46462 | -0.15907 |
| S_Q_AB_nCi_2_SS5_T_KA_h_MAS | 0.1676 | 0.1430 | 0.07672 | 0.0922 | -0.04331 | -0.11835 | -0.01913 | 0.02859 | 0.24629 | -0.04650 | -0.43464 | -0.15013 |
| S_Q_AB_nCi_2_SS6_T_KA_h_MAS | 0.1316 | 0.0956 | -0.01693 | 0.1135 | 0.02401 | -0.16675 | -0.03377 | -0.01521 | 0.21968 | 0.03575 | -0.47833 | -0.15325 |
| VC_Q_AB_nCi_2_SS0_T_KA_h_MAS | 0.2115 | 0.0089 | 0.18781 | 0.0998 | 0.08096 | -0.11015 | -0.01614 | -0.02258 | 0.21107 | 0.26090 | -0.15814 | -0.29208 |
| VC_Q_AB_nCi_2_SS1_T_KA_h_MAS | 0.3344 | 0.1019 | 0.31788 | 0.0754 | -0.13642 | -0.20398 | -0.01310 | 0.03526 | 0.25701 | 0.17792 | -0.20683 | -0.35348 |
| VC_Q_AB_nCi_2_SS2_T_KA_h_MAS | 0.2927 | 0.1346 | 0.17369 | 0.1103 | -0.05522 | -0.21977 | -0.02529 | -0.00491 | 0.28166 | 0.17009 | -0.37403 | -0.26835 |
| VC_Q_AB_nCi_2_SS3_T_KA_h_MAS | 0.3781 | 0.1784 | 0.22752 | 0.0878 | -0.19520 | -0.25992 | 0.00844 | 0.07026 | 0.33795 | 0.11255 | -0.30108 | -0.23130 |
| VC_Q_AB_nCi_2_SS4_T_KA_h_MAS | 0.2862 | 0.1533 | 0.13595 | 0.0871 | -0.11026 | -0.29680 | -0.02178 | 0.04550 | 0.35648 | 0.15204 | -0.44094 | -0.21428 |
| VC_Q_AB_nCi_2_SS5_T_KA_h_MAS | 0.3450 | 0.1709 | 0.22471 | 0.0645 | -0.18883 | -0.26762 | 0.00049 | 0.11583 | 0.40978 | 0.11169 | -0.30974 | -0.19578 |
| VC_Q_AB_nCi_2_SS6_T_KA_h_MAS | 0.2643 | 0.1475 | 0.14096 | 0.0664 | -0.12309 | -0.30513 | -0.01865 | 0.07618 | 0.41649 | 0.14196 | -0.43748 | -0.19647 |
| PN_Q_AB_nCi_2_SS0_T_KA_v_MAS | 0.0343 | 0.7337 | 0.20639 | 0.0487 | 0.03162 | -0.10615 | -0.15729 | -0.09865 | -0.09301 | 0.00719 | 0.22477 | 0.45265 |
| PN_Q_AB_nCi_2_SS1_T_KA_v_MAS | 0.0111 | 0.7651 | 0.08905 | 0.0117 | 0.06655 | -0.22676 | -0.13550 | -0.07322 | -0.06000 | 0.01621 | 0.20593 | 0.41274 |
| PN_Q_AB_nCi_2_SS2_T_KA_v_MAS | 0.0287 | 0.7438 | 0.18135 | 0.0345 | 0.08762 | -0.07868 | -0.14827 | -0.10239 | -0.13173 | 0.00833 | 0.24457 | 0.44519 |
| PN_Q_AB_nCi_2_SS3_T_KA_v_MAS | 0.0120 | 0.7657 | 0.12809 | 0.0263 | 0.08955 | -0.16248 | -0.13947 | -0.09360 | -0.11945 | 0.00179 | 0.22754 | 0.41264 |
| PN_Q_AB_nCi_2_SS4_T_KA_v_MAS | 0.0264 | 0.7493 | 0.16432 | 0.0301 | 0.10865 | -0.06952 | -0.15195 | -0.10331 | -0.14527 | 0.01285 | 0.24883 | 0.43172 |
| PN_Q_AB_nCi_2_SS5_T_KA_v_MAS | 0.0132 | 0.7615 | 0.13614 | 0.0307 | 0.10764 | -0.12763 | -0.14492 | -0.09879 | -0.14610 | -0.00062 | 0.23658 | 0.40475 |
| PN_Q_AB_nCi_2_SS6_T_KA_v_MAS | 0.0267 | 0.7503 | 0.15540 | 0.0292 | 0.11962 | -0.06191 | -0.15655 | -0.10406 | -0.15706 | 0.01520 | 0.24798 | 0.42256 |
| P2_Q_AB_nCi_2_SS1_T_KA_v_MAS | 0.0082 | 0.7261 | 0.20145 | -0.0249 | 0.05792 | -0.32340 | -0.14880 | -0.04348 | -0.03301 | 0.08170 | 0.20017 | 0.29876 |
| P2_Q_AB_nCi_2_SS2_T_KA_v_MAS | 0.0381 | 0.7197 | 0.20609 | 0.0292 | 0.08876 | -0.12802 | -0.15772 | -0.10546 | -0.17338 | 0.02231 | 0.27046 | 0.36680 |
| P2_Q_AB_nCi_2_SS3_T_KA_v_MAS | 0.0061 | 0.7484 | 0.20525 | 0.0096 | 0.11049 | -0.21674 | -0.14414 | -0.08109 | -0.12894 | 0.02255 | 0.25255 | 0.30184 |
| P2_Q_AB_nCi_2_SS4_T_KA_v_MAS | 0.0281 | 0.7222 | 0.20698 | 0.0230 | 0.10565 | -0.10117 | -0.16830 | -0.11020 | -0.18684 | 0.02480 | 0.28640 | 0.33095 |
| P2_Q_AB_nCi_2_SS5_T_KA_v_MAS | 0.0027 | 0.7408 | 0.20842 | 0.0195 | 0.12180 | -0.15719 | -0.15428 | -0.09234 | -0.16051 | 0.00364 | 0.27314 | 0.28085 |
| P2_Q_AB_nCi_2_SS6_T_KA_v_MAS | 0.0275 | 0.7231 | 0.20550 | 0.0241 | 0.10868 | -0.07553 | -0.17647 | -0.11538 | -0.18917 | 0.01463 | 0.29058 | 0.30702 |
| P3_Q_AB_nCi_2_SS1_T_KA_v_MAS | 0.0068 | 0.6418 | 0.32569 | -0.0580 | 0.02690 | -0.38219 | -0.15036 | -0.01079 | 0.01110 | 0.14356 | 0.17443 | 0.18361 |
| P3_Q_AB_nCi_2_SS2_T_KA_v_MAS | 0.0361 | 0.6809 | 0.24091 | 0.0213 | 0.06126 | -0.17093 | -0.16736 | -0.10205 | -0.19403 | 0.04141 | 0.29389 | 0.28313 |
| P3_Q_AB_nCi_2_SS3_T_KA_v_MAS | -0.0090 | 0.7082 | 0.30097 | -0.0079 | 0.09482 | -0.26170 | -0.14059 | -0.06051 | -0.10978 | 0.04678 | 0.26650 | 0.18638 |
| P3_Q_AB_nCi_2_SS4_T_KA_v_MAS | 0.0168 | 0.6713 | 0.26463 | 0.0135 | 0.05923 | -0.12837 | -0.18153 | -0.11040 | -0.19706 | 0.03747 | 0.32054 | 0.21760 |
| P3_Q_AB_nCi_2_SS5_T_KA_v_MAS | -0.0189 | 0.6970 | 0.29281 | 0.0074 | 0.08929 | -0.18318 | -0.15451 | -0.07796 | -0.13789 | 0.00796 | 0.29871 | 0.15259 |
| P3_Q_AB_nCi_2_SS6_T_KA_v_MAS | 0.0161 | 0.6686 | 0.26668 | 0.0168 | 0.04732 | -0.08687 | -0.18893 | -0.12003 | -0.18011 | 0.01092 | 0.32649 | 0.17885 |
| HM_Q_AB_nCi_2_SS0_T_KA_v_MAS | -0.0754 | 0.7279 | 0.15049 | 0.0126 | -0.05972 | 0.02352 | -0.13802 | -0.06833 | -0.01775 | 0.00781 | 0.18064 | 0.54233 |
| HM_Q_AB_nCi_2_SS1_T_KA_v_MAS | -0.0112 | 0.7307 | 0.03044 | 0.0513 | -0.00221 | -0.04275 | -0.10576 | -0.08437 | -0.02820 | -0.04669 | 0.19221 | 0.55114 |
| HM_Q_AB_nCi_2_SS2_T_KA_v_MAS | -0.0320 | 0.7272 | 0.16007 | 0.0281 | 0.01138 | 0.02214 | -0.13669 | -0.06826 | -0.00459 | 0.00554 | 0.21289 | 0.54426 |
| HM_Q_AB_nCi_2_SS3_T_KA_v_MAS | -0.0185 | 0.7354 | 0.09205 | 0.0453 | -0.03500 | -0.05875 | -0.11650 | -0.08578 | -0.03489 | -0.01216 | 0.18337 | 0.55758 |
| HM_Q_AB_nCi_2_SS4_T_KA_v_MAS | -0.0242 | 0.7317 | 0.15169 | 0.0314 | 0.01174 | -0.00189 | -0.12796 | -0.07043 | -0.00968 | 0.00350 | 0.20907 | 0.55260 |
| HM_Q_AB_nCi_2_SS5_T_KA_v_MAS | -0.0162 | 0.7361 | 0.10131 | 0.0426 | -0.03282 | -0.07443 | -0.11358 | -0.08415 | -0.03385 | 0.00076 | 0.17812 | 0.56279 |
| HM_Q_AB_nCi_2_SS6_T_KA_v_MAS | -0.0211 | 0.7329 | 0.13985 | 0.0294 | 0.01466 | -0.02774 | -0.12162 | -0.07008 | -0.01769 | 0.01304 | 0.20322 | 0.56084 |
| S_Q_AB_nCi_2_SS0_T_KA_v_MAS | -0.0443 | -0.8282 | -0.11413 | -0.0474 | -0.22085 | -0.07532 | -0.04308 | 0.04000 | 0.00173 | -0.00986 | -0.00476 | -0.11623 |
| S_Q_AB_nCi_2_SS1_T_KA_v_MAS | 0.0369 | -0.1304 | 0.55935 | -0.0712 | -0.21841 | -0.17017 | -0.11292 | 0.09435 | 0.24259 | 0.24160 | -0.07919 | -0.14127 |
| S_Q_AB_nCi_2_SS2_T_KA_v_MAS | -0.0553 | -0.5324 | 0.21138 | -0.0837 | -0.34271 | -0.09254 | -0.07162 | 0.09565 | 0.17817 | 0.14952 | -0.01043 | -0.28300 |
| S_Q_AB_nCi_2_SS3_T_KA_v_MAS | -0.0841 | -0.2245 | 0.51916 | -0.0530 | -0.32910 | -0.06840 | 0.00910 | 0.11273 | 0.31820 | 0.16809 | -0.03559 | -0.24289 |
| S_Q_AB_nCi_2_SS4_T_KA_v_MAS | -0.0803 | -0.3366 | 0.32046 | -0.0490 | -0.43456 | 0.01428 | -0.07129 | 0.04966 | 0.27407 | 0.07707 | 0.03174 | -0.25801 |
| S_Q_AB_nCi_2_SS5_T_KA_v_MAS | -0.1088 | -0.1531 | 0.42881 | -0.0329 | -0.41557 | -0.00457 | 0.01300 | 0.07031 | 0.39414 | 0.06888 | -0.00150 | -0.21415 |
| S_Q_AB_nCi_2_SS6_T_KA_v_MAS | -0.0549 | -0.2145 | 0.30161 | -0.0183 | -0.46639 | 0.05213 | -0.04101 | 0.01133 | 0.36430 | -0.00505 | 0.03173 | -0.23494 |
| SD_Q_AB_nCi_2_SS1_T_KA_v_MAS | -0.0395 | -0.1459 | 0.43477 | -0.1350 | -0.01211 | -0.38955 | -0.05550 | 0.10367 | 0.08144 | 0.26267 | -0.05554 | -0.42330 |
| SD_Q_AB_nCi_2_SS2_T_KA_v_MAS | 0.0254 | -0.5581 | 0.01356 | -0.0344 | -0.01215 | -0.17841 | 0.06462 | 0.02122 | -0.14520 | 0.08430 | -0.09064 | -0.59253 |
| SD_Q_AB_nCi_2_SS3_T_KA_v_MAS | -0.0579 | -0.2756 | 0.35914 | -0.0844 | 0.10353 | -0.24753 | 0.01676 | 0.07090 | -0.04299 | 0.12693 | 0.01524 | -0.63484 |
| SD_Q_AB_nCi_2_SS4_T_KA_v_MAS | -0.0073 | -0.4634 | 0.11900 | -0.0367 | -0.01772 | -0.11463 | 0.00331 | -0.00777 | -0.16522 | 0.07895 | -0.00021 | -0.63691 |
| SD_Q_AB_nCi_2_SS5_T_KA_v_MAS | -0.0788 | -0.2294 | 0.34181 | -0.0541 | 0.08326 | -0.12876 | -0.01800 | 0.03438 | -0.07452 | 0.04929 | 0.08590 | -0.66550 |
| SD_Q_AB_nCi_2_SS6_T_KA_v_MAS | -0.0105 | -0.3591 | 0.17526 | -0.0192 | -0.03869 | -0.03174 | -0.03814 | -0.04083 | -0.14165 | 0.02016 | 0.05809 | -0.64729 |
| VC_Q_AB_nCi_2_SS0_T_KA_v_MAS | -0.0367 | -0.6934 | -0.15265 | -0.0293 | -0.05304 | -0.03330 | 0.10176 | 0.08584 | 0.02113 | -0.01029 | -0.12894 | -0.55025 |
| VC_Q_AB_nCi_2_SS1_T_KA_v_MAS | -0.0662 | -0.4367 | 0.32432 | -0.1268 | -0.04839 | -0.24335 | 0.00390 | 0.13177 | 0.11142 | 0.21406 | -0.11970 | -0.55903 |
| VC_Q_AB_nCi_2_SS2_T_KA_v_MAS | -0.0324 | -0.6718 | -0.07337 | -0.0541 | -0.05463 | -0.09994 | 0.09434 | 0.06997 | -0.02777 | 0.05510 | -0.13925 | -0.62213 |
| VC_Q_AB_nCi_2_SS3_T_KA_v_MAS | -0.0808 | -0.5364 | 0.19974 | -0.0854 | 0.03246 | -0.11609 | 0.06375 | 0.10725 | 0.03684 | 0.09343 | -0.06729 | -0.68964 |
| VC_Q_AB_nCi_2_SS4_T_KA_v_MAS | -0.0561 | -0.6317 | 0.00471 | -0.0541 | -0.07077 | -0.05636 | 0.05848 | 0.05222 | -0.03469 | 0.05011 | -0.08558 | -0.66539 |
| VC_Q_AB_nCi_2_SS5_T_KA_v_MAS | -0.0986 | -0.5198 | 0.18057 | -0.0666 | 0.00698 | -0.03816 | 0.04429 | 0.08436 | 0.02820 | 0.03679 | -0.02465 | -0.72206 |
| VC_Q_AB_nCi_2_SS6_T_KA_v_MAS | -0.0597 | -0.5857 | 0.04680 | -0.0427 | -0.09514 | 0.00002 | 0.03610 | 0.03124 | -0.00997 | 0.00725 | -0.05157 | -0.68840 |
| S_Q_AB_nCi_2_MP0_T_KA_a_MAS | 0.1054 | -0.2223 | -0.18648 | 0.4309 | 0.08427 | 0.07587 | -0.00745 | 0.03244 | -0.49983 | 0.04425 | -0.02529 | -0.17038 |
| S_Q_AB_nCi_2_MP1_T_KA_a_MAS | 0.1014 | -0.0577 | 0.11660 | 0.0933 | 0.07543 | 0.29526 | 0.02611 | -0.00634 | -0.26024 | -0.07630 | 0.04608 | -0.04520 |
| S_Q_AB_nCi_2_MP2_T_KA_a_MAS | 0.0676 | -0.2859 | -0.02433 | 0.1692 | -0.09850 | 0.26303 | -0.15455 | -0.05413 | -0.52082 | -0.07696 | -0.06553 | -0.21118 |
| VC_Q_AB_nCi_2_MP0_T_KA_a_MAS | 0.0312 | -0.3034 | 0.03880 | 0.4053 | 0.15124 | 0.06358 | 0.03018 | -0.02101 | -0.64889 | -0.00511 | -0.04303 | -0.22855 |
| VC_Q_AB_nCi_2_MP2_T_KA_a_MAS | -0.0647 | -0.3977 | 0.01088 | 0.0572 | -0.17704 | 0.06511 | -0.24067 | -0.05735 | -0.50888 | -0.03039 | -0.12535 | -0.18470 |
| VC_Q_AB_nCi_2_MP4_T_KA_a_MAS | -0.1398 | -0.3908 | -0.06073 | -0.0711 | -0.34141 | -0.10123 | -0.28774 | -0.01622 | -0.32344 | 0.08229 | -0.13289 | -0.07391 |
| S_Q_AB_nCi_2_MP0_T_KA_h_MAS | 0.3201 | 0.2789 | -0.00902 | 0.1709 | 0.04989 | 0.04529 | -0.05908 | 0.00022 | 0.06589 | -0.01950 | -0.14446 | -0.00132 |
| S_Q_AB_nCi_2_MP1_T_KA_h_MAS | 0.2434 | 0.1030 | 0.08051 | 0.0784 | 0.00456 | -0.18556 | 0.02823 | 0.04715 | 0.39569 | 0.02951 | -0.43206 | -0.23135 |
| S_Q_AB_nCi_2_MP2_T_KA_h_MAS | 0.1250 | 0.0496 | 0.02909 | 0.0496 | -0.00714 | -0.26221 | 0.00421 | 0.03842 | 0.38558 | 0.12092 | -0.48305 | -0.24023 |
| S_Q_AB_nCi_2_MP3_T_KA_h_MAS | 0.2287 | 0.1105 | 0.18761 | 0.0612 | -0.14834 | -0.13629 | 0.01246 | 0.11537 | 0.71385 | 0.06677 | -0.19175 | -0.22868 |
| S_Q_AB_nCi_2_MP4_T_KA_h_MAS | 0.1798 | 0.1246 | 0.12964 | 0.0090 | -0.10803 | -0.21715 | 0.02027 | 0.10497 | 0.66824 | 0.15761 | -0.23856 | -0.22733 |
| S_Q_AB_nCi_2_MP5_T_KA_h_MAS | 0.2326 | 0.1251 | 0.19804 | 0.0396 | -0.14915 | -0.10748 | 0.01392 | 0.13756 | 0.73405 | 0.10773 | -0.10938 | -0.20234 |
| S_Q_AB_nCi_2_MP6_T_KA_h_MAS | 0.2146 | 0.1256 | 0.13466 | 0.0104 | -0.12934 | -0.19215 | 0.03926 | 0.12620 | 0.69198 | 0.16285 | -0.14470 | -0.20071 |
| VC_Q_AB_nCi_2_MP0_T_KA_h_MAS | 0.2115 | 0.0089 | 0.18781 | 0.0998 | 0.08096 | -0.11015 | -0.01614 | -0.02258 | 0.21107 | 0.26090 | -0.15814 | -0.29208 |
| VC_Q_AB_nCi_2_MP1_T_KA_h_MAS | 0.2414 | 0.0514 | 0.21655 | -0.0205 | -0.14349 | -0.38591 | -0.05535 | 0.10736 | 0.37932 | 0.32543 | -0.21742 | -0.31888 |
| VC_Q_AB_nCi_2_MP2_T_KA_h_MAS | 0.1989 | 0.0745 | 0.14205 | 0.0760 | -0.08085 | -0.25854 | -0.05338 | 0.03842 | 0.43244 | 0.20807 | -0.33360 | -0.27378 |
| VC_Q_AB_nCi_2_MP3_T_KA_h_MAS | 0.2397 | 0.1072 | 0.28672 | 0.1251 | -0.13809 | -0.02341 | -0.07676 | 0.03476 | 0.46511 | 0.01569 | -0.13053 | -0.32439 |
| VC_Q_AB_nCi_2_MP4_T_KA_h_MAS | 0.2029 | 0.1261 | 0.19274 | 0.1484 | -0.11590 | 0.02135 | -0.08118 | 0.01957 | 0.52652 | -0.04508 | -0.12704 | -0.28344 |
| VC_Q_AB_nCi_2_MP5_T_KA_h_MAS | 0.2499 | 0.1483 | 0.22218 | 0.1700 | -0.12307 | 0.12820 | -0.08550 | 0.01570 | 0.49480 | -0.15797 | -0.05071 | -0.27515 |
| VC_Q_AB_nCi_2_MP6_T_KA_h_MAS | 0.2373 | 0.1545 | 0.16126 | 0.1722 | -0.11800 | 0.10183 | -0.07532 | 0.02666 | 0.53215 | -0.15132 | -0.05629 | -0.25795 |
| PN_Q_AB_nCi_2_MP0_T_KA_v_MAS | -0.9450 | 0.0016 | 0.04699 | -0.1087 | -0.06839 | -0.00848 | -0.06284 | 0.02397 | 0.00210 | 0.07577 | 0.02907 | 0.15162 |
| PN_Q_AB_nCi_2_MP1_T_KA_v_MAS | -0.9553 | -0.0163 | 0.00140 | -0.1207 | -0.02839 | -0.01854 | -0.05544 | 0.02856 | -0.01285 | 0.07615 | 0.03023 | 0.10668 |
| PN_Q_AB_nCi_2_MP2_T_KA_v_MAS | -0.9511 | -0.0075 | 0.01290 | -0.1186 | -0.01352 | 0.01017 | -0.06427 | 0.02310 | -0.02920 | 0.07882 | 0.03777 | 0.12846 |
| PN_Q_AB_nCi_2_MP3_T_KA_v_MAS | -0.9543 | -0.0119 | 0.00452 | -0.1182 | -0.01857 | -0.00294 | -0.06196 | 0.02492 | -0.03219 | 0.07594 | 0.03273 | 0.10737 |
| PN_Q_AB_nCi_2_MP4_T_KA_v_MAS | -0.9496 | -0.0065 | 0.00745 | -0.1180 | -0.00459 | 0.01481 | -0.06924 | 0.02119 | -0.04045 | 0.07959 | 0.03610 | 0.12524 |
| PN_Q_AB_nCi_2_MP5_T_KA_v_MAS | -0.9530 | -0.0103 | 0.00425 | -0.1171 | -0.01484 | 0.00306 | -0.06598 | 0.02349 | -0.03900 | 0.07571 | 0.03206 | 0.10747 |
| PN_Q_AB_nCi_2_MP6_T_KA_v_MAS | -0.9484 | -0.0061 | 0.00548 | -0.1172 | -0.00169 | 0.01657 | -0.07243 | 0.02009 | -0.04545 | 0.07945 | 0.03389 | 0.12410 |
| P2_Q_AB_nCi_2_MP0_T_KA_v_MAS | -0.9510 | -0.0414 | 0.03748 | -0.1123 | -0.07739 | -0.01481 | -0.05526 | 0.02748 | 0.00508 | 0.07666 | 0.01991 | 0.10449 |
| P2_Q_AB_nCi_2_MP1_T_KA_v_MAS | -0.9590 | -0.0461 | 0.02761 | -0.1235 | -0.03012 | -0.01953 | -0.05902 | 0.03065 | -0.01293 | 0.08656 | 0.03389 | 0.04838 |
| P2_Q_AB_nCi_2_MP2_T_KA_v_MAS | -0.9595 | -0.0440 | 0.02936 | -0.1148 | -0.02311 | 0.02173 | -0.06676 | 0.01512 | -0.02931 | 0.07028 | 0.04267 | 0.05576 |
| P2_Q_AB_nCi_2_MP3_T_KA_v_MAS | -0.9584 | -0.0327 | 0.03694 | -0.1084 | -0.02970 | 0.01822 | -0.06822 | 0.01272 | -0.02013 | 0.05378 | 0.04437 | 0.03095 |
| P2_Q_AB_nCi_2_MP4_T_KA_v_MAS | -0.9558 | -0.0284 | 0.03399 | -0.1039 | -0.02521 | 0.04261 | -0.07641 | 0.00149 | -0.01866 | 0.04265 | 0.04712 | 0.04131 |
| P2_Q_AB_nCi_2_MP5_T_KA_v_MAS | -0.9537 | -0.0188 | 0.03733 | -0.0980 | -0.03340 | 0.03470 | -0.07352 | 0.00204 | -0.00467 | 0.02732 | 0.04569 | 0.02301 |
| P2_Q_AB_nCi_2_MP6_T_KA_v_MAS | -0.9507 | -0.0160 | 0.03284 | -0.0956 | -0.02749 | 0.05153 | -0.08031 | -0.00677 | -0.00203 | 0.02176 | 0.04526 | 0.03412 |
| P3_Q_AB_nCi_2_MP0_T_KA_v_MAS | -0.9495 | -0.0678 | 0.03044 | -0.1151 | -0.08866 | -0.01788 | -0.05209 | 0.03103 | 0.00784 | 0.07771 | 0.01372 | 0.07653 |
| P3_Q_AB_nCi_2_MP1_T_KA_v_MAS | -0.9565 | -0.0629 | 0.05011 | -0.1237 | -0.04280 | -0.02174 | -0.05979 | 0.03279 | -0.00260 | 0.09265 | 0.03498 | 0.01184 |
| P3_Q_AB_nCi_2_MP2_T_KA_v_MAS | -0.9589 | -0.0639 | 0.04510 | -0.1116 | -0.04522 | 0.02548 | -0.06619 | 0.01035 | -0.01502 | 0.06334 | 0.04541 | 0.00950 |
| P3_Q_AB_nCi_2_MP3_T_KA_v_MAS | -0.9530 | -0.0428 | 0.05864 | -0.1018 | -0.05196 | 0.02326 | -0.06782 | 0.00698 | 0.00485 | 0.03975 | 0.04992 | -0.01582 |
| P3_Q_AB_nCi_2_MP4_T_KA_v_MAS | -0.9497 | -0.0398 | 0.05173 | -0.0962 | -0.05552 | 0.04921 | -0.07524 | -0.00874 | 0.01349 | 0.02204 | 0.05161 | -0.01089 |
| P3_Q_AB_nCi_2_MP5_T_KA_v_MAS | -0.9418 | -0.0226 | 0.05738 | -0.0877 | -0.06044 | 0.04001 | -0.07116 | -0.00742 | 0.03415 | 0.00297 | 0.05093 | -0.02795 |
| P3_Q_AB_nCi_2_MP6_T_KA_v_MAS | -0.9381 | -0.0219 | 0.04934 | -0.0851 | -0.06161 | 0.05721 | -0.07708 | -0.01941 | 0.04233 | -0.00544 | 0.04868 | -0.02159 |
| HM_Q_AB_nCi_2_MP0_T_KA_v_MAS | -0.8896 | 0.1112 | 0.06100 | -0.1043 | -0.08604 | 0.02300 | -0.08781 | 0.03339 | 0.01516 | 0.07568 | 0.05585 | 0.28645 |
| HM_Q_AB_nCi_2_MP1_T_KA_v_MAS | -0.8932 | 0.0553 | -0.00212 | -0.1008 | -0.10534 | -0.02990 | -0.03811 | 0.03341 | 0.07640 | 0.04679 | 0.01326 | 0.26486 |
| HM_Q_AB_nCi_2_MP2_T_KA_v_MAS | -0.8694 | 0.0729 | 0.04885 | -0.1115 | -0.09056 | -0.04459 | -0.04811 | 0.04658 | 0.07457 | 0.08647 | 0.03167 | 0.31630 |
| HM_Q_AB_nCi_2_MP3_T_KA_v_MAS | -0.8534 | 0.0195 | -0.01492 | -0.1359 | -0.08401 | -0.12362 | -0.02199 | 0.06570 | 0.08224 | 0.11658 | 0.00698 | 0.30583 |
| HM_Q_AB_nCi_2_MP6_T_KA_v_MAS | -0.7830 | -0.0285 | 0.00676 | -0.1722 | -0.03144 | -0.17664 | -0.00741 | 0.09984 | 0.03695 | 0.20233 | 0.01903 | 0.32095 |
| S_Q_AB_nCi_2_MP0_T_KA_v_MAS | -0.0425 | -0.8302 | -0.12214 | -0.0396 | -0.21681 | -0.06037 | -0.02652 | 0.04393 | -0.00272 | -0.02329 | -0.02002 | -0.14138 |
| S_Q_AB_nCi_2_MP1_T_KA_v_MAS | 0.0133 | -0.1593 | 0.41560 | 0.0141 | -0.36858 | -0.07841 | -0.03825 | 0.05294 | 0.51419 | 0.07488 | -0.02271 | -0.24377 |
| S_Q_AB_nCi_2_MP2_T_KA_v_MAS | 0.0870 | -0.1023 | 0.27983 | 0.0454 | -0.42401 | 0.01277 | 0.01941 | 0.02050 | 0.53842 | -0.08476 | -0.02784 | -0.26272 |
| S_Q_AB_nCi_2_MP3_T_KA_v_MAS | 0.1075 | -0.0509 | 0.22280 | 0.0498 | -0.36940 | -0.06558 | 0.05942 | 0.06024 | 0.61473 | -0.05428 | -0.04020 | -0.22686 |
| S_Q_AB_nCi_2_MP4_T_KA_v_MAS | 0.1672 | -0.0569 | 0.16789 | 0.0405 | -0.39050 | -0.05422 | 0.07868 | 0.05882 | 0.57114 | -0.05856 | -0.05625 | -0.25929 |
| S_Q_AB_nCi_2_MP5_T_KA_v_MAS | 0.1893 | -0.0222 | 0.16139 | 0.0473 | -0.35386 | -0.10515 | 0.09784 | 0.09515 | 0.59711 | -0.02121 | -0.04535 | -0.23204 |
| S_Q_AB_nCi_2_MP6_T_KA_v_MAS | 0.2270 | -0.0420 | 0.12113 | 0.0414 | -0.37260 | -0.09305 | 0.10372 | 0.09074 | 0.55094 | -0.02354 | -0.05719 | -0.25859 |
| SD_Q_AB_nCi_2_MP1_T_KA_v_MAS | -0.8786 | -0.1694 | 0.13496 | -0.1171 | -0.03396 | -0.01455 | -0.06580 | 0.04315 | -0.01461 | 0.12992 | 0.02310 | -0.21036 |
| SD_Q_AB_nCi_2_MP2_T_KA_v_MAS | -0.8657 | -0.1808 | 0.07577 | -0.0804 | -0.05444 | 0.07250 | -0.06583 | -0.01853 | -0.03505 | 0.04286 | 0.03121 | -0.25745 |
| SD_Q_AB_nCi_2_MP3_T_KA_v_MAS | -0.8619 | -0.0995 | 0.13167 | -0.0597 | -0.05998 | 0.08842 | -0.07872 | -0.02324 | 0.01427 | -0.00575 | 0.05586 | -0.24278 |
| SD_Q_AB_nCi_2_MP4_T_KA_v_MAS | -0.8355 | -0.0968 | 0.09714 | -0.0443 | -0.07871 | 0.12454 | -0.08446 | -0.05673 | 0.03189 | -0.04983 | 0.04654 | -0.24474 |
| SD_Q_AB_nCi_2_MP5_T_KA_v_MAS | -0.8214 | -0.0482 | 0.11800 | -0.0310 | -0.07703 | 0.11765 | -0.08095 | -0.05409 | 0.07161 | -0.07992 | 0.05362 | -0.24055 |
| SD_Q_AB_nCi_2_MP6_T_KA_v_MAS | -0.7984 | -0.0512 | 0.09144 | -0.0233 | -0.08784 | 0.13764 | -0.08453 | -0.07718 | 0.08512 | -0.10068 | 0.03977 | -0.24087 |
| VC_Q_AB_nCi_2_MP0_T_KA_v_MAS | -0.0367 | -0.6934 | -0.15265 | -0.0293 | -0.05304 | -0.03330 | 0.10176 | 0.08584 | 0.02113 | -0.01029 | -0.12894 | -0.55025 |
| VC_Q_AB_nCi_2_MP1_T_KA_v_MAS | -0.0329 | -0.4461 | 0.34790 | -0.0546 | 0.04213 | -0.00808 | -0.07841 | 0.06518 | -0.00399 | 0.16559 | 0.02288 | -0.66578 |
| VC_Q_AB_nCi_2_MP2_T_KA_v_MAS | 0.0519 | -0.4601 | 0.17659 | 0.0660 | -0.06543 | 0.16663 | -0.05985 | -0.01346 | 0.01735 | -0.10891 | 0.04302 | -0.67914 |
| VC_Q_AB_nCi_2_MP3_T_KA_v_MAS | 0.1110 | -0.2404 | 0.28738 | 0.1138 | -0.05841 | 0.23745 | -0.09107 | -0.02236 | 0.13325 | -0.20816 | 0.09100 | -0.60146 |
| VC_Q_AB_nCi_2_MP4_T_KA_v_MAS | 0.1594 | -0.2088 | 0.18502 | 0.1387 | -0.12292 | 0.23942 | -0.07130 | -0.02559 | 0.19147 | -0.28408 | 0.06911 | -0.53126 |
| VC_Q_AB_nCi_2_MP5_T_KA_v_MAS | 0.2039 | -0.0994 | 0.20198 | 0.1549 | -0.08948 | 0.23881 | -0.06772 | -0.00796 | 0.25226 | -0.30989 | 0.07481 | -0.47181 |
| VC_Q_AB_nCi_2_MP6_T_KA_v_MAS | 0.2333 | -0.0998 | 0.14556 | 0.1591 | -0.12609 | 0.22073 | -0.05227 | 0.00340 | 0.28154 | -0.32228 | 0.05405 | -0.44257 |
| S_Q_AB_nCi_2_SS0_A_KA_a_MAS | 0.3033 | 0.5412 | 0.07633 | 0.0609 | 0.12878 | -0.00141 | 0.17736 | -0.04272 | -0.16645 | 0.02163 | -0.07683 | -0.03865 |
| S_Q_AB_nCi_2_SS1_A_KA_a_MAS | 0.1397 | 0.0649 | 0.03360 | 0.0301 | 0.16955 | 0.03425 | 0.54038 | 0.02836 | -0.03362 | 0.04650 | 0.10682 | 0.05809 |
| S_Q_AB_nCi_2_SS3_A_KA_a_MAS | 0.1270 | 0.1305 | 0.05685 | 0.0181 | 0.12474 | -0.01363 | 0.56654 | 0.01915 | -0.06580 | 0.04476 | 0.10146 | 0.12087 |
| S_Q_AB_nCi_2_SS5_A_KA_a_MAS | 0.1346 | 0.1573 | 0.05575 | 0.0183 | 0.11196 | -0.03194 | 0.59229 | 0.01592 | -0.06833 | 0.03954 | 0.09493 | 0.12975 |
| VC_Q_AB_nCi_2_SS0_A_KA_a_MAS | 0.2736 | 0.5894 | 0.09329 | 0.0707 | 0.10216 | -0.01797 | 0.22205 | -0.06706 | -0.14085 | 0.01672 | -0.06753 | -0.00666 |
| S_Q_AB_nCi_2_SS1_A_KA_h_MAS | 0.2985 | 0.6624 | 0.01861 | 0.1260 | -0.01091 | 0.00565 | 0.02512 | 0.00186 | 0.00437 | -0.00646 | -0.13247 | -0.04881 |
| S_Q_AB_nCi_2_SS2_A_KA_h_MAS | 0.2265 | 0.8068 | 0.12902 | 0.0765 | 0.21115 | 0.01341 | 0.05138 | -0.00202 | 0.06541 | 0.11082 | -0.09317 | 0.03035 |
| S_Q_AB_nCi_2_SS3_A_KA_h_MAS | 0.2270 | 0.7982 | 0.05914 | 0.0582 | 0.14208 | 0.04268 | 0.03473 | 0.00336 | 0.02870 | -0.00990 | 0.07627 | 0.05098 |
| S_Q_AB_nCi_2_SS4_A_KA_h_MAS | 0.1933 | 0.8248 | 0.14220 | 0.0703 | 0.20058 | -0.03062 | 0.02863 | 0.01511 | 0.10811 | 0.09614 | -0.13840 | 0.01952 |
| S_Q_AB_nCi_2_SS5_A_KA_h_MAS | 0.1768 | 0.8082 | 0.08600 | 0.0354 | 0.16133 | 0.07784 | 0.03112 | 0.01891 | 0.03665 | 0.01821 | 0.11519 | 0.06731 |
| S_Q_AB_nCi_2_SS6_A_KA_h_MAS | 0.1772 | 0.8319 | 0.14120 | 0.0631 | 0.18974 | -0.05038 | 0.02716 | 0.01766 | 0.11170 | 0.09186 | -0.14791 | 0.01876 |
| SD_Q_AB_nCi_2_SS2_A_KA_h_MAS | -0.4513 | -0.1915 | -0.01211 | -0.0814 | 0.01446 | -0.01501 | 0.08721 | 0.03511 | 0.07831 | -0.01455 | -0.67986 | -0.09779 |
| VC_Q_AB_nCi_2_SS0_A_KA_h_MAS | 0.1169 | 0.9037 | 0.11913 | 0.0325 | 0.17354 | 0.03748 | 0.09641 | -0.01745 | 0.06661 | 0.07425 | 0.02030 | 0.07139 |
| VC_Q_AB_nCi_2_SS1_A_KA_h_MAS | 0.0983 | 0.9133 | 0.09008 | 0.0329 | 0.10274 | -0.09452 | 0.10386 | -0.00962 | 0.05864 | 0.05207 | -0.10004 | -0.02491 |
| VC_Q_AB_nCi_2_SS2_A_KA_h_MAS | 0.1185 | 0.8993 | 0.09692 | 0.0345 | 0.14747 | -0.02236 | 0.10567 | -0.01679 | 0.04646 | 0.07404 | -0.14259 | -0.00299 |
| VC_Q_AB_nCi_2_SS3_A_KA_h_MAS | 0.1279 | 0.9231 | 0.05819 | 0.0352 | 0.13526 | -0.00046 | 0.06913 | 0.00047 | 0.04285 | 0.01936 | -0.05378 | 0.01314 |
| VC_Q_AB_nCi_2_SS4_A_KA_h_MAS | 0.1301 | 0.8890 | 0.08945 | 0.0479 | 0.10456 | -0.07070 | 0.11642 | -0.02410 | 0.06298 | 0.07614 | -0.20890 | -0.00304 |
| VC_Q_AB_nCi_2_SS5_A_KA_h_MAS | 0.1258 | 0.9141 | 0.07316 | 0.0450 | 0.16449 | 0.02694 | 0.07233 | 0.00282 | 0.04919 | 0.00837 | -0.03024 | 0.04048 |
| VC_Q_AB_nCi_2_SS6_A_KA_h_MAS | 0.1351 | 0.8814 | 0.09745 | 0.0526 | 0.08802 | -0.09250 | 0.11920 | -0.01856 | 0.10116 | 0.06866 | -0.22379 | 0.00532 |
| PN_Q_AB_nCi_2_SS0_A_KA_v_MAS | -0.1986 | -0.5352 | -0.17471 | -0.1318 | -0.53586 | 0.07713 | -0.27376 | 0.04394 | 0.00920 | 0.07131 | -0.16219 | 0.03247 |
| PN_Q_AB_nCi_2_SS1_A_KA_v_MAS | -0.1679 | -0.6652 | -0.09070 | -0.0735 | -0.53021 | 0.25651 | -0.11335 | 0.04106 | 0.03375 | 0.02305 | -0.16930 | -0.07219 |
| PN_Q_AB_nCi_2_SS2_A_KA_v_MAS | -0.1833 | -0.6318 | -0.17298 | -0.1044 | -0.52778 | 0.11615 | -0.19907 | 0.05870 | 0.06747 | 0.04884 | -0.17148 | -0.02274 |
| PN_Q_AB_nCi_2_SS3_A_KA_v_MAS | -0.1718 | -0.6503 | -0.11200 | -0.0861 | -0.56555 | 0.17545 | -0.13211 | 0.04779 | 0.05905 | 0.04406 | -0.18753 | -0.04995 |
| PN_Q_AB_nCi_2_SS4_A_KA_v_MAS | -0.1754 | -0.6509 | -0.15768 | -0.0949 | -0.53561 | 0.11656 | -0.17092 | 0.05772 | 0.07598 | 0.04127 | -0.17695 | -0.02778 |
| PN_Q_AB_nCi_2_SS5_A_KA_v_MAS | -0.1710 | -0.6468 | -0.11774 | -0.0895 | -0.57471 | 0.14187 | -0.12701 | 0.05036 | 0.07988 | 0.04654 | -0.19576 | -0.04190 |
| PN_Q_AB_nCi_2_SS6_A_KA_v_MAS | -0.1709 | -0.6555 | -0.15025 | -0.0919 | -0.54207 | 0.10831 | -0.15649 | 0.05629 | 0.08134 | 0.03829 | -0.18103 | -0.02721 |
| P2_Q_AB_nCi_2_SS1_A_KA_v_MAS | -0.1856 | -0.0270 | -0.06445 | -0.0139 | -0.76738 | 0.23321 | -0.10023 | -0.03169 | -0.06285 | -0.02222 | -0.25769 | 0.04208 |
| P2_Q_AB_nCi_2_SS2_A_KA_v_MAS | -0.1821 | -0.1535 | -0.08298 | -0.0576 | -0.73549 | 0.12773 | -0.15783 | 0.00061 | 0.10762 | -0.01114 | -0.29534 | 0.13940 |
| P2_Q_AB_nCi_2_SS3_A_KA_v_MAS | -0.1743 | -0.3185 | -0.06046 | -0.0454 | -0.74353 | 0.17856 | -0.10354 | 0.02862 | 0.14988 | -0.03747 | -0.26579 | 0.02347 |
| P2_Q_AB_nCi_2_SS4_A_KA_v_MAS | -0.1835 | -0.3638 | -0.08198 | -0.0624 | -0.70007 | 0.11379 | -0.13417 | 0.04294 | 0.18907 | -0.02218 | -0.28231 | 0.08116 |
| P2_Q_AB_nCi_2_SS5_A_KA_v_MAS | -0.1728 | -0.4390 | -0.06974 | -0.0654 | -0.70826 | 0.14715 | -0.11657 | 0.04648 | 0.16971 | -0.02209 | -0.24431 | 0.02018 |
| P2_Q_AB_nCi_2_SS6_A_KA_v_MAS | -0.1804 | -0.4569 | -0.08385 | -0.0730 | -0.66989 | 0.09469 | -0.12299 | 0.04820 | 0.18821 | -0.01519 | -0.26138 | 0.06930 |
| P3_Q_AB_nCi_2_SS1_A_KA_v_MAS | -0.1566 | -0.0071 | -0.05504 | 0.0014 | -0.77211 | 0.27801 | -0.09080 | -0.04259 | -0.07530 | -0.03931 | -0.22331 | 0.02160 |
| P3_Q_AB_nCi_2_SS2_A_KA_v_MAS | -0.1550 | -0.0965 | -0.08676 | -0.0389 | -0.74335 | 0.18101 | -0.20821 | -0.02697 | 0.07851 | -0.02656 | -0.26109 | 0.17851 |
| P3_Q_AB_nCi_2_SS3_A_KA_v_MAS | -0.1504 | -0.2327 | -0.06098 | -0.0280 | -0.78365 | 0.24174 | -0.12572 | 0.00286 | 0.11411 | -0.04509 | -0.23380 | 0.04870 |
| P3_Q_AB_nCi_2_SS4_A_KA_v_MAS | -0.1575 | -0.2742 | -0.07907 | -0.0399 | -0.73829 | 0.16046 | -0.15522 | 0.00855 | 0.16589 | -0.03484 | -0.27047 | 0.13698 |
| P3_Q_AB_nCi_2_SS5_A_KA_v_MAS | -0.1490 | -0.3447 | -0.06767 | -0.0477 | -0.75449 | 0.20864 | -0.13657 | 0.02139 | 0.15225 | -0.03057 | -0.22118 | 0.06048 |
| P3_Q_AB_nCi_2_SS6_A_KA_v_MAS | -0.1544 | -0.3643 | -0.07752 | -0.0501 | -0.71558 | 0.13569 | -0.13723 | 0.01653 | 0.17964 | -0.02798 | -0.25875 | 0.12727 |
| HM_Q_AB_nCi_2_SS1_A_KA_v_MAS | -0.2577 | -0.0651 | -0.09631 | -0.0779 | -0.59410 | 0.01698 | -0.06952 | 0.04938 | 0.06074 | 0.04482 | -0.30428 | 0.03025 |
| HM_Q_AB_nCi_2_SS3_A_KA_v_MAS | -0.3021 | -0.4717 | -0.02595 | -0.1191 | -0.43560 | 0.07476 | -0.05942 | 0.11095 | 0.25529 | 0.01071 | -0.16890 | -0.10989 |
| HM_Q_AB_nCi_2_SS5_A_KA_v_MAS | -0.3309 | -0.5289 | -0.11139 | -0.1700 | -0.39740 | 0.10769 | -0.11042 | 0.10240 | 0.13349 | 0.07505 | -0.10549 | -0.11770 |
| HM_Q_AB_nCi_2_SS6_A_KA_v_MAS | -0.3377 | -0.4965 | -0.15567 | -0.1746 | -0.29826 | 0.08659 | -0.12432 | 0.10989 | 0.10035 | 0.07552 | -0.02055 | -0.09202 |
| S_Q_AB_nCi_2_SS3_A_KA_v_MAS | 0.0550 | 0.8592 | 0.08740 | 0.0289 | 0.04496 | 0.10827 | 0.03896 | -0.05191 | 0.04005 | -0.02915 | 0.13092 | 0.00715 |
| S_Q_AB_nCi_2_SS5_A_KA_v_MAS | 0.0549 | 0.8465 | 0.06653 | 0.0064 | 0.06390 | 0.14381 | 0.01732 | -0.03618 | 0.03210 | -0.01265 | 0.14562 | 0.02366 |
| SD_Q_AB_nCi_2_SS0_A_KA_v_MAS | -0.1249 | -0.2048 | -0.12339 | -0.0677 | -0.53763 | 0.14392 | -0.48178 | -0.05049 | -0.12549 | 0.01844 | -0.08834 | 0.23109 |
| SD_Q_AB_nCi_2_SS1_A_KA_v_MAS | -0.1293 | 0.0520 | -0.01036 | 0.0401 | -0.70290 | 0.26280 | -0.05856 | -0.10527 | -0.15341 | -0.05554 | -0.19982 | 0.03755 |
| SD_Q_AB_nCi_2_SS2_A_KA_v_MAS | -0.1291 | -0.2177 | -0.09726 | -0.0205 | -0.68928 | 0.20749 | -0.26785 | -0.06788 | -0.01777 | -0.02796 | -0.22300 | 0.24765 |
| SD_Q_AB_nCi_2_SS3_A_KA_v_MAS | -0.0644 | -0.1231 | -0.05852 | 0.0229 | -0.74458 | 0.28087 | -0.16685 | -0.08126 | -0.07187 | -0.05406 | -0.16767 | 0.14155 |
| SD_Q_AB_nCi_2_SS4_A_KA_v_MAS | -0.0957 | -0.2684 | -0.06198 | 0.0091 | -0.71001 | 0.18249 | -0.15290 | -0.07783 | 0.01021 | -0.02937 | -0.25537 | 0.24467 |
| SD_Q_AB_nCi_2_SS5_A_KA_v_MAS | -0.0361 | -0.1695 | -0.03516 | 0.0330 | -0.72515 | 0.26438 | -0.15509 | -0.07793 | 0.01488 | -0.05850 | -0.16589 | 0.19879 |
| SD_Q_AB_nCi_2_SS6_A_KA_v_MAS | -0.0759 | -0.2739 | -0.03302 | 0.0213 | -0.70791 | 0.16312 | -0.12455 | -0.07333 | 0.06681 | -0.03662 | -0.25997 | 0.25293 |
| VC_Q_AB_nCi_2_SS0_A_KA_v_MAS | 0.0429 | 0.9269 | 0.12678 | 0.0357 | 0.15053 | 0.04075 | 0.02924 | -0.03030 | 0.01766 | 0.00288 | 0.04833 | 0.07138 |
| VC_Q_AB_nCi_2_SS1_A_KA_v_MAS | 0.0014 | 0.9301 | 0.09641 | 0.0032 | 0.09236 | -0.12454 | 0.13242 | -0.00975 | 0.05453 | 0.01787 | -0.04480 | 0.00031 |
| VC_Q_AB_nCi_2_SS2_A_KA_v_MAS | 0.0262 | 0.9321 | 0.11732 | 0.0350 | 0.05007 | 0.03441 | 0.09665 | -0.02537 | 0.02931 | -0.02183 | -0.04580 | 0.06930 |
| VC_Q_AB_nCi_2_SS3_A_KA_v_MAS | 0.0418 | 0.9474 | 0.08750 | 0.0289 | 0.07491 | -0.02042 | 0.10112 | -0.01691 | 0.03355 | -0.01822 | -0.01815 | 0.03165 |
| VC_Q_AB_nCi_2_SS4_A_KA_v_MAS | 0.0464 | 0.9282 | 0.11521 | 0.0471 | 0.03929 | 0.02241 | 0.12553 | -0.03595 | 0.03865 | -0.01042 | -0.06249 | 0.07852 |
| VC_Q_AB_nCi_2_SS5_A_KA_v_MAS | 0.0578 | 0.9407 | 0.10063 | 0.0436 | 0.09144 | 0.01213 | 0.09863 | -0.02298 | 0.04192 | -0.02507 | -0.00163 | 0.05667 |
| VC_Q_AB_nCi_2_SS6_A_KA_v_MAS | 0.0560 | 0.9255 | 0.12531 | 0.0506 | 0.04124 | 0.01456 | 0.12689 | -0.03326 | 0.06859 | -0.00811 | -0.06449 | 0.08601 |
| S_Q_AB_nCi_2_MP0_A_KA_a_MAS | 0.3033 | 0.5412 | 0.07633 | 0.0609 | 0.12878 | -0.00141 | 0.17736 | -0.04272 | -0.16645 | 0.02163 | -0.07683 | -0.03865 |
| S_Q_AB_nCi_2_MP1_A_KA_a_MAS | 0.1260 | 0.0880 | 0.04046 | 0.0154 | 0.15587 | 0.01661 | 0.51996 | -0.00080 | -0.05817 | 0.03127 | 0.11497 | 0.06039 |
| S_Q_AB_nCi_2_MP3_A_KA_a_MAS | 0.1064 | 0.1567 | 0.06266 | 0.0172 | 0.12117 | -0.05192 | 0.60489 | 0.01292 | -0.06932 | 0.03374 | 0.10294 | 0.12194 |
| S_Q_AB_nCi_2_MP5_A_KA_a_MAS | 0.1240 | 0.1528 | 0.05102 | 0.0280 | 0.11563 | -0.05880 | 0.64258 | 0.02045 | -0.06653 | 0.03078 | 0.09186 | 0.13103 |
| VC_Q_AB_nCi_2_MP0_A_KA_a_MAS | 0.2736 | 0.5894 | 0.09329 | 0.0707 | 0.10216 | -0.01797 | 0.22205 | -0.06706 | -0.14085 | 0.01672 | -0.06753 | -0.00666 |
| S_Q_AB_nCi_2_MP2_A_KA_h_MAS | 0.2251 | 0.7489 | 0.11190 | 0.0745 | 0.25236 | -0.05595 | 0.01905 | 0.00793 | 0.06635 | 0.12340 | -0.15255 | -0.06487 |
| S_Q_AB_nCi_2_MP3_A_KA_h_MAS | 0.1291 | 0.8549 | 0.04738 | 0.0486 | 0.06938 | 0.10628 | -0.05571 | 0.01443 | 0.12566 | 0.01980 | 0.07232 | -0.03226 |
| S_Q_AB_nCi_2_MP4_A_KA_h_MAS | 0.2040 | 0.7868 | 0.07969 | 0.0719 | 0.23290 | 0.01959 | -0.02356 | 0.04280 | 0.16929 | 0.08837 | -0.08001 | -0.09603 |
| S_Q_AB_nCi_2_MP5_A_KA_h_MAS | 0.1472 | 0.8171 | 0.02296 | 0.0565 | 0.11017 | 0.19950 | -0.08676 | 0.03182 | 0.11692 | 0.01682 | 0.12619 | -0.03077 |
| S_Q_AB_nCi_2_MP6_A_KA_h_MAS | 0.2022 | 0.7849 | 0.04886 | 0.0662 | 0.20540 | 0.07334 | -0.03337 | 0.05778 | 0.20311 | 0.07205 | -0.00288 | -0.10126 |
| VC_Q_AB_nCi_2_MP0_A_KA_h_MAS | 0.1169 | 0.9037 | 0.11913 | 0.0325 | 0.17354 | 0.03748 | 0.09641 | -0.01745 | 0.06661 | 0.07425 | 0.02030 | 0.07139 |
| VC_Q_AB_nCi_2_MP1_A_KA_h_MAS | 0.0772 | 0.9133 | 0.06326 | 0.0164 | 0.09479 | -0.16289 | 0.07682 | -0.00758 | 0.08923 | 0.07921 | -0.13978 | -0.03621 |
| VC_Q_AB_nCi_2_MP2_A_KA_h_MAS | 0.1293 | 0.8782 | 0.10069 | 0.0471 | 0.13315 | -0.08440 | 0.09841 | -0.01863 | 0.09506 | 0.09015 | -0.19596 | -0.04247 |
| VC_Q_AB_nCi_2_MP3_A_KA_h_MAS | 0.1078 | 0.9280 | 0.06396 | 0.0348 | 0.08455 | -0.03111 | 0.05656 | -0.00254 | 0.12882 | 0.04436 | -0.10136 | -0.01765 |
| VC_Q_AB_nCi_2_MP4_A_KA_h_MAS | 0.1468 | 0.8647 | 0.08041 | 0.0595 | 0.07237 | -0.08137 | 0.11494 | -0.01814 | 0.17088 | 0.08460 | -0.20717 | -0.06415 |
| VC_Q_AB_nCi_2_MP5_A_KA_h_MAS | 0.1331 | 0.9147 | 0.07211 | 0.0633 | 0.10245 | 0.06040 | 0.04463 | -0.00520 | 0.15085 | 0.01193 | -0.04395 | -0.00980 |
| VC_Q_AB_nCi_2_MP6_A_KA_h_MAS | 0.1620 | 0.8531 | 0.07532 | 0.0698 | 0.04251 | -0.04719 | 0.11081 | -0.00295 | 0.24427 | 0.06463 | -0.16771 | -0.07295 |
| P2_Q_AB_nCi_2_MP0_A_KA_v_MAS | -0.7348 | -0.0859 | -0.05500 | -0.1360 | -0.34292 | 0.14687 | -0.26790 | 0.03787 | -0.01650 | 0.12321 | -0.08840 | 0.21167 |
| P3_Q_AB_nCi_2_MP0_A_KA_v_MAS | -0.7177 | -0.0890 | -0.05847 | -0.1344 | -0.35499 | 0.14372 | -0.29469 | 0.03046 | -0.02252 | 0.11596 | -0.09472 | 0.19894 |
| HM_Q_AB_nCi_2_MP0_A_KA_v_MAS | -0.7754 | -0.0738 | -0.04063 | -0.1352 | -0.28679 | 0.15812 | -0.15328 | 0.05997 | 0.01151 | 0.13660 | -0.07274 | 0.23620 |
| S_Q_AB_nCi_2_MP2_A_KA_v_MAS | 0.0993 | 0.8554 | 0.01581 | 0.0607 | 0.18242 | 0.13693 | 0.07845 | -0.05661 | 0.03693 | -0.01881 | 0.03706 | 0.02688 |
| S_Q_AB_nCi_2_MP3_A_KA_v_MAS | 0.0806 | 0.8451 | -0.05431 | 0.0411 | 0.02463 | 0.17535 | 0.02564 | -0.01516 | 0.05347 | -0.05363 | 0.14734 | -0.06740 |
| S_Q_AB_nCi_2_MP4_A_KA_v_MAS | 0.1120 | 0.8450 | -0.05110 | 0.0585 | 0.13547 | 0.17435 | 0.01585 | 0.00166 | 0.10451 | -0.03689 | 0.04360 | 0.00290 |
| S_Q_AB_nCi_2_MP5_A_KA_v_MAS | 0.1299 | 0.7889 | -0.08410 | 0.0538 | 0.04061 | 0.22092 | -0.03065 | 0.02492 | 0.02370 | -0.05375 | 0.13738 | -0.05025 |
| S_Q_AB_nCi_2_MP6_A_KA_v_MAS | 0.1529 | 0.8007 | -0.08452 | 0.0652 | 0.12851 | 0.18588 | -0.01199 | 0.03045 | 0.09311 | -0.03446 | 0.05063 | -0.00632 |
| SD_Q_AB_nCi_2_MP0_A_KA_v_MAS | -0.6857 | -0.1945 | -0.05907 | -0.1357 | -0.36179 | 0.11171 | -0.28916 | 0.03250 | -0.02949 | 0.11463 | -0.10930 | 0.17825 |
| VC_Q_AB_nCi_2_MP0_A_KA_v_MAS | 0.0429 | 0.9269 | 0.12678 | 0.0357 | 0.15053 | 0.04075 | 0.02924 | -0.03030 | 0.01766 | 0.00288 | 0.04833 | 0.07138 |
| VC_Q_AB_nCi_2_MP1_A_KA_v_MAS | 0.0094 | 0.9264 | 0.06292 | -0.0070 | 0.08822 | -0.13324 | 0.14763 | -0.00608 | 0.07435 | 0.02474 | -0.04739 | -0.02910 |
| VC_Q_AB_nCi_2_MP2_A_KA_v_MAS | 0.0483 | 0.9299 | 0.08154 | 0.0343 | 0.02895 | 0.04362 | 0.13443 | -0.02335 | 0.07345 | -0.01652 | -0.06489 | 0.03657 |
| VC_Q_AB_nCi_2_MP3_A_KA_v_MAS | 0.0638 | 0.9430 | 0.05321 | 0.0284 | 0.01961 | 0.00277 | 0.12279 | -0.01077 | 0.08498 | -0.01576 | -0.03012 | -0.02839 |
| VC_Q_AB_nCi_2_MP4_A_KA_v_MAS | 0.0853 | 0.9192 | 0.06437 | 0.0490 | -0.00454 | 0.06692 | 0.13995 | -0.02886 | 0.10752 | -0.01729 | -0.07705 | 0.01986 |
| VC_Q_AB_nCi_2_MP5_A_KA_v_MAS | 0.1049 | 0.9253 | 0.05215 | 0.0632 | 0.01581 | 0.08170 | 0.09267 | -0.01051 | 0.10299 | -0.04666 | -0.00337 | -0.02165 |
| VC_Q_AB_nCi_2_MP6_A_KA_v_MAS | 0.1211 | 0.9019 | 0.06026 | 0.0683 | -0.01378 | 0.09681 | 0.11785 | -0.01627 | 0.15348 | -0.03559 | -0.06639 | 0.00784 |
| PN_Q_AB_nCi_2_SS0_M_KA_a_MAS | 0.0432 | 0.1366 | 0.91012 | 0.0783 | 0.02628 | 0.06277 | 0.07829 | 0.02161 | 0.07165 | 0.11568 | 0.04251 | -0.06970 |
| P2_Q_AB_nCi_2_SS2_M_KA_a_MAS | 0.1304 | 0.1215 | 0.62843 | 0.5412 | -0.00890 | 0.28079 | 0.00575 | -0.01987 | -0.06142 | -0.09093 | 0.07421 | -0.04659 |
| P2_Q_AB_nCi_2_SS4_M_KA_a_MAS | 0.0880 | 0.2880 | 0.60172 | 0.3549 | -0.06591 | 0.22701 | -0.00989 | 0.02162 | 0.15269 | -0.10377 | 0.01998 | -0.01131 |
| P3_Q_AB_nCi_2_SS2_M_KA_a_MAS | 0.1489 | 0.1251 | 0.63842 | 0.5567 | 0.00276 | 0.27001 | 0.01190 | -0.01304 | -0.05777 | -0.06611 | 0.06937 | -0.04972 |
| P3_Q_AB_nCi_2_SS4_M_KA_a_MAS | 0.1159 | 0.2897 | 0.61593 | 0.3894 | -0.05111 | 0.22230 | -0.00268 | 0.02650 | 0.14878 | -0.08165 | 0.01929 | -0.01558 |
| S_Q_AB_nCi_2_SS0_M_KA_a_MAS | 0.1735 | 0.0588 | 0.15722 | 0.9602 | 0.02336 | 0.00734 | 0.02598 | 0.00525 | -0.00533 | 0.00641 | 0.00766 | 0.01206 |
| S_Q_AB_nCi_2_SS2_M_KA_a_MAS | 0.2094 | 0.0540 | 0.16112 | 0.9479 | 0.02746 | 0.01293 | 0.02794 | 0.02269 | -0.01922 | 0.02531 | 0.00172 | 0.00483 |
| S_Q_AB_nCi_2_SS4_M_KA_a_MAS | 0.2125 | 0.0589 | 0.11342 | 0.9449 | 0.01840 | 0.02433 | 0.02231 | 0.01855 | -0.02202 | -0.00205 | 0.00197 | 0.01183 |
| S_Q_AB_nCi_2_SS6_M_KA_a_MAS | 0.2113 | 0.0592 | 0.08491 | 0.9351 | 0.03093 | 0.03426 | 0.02943 | 0.02429 | -0.01671 | -0.01863 | -0.00023 | 0.01181 |
| SD_Q_AB_nCi_2_SS0_M_KA_a_MAS | 0.1361 | 0.1107 | 0.79579 | 0.4626 | 0.04811 | 0.10037 | 0.06092 | -0.01240 | -0.00804 | 0.11756 | 0.02359 | -0.07003 |
| SD_Q_AB_nCi_2_SS2_M_KA_a_MAS | 0.1069 | 0.1423 | 0.82089 | 0.3644 | 0.02031 | 0.22028 | 0.03323 | -0.00994 | -0.01049 | 0.00559 | 0.06431 | -0.07126 |
| SD_Q_AB_nCi_2_SS4_M_KA_a_MAS | 0.0953 | 0.2761 | 0.77259 | 0.2678 | -0.02099 | 0.18193 | 0.03126 | 0.02258 | 0.15441 | 0.00532 | 0.02385 | -0.03603 |
| SD_Q_AB_nCi_2_SS6_M_KA_a_MAS | 0.0889 | 0.3157 | 0.72616 | 0.2103 | -0.01829 | 0.14554 | 0.04096 | 0.03947 | 0.23135 | 0.02641 | 0.00087 | -0.02076 |
| VC_Q_AB_nCi_2_SS0_M_KA_a_MAS | 0.1961 | 0.0531 | 0.09745 | 0.9634 | 0.01379 | 0.01118 | 0.02340 | 0.01259 | -0.00509 | -0.00538 | 0.01156 | 0.02184 |
| S_Q_AB_nCi_2_SS0_M_KA_h_MAS | 0.2821 | 0.1009 | 0.25036 | 0.8879 | 0.04199 | -0.02920 | 0.03863 | 0.04893 | 0.06311 | 0.09060 | -0.01377 | -0.00139 |
| S_Q_AB_nCi_2_SS2_M_KA_h_MAS | 0.2835 | 0.0972 | 0.23460 | 0.8927 | 0.04435 | -0.01286 | 0.03414 | 0.04586 | 0.05101 | 0.07229 | -0.00958 | -0.00437 |
| S_Q_AB_nCi_2_SS4_M_KA_h_MAS | 0.2778 | 0.1006 | 0.17089 | 0.9150 | 0.03106 | -0.01502 | 0.03076 | 0.04720 | 0.06009 | 0.04896 | -0.01406 | 0.00484 |
| S_Q_AB_nCi_2_SS6_M_KA_h_MAS | 0.2648 | 0.1032 | 0.13100 | 0.9251 | 0.02547 | -0.01814 | 0.02890 | 0.05627 | 0.06614 | 0.03267 | -0.01345 | 0.00672 |
| VC_Q_AB_nCi_2_SS0_M_KA_h_MAS | 0.2521 | 0.0705 | 0.13515 | 0.9422 | 0.02256 | -0.00508 | 0.02867 | 0.03320 | 0.02908 | 0.03795 | 0.00033 | 0.01656 |
| VC_Q_AB_nCi_2_SS1_M_KA_h_MAS | 0.2603 | 0.0843 | 0.11853 | 0.9396 | 0.01302 | 0.01422 | 0.02192 | 0.03819 | 0.01546 | 0.02446 | -0.00513 | 0.00968 |
| VC_Q_AB_nCi_2_SS2_M_KA_h_MAS | 0.2428 | 0.0686 | 0.15287 | 0.9351 | 0.02552 | 0.03491 | 0.02231 | 0.02362 | 0.00140 | -0.00701 | 0.01169 | 0.00701 |
| VC_Q_AB_nCi_2_SS3_M_KA_h_MAS | 0.2392 | 0.0853 | 0.09887 | 0.9471 | -0.00138 | 0.01253 | 0.01868 | 0.04401 | 0.02040 | -0.01056 | -0.00295 | 0.01472 |
| VC_Q_AB_nCi_2_SS4_M_KA_h_MAS | 0.2404 | 0.1051 | 0.16436 | 0.9346 | 0.01094 | 0.01645 | 0.01455 | 0.02992 | 0.04315 | -0.01657 | -0.00017 | 0.00920 |
| VC_Q_AB_nCi_2_SS5_M_KA_h_MAS | 0.2363 | 0.0807 | 0.11378 | 0.9483 | 0.01042 | 0.01692 | 0.02069 | 0.03999 | 0.00466 | -0.00561 | 0.00328 | 0.01370 |
| VC_Q_AB_nCi_2_SS6_M_KA_h_MAS | 0.2392 | 0.1211 | 0.16486 | 0.9293 | 0.00459 | 0.00459 | 0.01154 | 0.03442 | 0.06346 | -0.01596 | -0.00755 | 0.01181 |
| PN_Q_AB_nCi_2_SS0_M_KA_v_MAS | 0.0348 | 0.1793 | 0.90672 | 0.0757 | 0.06221 | -0.04554 | 0.08299 | 0.03632 | 0.12763 | 0.11984 | 0.03016 | -0.06090 |
| PN_Q_AB_nCi_2_SS1_M_KA_v_MAS | 0.0316 | 0.2211 | 0.85981 | 0.0721 | 0.06283 | -0.13975 | 0.07256 | 0.04833 | 0.19876 | 0.10483 | 0.01993 | -0.04353 |
| PN_Q_AB_nCi_2_SS2_M_KA_v_MAS | 0.0216 | 0.2059 | 0.90342 | 0.0607 | 0.06196 | -0.02970 | 0.07960 | 0.03883 | 0.13480 | 0.10251 | 0.03958 | -0.05334 |
| PN_Q_AB_nCi_2_SS3_M_KA_v_MAS | 0.0261 | 0.2376 | 0.86348 | 0.0653 | 0.07983 | -0.11408 | 0.08025 | 0.04814 | 0.19133 | 0.11761 | 0.02632 | -0.03563 |
| PN_Q_AB_nCi_2_SS4_M_KA_v_MAS | 0.0181 | 0.2386 | 0.88281 | 0.0511 | 0.06255 | -0.05453 | 0.08021 | 0.04750 | 0.17162 | 0.11529 | 0.02918 | -0.04287 |
| PN_Q_AB_nCi_2_SS5_M_KA_v_MAS | 0.0242 | 0.2373 | 0.86409 | 0.0605 | 0.09097 | -0.10674 | 0.08176 | 0.04742 | 0.18392 | 0.12255 | 0.03002 | -0.03583 |
| PN_Q_AB_nCi_2_SS6_M_KA_v_MAS | 0.0158 | 0.2466 | 0.87064 | 0.0457 | 0.06926 | -0.06801 | 0.08220 | 0.05139 | 0.18478 | 0.12580 | 0.02547 | -0.03884 |
| P2_Q_AB_nCi_2_SS2_M_KA_v_MAS | 0.1303 | 0.1477 | 0.66529 | 0.6197 | 0.03246 | 0.09649 | 0.02813 | -0.00393 | 0.01225 | -0.06712 | 0.04298 | -0.04071 |
| P2_Q_AB_nCi_2_SS3_M_KA_v_MAS | 0.1152 | 0.2326 | 0.66735 | 0.5431 | 0.03195 | -0.06732 | 0.04527 | 0.04433 | 0.17838 | -0.04556 | -0.00367 | -0.01776 |
| P2_Q_AB_nCi_2_SS4_M_KA_v_MAS | 0.0928 | 0.2659 | 0.68683 | 0.4669 | -0.00820 | 0.02973 | 0.01623 | 0.02957 | 0.17431 | -0.07182 | 0.00467 | -0.02085 |
| P2_Q_AB_nCi_2_SS5_M_KA_v_MAS | 0.0961 | 0.2677 | 0.72141 | 0.4445 | 0.05202 | -0.07564 | 0.05187 | 0.05857 | 0.22312 | -0.02727 | 0.00070 | -0.01935 |
| P2_Q_AB_nCi_2_SS6_M_KA_v_MAS | 0.0690 | 0.2882 | 0.71818 | 0.3726 | 0.01849 | -0.02643 | 0.04212 | 0.05535 | 0.24401 | -0.03712 | -0.00204 | -0.01935 |
| P3_Q_AB_nCi_2_SS2_M_KA_v_MAS | 0.1428 | 0.1529 | 0.66414 | 0.6382 | 0.04208 | 0.08265 | 0.03272 | -0.00006 | 0.02055 | -0.04381 | 0.03735 | -0.04053 |
| P3_Q_AB_nCi_2_SS3_M_KA_v_MAS | 0.1314 | 0.2295 | 0.66176 | 0.5683 | 0.04243 | -0.06991 | 0.05031 | 0.04467 | 0.16883 | -0.01497 | -0.00347 | -0.02060 |
| P3_Q_AB_nCi_2_SS4_M_KA_v_MAS | 0.1136 | 0.2630 | 0.68056 | 0.5182 | 0.00947 | 0.01982 | 0.02201 | 0.02984 | 0.16765 | -0.04636 | 0.00328 | -0.02225 |
| P3_Q_AB_nCi_2_SS6_M_KA_v_MAS | 0.0959 | 0.2863 | 0.69912 | 0.4438 | 0.03136 | -0.02941 | 0.04003 | 0.05002 | 0.23044 | -0.01587 | -0.00519 | -0.02041 |
| HM_Q_AB_nCi_2_SS2_M_KA_v_MAS | 0.0610 | 0.1407 | 0.60384 | 0.5351 | -0.02270 | 0.14316 | -0.00040 | -0.02740 | -0.00456 | -0.14519 | 0.06727 | -0.02660 |
| S_Q_AB_nCi_2_SS0_M_KA_v_MAS | 0.1716 | 0.0526 | 0.14619 | 0.9635 | 0.01508 | 0.00974 | 0.02645 | 0.00602 | -0.00606 | 0.00277 | 0.01170 | 0.01574 |
| S_Q_AB_nCi_2_SS2_M_KA_v_MAS | 0.1831 | 0.0480 | 0.14336 | 0.9628 | 0.02157 | 0.01245 | 0.02509 | 0.00559 | -0.01019 | 0.00616 | 0.00373 | 0.01166 |
| S_Q_AB_nCi_2_SS4_M_KA_v_MAS | 0.1765 | 0.0551 | 0.06178 | 0.9715 | 0.00660 | 0.01636 | 0.01899 | 0.00645 | -0.00154 | -0.02498 | 0.00351 | 0.02491 |
| S_Q_AB_nCi_2_SS6_M_KA_v_MAS | 0.1658 | 0.0538 | 0.01988 | 0.9731 | 0.00276 | 0.01469 | 0.01768 | 0.01313 | 0.00388 | -0.04099 | 0.00282 | 0.02830 |
| SD_Q_AB_nCi_2_SS0_M_KA_v_MAS | 0.1313 | 0.1573 | 0.80386 | 0.4672 | 0.08026 | -0.02949 | 0.06646 | 0.00721 | 0.07771 | 0.12114 | 0.00569 | -0.05932 |
| SD_Q_AB_nCi_2_SS1_M_KA_v_MAS | 0.1402 | 0.2140 | 0.73203 | 0.4452 | 0.06638 | -0.11429 | 0.05318 | 0.01270 | 0.15170 | 0.08232 | -0.02058 | -0.05627 |
| SD_Q_AB_nCi_2_SS2_M_KA_v_MAS | 0.1064 | 0.1753 | 0.82038 | 0.4457 | 0.06676 | 0.03806 | 0.04855 | 0.00237 | 0.05924 | 0.03605 | 0.02949 | -0.05975 |
| SD_Q_AB_nCi_2_SS3_M_KA_v_MAS | 0.1208 | 0.2289 | 0.72707 | 0.4645 | 0.07187 | -0.08288 | 0.06323 | 0.02781 | 0.15930 | 0.07819 | -0.01786 | -0.03988 |
| SD_Q_AB_nCi_2_SS4_M_KA_v_MAS | 0.0957 | 0.2458 | 0.80339 | 0.3900 | 0.05409 | -0.01194 | 0.04638 | 0.02170 | 0.14820 | 0.05607 | 0.00691 | -0.04682 |
| SD_Q_AB_nCi_2_SS5_M_KA_v_MAS | 0.1253 | 0.2231 | 0.75062 | 0.4400 | 0.10225 | -0.07689 | 0.06381 | 0.02279 | 0.14481 | 0.10466 | -0.00250 | -0.04289 |
| SD_Q_AB_nCi_2_SS6_M_KA_v_MAS | 0.0975 | 0.2641 | 0.78231 | 0.3628 | 0.06188 | -0.04279 | 0.04888 | 0.02808 | 0.18408 | 0.08219 | -0.00507 | -0.03952 |
| VC_Q_AB_nCi_2_SS0_M_KA_v_MAS | 0.1953 | 0.0479 | 0.09342 | 0.9640 | 0.00856 | 0.01391 | 0.02461 | 0.01373 | -0.00636 | -0.00767 | 0.01402 | 0.02381 |
| VC_Q_AB_nCi_2_SS1_M_KA_v_MAS | 0.2097 | 0.0545 | 0.08854 | 0.9551 | -0.00088 | 0.03466 | 0.01837 | 0.01504 | -0.02364 | -0.02046 | 0.00915 | 0.01891 |
| VC_Q_AB_nCi_2_SS2_M_KA_v_MAS | 0.1896 | 0.0361 | 0.10164 | 0.9583 | 0.00310 | 0.04644 | 0.01893 | 0.01101 | -0.02835 | -0.05601 | 0.01862 | 0.01745 |
| VC_Q_AB_nCi_2_SS3_M_KA_v_MAS | 0.1959 | 0.0552 | 0.07066 | 0.9597 | -0.01372 | 0.02090 | 0.01865 | 0.03022 | -0.00526 | -0.04619 | 0.00466 | 0.02330 |
| VC_Q_AB_nCi_2_SS4_M_KA_v_MAS | 0.1919 | 0.0649 | 0.12024 | 0.9573 | -0.00764 | 0.02949 | 0.01261 | 0.02004 | 0.01055 | -0.05740 | 0.00842 | 0.01882 |
| VC_Q_AB_nCi_2_SS5_M_KA_v_MAS | 0.1998 | 0.0495 | 0.09001 | 0.9611 | 0.00117 | 0.02332 | 0.02005 | 0.02600 | -0.01502 | -0.03250 | 0.01115 | 0.01985 |
| VC_Q_AB_nCi_2_SS6_M_KA_v_MAS | 0.1983 | 0.0769 | 0.12848 | 0.9534 | -0.00820 | 0.01905 | 0.01071 | 0.02264 | 0.02870 | -0.04689 | 0.00284 | 0.01969 |
| S_Q_AB_nCi_2_MP0_M_KA_a_MAS | 0.1735 | 0.0588 | 0.15722 | 0.9602 | 0.02336 | 0.00734 | 0.02598 | 0.00525 | -0.00533 | 0.00641 | 0.00766 | 0.01206 |
| S_Q_AB_nCi_2_MP2_M_KA_a_MAS | 0.2042 | 0.0544 | 0.15485 | 0.9543 | 0.02614 | 0.01688 | 0.02707 | 0.01442 | -0.01332 | 0.01203 | 0.00790 | 0.01203 |
| S_Q_AB_nCi_2_MP4_M_KA_a_MAS | 0.2075 | 0.0545 | 0.11161 | 0.9439 | 0.03505 | 0.02697 | 0.02870 | 0.01651 | -0.01188 | -0.01253 | 0.01063 | 0.00608 |
| S_Q_AB_nCi_2_MP6_M_KA_a_MAS | 0.2153 | 0.0697 | 0.08684 | 0.8930 | 0.06769 | 0.03063 | 0.03866 | 0.03372 | 0.01049 | -0.01351 | 0.00019 | 0.00873 |
| VC_Q_AB_nCi_2_MP0_M_KA_a_MAS | 0.1961 | 0.0531 | 0.09745 | 0.9634 | 0.01379 | 0.01118 | 0.02340 | 0.01259 | -0.00509 | -0.00538 | 0.01156 | 0.02184 |
| S_Q_AB_nCi_2_MP0_M_KA_h_MAS | 0.2821 | 0.1009 | 0.25036 | 0.8879 | 0.04199 | -0.02920 | 0.03863 | 0.04893 | 0.06311 | 0.09060 | -0.01377 | -0.00139 |
| S_Q_AB_nCi_2_MP2_M_KA_h_MAS | 0.2738 | 0.0979 | 0.22930 | 0.8997 | 0.04634 | -0.01881 | 0.03260 | 0.04628 | 0.04655 | 0.07553 | -0.00746 | -0.00479 |
| S_Q_AB_nCi_2_MP4_M_KA_h_MAS | 0.2578 | 0.0917 | 0.16771 | 0.9168 | 0.02718 | -0.01607 | 0.03547 | 0.05083 | 0.02809 | 0.04361 | -0.01053 | 0.00119 |
| S_Q_AB_nCi_2_MP6_M_KA_h_MAS | 0.2433 | 0.0864 | 0.14114 | 0.9149 | 0.01717 | -0.00434 | 0.03212 | 0.05035 | 0.02158 | 0.01146 | -0.00460 | 0.00230 |
| VC_Q_AB_nCi_2_MP0_M_KA_h_MAS | 0.2521 | 0.0705 | 0.13515 | 0.9422 | 0.02256 | -0.00508 | 0.02867 | 0.03320 | 0.02908 | 0.03795 | 0.00033 | 0.01656 |
| VC_Q_AB_nCi_2_MP1_M_KA_h_MAS | 0.2580 | 0.0874 | 0.11616 | 0.9405 | 0.01384 | 0.01046 | 0.02236 | 0.03651 | 0.01671 | 0.02512 | -0.00512 | 0.00959 |
| VC_Q_AB_nCi_2_MP2_M_KA_h_MAS | 0.2308 | 0.0745 | 0.13900 | 0.9394 | 0.02321 | 0.02908 | 0.02068 | 0.02221 | 0.00472 | -0.01582 | 0.01207 | 0.00808 |
| VC_Q_AB_nCi_2_MP3_M_KA_h_MAS | 0.2253 | 0.0809 | 0.09238 | 0.9507 | -0.00244 | 0.00889 | 0.01963 | 0.04156 | 0.01099 | -0.01883 | -0.00034 | 0.01479 |
| VC_Q_AB_nCi_2_MP4_M_KA_h_MAS | 0.2231 | 0.1018 | 0.14799 | 0.9345 | 0.00676 | 0.00663 | 0.01451 | 0.03118 | 0.03786 | -0.02284 | 0.00056 | 0.00936 |
| VC_Q_AB_nCi_2_MP5_M_KA_h_MAS | 0.2263 | 0.0740 | 0.11502 | 0.9480 | 0.00261 | 0.01343 | 0.02235 | 0.03488 | -0.00105 | -0.01767 | 0.00469 | 0.01098 |
| VC_Q_AB_nCi_2_MP6_M_KA_h_MAS | 0.2233 | 0.1089 | 0.15271 | 0.9279 | 0.00015 | -0.00333 | 0.01410 | 0.03246 | 0.05168 | -0.02285 | -0.00412 | 0.01051 |
| S_Q_AB_nCi_2_MP0_M_KA_v_MAS | 0.1716 | 0.0526 | 0.14619 | 0.9635 | 0.01508 | 0.00974 | 0.02645 | 0.00602 | -0.00606 | 0.00277 | 0.01170 | 0.01574 |
| S_Q_AB_nCi_2_MP2_M_KA_v_MAS | 0.1871 | 0.0579 | 0.15605 | 0.9539 | 0.03132 | 0.00648 | 0.02326 | 0.00300 | -0.00330 | 0.01291 | -0.00155 | 0.00807 |
| S_Q_AB_nCi_2_MP4_M_KA_v_MAS | 0.2008 | 0.0681 | 0.09792 | 0.9499 | 0.01214 | 0.00924 | 0.02329 | 0.01375 | 0.01045 | -0.00359 | -0.00532 | 0.00898 |
| S_Q_AB_nCi_2_MP6_M_KA_v_MAS | 0.2209 | 0.0806 | 0.08507 | 0.9328 | 0.00921 | 0.01336 | 0.02338 | 0.03720 | 0.02653 | -0.01086 | -0.00980 | 0.00499 |
| VC_Q_AB_nCi_2_MP0_M_KA_v_MAS | 0.1953 | 0.0479 | 0.09342 | 0.9640 | 0.00856 | 0.01391 | 0.02461 | 0.01373 | -0.00636 | -0.00767 | 0.01402 | 0.02381 |
| VC_Q_AB_nCi_2_MP1_M_KA_v_MAS | 0.2111 | 0.0557 | 0.09327 | 0.9548 | 0.00281 | 0.03421 | 0.01863 | 0.01500 | -0.02518 | -0.01873 | 0.00858 | 0.01684 |
| VC_Q_AB_nCi_2_MP2_M_KA_v_MAS | 0.1884 | 0.0462 | 0.09742 | 0.9577 | 0.00463 | 0.04196 | 0.01514 | 0.01107 | -0.01877 | -0.06084 | 0.01618 | 0.01813 |
| VC_Q_AB_nCi_2_MP3_M_KA_v_MAS | 0.2049 | 0.0585 | 0.08434 | 0.9578 | -0.00740 | 0.01716 | 0.01835 | 0.03006 | -0.00174 | -0.03854 | 0.00111 | 0.01516 |
| VC_Q_AB_nCi_2_MP4_M_KA_v_MAS | 0.1991 | 0.0724 | 0.12365 | 0.9521 | -0.00421 | 0.01932 | 0.01089 | 0.02330 | 0.02369 | -0.05181 | 0.00331 | 0.01406 |
| VC_Q_AB_nCi_2_MP5_M_KA_v_MAS | 0.2202 | 0.0611 | 0.11373 | 0.9536 | 0.00038 | 0.01689 | 0.02173 | 0.03128 | 0.00286 | -0.02656 | 0.00478 | 0.00812 |
| VC_Q_AB_nCi_2_MP6_M_KA_v_MAS | 0.2175 | 0.0849 | 0.14146 | 0.9424 | -0.00477 | 0.00814 | 0.01291 | 0.03021 | 0.04651 | -0.03773 | -0.00273 | 0.01011 |
| S_Q_AB_nCi_2_SS0_X_KA_a_MAS | 0.2952 | 0.5441 | 0.09240 | 0.0483 | 0.21671 | 0.01932 | 0.15763 | -0.04660 | -0.14291 | 0.02353 | -0.06052 | -0.01785 |
| S_Q_AB_nCi_2_SS1_X_KA_a_MAS | 0.1204 | 0.0798 | 0.02601 | 0.0145 | 0.21403 | 0.08173 | 0.56118 | 0.01613 | -0.03653 | 0.07350 | 0.10250 | 0.04566 |
| S_Q_AB_nCi_2_SS2_X_KA_a_MAS | 0.1546 | 0.2035 | 0.05513 | 0.0280 | 0.04997 | 0.08277 | 0.40669 | -0.02341 | -0.13240 | 0.01764 | -0.00404 | 0.05929 |
| S_Q_AB_nCi_2_SS3_X_KA_a_MAS | 0.1072 | 0.1452 | 0.05159 | 0.0020 | 0.19345 | 0.03649 | 0.58489 | 0.01537 | -0.06066 | 0.06821 | 0.08858 | 0.10891 |
| S_Q_AB_nCi_2_SS4_X_KA_a_MAS | 0.1412 | 0.1597 | 0.02838 | 0.0005 | 0.02445 | 0.01954 | 0.57015 | -0.01326 | -0.05772 | 0.02376 | -0.03481 | 0.06093 |
| S_Q_AB_nCi_2_SS5_X_KA_a_MAS | 0.1122 | 0.1573 | 0.04109 | 0.0003 | 0.17914 | 0.00994 | 0.62025 | 0.01529 | -0.06034 | 0.06417 | 0.07180 | 0.12198 |
| S_Q_AB_nCi_2_SS6_X_KA_a_MAS | 0.1283 | 0.1552 | 0.02409 | -0.0096 | 0.02174 | 0.01019 | 0.59978 | -0.00788 | -0.04087 | 0.01863 | -0.04584 | 0.06110 |
| VC_Q_AB_nCi_2_SS0_X_KA_a_MAS | 0.2639 | 0.5926 | 0.10677 | 0.0566 | 0.20855 | 0.01146 | 0.20145 | -0.06660 | -0.11535 | 0.02186 | -0.06693 | 0.00406 |
| S_Q_AB_nCi_2_SS1_X_KA_h_MAS | 0.2981 | 0.6480 | 0.01394 | 0.1304 | -0.00804 | 0.00007 | 0.04542 | 0.01591 | -0.00055 | -0.00167 | -0.12777 | -0.07191 |
| S_Q_AB_nCi_2_SS2_X_KA_h_MAS | 0.2397 | 0.7837 | 0.11633 | 0.0759 | 0.28986 | 0.02479 | 0.06902 | -0.00060 | 0.07210 | 0.11326 | -0.11495 | 0.05370 |
| S_Q_AB_nCi_2_SS3_X_KA_h_MAS | 0.2323 | 0.7910 | 0.06463 | 0.0551 | 0.13923 | 0.03001 | 0.06837 | 0.03244 | 0.03025 | -0.00667 | 0.08054 | -0.00161 |
| S_Q_AB_nCi_2_SS4_X_KA_h_MAS | 0.2074 | 0.8073 | 0.12151 | 0.0685 | 0.26156 | -0.02390 | 0.05760 | 0.01516 | 0.10788 | 0.10525 | -0.16326 | 0.03276 |
| S_Q_AB_nCi_2_SS5_X_KA_h_MAS | 0.1810 | 0.8077 | 0.09948 | 0.0252 | 0.15199 | 0.06468 | 0.06016 | 0.04426 | 0.04313 | 0.01466 | 0.12878 | 0.01705 |
| S_Q_AB_nCi_2_SS6_X_KA_h_MAS | 0.1927 | 0.8123 | 0.12240 | 0.0601 | 0.25069 | -0.04523 | 0.05649 | 0.01685 | 0.11121 | 0.10055 | -0.17379 | 0.03246 |
| SD_Q_AB_nCi_2_SS2_X_KA_h_MAS | -0.4655 | -0.1921 | -0.00700 | -0.0831 | 0.00141 | -0.03078 | 0.08179 | 0.04053 | 0.07955 | -0.01794 | -0.66754 | -0.08943 |
| VC_Q_AB_nCi_2_SS0_X_KA_h_MAS | 0.1243 | 0.8877 | 0.11091 | 0.0279 | 0.24225 | 0.03726 | 0.12770 | -0.01433 | 0.07709 | 0.07795 | 0.00281 | 0.09060 |
| VC_Q_AB_nCi_2_SS1_X_KA_h_MAS | 0.1076 | 0.9021 | 0.08384 | 0.0316 | 0.14010 | -0.09758 | 0.13484 | -0.00418 | 0.06305 | 0.05797 | -0.10749 | -0.01822 |
| VC_Q_AB_nCi_2_SS2_X_KA_h_MAS | 0.1207 | 0.8836 | 0.08972 | 0.0287 | 0.20731 | -0.02929 | 0.13764 | -0.01320 | 0.05773 | 0.07837 | -0.15778 | 0.01862 |
| VC_Q_AB_nCi_2_SS3_X_KA_h_MAS | 0.1325 | 0.9162 | 0.05150 | 0.0310 | 0.16783 | -0.01147 | 0.10837 | 0.00883 | 0.05008 | 0.02018 | -0.05242 | 0.01136 |
| VC_Q_AB_nCi_2_SS4_X_KA_h_MAS | 0.1332 | 0.8692 | 0.08459 | 0.0415 | 0.18121 | -0.06953 | 0.14436 | -0.01939 | 0.07809 | 0.08188 | -0.23192 | 0.02653 |
| VC_Q_AB_nCi_2_SS5_X_KA_h_MAS | 0.1296 | 0.9065 | 0.06625 | 0.0398 | 0.20164 | 0.01377 | 0.11611 | 0.01235 | 0.06029 | 0.00879 | -0.03061 | 0.03710 |
| VC_Q_AB_nCi_2_SS6_X_KA_h_MAS | 0.1391 | 0.8591 | 0.09054 | 0.0460 | 0.17247 | -0.08770 | 0.14761 | -0.01355 | 0.11736 | 0.07591 | -0.25077 | 0.03756 |
| PN_Q_AB_nCi_2_SS0_X_KA_v_MAS | -0.2649 | -0.3910 | -0.06059 | -0.0891 | -0.72482 | -0.16213 | -0.27442 | 0.06807 | -0.01437 | 0.01169 | 0.07178 | -0.04290 |
| PN_Q_AB_nCi_2_SS1_X_KA_v_MAS | -0.2373 | -0.5248 | -0.00857 | -0.0525 | -0.73155 | -0.00296 | -0.12475 | 0.06414 | 0.01749 | -0.02721 | 0.04847 | -0.14509 |
| PN_Q_AB_nCi_2_SS2_X_KA_v_MAS | -0.2549 | -0.4771 | -0.05729 | -0.0751 | -0.72878 | -0.12188 | -0.20299 | 0.07783 | 0.03871 | -0.00854 | 0.06403 | -0.09942 |
| PN_Q_AB_nCi_2_SS3_X_KA_v_MAS | -0.2391 | -0.4988 | -0.01713 | -0.0639 | -0.75418 | -0.06505 | -0.14240 | 0.06695 | 0.03741 | -0.01372 | 0.04780 | -0.13087 |
| PN_Q_AB_nCi_2_SS4_X_KA_v_MAS | -0.2457 | -0.4846 | -0.04022 | -0.0700 | -0.73706 | -0.11931 | -0.17561 | 0.07414 | 0.04904 | -0.01714 | 0.07082 | -0.11486 |
| PN_Q_AB_nCi_2_SS5_X_KA_v_MAS | -0.2368 | -0.4872 | -0.01857 | -0.0665 | -0.75971 | -0.09180 | -0.13679 | 0.06734 | 0.05444 | -0.01384 | 0.05002 | -0.12932 |
| PN_Q_AB_nCi_2_SS6_X_KA_v_MAS | -0.2400 | -0.4840 | -0.03461 | -0.0681 | -0.74264 | -0.12378 | -0.16122 | 0.07141 | 0.05492 | -0.02060 | 0.07255 | -0.11919 |
| P2_Q_AB_nCi_2_SS1_X_KA_v_MAS | -0.2126 | -0.0287 | 0.02715 | -0.0018 | -0.86420 | -0.14890 | -0.05505 | 0.00927 | -0.06140 | -0.09288 | 0.12930 | -0.11423 |
| P2_Q_AB_nCi_2_SS2_X_KA_v_MAS | -0.2407 | -0.1167 | 0.01926 | -0.0253 | -0.85532 | -0.17590 | -0.15541 | 0.04234 | 0.04556 | -0.06850 | 0.03364 | 0.00378 |
| P2_Q_AB_nCi_2_SS3_X_KA_v_MAS | -0.2168 | -0.2135 | 0.02814 | -0.0275 | -0.87010 | -0.13860 | -0.08071 | 0.04866 | 0.07393 | -0.08849 | 0.07837 | -0.12779 |
| P2_Q_AB_nCi_2_SS4_X_KA_v_MAS | -0.2355 | -0.2482 | 0.01863 | -0.0356 | -0.84250 | -0.16992 | -0.12348 | 0.06326 | 0.10027 | -0.07223 | 0.04906 | -0.06613 |
| P2_Q_AB_nCi_2_SS5_X_KA_v_MAS | -0.2147 | -0.2835 | 0.02204 | -0.0404 | -0.84709 | -0.14231 | -0.09482 | 0.05743 | 0.08904 | -0.07708 | 0.08694 | -0.13002 |
| P2_Q_AB_nCi_2_SS6_X_KA_v_MAS | -0.2306 | -0.3017 | 0.01819 | -0.0442 | -0.82585 | -0.17167 | -0.11463 | 0.06374 | 0.10405 | -0.06811 | 0.06658 | -0.08222 |
| P3_Q_AB_nCi_2_SS1_X_KA_v_MAS | -0.1729 | -0.0158 | 0.02536 | 0.0107 | -0.85500 | -0.13713 | -0.02352 | 0.00150 | -0.07465 | -0.10271 | 0.16188 | -0.15532 |
| P3_Q_AB_nCi_2_SS2_X_KA_v_MAS | -0.2066 | -0.0730 | 0.01203 | -0.0045 | -0.86939 | -0.17261 | -0.15220 | 0.02620 | 0.00672 | -0.08385 | 0.08830 | -0.01924 |
| P3_Q_AB_nCi_2_SS3_X_KA_v_MAS | -0.1811 | -0.1362 | 0.02710 | -0.0113 | -0.87785 | -0.13560 | -0.06339 | 0.02993 | 0.03294 | -0.10020 | 0.14030 | -0.15168 |
| P3_Q_AB_nCi_2_SS4_X_KA_v_MAS | -0.2046 | -0.1699 | 0.02184 | -0.0145 | -0.86756 | -0.17211 | -0.10567 | 0.04330 | 0.06234 | -0.08573 | 0.09834 | -0.07922 |
| P3_Q_AB_nCi_2_SS5_X_KA_v_MAS | -0.1803 | -0.1901 | 0.02467 | -0.0222 | -0.86221 | -0.14268 | -0.07530 | 0.03838 | 0.05688 | -0.09164 | 0.14705 | -0.14823 |
| P3_Q_AB_nCi_2_SS6_X_KA_v_MAS | -0.2003 | -0.2139 | 0.02328 | -0.0229 | -0.85450 | -0.17470 | -0.09282 | 0.04547 | 0.07612 | -0.08176 | 0.11065 | -0.09449 |
| HM_Q_AB_nCi_2_SS1_X_KA_v_MAS | -0.3404 | -0.0804 | -0.00180 | -0.0588 | -0.72589 | -0.19908 | -0.11680 | 0.08943 | 0.06367 | -0.01721 | -0.06000 | 0.01678 |
| HM_Q_AB_nCi_2_SS2_X_KA_v_MAS | -0.3447 | -0.2458 | 0.00383 | -0.1011 | -0.53640 | -0.14266 | -0.08202 | 0.13186 | 0.25719 | 0.01866 | -0.10844 | -0.07211 |
| HM_Q_AB_nCi_2_SS3_X_KA_v_MAS | -0.3395 | -0.4307 | 0.02672 | -0.1004 | -0.56152 | -0.06570 | -0.09859 | 0.12521 | 0.21388 | -0.00956 | -0.05044 | -0.12316 |
| HM_Q_AB_nCi_2_SS4_X_KA_v_MAS | -0.3398 | -0.3797 | -0.06742 | -0.1317 | -0.49955 | -0.06603 | -0.16250 | 0.14232 | 0.16974 | 0.01811 | 0.07724 | -0.14531 |
| HM_Q_AB_nCi_2_SS5_X_KA_v_MAS | -0.3638 | -0.4653 | -0.04307 | -0.1468 | -0.55433 | -0.02487 | -0.14521 | 0.10829 | 0.10726 | 0.04446 | 0.01841 | -0.13803 |
| HM_Q_AB_nCi_2_SS6_X_KA_v_MAS | -0.3603 | -0.4310 | -0.09213 | -0.1554 | -0.49912 | -0.06614 | -0.15186 | 0.10490 | 0.07652 | 0.04205 | 0.12697 | -0.15274 |
| S_Q_AB_nCi_2_SS3_X_KA_v_MAS | 0.0582 | 0.8022 | 0.06425 | 0.0393 | -0.13484 | 0.00384 | 0.12174 | -0.03925 | 0.00940 | -0.06700 | 0.20207 | -0.09354 |
| S_Q_AB_nCi_2_SS5_X_KA_v_MAS | 0.0585 | 0.7954 | 0.03909 | 0.0176 | -0.14931 | 0.03145 | 0.10582 | -0.02486 | -0.00864 | -0.05499 | 0.22852 | -0.09376 |
| SD_Q_AB_nCi_2_SS1_X_KA_v_MAS | -0.1533 | 0.0119 | 0.05258 | 0.0219 | -0.80061 | -0.14294 | -0.00092 | -0.02959 | -0.10509 | -0.10209 | 0.19149 | -0.18345 |
| SD_Q_AB_nCi_2_SS2_X_KA_v_MAS | -0.2011 | -0.1326 | 0.01299 | -0.0005 | -0.84077 | -0.17497 | -0.17045 | 0.00654 | -0.04615 | -0.08252 | 0.14147 | -0.02397 |
| SD_Q_AB_nCi_2_SS3_X_KA_v_MAS | -0.1306 | -0.0558 | 0.03528 | 0.0103 | -0.82582 | -0.13139 | -0.06695 | -0.01665 | -0.06163 | -0.10850 | 0.21462 | -0.14478 |
| SD_Q_AB_nCi_2_SS4_X_KA_v_MAS | -0.1813 | -0.1545 | 0.04575 | 0.0085 | -0.84639 | -0.16872 | -0.10199 | 0.00088 | -0.01744 | -0.08603 | 0.13266 | -0.04580 |
| SD_Q_AB_nCi_2_SS5_X_KA_v_MAS | -0.1172 | -0.0740 | 0.04666 | 0.0156 | -0.82275 | -0.14062 | -0.06288 | -0.01474 | -0.01566 | -0.11188 | 0.21769 | -0.12721 |
| SD_Q_AB_nCi_2_SS6_X_KA_v_MAS | -0.1675 | -0.1528 | 0.05695 | 0.0126 | -0.84248 | -0.16892 | -0.07817 | 0.00247 | 0.01896 | -0.08886 | 0.13298 | -0.05480 |
| VC_Q_AB_nCi_2_SS0_X_KA_v_MAS | 0.0516 | 0.9228 | 0.11768 | 0.0453 | 0.16323 | 0.01706 | 0.06398 | -0.02346 | 0.01419 | 0.00084 | 0.04394 | 0.07423 |
| VC_Q_AB_nCi_2_SS1_X_KA_v_MAS | 0.0055 | 0.9153 | 0.09346 | 0.0028 | 0.06116 | -0.16186 | 0.18737 | -0.00726 | 0.05098 | 0.00920 | -0.01341 | -0.02635 |
| VC_Q_AB_nCi_2_SS2_X_KA_v_MAS | 0.0188 | 0.9303 | 0.11323 | 0.0391 | 0.00768 | -0.02708 | 0.14790 | -0.01281 | 0.02477 | -0.03277 | -0.01925 | 0.04664 |
| VC_Q_AB_nCi_2_SS3_X_KA_v_MAS | 0.0406 | 0.9381 | 0.08278 | 0.0272 | 0.02440 | -0.07317 | 0.16064 | -0.01120 | 0.02771 | -0.03401 | 0.02782 | -0.00854 |
| VC_Q_AB_nCi_2_SS4_X_KA_v_MAS | 0.0390 | 0.9262 | 0.11495 | 0.0485 | 0.00991 | -0.03014 | 0.17709 | -0.02123 | 0.03963 | -0.01917 | -0.03860 | 0.05452 |
| VC_Q_AB_nCi_2_SS5_X_KA_v_MAS | 0.0580 | 0.9374 | 0.09281 | 0.0437 | 0.03626 | -0.04486 | 0.16097 | -0.01659 | 0.03516 | -0.04089 | 0.04453 | 0.01155 |
| VC_Q_AB_nCi_2_SS6_X_KA_v_MAS | 0.0496 | 0.9245 | 0.12071 | 0.0520 | 0.01289 | -0.03314 | 0.18072 | -0.01790 | 0.07069 | -0.01481 | -0.04164 | 0.05897 |
| S_Q_AB_nCi_2_MP0_X_KA_a_MAS | 0.2952 | 0.5441 | 0.09240 | 0.0483 | 0.21671 | 0.01932 | 0.15763 | -0.04660 | -0.14291 | 0.02353 | -0.06052 | -0.01785 |
| S_Q_AB_nCi_2_MP1_X_KA_a_MAS | 0.1077 | 0.0973 | 0.02601 | 0.0034 | 0.19518 | 0.05718 | 0.54876 | -0.01060 | -0.06379 | 0.05884 | 0.10636 | 0.05619 |
| S_Q_AB_nCi_2_MP2_X_KA_a_MAS | 0.1570 | 0.1511 | 0.04349 | 0.0260 | -0.08200 | 0.03416 | 0.52232 | -0.03963 | -0.11954 | -0.01091 | -0.01360 | 0.04046 |
| S_Q_AB_nCi_2_MP3_X_KA_a_MAS | 0.0867 | 0.1556 | 0.03956 | 0.0107 | 0.14943 | -0.02926 | 0.64937 | 0.00091 | -0.07514 | 0.05758 | 0.08391 | 0.11578 |
| S_Q_AB_nCi_2_MP4_X_KA_a_MAS | 0.1414 | 0.1189 | 0.02823 | 0.0074 | -0.12957 | -0.03994 | 0.62640 | -0.02555 | -0.05841 | -0.02212 | -0.03795 | 0.03776 |
| S_Q_AB_nCi_2_MP5_X_KA_a_MAS | 0.0987 | 0.1417 | 0.02347 | 0.0203 | 0.12836 | -0.04975 | 0.68799 | 0.00883 | -0.07035 | 0.05437 | 0.06849 | 0.11720 |
| S_Q_AB_nCi_2_MP6_X_KA_a_MAS | 0.1249 | 0.0951 | 0.02032 | 0.0049 | -0.12614 | -0.06633 | 0.66211 | -0.01049 | -0.04219 | -0.01887 | -0.03230 | 0.02849 |
| VC_Q_AB_nCi_2_MP0_X_KA_a_MAS | 0.2639 | 0.5926 | 0.10677 | 0.0566 | 0.20855 | 0.01146 | 0.20145 | -0.06660 | -0.11535 | 0.02186 | -0.06693 | 0.00406 |
| S_Q_AB_nCi_2_MP2_X_KA_h_MAS | 0.2347 | 0.7186 | 0.09778 | 0.0727 | 0.33342 | -0.04464 | 0.03813 | 0.00966 | 0.07439 | 0.12925 | -0.17709 | -0.03403 |
| S_Q_AB_nCi_2_MP3_X_KA_h_MAS | 0.1444 | 0.8438 | 0.02318 | 0.0496 | 0.01026 | 0.06158 | 0.00925 | 0.02672 | 0.11762 | 0.00840 | 0.10123 | -0.07388 |
| S_Q_AB_nCi_2_MP4_X_KA_h_MAS | 0.2145 | 0.7600 | 0.06053 | 0.0673 | 0.30259 | 0.01976 | 0.00413 | 0.04321 | 0.17279 | 0.10021 | -0.10749 | -0.07316 |
| S_Q_AB_nCi_2_MP5_X_KA_h_MAS | 0.1605 | 0.8198 | -0.00723 | 0.0550 | 0.03933 | 0.15026 | -0.01454 | 0.04167 | 0.10706 | 0.00746 | 0.16007 | -0.07477 |
| S_Q_AB_nCi_2_MP6_X_KA_h_MAS | 0.2114 | 0.7622 | 0.03490 | 0.0618 | 0.27556 | 0.07101 | -0.00773 | 0.05879 | 0.20924 | 0.08614 | -0.03087 | -0.07969 |
| VC_Q_AB_nCi_2_MP0_X_KA_h_MAS | 0.1243 | 0.8877 | 0.11091 | 0.0279 | 0.24225 | 0.03726 | 0.12770 | -0.01433 | 0.07709 | 0.07795 | 0.00281 | 0.09060 |
| VC_Q_AB_nCi_2_MP1_X_KA_h_MAS | 0.0827 | 0.9041 | 0.05763 | 0.0139 | 0.10847 | -0.17605 | 0.11422 | -0.00185 | 0.09269 | 0.08449 | -0.13634 | -0.04239 |
| VC_Q_AB_nCi_2_MP2_X_KA_h_MAS | 0.1299 | 0.8566 | 0.09661 | 0.0408 | 0.19679 | -0.09290 | 0.12848 | -0.01462 | 0.10921 | 0.09577 | -0.21391 | -0.01894 |
| VC_Q_AB_nCi_2_MP3_X_KA_h_MAS | 0.1134 | 0.9230 | 0.05285 | 0.0308 | 0.06986 | -0.06199 | 0.11456 | 0.00522 | 0.13286 | 0.03968 | -0.07993 | -0.04040 |
| VC_Q_AB_nCi_2_MP4_X_KA_h_MAS | 0.1475 | 0.8406 | 0.08053 | 0.0517 | 0.15456 | -0.08131 | 0.13958 | -0.01312 | 0.19067 | 0.09114 | -0.23110 | -0.03311 |
| VC_Q_AB_nCi_2_MP5_X_KA_h_MAS | 0.1399 | 0.9134 | 0.05830 | 0.0610 | 0.08761 | 0.02530 | 0.10903 | 0.00557 | 0.15847 | 0.00542 | -0.02282 | -0.03440 |
| VC_Q_AB_nCi_2_MP6_X_KA_h_MAS | 0.1634 | 0.8311 | 0.07411 | 0.0625 | 0.13218 | -0.04258 | 0.13457 | 0.00223 | 0.26596 | 0.07329 | -0.19529 | -0.04017 |
| S_Q_AB_nCi_2_MP1_X_KA_v_MAS | 0.1127 | 0.6814 | -0.04839 | 0.0346 | -0.16809 | -0.02830 | 0.20236 | -0.05545 | -0.00978 | -0.06452 | 0.10410 | -0.14809 |
| S_Q_AB_nCi_2_MP2_X_KA_v_MAS | 0.1252 | 0.8224 | -0.03116 | 0.0778 | -0.04724 | 0.02461 | 0.19311 | -0.03709 | -0.02404 | -0.04620 | 0.13337 | -0.10095 |
| S_Q_AB_nCi_2_MP3_X_KA_v_MAS | 0.0883 | 0.7944 | -0.07638 | 0.0464 | -0.11955 | 0.08600 | 0.11809 | -0.01673 | 0.02293 | -0.08501 | 0.20841 | -0.13532 |
| S_Q_AB_nCi_2_MP4_X_KA_v_MAS | 0.1222 | 0.8087 | -0.09539 | 0.0701 | -0.09678 | 0.05600 | 0.14488 | 0.01710 | 0.04584 | -0.05780 | 0.14092 | -0.12307 |
| S_Q_AB_nCi_2_MP5_X_KA_v_MAS | 0.1348 | 0.7510 | -0.11024 | 0.0548 | -0.11091 | 0.12996 | 0.07670 | 0.01362 | -0.01034 | -0.08336 | 0.20466 | -0.12596 |
| S_Q_AB_nCi_2_MP6_X_KA_v_MAS | 0.1561 | 0.7694 | -0.12361 | 0.0722 | -0.08878 | 0.07412 | 0.11944 | 0.03942 | 0.04258 | -0.05321 | 0.14476 | -0.12697 |
| VC_Q_AB_nCi_2_MP0_X_KA_v_MAS | 0.0516 | 0.9228 | 0.11768 | 0.0453 | 0.16323 | 0.01706 | 0.06398 | -0.02346 | 0.01419 | 0.00084 | 0.04394 | 0.07423 |
| VC_Q_AB_nCi_2_MP1_X_KA_v_MAS | 0.0146 | 0.9073 | 0.06204 | -0.0083 | 0.04549 | -0.17031 | 0.20526 | -0.00383 | 0.06964 | 0.01312 | -0.00984 | -0.06260 |
| VC_Q_AB_nCi_2_MP2_X_KA_v_MAS | 0.0465 | 0.9225 | 0.06843 | 0.0382 | -0.06471 | -0.03242 | 0.20783 | -0.00897 | 0.05686 | -0.03637 | -0.01138 | -0.02065 |
| VC_Q_AB_nCi_2_MP3_X_KA_v_MAS | 0.0706 | 0.9214 | 0.04311 | 0.0299 | -0.05571 | -0.05305 | 0.18875 | -0.00566 | 0.07166 | -0.03975 | 0.02840 | -0.07574 |
| VC_Q_AB_nCi_2_MP4_X_KA_v_MAS | 0.0847 | 0.9064 | 0.05058 | 0.0553 | -0.09758 | -0.00697 | 0.21704 | -0.00829 | 0.09294 | -0.03814 | -0.02080 | -0.04032 |
| VC_Q_AB_nCi_2_MP5_X_KA_v_MAS | 0.1132 | 0.9082 | 0.03394 | 0.0662 | -0.06693 | 0.01752 | 0.16787 | -0.00289 | 0.08758 | -0.07028 | 0.05449 | -0.07390 |
| VC_Q_AB_nCi_2_MP6_X_KA_v_MAS | 0.1209 | 0.8877 | 0.04117 | 0.0755 | -0.10346 | 0.02561 | 0.20038 | 0.00751 | 0.13945 | -0.05344 | -0.01333 | -0.05246 |
| MIC_N1_F_AB_nCi_2_NS0_T_KA_e_MAS | -0.0736 | -0.5812 | -0.16493 | -0.0100 | -0.64616 | -0.03106 | -0.14937 | 0.00800 | -0.04736 | -0.06827 | -0.08494 | -0.23984 |
| MIC_PN_F_AB_nCi_2_NS0_T_KA_e_MAS | -0.1647 | -0.8130 | -0.11994 | -0.0861 | -0.12570 | -0.01051 | 0.00491 | 0.08285 | -0.00470 | 0.06581 | -0.11716 | -0.20609 |
| MIC_N1_F_AB_nCi_2_NS1_T_KA_e_MAS | 0.4526 | -0.3371 | 0.02228 | 0.1896 | -0.36328 | 0.14090 | -0.06761 | -0.11365 | -0.25829 | 0.06098 | -0.09426 | -0.31250 |
| MIC_PN_F_AB_nCi_2_NS1_T_KA_e_MAS | -0.6639 | 0.0217 | 0.16021 | -0.2643 | 0.29306 | -0.11320 | 0.02961 | 0.00741 | 0.07565 | 0.11134 | 0.10082 | 0.07462 |
| MIC_N1_F_AB_nCi_2_NS2_T_KA_e_MAS | 0.7987 | -0.0918 | -0.06351 | 0.2410 | -0.00970 | 0.09817 | -0.04656 | -0.04211 | -0.16739 | 0.11483 | -0.14879 | -0.10625 |
| MIC_PN_F_AB_nCi_2_NS2_T_KA_e_MAS | -0.8969 | -0.0397 | 0.03858 | -0.2221 | -0.04025 | -0.07312 | -0.00186 | 0.00316 | 0.07058 | -0.02810 | 0.10678 | 0.08799 |
| MIC_N1_F_AB_nCi_2_NS3_T_KA_e_MAS | 0.8818 | 0.0017 | -0.02541 | 0.1897 | 0.10503 | 0.05012 | -0.00323 | 0.06278 | -0.05607 | 0.13902 | -0.11161 | -0.08444 |
| MIC_PN_F_AB_nCi_2_NS3_T_KA_e_MAS | -0.9207 | -0.0786 | 0.01039 | -0.1912 | -0.10662 | -0.03984 | -0.01689 | -0.03280 | 0.03417 | -0.04235 | 0.08132 | 0.06867 |
| MIC_N1_F_AB_nCi_2_NS4_T_KA_e_MAS | 0.8981 | 0.0183 | -0.01771 | 0.1742 | 0.12660 | 0.02929 | 0.00873 | 0.10901 | -0.01098 | 0.11850 | -0.10431 | -0.08046 |
| MIC_PN_F_AB_nCi_2_NS4_T_KA_e_MAS | -0.9277 | -0.0811 | 0.00906 | -0.1753 | -0.11719 | -0.02619 | -0.02325 | -0.04863 | 0.01564 | -0.03519 | 0.07452 | 0.06733 |
| MIC_N1_F_AB_nCi_2_NS5_T_KA_e_MAS | 0.8991 | 0.0356 | -0.01976 | 0.1673 | 0.14121 | 0.03059 | 0.01476 | 0.13492 | 0.00780 | 0.09292 | -0.10508 | -0.07445 |
| MIC_PN_F_AB_nCi_2_NS5_T_KA_e_MAS | -0.9275 | -0.0868 | 0.01259 | -0.1676 | -0.12819 | -0.03052 | -0.02428 | -0.05711 | 0.00787 | -0.02845 | 0.07912 | 0.06104 |
| MIC_N1_F_AB_nCi_2_NS6_T_KA_e_MAS | 0.9014 | 0.0392 | -0.01049 | 0.1701 | 0.14167 | 0.02181 | 0.01942 | 0.15232 | 0.02666 | 0.07460 | -0.09821 | -0.06809 |
| MIC_PN_F_AB_nCi_2_NS6_T_KA_e_MAS | -0.9286 | -0.0858 | 0.00864 | -0.1710 | -0.12855 | -0.02056 | -0.02560 | -0.06402 | -0.00178 | -0.02075 | 0.07010 | 0.05493 |
| TIC_F_AB_nCi_2_NS1_T_KA_e_MAS | 0.7980 | -0.0911 | 0.04706 | 0.1101 | -0.06502 | 0.04730 | 0.00319 | 0.53835 | -0.02416 | 0.03397 | -0.01596 | -0.05967 |
| TIC_F_AB_nCi_2_NS2_T_KA_e_MAS | 0.8000 | -0.0356 | 0.03406 | 0.1037 | 0.01707 | 0.03383 | 0.00512 | 0.55505 | 0.01847 | 0.06505 | -0.02824 | -0.01050 |
| TIC_F_AB_nCi_2_NS3_T_KA_e_MAS | 0.7852 | -0.0146 | 0.04297 | 0.0848 | 0.04205 | 0.02345 | 0.01441 | 0.58409 | 0.04879 | 0.07474 | -0.01929 | -0.00772 |
| TIC_F_AB_nCi_2_NS4_T_KA_e_MAS | 0.7749 | -0.0109 | 0.04312 | 0.0794 | 0.04600 | 0.01825 | 0.01780 | 0.60255 | 0.05703 | 0.06625 | -0.01686 | -0.00674 |
| TIC_F_AB_nCi_2_NS5_T_KA_e_MAS | 0.7676 | -0.0071 | 0.04259 | 0.0768 | 0.04742 | 0.01758 | 0.01904 | 0.61458 | 0.05957 | 0.05794 | -0.01605 | -0.00541 |
| TIC_F_AB_nCi_2_NS6_T_KA_e_MAS | 0.7635 | -0.0056 | 0.04387 | 0.0766 | 0.04610 | 0.01555 | 0.02060 | 0.62135 | 0.06230 | 0.05146 | -0.01466 | -0.00429 |
| SIC_F_AB_nCi_2_NS0_T_KA_e_MAS | -0.4426 | -0.5224 | -0.12494 | -0.0661 | -0.58202 | -0.02777 | -0.12719 | -0.00187 | -0.01194 | -0.00997 | -0.09822 | -0.20954 |
| SIC_F_AB_nCi_2_NS1_T_KA_e_MAS | -0.3730 | -0.3909 | 0.00616 | 0.0728 | -0.40384 | 0.13608 | -0.07528 | -0.19729 | -0.24201 | 0.10698 | -0.11852 | -0.31203 |
| SIC_F_AB_nCi_2_NS2_T_KA_e_MAS | -0.1163 | -0.2456 | -0.14215 | 0.1967 | -0.10635 | 0.15065 | -0.08880 | -0.29504 | -0.29046 | 0.20471 | -0.26146 | -0.17653 |
| ES_N1_F_AB_nCi_2_NS0_T_KA_e_MAS | 0.7955 | -0.0229 | 0.03478 | 0.0659 | 0.02957 | 0.00635 | 0.02044 | 0.59424 | 0.05218 | 0.01080 | 0.00455 | -0.01886 |
| ES_PN_F_AB_nCi_2_NS0_T_KA_e_MAS | -0.0488 | -0.7559 | -0.20262 | -0.0879 | -0.13528 | -0.01841 | -0.03350 | 0.09654 | 0.04913 | 0.02187 | -0.14898 | -0.47781 |
| ES_N1_F_AB_nCi_2_NS1_T_KA_e_MAS | 0.8171 | -0.0067 | -0.00362 | 0.0797 | 0.02077 | 0.00053 | 0.03446 | 0.55027 | -0.09917 | 0.01077 | -0.00139 | 0.02404 |
| ES_PN_F_AB_nCi_2_NS1_T_KA_e_MAS | 0.1482 | -0.1235 | -0.36990 | 0.0322 | -0.02693 | -0.03888 | 0.04373 | -0.19119 | -0.80899 | 0.06616 | -0.03116 | 0.09042 |
| ES_N1_F_AB_nCi_2_NS2_T_KA_e_MAS | 0.8039 | -0.0252 | -0.02109 | 0.0744 | 0.00254 | -0.03038 | 0.04218 | 0.51264 | -0.22852 | 0.02275 | 0.00129 | 0.01121 |
| ES_PN_F_AB_nCi_2_NS2_T_KA_e_MAS | 0.1188 | -0.1246 | -0.29514 | 0.0020 | -0.06083 | -0.12719 | 0.04063 | -0.18116 | -0.84477 | 0.10413 | 0.01520 | -0.01306 |
| ES_N1_F_AB_nCi_2_NS3_T_KA_e_MAS | 0.7775 | -0.0311 | -0.03983 | 0.0741 | 0.01402 | -0.01891 | 0.03807 | 0.44394 | -0.35768 | 0.01938 | 0.02087 | 0.02255 |
| ES_PN_F_AB_nCi_2_NS3_T_KA_e_MAS | 0.1223 | -0.0877 | -0.26573 | 0.0063 | 0.00215 | -0.06927 | 0.01142 | -0.19283 | -0.86727 | 0.08653 | 0.06667 | 0.02412 |
| ES_N1_F_AB_nCi_2_NS4_T_KA_e_MAS | 0.7333 | -0.0427 | -0.05133 | 0.0726 | -0.00729 | -0.02909 | 0.04545 | 0.39038 | -0.44830 | 0.00826 | 0.03443 | 0.00872 |
| ES_PN_F_AB_nCi_2_NS4_T_KA_e_MAS | 0.1081 | -0.0904 | -0.24207 | 0.0057 | -0.03154 | -0.07953 | 0.02197 | -0.19020 | -0.86319 | 0.06728 | 0.08703 | -0.01169 |
| ES_N1_F_AB_nCi_2_NS5_T_KA_e_MAS | 0.6863 | -0.0467 | -0.06219 | 0.0699 | 0.00337 | -0.01188 | 0.03986 | 0.32456 | -0.52404 | -0.00362 | 0.05368 | 0.01393 |
| ES_PN_F_AB_nCi_2_NS5_T_KA_e_MAS | 0.1110 | -0.0773 | -0.22987 | 0.0074 | 0.00039 | -0.04503 | 0.00783 | -0.19369 | -0.86284 | 0.04981 | 0.10952 | 0.00617 |
| ES_N1_F_AB_nCi_2_NS6_T_KA_e_MAS | 0.6377 | -0.0527 | -0.07076 | 0.0682 | -0.01573 | -0.01485 | 0.04641 | 0.28088 | -0.56933 | -0.01822 | 0.06871 | 0.00049 |
| ES_PN_F_AB_nCi_2_NS6_T_KA_e_MAS | 0.1044 | -0.0782 | -0.21969 | 0.0078 | -0.02575 | -0.04885 | 0.01808 | -0.18992 | -0.85020 | 0.03342 | 0.12490 | -0.01545 |
| IB_PN_F_AB_nCi_2_NS0_T_KA_e_MAS | 0.8022 | 0.0708 | 0.01872 | 0.0744 | 0.03727 | -0.01836 | 0.03152 | 0.55957 | -0.10256 | -0.04934 | 0.01062 | 0.03502 |
| IB_PN_F_AB_nCi_2_NS1_T_KA_e_MAS | 0.7344 | 0.0717 | 0.10202 | 0.0666 | 0.04016 | 0.02225 | 0.01581 | 0.60476 | 0.20138 | -0.08175 | 0.02885 | 0.00059 |
| IB_PN_F_AB_nCi_2_NS2_T_KA_e_MAS | 0.5865 | 0.0867 | 0.14676 | 0.0898 | 0.00370 | 0.10179 | 0.01466 | 0.55057 | 0.39810 | -0.21660 | 0.05688 | 0.00966 |
| IB_PN_F_AB_nCi_2_NS3_T_KA_e_MAS | 0.4077 | 0.0974 | 0.13326 | 0.0954 | -0.03564 | 0.13470 | 0.00447 | 0.47391 | 0.51250 | -0.31844 | 0.06726 | 0.02448 |
| MIC_N1_F_AB_nCi_2_NS0_M_KA_e_MAS | 0.1025 | 0.1744 | 0.88345 | 0.2651 | 0.08038 | -0.04131 | 0.07452 | 0.01819 | 0.10272 | 0.12983 | 0.01348 | -0.06771 |
| MIC_PN_F_AB_nCi_2_NS0_M_KA_e_MAS | 0.1025 | 0.1744 | 0.88345 | 0.2651 | 0.08038 | -0.04131 | 0.07452 | 0.01819 | 0.10272 | 0.12983 | 0.01348 | -0.06771 |
| MIC_N1_F_AB_nCi_2_NS1_M_KA_e_MAS | 0.1183 | 0.1379 | 0.87225 | 0.2856 | 0.07441 | 0.03095 | 0.07918 | 0.00794 | 0.03836 | 0.12354 | 0.02485 | -0.08641 |
| MIC_PN_F_AB_nCi_2_NS1_M_KA_e_MAS | 0.0605 | 0.1522 | 0.78547 | 0.4122 | 0.08724 | -0.09213 | 0.05901 | -0.02210 | 0.12479 | 0.12063 | 0.01709 | -0.05329 |
| MIC_N1_F_AB_nCi_2_NS2_M_KA_e_MAS | 0.1822 | 0.2067 | 0.78509 | 0.2231 | 0.11249 | -0.12311 | 0.09686 | 0.04416 | 0.14427 | 0.23890 | -0.03952 | -0.06659 |
| MIC_PN_F_AB_nCi_2_NS2_M_KA_e_MAS | 0.0205 | 0.1351 | 0.76613 | 0.4568 | 0.08398 | -0.05329 | 0.06063 | -0.03774 | 0.07660 | 0.14409 | 0.03452 | -0.04576 |
| MIC_N1_F_AB_nCi_2_NS3_M_KA_e_MAS | 0.2393 | 0.1834 | 0.75539 | 0.2749 | 0.12694 | -0.09874 | 0.08889 | 0.03271 | 0.10506 | 0.27377 | -0.03361 | -0.06832 |
| MIC_PN_F_AB_nCi_2_NS3_M_KA_e_MAS | -0.0368 | 0.0750 | 0.69646 | 0.5848 | 0.07261 | 0.00824 | 0.05565 | -0.07933 | -0.00629 | 0.05847 | 0.06021 | -0.03998 |
| MIC_N1_F_AB_nCi_2_NS4_M_KA_e_MAS | 0.2687 | 0.1569 | 0.70984 | 0.2999 | 0.15511 | -0.09997 | 0.09207 | 0.02824 | 0.06967 | 0.28558 | -0.02882 | -0.07549 |
| MIC_PN_F_AB_nCi_2_NS4_M_KA_e_MAS | -0.0946 | 0.0567 | 0.63512 | 0.6617 | 0.04957 | 0.01728 | 0.04966 | -0.07603 | -0.03232 | 0.04609 | 0.06592 | -0.03080 |
| MIC_N1_F_AB_nCi_2_NS5_M_KA_e_MAS | 0.3078 | 0.1323 | 0.68388 | 0.3623 | 0.15467 | -0.06550 | 0.08533 | 0.01686 | 0.03060 | 0.27729 | -0.02582 | -0.07436 |
| MIC_PN_F_AB_nCi_2_NS5_M_KA_e_MAS | -0.1339 | 0.0716 | 0.60168 | 0.6983 | 0.01515 | 0.01140 | 0.03745 | -0.08290 | -0.00558 | 0.01509 | 0.06455 | -0.01897 |
| MIC_N1_F_AB_nCi_2_NS6_M_KA_e_MAS | 0.3254 | 0.1156 | 0.63889 | 0.4173 | 0.15204 | -0.05786 | 0.08170 | 0.01809 | 0.00447 | 0.27237 | -0.02354 | -0.07514 |
| MIC_PN_F_AB_nCi_2_NS6_M_KA_e_MAS | -0.1607 | 0.0844 | 0.60193 | 0.6989 | -0.00420 | 0.01559 | 0.02913 | -0.08537 | -0.00077 | 0.00075 | 0.06481 | -0.01516 |
| SIC_F_AB_nCi_2_NS2_M_KA_e_MAS | -0.0190 | 0.1734 | 0.81059 | 0.2410 | 0.08984 | -0.10878 | 0.09507 | 0.00218 | 0.13029 | 0.21367 | -0.02244 | -0.06315 |
| SIC_F_AB_nCi_2_NS3_M_KA_e_MAS | 0.0400 | 0.1560 | 0.79422 | 0.3007 | 0.10567 | -0.08771 | 0.08675 | -0.01522 | 0.08795 | 0.25075 | -0.01639 | -0.06985 |
| SIC_F_AB_nCi_2_NS4_M_KA_e_MAS | 0.0765 | 0.1339 | 0.75095 | 0.3293 | 0.13996 | -0.09113 | 0.09230 | -0.02363 | 0.05085 | 0.26604 | -0.01430 | -0.07775 |
| SIC_F_AB_nCi_2_NS5_M_KA_e_MAS | 0.1203 | 0.1136 | 0.72942 | 0.3983 | 0.14264 | -0.05725 | 0.08590 | -0.04135 | 0.00889 | 0.25961 | -0.01142 | -0.08001 |
| SIC_F_AB_nCi_2_NS6_M_KA_e_MAS | 0.1406 | 0.0995 | 0.68370 | 0.4563 | 0.14333 | -0.05124 | 0.08213 | -0.04453 | -0.01832 | 0.25662 | -0.01173 | -0.08076 |
| ES_PN_F_AB_nCi_2_NS0_M_KA_e_MAS | 0.0348 | 0.1793 | 0.90672 | 0.0757 | 0.06221 | -0.04554 | 0.08299 | 0.03632 | 0.12763 | 0.11984 | 0.03016 | -0.06090 |
| ES_PN_F_AB_nCi_2_NS1_M_KA_e_MAS | 0.0092 | 0.1685 | 0.89649 | 0.0897 | 0.07571 | -0.05074 | 0.07438 | 0.03455 | 0.11573 | 0.09583 | 0.04402 | -0.05092 |
| ES_PN_F_AB_nCi_2_NS3_M_KA_e_MAS | 0.0565 | 0.2096 | 0.77460 | 0.0505 | 0.11010 | -0.18566 | 0.09174 | 0.02939 | 0.10667 | 0.23894 | -0.01821 | -0.05359 |
| ES_N1_F_AB_nCi_2_NS4_M_KA_e_MAS | 0.4164 | 0.2032 | 0.57469 | -0.0250 | 0.13167 | -0.20555 | 0.08774 | 0.25068 | 0.10859 | 0.26684 | -0.08586 | -0.04215 |
| ES_PN_F_AB_nCi_2_NS5_M_KA_e_MAS | 0.0753 | 0.1696 | 0.69793 | 0.0479 | 0.12746 | -0.21048 | 0.08868 | 0.00089 | 0.01428 | 0.26194 | -0.02648 | -0.07250 |
| MIC_N1_F_AB_nCi_2_NS0_X_KA_e_MAS | -0.0738 | -0.5808 | -0.16567 | -0.0106 | -0.64668 | -0.03154 | -0.14854 | 0.00793 | -0.04777 | -0.06812 | -0.08442 | -0.23899 |
| MIC_PN_F_AB_nCi_2_NS0_X_KA_e_MAS | -0.1648 | -0.8130 | -0.12019 | -0.0863 | -0.12585 | -0.01067 | 0.00520 | 0.08284 | -0.00484 | 0.06588 | -0.11700 | -0.20583 |
| MIC_N1_F_AB_nCi_2_NS1_X_KA_e_MAS | 0.1490 | -0.6097 | -0.16296 | 0.0836 | -0.54461 | 0.10064 | -0.11593 | -0.03546 | -0.14804 | -0.06481 | -0.11618 | -0.19373 |
| MIC_PN_F_AB_nCi_2_NS1_X_KA_e_MAS | -0.4123 | -0.5328 | 0.01030 | -0.0844 | 0.15289 | 0.22600 | -0.07680 | 0.03953 | -0.07179 | 0.04708 | 0.11245 | -0.02530 |
| MIC_N1_F_AB_nCi_2_NS2_X_KA_e_MAS | 0.2947 | -0.6387 | -0.17239 | 0.0965 | -0.39995 | 0.10276 | -0.15113 | -0.00513 | -0.16980 | -0.01481 | -0.08207 | -0.21287 |
| MIC_PN_F_AB_nCi_2_NS2_X_KA_e_MAS | -0.7308 | -0.1634 | -0.00383 | -0.1521 | 0.11521 | 0.07964 | -0.04386 | -0.04963 | -0.05894 | 0.02902 | 0.15583 | 0.05403 |
| MIC_N1_F_AB_nCi_2_NS3_X_KA_e_MAS | 0.4928 | -0.5611 | -0.16149 | 0.1470 | -0.22083 | 0.18705 | -0.13458 | -0.00617 | -0.22367 | 0.01836 | -0.06530 | -0.13161 |
| MIC_PN_F_AB_nCi_2_NS3_X_KA_e_MAS | -0.8380 | 0.0499 | 0.02640 | -0.1690 | 0.01668 | -0.01399 | 0.01617 | -0.03689 | 0.01956 | 0.03145 | 0.09097 | 0.05801 |
| MIC_N1_F_AB_nCi_2_NS4_X_KA_e_MAS | 0.5861 | -0.4941 | -0.15103 | 0.1634 | -0.09043 | 0.19366 | -0.11857 | 0.02408 | -0.23768 | 0.03442 | -0.01494 | -0.12815 |
| MIC_PN_F_AB_nCi_2_NS4_X_KA_e_MAS | -0.8770 | 0.0719 | 0.01470 | -0.1611 | -0.06036 | -0.02527 | 0.01456 | -0.04440 | 0.04783 | 0.00861 | 0.06274 | 0.05154 |
| MIC_N1_F_AB_nCi_2_NS5_X_KA_e_MAS | 0.7013 | -0.3793 | -0.11303 | 0.1831 | -0.01044 | 0.18283 | -0.07678 | 0.04962 | -0.17192 | 0.10476 | -0.04688 | -0.10706 |
| MIC_PN_F_AB_nCi_2_NS5_X_KA_e_MAS | -0.8992 | 0.0425 | -0.00275 | -0.1719 | -0.11256 | -0.02464 | 0.00373 | -0.04333 | 0.03088 | -0.00708 | 0.05858 | 0.04137 |
| MIC_N1_F_AB_nCi_2_NS6_X_KA_e_MAS | 0.7182 | -0.3337 | -0.10440 | 0.1867 | 0.02481 | 0.16538 | -0.07589 | 0.07343 | -0.16332 | 0.09817 | -0.02518 | -0.11552 |
| MIC_PN_F_AB_nCi_2_NS6_X_KA_e_MAS | -0.9045 | 0.0245 | 0.00032 | -0.1678 | -0.13125 | -0.00658 | 0.00587 | -0.05138 | 0.03144 | -0.01114 | 0.04524 | 0.03907 |
| TIC_F_AB_nCi_2_NS1_X_KA_e_MAS | 0.7095 | -0.2688 | -0.04111 | 0.1108 | -0.19159 | 0.06814 | -0.03117 | 0.54079 | -0.03721 | -0.03320 | -0.02234 | -0.05214 |
| TIC_F_AB_nCi_2_NS5_X_KA_e_MAS | 0.7561 | -0.1429 | 0.01006 | 0.0980 | 0.01393 | 0.07131 | -0.00944 | 0.58500 | 0.00263 | 0.06715 | 0.00111 | -0.01134 |
| TIC_F_AB_nCi_2_NS6_X_KA_e_MAS | 0.7506 | -0.1287 | 0.01161 | 0.0967 | 0.02214 | 0.06545 | -0.00879 | 0.59821 | 0.00561 | 0.06279 | 0.00731 | -0.01376 |
| SIC_F_AB_nCi_2_NS0_X_KA_e_MAS | -0.4428 | -0.5220 | -0.12556 | -0.0666 | -0.58241 | -0.02819 | -0.12646 | -0.00193 | -0.01229 | -0.00984 | -0.09776 | -0.20879 |
| SIC_F_AB_nCi_2_NS1_X_KA_e_MAS | -0.3399 | -0.5820 | -0.14570 | 0.0044 | -0.52977 | 0.08279 | -0.10791 | -0.07456 | -0.11687 | -0.01093 | -0.13805 | -0.19107 |
| SIC_F_AB_nCi_2_NS2_X_KA_e_MAS | -0.2192 | -0.6488 | -0.17288 | 0.0232 | -0.43224 | 0.09176 | -0.15099 | -0.07730 | -0.15762 | 0.02558 | -0.11172 | -0.22598 |
| SIC_F_AB_nCi_2_NS3_X_KA_e_MAS | -0.1034 | -0.6450 | -0.19370 | 0.0763 | -0.29028 | 0.19311 | -0.15347 | -0.12493 | -0.25215 | 0.05374 | -0.10653 | -0.16422 |
| SIC_F_AB_nCi_2_NS4_X_KA_e_MAS | -0.0157 | -0.6175 | -0.20104 | 0.1024 | -0.16130 | 0.21940 | -0.14829 | -0.12501 | -0.30115 | 0.07318 | -0.05620 | -0.17416 |
| SIC_F_AB_nCi_2_NS5_X_KA_e_MAS | 0.0758 | -0.5507 | -0.18485 | 0.1406 | -0.08087 | 0.23957 | -0.11397 | -0.13500 | -0.26649 | 0.16204 | -0.10656 | -0.16980 |
| ES_PN_F_AB_nCi_2_NS0_X_KA_e_MAS | -0.1660 | -0.7428 | -0.18101 | -0.0906 | -0.42336 | -0.03509 | -0.07118 | 0.10679 | 0.05038 | 0.00740 | -0.10930 | -0.35771 |
| ES_PN_F_AB_nCi_2_NS1_X_KA_e_MAS | -0.1161 | -0.5748 | -0.06818 | -0.0144 | -0.69045 | -0.01133 | 0.04266 | 0.01492 | -0.03544 | -0.00404 | -0.08515 | -0.23862 |
| ES_PN_F_AB_nCi_2_NS2_X_KA_e_MAS | -0.0944 | -0.5674 | -0.11699 | -0.0221 | -0.65469 | -0.15677 | 0.07208 | 0.01107 | -0.08830 | 0.02109 | -0.13478 | -0.26891 |
| ES_PN_F_AB_nCi_2_NS3_X_KA_e_MAS | -0.0542 | -0.5099 | -0.12953 | -0.0183 | -0.66975 | -0.11268 | 0.07293 | -0.03320 | -0.20157 | 0.04548 | -0.02813 | -0.23230 |
| ES_PN_F_AB_nCi_2_NS4_X_KA_e_MAS | -0.0476 | -0.4720 | -0.13387 | -0.0130 | -0.64612 | -0.17971 | 0.10454 | -0.04494 | -0.24443 | 0.04416 | -0.05315 | -0.24379 |
| ES_PN_F_AB_nCi_2_NS5_X_KA_e_MAS | -0.0144 | -0.4228 | -0.14777 | -0.0124 | -0.61285 | -0.13972 | 0.09560 | -0.07530 | -0.33114 | 0.05504 | 0.02172 | -0.21444 |
| ES_PN_F_AB_nCi_2_NS6_X_KA_e_MAS | -0.0122 | -0.3860 | -0.14657 | -0.0084 | -0.59806 | -0.17302 | 0.11764 | -0.08099 | -0.34532 | 0.04747 | 0.01403 | -0.22280 |
| IB_PN_F_AB_nCi_2_NS2_X_KA_e_MAS | 0.4047 | -0.1801 | -0.08501 | 0.0496 | -0.01079 | 0.17535 | -0.16473 | 0.30462 | -0.25692 | -0.15259 | 0.21949 | 0.01462 |
| IB_PN_F_AB_nCi_2_NS3_X_KA_e_MAS | 0.3726 | 0.1881 | -0.05473 | 0.0825 | 0.06869 | 0.16613 | -0.14607 | 0.15004 | -0.12200 | -0.06240 | 0.13428 | 0.13280 |
| ZM1 | 0.8250 | 0.0307 | 0.04508 | 0.0593 | 0.06426 | -0.00502 | 0.02683 | 0.52427 | 0.04685 | 0.15504 | 0.01420 | 0.00885 |
| ZM1V | 0.7503 | -0.2384 | -0.03786 | 0.0651 | 0.04953 | 0.01081 | 0.03671 | 0.56238 | -0.09020 | 0.02158 | -0.03273 | -0.11339 |
| ZM2 | 0.8254 | 0.0274 | 0.04913 | 0.0545 | 0.08298 | -0.00868 | 0.02466 | 0.48676 | 0.05209 | 0.23118 | 0.01644 | 0.00950 |
| ZM2V | 0.7924 | -0.1753 | -0.01262 | 0.0638 | 0.11427 | 0.04328 | 0.02218 | 0.50840 | -0.13006 | 0.08249 | -0.04578 | -0.01457 |
| Qindex | 0.7887 | 0.0427 | 0.03208 | 0.0340 | 0.11721 | -0.02451 | 0.02934 | 0.34716 | 0.03065 | 0.44026 | 0.02630 | 0.01683 |
| SNar | 0.8290 | 0.0601 | -0.00354 | 0.0853 | 0.06165 | 0.04045 | 0.02895 | 0.52437 | 0.01684 | 0.06187 | 0.02089 | 0.06140 |
| HNar | 0.3357 | 0.3238 | -0.38279 | 0.1030 | 0.14293 | 0.25518 | 0.03878 | -0.19771 | -0.24594 | 0.06080 | 0.11843 | 0.44609 |
| GNar | 0.4835 | 0.2802 | -0.33900 | 0.0981 | 0.18419 | 0.19229 | 0.03659 | -0.21077 | -0.25382 | 0.22821 | 0.11440 | 0.37763 |
| Xt | -0.9379 | -0.1769 | 0.07323 | -0.1214 | -0.11912 | -0.01980 | -0.01649 | 0.00097 | 0.07624 | 0.00810 | -0.06001 | -0.07551 |
| Dz | 0.7918 | -0.0394 | 0.03022 | 0.0658 | 0.03283 | 0.00783 | 0.02449 | 0.59752 | 0.05333 | 0.01062 | 0.00323 | -0.02994 |
| Ram | 0.7820 | -0.0504 | 0.09211 | 0.0224 | 0.08961 | -0.05461 | 0.01659 | 0.48433 | 0.05081 | 0.31914 | 0.00748 | -0.06327 |
| Pol | 0.8077 | 0.0121 | 0.07741 | 0.0417 | 0.10743 | -0.01175 | 0.01325 | 0.48114 | 0.07038 | 0.25795 | 0.02516 | -0.02036 |
| LPRS | 0.7395 | 0.0131 | 0.04518 | 0.0617 | 0.01907 | 0.00717 | 0.02503 | 0.66177 | 0.05291 | -0.01621 | 0.00510 | 0.00707 |
| VDA | 0.5976 | 0.0167 | 0.02351 | 0.0549 | -0.01779 | 0.00262 | 0.02670 | 0.76962 | 0.04461 | -0.10073 | 0.00663 | 0.00989 |
| MSD | -0.7521 | 0.0012 | -0.02574 | -0.0295 | -0.14083 | 0.01676 | 0.01170 | -0.01836 | 0.14060 | -0.49112 | -0.13577 | 0.05446 |
| SMTI | 0.3905 | -0.0190 | 0.01066 | 0.0266 | -0.03821 | 0.01146 | 0.01942 | 0.85616 | -0.02349 | -0.03409 | 0.01854 | -0.00022 |
| SMTIV | 0.3668 | -0.0424 | -0.00465 | 0.0277 | -0.03848 | 0.01293 | 0.01978 | 0.84588 | -0.04577 | -0.03666 | 0.01868 | -0.01671 |
| GMTI | 0.3914 | -0.0192 | 0.00681 | 0.0275 | -0.03560 | 0.01486 | 0.01866 | 0.85457 | -0.02563 | -0.02423 | 0.02027 | 0.00206 |
| GMTIV | 0.3394 | -0.0564 | -0.02016 | 0.0264 | -0.03605 | 0.01613 | 0.01993 | 0.82456 | -0.06659 | -0.03126 | 0.02158 | -0.02922 |
| Xu | 0.8546 | 0.0558 | 0.03775 | 0.0889 | 0.03614 | 0.00290 | 0.02659 | 0.49002 | 0.06370 | -0.05904 | 0.00220 | 0.01001 |
| SPI | 0.6923 | -0.0766 | 0.14311 | 0.0286 | 0.01671 | -0.06910 | 0.01064 | 0.65778 | 0.09112 | 0.02058 | -0.02745 | -0.11464 |
| W | 0.3857 | -0.0191 | 0.01422 | 0.0255 | -0.04147 | 0.00808 | 0.02013 | 0.85761 | -0.02159 | -0.04547 | 0.01692 | -0.00243 |
| WA | 0.7329 | 0.1066 | 0.00634 | 0.1100 | -0.01896 | -0.00388 | 0.03426 | 0.48358 | 0.11047 | -0.33405 | -0.02609 | 0.02894 |
| Har | 0.7989 | 0.0162 | 0.05236 | 0.0567 | 0.05805 | 0.00583 | 0.02374 | 0.57061 | 0.04581 | 0.12320 | 0.01166 | 0.00785 |
| Har2 | 0.7147 | -0.0099 | 0.05928 | 0.0428 | 0.03564 | 0.00981 | 0.02190 | 0.67520 | 0.03814 | 0.09751 | 0.00862 | 0.00302 |
| QW | 0.3699 | -0.0125 | 0.01300 | 0.0241 | -0.05159 | 0.00040 | 0.02383 | 0.84969 | -0.02241 | -0.08296 | 0.01525 | -0.00517 |
| TI1 | 0.4179 | -0.0330 | -0.02748 | 0.0327 | 0.00843 | 0.05485 | 0.00477 | 0.72966 | -0.04941 | 0.12911 | 0.03394 | 0.01187 |
| TI2 | 0.3898 | 0.1381 | -0.03449 | 0.1078 | -0.11310 | -0.01666 | 0.04603 | 0.31032 | 0.17046 | -0.60682 | -0.07120 | 0.03467 |
| STN | 0.7480 | 0.1108 | -0.10442 | 0.0708 | 0.16884 | 0.08386 | 0.03102 | 0.21485 | -0.08246 | 0.42762 | 0.06341 | 0.14444 |
| HyDp | 0.2833 | -0.0042 | -0.00565 | 0.0259 | -0.05808 | -0.00125 | 0.02068 | 0.83857 | -0.03960 | -0.06911 | 0.02189 | -0.01215 |
| RHyDp | 0.7882 | 0.0103 | 0.05690 | 0.0531 | 0.05682 | 0.00547 | 0.02310 | 0.58293 | 0.04660 | 0.12874 | 0.01071 | 0.00517 |
| w | 0.4059 | -0.0247 | 0.04210 | 0.0185 | -0.01339 | 0.01892 | 0.01188 | 0.79640 | -0.02331 | 0.06472 | -0.00177 | 0.02093 |
| ww | 0.2825 | -0.0236 | 0.03063 | 0.0163 | -0.02906 | 0.02199 | -0.00147 | 0.70839 | -0.04899 | 0.05314 | -0.01061 | 0.02731 |
| Rww | 0.5338 | -0.0301 | 0.10705 | 0.0593 | -0.13313 | -0.06266 | 0.01032 | 0.60495 | 0.13858 | -0.35945 | -0.03348 | -0.10436 |
| D/D | 0.4611 | -0.0181 | 0.01667 | 0.0371 | -0.05853 | -0.00195 | 0.02152 | 0.80019 | 0.01658 | -0.15583 | 0.01811 | -0.02088 |
| WhetZ | 0.3893 | -0.0136 | 0.01963 | 0.0237 | -0.03479 | 0.00616 | 0.02102 | 0.85891 | -0.00066 | -0.03777 | 0.01142 | 0.00187 |
| Whetm | 0.3892 | -0.0136 | 0.01963 | 0.0237 | -0.03475 | 0.00618 | 0.02105 | 0.85892 | -0.00065 | -0.03775 | 0.01143 | 0.00187 |
| Whetv | 0.3665 | -0.0400 | 0.00651 | 0.0271 | -0.03864 | 0.02158 | 0.01462 | 0.85317 | -0.00537 | -0.03784 | 0.02636 | -0.01081 |
| Whete | 0.3891 | -0.0136 | 0.01894 | 0.0246 | -0.04034 | 0.00353 | 0.02084 | 0.85851 | -0.00232 | -0.03932 | 0.01286 | 0.00080 |
| Whetp | 0.3635 | -0.0436 | 0.00545 | 0.0269 | -0.03707 | 0.02499 | 0.01381 | 0.85227 | -0.00565 | -0.03800 | 0.02654 | -0.01065 |
| J | -0.5774 | -0.2012 | 0.26659 | -0.1092 | -0.17668 | -0.18212 | -0.02790 | 0.02322 | 0.20168 | -0.23328 | -0.00434 | -0.29167 |
| JhetZ | -0.5734 | -0.2279 | 0.22072 | -0.1068 | -0.37355 | -0.23158 | -0.06804 | 0.02193 | -0.00916 | -0.19215 | 0.15047 | -0.31947 |
| Jhetm | -0.5719 | -0.2270 | 0.22038 | -0.1068 | -0.37462 | -0.23182 | -0.06864 | 0.02211 | -0.00888 | -0.19116 | 0.15178 | -0.31979 |
| Jhetv | -0.6316 | 0.0048 | 0.18152 | -0.1292 | -0.08914 | -0.28265 | -0.04826 | -0.00894 | -0.05606 | -0.17364 | 0.05631 | -0.19593 |
| Jhete | -0.6440 | -0.2750 | 0.20503 | -0.1200 | -0.19522 | -0.13500 | -0.05564 | 0.00198 | -0.03411 | -0.22234 | 0.00843 | -0.29524 |
| Jhetp | -0.6010 | 0.0383 | 0.18850 | -0.1267 | -0.16432 | -0.34597 | -0.05358 | 0.00156 | -0.04317 | -0.16165 | 0.13698 | -0.20752 |
| MAXDN | 0.2832 | -0.3215 | -0.17841 | 0.0150 | -0.32525 | -0.29026 | 0.09516 | 0.09818 | -0.04367 | -0.02383 | -0.01945 | -0.53479 |
| MAXDP | 0.6825 | -0.1534 | 0.02865 | 0.0906 | 0.12256 | -0.19504 | 0.13847 | 0.10399 | 0.00378 | 0.15621 | -0.11820 | -0.19364 |
| DELS | 0.6232 | -0.2787 | -0.05321 | 0.0260 | 0.03414 | -0.05288 | 0.01067 | 0.64132 | 0.05916 | 0.09296 | 0.00131 | -0.18042 |
| TIE | 0.4276 | -0.0693 | 0.06555 | 0.0110 | -0.03702 | -0.07843 | 0.03595 | 0.65238 | 0.09428 | -0.10676 | -0.00785 | -0.05713 |
| S0K | 0.7476 | -0.0039 | 0.04392 | 0.0756 | 0.04653 | 0.01186 | 0.02442 | 0.64146 | 0.05962 | 0.04669 | -0.01683 | -0.01189 |
| S1K | 0.7327 | 0.0107 | 0.10239 | 0.0556 | -0.01595 | -0.02166 | 0.00452 | 0.63662 | 0.13633 | -0.09038 | 0.00956 | -0.03581 |
| S2K | 0.5889 | 0.0504 | 0.07277 | 0.0859 | -0.05732 | 0.00855 | -0.00413 | 0.63876 | 0.17786 | -0.37241 | 0.00437 | 0.02532 |
| S3K | 0.2929 | 0.0721 | 0.11290 | 0.0499 | -0.14638 | -0.05326 | 0.02691 | 0.59062 | 0.24929 | -0.55205 | -0.04749 | -0.01337 |
| PHI | 0.4906 | 0.0410 | 0.11218 | 0.0692 | -0.09169 | -0.02624 | -0.01446 | 0.63547 | 0.24076 | -0.42317 | 0.00898 | -0.00056 |
| BLI | -0.1915 | 0.3189 | 0.31674 | 0.0125 | -0.45957 | -0.25832 | -0.07756 | 0.07847 | 0.41710 | -0.13859 | 0.27843 | 0.01483 |
| PW2 | 0.4925 | -0.0196 | 0.04526 | -0.0742 | 0.06701 | -0.21548 | 0.02757 | -0.08385 | -0.04657 | 0.66289 | 0.07607 | -0.19879 |
| PW3 | 0.5459 | -0.0395 | -0.02786 | 0.0237 | 0.22511 | -0.02224 | -0.03858 | -0.13452 | -0.09437 | 0.59344 | 0.18963 | -0.01961 |
| PW4 | 0.5987 | 0.0451 | -0.08290 | 0.0840 | 0.23308 | 0.04720 | -0.01457 | -0.20313 | -0.19909 | 0.46104 | 0.13945 | 0.11124 |
| PW5 | 0.6598 | 0.0925 | -0.08782 | 0.0748 | 0.24357 | 0.07504 | -0.03694 | -0.19366 | -0.21931 | 0.38563 | 0.07563 | 0.09715 |
| PJI2 | 0.3268 | 0.1339 | -0.03113 | 0.0972 | 0.00365 | -0.03237 | 0.00228 | -0.10760 | -0.01556 | -0.19268 | -0.07192 | -0.11383 |
| CSI | 0.6059 | 0.0306 | 0.00631 | 0.0660 | -0.00806 | 0.00572 | 0.02700 | 0.75243 | 0.05370 | -0.08365 | 0.00505 | 0.02039 |
| ECC | 0.5932 | 0.0285 | 0.01506 | 0.0637 | -0.01520 | -0.00074 | 0.02684 | 0.75834 | 0.05904 | -0.11112 | 0.00146 | 0.01266 |
| AECC | 0.7187 | 0.1295 | -0.01267 | 0.1232 | -0.00201 | -0.00859 | 0.03779 | 0.44904 | 0.12194 | -0.33185 | -0.03719 | 0.03975 |
| DECC | 0.6389 | 0.1785 | -0.01755 | 0.1279 | -0.00021 | -0.03659 | 0.05433 | 0.37345 | 0.15414 | -0.33865 | -0.07608 | 0.00452 |
| MDDD | 0.5986 | 0.0377 | 0.01930 | 0.0643 | -0.01712 | 0.00045 | 0.02091 | 0.74004 | 0.06349 | -0.10259 | -0.00683 | -0.00922 |
| UNIP | 0.5904 | 0.0187 | 0.02464 | 0.0550 | -0.01650 | -0.00383 | 0.03141 | 0.76286 | 0.04895 | -0.10870 | 0.00576 | 0.01783 |
| CENT | 0.3673 | -0.0206 | 0.00819 | 0.0190 | -0.03744 | 0.01747 | 0.01290 | 0.83257 | -0.03528 | -0.03195 | 0.01733 | -0.01505 |
| VAR | 0.5787 | 0.0264 | 0.01703 | 0.0692 | -0.01310 | 0.01871 | 0.01073 | 0.75083 | 0.05649 | -0.08394 | -0.00642 | -0.00478 |
| BAC | 0.4166 | -0.0987 | 0.13810 | -0.0261 | -0.03740 | -0.05333 | 0.01071 | 0.78148 | 0.05953 | 0.00661 | 0.00438 | -0.09898 |
| Lop | -0.1478 | 0.0564 | 0.27289 | 0.1045 | -0.11340 | -0.07406 | -0.03244 | 0.05419 | 0.30714 | -0.50048 | -0.23634 | -0.24456 |
| ICR | 0.7849 | 0.1776 | -0.02878 | 0.1632 | 0.04291 | -0.00307 | 0.04046 | 0.17641 | 0.08968 | -0.33673 | -0.08108 | -0.00248 |
| D/Dr03 | 0.1544 | -0.0175 | 0.10142 | -0.0369 | 0.03140 | -0.05698 | 0.00809 | 0.04479 | 0.07346 | 0.22159 | -0.03769 | 0.05152 |
| D/Dr04 | 0.1355 | -0.1041 | -0.02269 | 0.1047 | -0.12430 | -0.11911 | 0.01907 | -0.00675 | -0.00051 | 0.01547 | -0.06226 | 0.04655 |
| D/Dr05 | 0.3777 | -0.0416 | -0.02041 | 0.0629 | -0.10059 | -0.01450 | 0.02587 | 0.29586 | 0.07125 | 0.21339 | -0.11799 | 0.12709 |
| D/Dr06 | 0.4820 | -0.0050 | -0.07701 | 0.0408 | 0.05546 | 0.08391 | -0.00219 | 0.60012 | -0.06731 | 0.02023 | 0.10394 | -0.01465 |
| D/Dr07 | 0.2098 | -0.0437 | 0.07227 | 0.0287 | 0.01189 | -0.01365 | 0.00602 | -0.00834 | 0.03894 | 0.25296 | -0.02113 | 0.05324 |
| D/Dr08 | 0.1963 | -0.0814 | -0.01505 | 0.0600 | -0.04515 | -0.04925 | 0.00901 | 0.07906 | 0.08301 | 0.24061 | -0.05164 | 0.06368 |
| D/Dr09 | 0.2999 | 0.0123 | 0.00194 | 0.0575 | 0.03170 | 0.05500 | 0.03292 | 0.04959 | 0.04460 | 0.34289 | -0.03385 | 0.05365 |
| D/Dr10 | 0.4843 | 0.0734 | 0.08024 | 0.0000 | 0.18377 | -0.01657 | 0.02379 | 0.20504 | 0.05116 | 0.39289 | 0.00713 | -0.04086 |
| D/Dr11 | 0.2073 | -0.0154 | 0.07374 | -0.0110 | 0.03052 | 0.05720 | -0.01750 | 0.03297 | 0.02462 | 0.29971 | -0.00459 | 0.04698 |
| D/Dr12 | 0.1976 | -0.0427 | 0.05963 | -0.0222 | 0.03529 | 0.02499 | 0.00977 | 0.12698 | 0.04561 | 0.29466 | -0.02373 | 0.03919 |
| T(N..N) | 0.1556 | -0.0640 | -0.02261 | 0.0316 | -0.12701 | 0.02130 | 0.00825 | 0.50607 | -0.02657 | -0.07594 | -0.06469 | 0.05966 |
| T(N..O) | 0.2062 | -0.0793 | -0.04335 | 0.0550 | -0.14100 | 0.01818 | 0.02425 | 0.64175 | -0.03415 | -0.07854 | -0.03298 | -0.00328 |
| T(N..S) | 0.1169 | -0.0161 | -0.02883 | 0.0605 | -0.16524 | -0.04767 | 0.01247 | 0.47371 | -0.08763 | -0.04320 | -0.02324 | -0.01160 |
| T(N..F) | 0.1113 | -0.0237 | -0.09131 | -0.0463 | -0.08143 | 0.00279 | -0.06935 | -0.06182 | -0.05226 | -0.08173 | -0.03321 | -0.11806 |
| T(N..Cl) | 0.0910 | -0.0156 | -0.05941 | -0.0571 | -0.17905 | 0.02717 | -0.23033 | 0.03240 | -0.07244 | -0.08315 | -0.09088 | 0.11391 |
| T(O..O) | 0.2039 | -0.0683 | -0.04728 | 0.0219 | -0.00390 | 0.02525 | 0.01386 | 0.66301 | -0.03687 | 0.00234 | 0.06309 | -0.08463 |
| T(O..F) | 0.1367 | -0.0317 | -0.07129 | -0.0160 | -0.03484 | -0.04053 | -0.09840 | -0.06457 | -0.00992 | -0.02945 | -0.05091 | -0.17139 |
| T(O..Cl) | 0.0967 | -0.0215 | -0.01588 | -0.0119 | -0.12755 | -0.01904 | -0.32453 | 0.10306 | -0.07997 | -0.01921 | -0.06309 | -0.02562 |
| T(Cl..Cl) | -0.0072 | -0.0647 | -0.00663 | -0.1075 | -0.18821 | -0.11265 | -0.26393 | -0.01095 | -0.02925 | 0.14252 | 0.09963 | 0.21464 |
| MW | 0.7845 | 0.0001 | 0.05253 | 0.0592 | -0.04592 | -0.03431 | -0.03625 | 0.59620 | 0.06002 | 0.01051 | 0.03341 | -0.00583 |
| AMW | -0.0885 | -0.3592 | -0.25486 | -0.1199 | -0.37174 | -0.26587 | -0.32966 | -0.02541 | -0.42769 | 0.09686 | 0.07979 | -0.06569 |
| Sv | 0.7782 | 0.1542 | 0.11605 | 0.0664 | 0.05412 | 0.00564 | 0.03715 | 0.55495 | 0.14765 | 0.00965 | 0.01835 | 0.04360 |
| Se | 0.7472 | 0.1294 | 0.13050 | 0.0620 | 0.06259 | 0.01961 | 0.05616 | 0.56926 | 0.22730 | 0.00660 | 0.01438 | 0.00849 |
| Sp | 0.7702 | 0.1711 | 0.12835 | 0.0658 | 0.04132 | -0.00360 | 0.03214 | 0.55297 | 0.16337 | 0.00817 | 0.02692 | 0.04491 |
| Ss | 0.7371 | -0.1660 | -0.03487 | 0.0489 | 0.00981 | -0.04131 | 0.01675 | 0.62424 | 0.00572 | -0.01469 | -0.02138 | -0.11811 |
| Mv | 0.0243 | -0.0659 | -0.31855 | -0.0741 | -0.13333 | -0.21255 | -0.28242 | -0.13897 | -0.73061 | 0.10022 | 0.09118 | 0.22396 |
| Me | -0.0429 | -0.6678 | -0.30140 | -0.1222 | -0.17423 | -0.13153 | -0.12171 | 0.03833 | -0.23394 | 0.05852 | -0.09201 | -0.38281 |
| Mp | -0.0291 | 0.0363 | -0.20297 | -0.0644 | -0.26467 | -0.29461 | -0.33934 | -0.11137 | -0.61967 | 0.09000 | 0.19240 | 0.24292 |
| Ms | -0.1828 | -0.6399 | -0.26332 | -0.1094 | -0.08718 | -0.20103 | -0.00345 | 0.10816 | -0.07888 | -0.03775 | -0.16612 | -0.51442 |
| nAT | 0.7404 | 0.1596 | 0.14224 | 0.0623 | 0.06757 | 0.02057 | 0.06058 | 0.55769 | 0.23834 | 0.00594 | 0.01567 | 0.02070 |
| nSK | 0.8019 | 0.0234 | 0.04909 | 0.0686 | 0.03573 | 0.00457 | 0.02431 | 0.58341 | 0.05232 | 0.01076 | 0.00771 | 0.00458 |
| nBT | 0.7564 | 0.1598 | 0.12979 | 0.0644 | 0.07498 | 0.02431 | 0.06017 | 0.54556 | 0.22403 | 0.03515 | 0.01838 | 0.02927 |
| nBO | 0.8205 | 0.0349 | 0.03149 | 0.0714 | 0.05283 | 0.01333 | 0.02627 | 0.55111 | 0.03874 | 0.06859 | 0.01369 | 0.02300 |
| nBM | 0.4060 | 0.0023 | -0.17995 | 0.0780 | -0.06197 | 0.06238 | 0.01401 | 0.16945 | -0.72457 | -0.23527 | 0.04420 | 0.13725 |
| SCBO | 0.8203 | 0.0232 | -0.00368 | 0.0800 | 0.02578 | -0.00005 | 0.03333 | 0.53983 | -0.10972 | 0.01344 | 0.00145 | 0.03803 |
| ARR | -0.1267 | 0.0658 | -0.27175 | 0.0073 | 0.02296 | 0.18527 | -0.07749 | -0.13932 | -0.74973 | -0.16554 | 0.15291 | 0.18758 |
| nCIC | 0.7378 | 0.1078 | -0.10245 | 0.0732 | 0.16113 | 0.07236 | 0.03333 | 0.17588 | -0.06844 | 0.46364 | 0.05281 | 0.14853 |
| nCIR | 0.4753 | 0.0858 | 0.02424 | 0.0270 | 0.12004 | 0.04605 | 0.03255 | 0.16701 | 0.00326 | 0.46661 | 0.00848 | 0.08275 |
| RBN | 0.4091 | 0.0570 | 0.10278 | 0.1138 | -0.13175 | 0.01370 | 0.01946 | 0.46308 | 0.21453 | -0.54762 | -0.05126 | -0.03313 |
| RBF | -0.0375 | -0.0672 | 0.11853 | 0.0996 | -0.23891 | -0.01176 | -0.03604 | 0.06741 | 0.20341 | -0.72928 | -0.13148 | -0.15259 |
| nDB | 0.4226 | -0.1335 | 0.05785 | 0.0410 | -0.12503 | -0.35705 | 0.13461 | 0.41976 | -0.08275 | -0.01274 | -0.27908 | -0.11214 |
| nAB | 0.2679 | 0.0472 | -0.20399 | 0.0653 | -0.01937 | 0.18954 | -0.03096 | 0.02696 | -0.71519 | -0.23602 | 0.14435 | 0.18174 |
| nH | 0.6400 | 0.2637 | 0.21121 | 0.0530 | 0.08989 | 0.03266 | 0.08710 | 0.49937 | 0.37907 | 0.00149 | 0.02134 | 0.03288 |
| nC | 0.7899 | 0.2376 | 0.11371 | 0.0692 | 0.15701 | -0.00003 | 0.04834 | 0.48494 | 0.05830 | 0.02785 | 0.01550 | 0.08133 |
| nN | 0.1727 | -0.2306 | -0.15167 | 0.0507 | -0.52603 | 0.22903 | 0.05406 | 0.23852 | -0.00290 | -0.15270 | -0.15455 | 0.18552 |
| nO | 0.5486 | -0.3710 | -0.03218 | 0.0453 | 0.16505 | -0.02464 | 0.06437 | 0.56472 | 0.06467 | 0.05410 | 0.02361 | -0.26730 |
| nS | 0.0253 | -0.0423 | 0.01727 | 0.0857 | -0.55391 | -0.29305 | -0.03807 | 0.17018 | -0.09844 | -0.10645 | 0.17620 | -0.08108 |
| nF | 0.1152 | -0.0282 | -0.09976 | -0.0543 | -0.04659 | -0.01645 | -0.11748 | -0.07179 | -0.03117 | -0.06345 | -0.03317 | -0.19943 |
| nCL | -0.0071 | -0.0482 | -0.01998 | -0.1157 | -0.29076 | -0.10545 | -0.52294 | -0.03342 | -0.08615 | 0.09483 | 0.03204 | 0.19850 |
| nX | 0.0578 | -0.0468 | -0.07283 | -0.1248 | -0.28270 | -0.10921 | -0.50168 | -0.07083 | -0.09654 | 0.03693 | 0.02128 | 0.03657 |
| nR05 | 0.3279 | -0.0258 | -0.04610 | 0.0674 | -0.08122 | -0.01626 | 0.04039 | 0.01153 | 0.10462 | 0.44819 | -0.13774 | 0.16126 |
| nR06 | 0.6502 | 0.1573 | -0.12517 | 0.0466 | 0.23639 | 0.10541 | 0.01189 | 0.16021 | -0.14108 | 0.30679 | 0.14598 | 0.07536 |
| nR07 | 0.1810 | -0.0182 | 0.06513 | 0.0232 | 0.03137 | 0.01638 | -0.02058 | -0.04952 | 0.04558 | 0.28418 | -0.00580 | 0.06111 |
| nR08 | 0.1341 | -0.0257 | -0.01683 | 0.0119 | -0.03057 | -0.03618 | 0.02325 | -0.00041 | 0.13579 | 0.31033 | -0.01588 | 0.10486 |
| nR09 | 0.2466 | 0.0460 | -0.03723 | 0.0717 | 0.05909 | 0.05030 | 0.04131 | -0.05414 | 0.03015 | 0.41529 | -0.03026 | 0.06511 |
| nR10 | 0.4371 | 0.1622 | 0.07599 | -0.0097 | 0.24150 | -0.05165 | 0.03749 | -0.01926 | 0.03497 | 0.47717 | 0.01485 | -0.01497 |
| nR11 | 0.1745 | 0.0203 | 0.04943 | -0.0051 | 0.03851 | 0.07332 | -0.03371 | -0.03079 | 0.02856 | 0.29508 | 0.01508 | 0.05103 |
| nR12 | 0.1885 | -0.0168 | 0.03413 | -0.0327 | 0.04644 | 0.02935 | 0.02526 | 0.03464 | 0.06867 | 0.31248 | -0.00585 | 0.05393 |
| nBnz | 0.2174 | 0.0936 | -0.18382 | 0.0350 | 0.11454 | 0.13932 | -0.03400 | 0.02306 | -0.65733 | -0.30218 | 0.21124 | 0.09578 |
| Ui | 0.3656 | -0.0056 | -0.16286 | 0.1109 | -0.03255 | 0.03927 | -0.01690 | -0.02654 | -0.76936 | -0.23669 | -0.01970 | 0.07069 |
| Hy | 0.1112 | -0.3907 | -0.30425 | -0.0613 | -0.02479 | 0.11441 | 0.05544 | 0.52289 | 0.21349 | -0.09083 | 0.01497 | -0.19104 |
| AMR | 0.7919 | 0.1463 | 0.10359 | 0.0706 | 0.00075 | -0.01251 | 0.00411 | 0.55554 | 0.05648 | -0.03176 | 0.03175 | 0.06276 |
| TPSA(NO) | 0.4773 | -0.4276 | -0.17760 | 0.0248 | -0.03727 | 0.01050 | 0.09263 | 0.61816 | 0.08213 | -0.01909 | -0.05662 | -0.21136 |
| TPSA(Tot) | 0.4556 | -0.4233 | -0.15585 | 0.0373 | -0.12725 | -0.04153 | 0.06237 | 0.62148 | 0.07142 | -0.03874 | -0.02154 | -0.20861 |
| MLOGP | 0.1422 | 0.6538 | 0.18698 | 0.0096 | 0.07631 | -0.25248 | -0.14193 | -0.32411 | -0.14286 | -0.02089 | -0.03098 | 0.27684 |
| MLOGP2 | 0.2358 | 0.5215 | 0.04194 | -0.0636 | 0.02602 | -0.14125 | -0.10246 | 0.31372 | 0.11500 | -0.09427 | 0.02753 | 0.07836 |
| ALOGP | 0.2222 | 0.6327 | 0.26965 | -0.0088 | 0.07502 | -0.21500 | -0.12660 | -0.11061 | -0.20033 | -0.17022 | 0.01303 | 0.22542 |
| ALOGP2 | 0.2455 | 0.4391 | 0.09098 | -0.0599 | 0.03085 | -0.12314 | -0.06176 | 0.27930 | 0.07442 | -0.22844 | 0.04130 | 0.03978 |
| LAI | 0.4251 | -0.1478 | -0.03620 | -0.0131 | 0.03029 | 0.05698 | -0.02169 | 0.52716 | 0.16083 | -0.03158 | 0.04635 | -0.05249 |
| GVWAI-80 | 0.0581 | 0.0742 | -0.00105 | 0.1399 | 0.05132 | 0.05564 | 0.05030 | -0.46185 | -0.34519 | -0.12784 | 0.03416 | 0.05793 |
| GVWAI-50 | 0.1222 | 0.0500 | -0.00346 | 0.1328 | 0.03454 | 0.12410 | 0.07872 | -0.24380 | -0.24108 | -0.17293 | 0.08260 | 0.07109 |
| Inflammat-80 | 0.1391 | 0.1000 | -0.01034 | 0.0560 | 0.07417 | 0.09254 | 0.04463 | -0.31281 | -0.30755 | -0.18339 | 0.05416 | 0.10206 |
| Inflammat-50 | 0.0240 | 0.0237 | -0.01309 | 0.0728 | 0.02322 | 0.00663 | -0.07025 | -0.08574 | -0.21919 | -0.09229 | 0.01831 | 0.03763 |
| Depressant-80 | 0.0884 | 0.1704 | 0.06343 | 0.1522 | 0.07268 | 0.14739 | 0.02441 | -0.25540 | -0.17774 | -0.16837 | 0.06870 | 0.07395 |
| Depressant-50 | 0.0286 | 0.1075 | 0.01973 | 0.0991 | 0.02209 | 0.13826 | -0.00322 | -0.11439 | -0.07192 | -0.08901 | 0.04361 | 0.04468 |
| Psychotic-80 | 0.2149 | 0.1592 | 0.02206 | 0.1040 | 0.03038 | 0.10099 | -0.06173 | -0.15819 | -0.09113 | -0.11675 | 0.04147 | 0.05715 |
| Hypertens-80 | 0.2407 | 0.0053 | -0.00716 | 0.1374 | 0.02026 | 0.06461 | 0.11754 | -0.39898 | -0.22774 | -0.13409 | 0.00400 | 0.06483 |
| Hypertens-50 | 0.2030 | -0.0788 | 0.01835 | 0.0778 | -0.00653 | 0.06555 | 0.05160 | -0.19275 | -0.09313 | -0.09421 | 0.05733 | -0.00070 |
| Hypnotic-80 | -0.1887 | -0.0140 | -0.00934 | 0.0473 | 0.08671 | 0.06882 | 0.11572 | -0.25270 | -0.35858 | -0.09663 | 0.04349 | 0.06279 |
| Hypnotic-50 | -0.0550 | -0.0041 | -0.00445 | -0.0314 | 0.08661 | -0.03755 | 0.07239 | -0.11705 | -0.15563 | -0.06348 | -0.03113 | 0.06241 |
| Neoplastic-80 | 0.1048 | -0.0547 | -0.04887 | 0.1169 | -0.00963 | 0.07070 | 0.09610 | -0.45455 | -0.30441 | -0.14830 | 0.01541 | 0.02660 |
| Neoplastic-50 | 0.1499 | -0.0747 | -0.00807 | 0.1250 | -0.00290 | 0.06375 | 0.12044 | -0.24822 | -0.20169 | -0.12199 | 0.06549 | 0.01654 |
| Infective-80 | -0.0393 | 0.0761 | -0.02909 | 0.0961 | 0.04041 | 0.07539 | 0.02072 | -0.42661 | -0.37400 | -0.11611 | 0.07000 | 0.08393 |
| Infective-50 | 0.0337 | -0.0139 | -0.07119 | 0.0442 | 0.02067 | -0.00950 | 0.12897 | -0.18423 | -0.31046 | -0.12448 | 0.05553 | 0.08412 |
| BLTF96 | -0.1919 | -0.6817 | -0.17927 | -0.0094 | -0.06373 | 0.25302 | 0.14341 | 0.14745 | 0.15714 | 0.03688 | 0.00749 | -0.27619 |
| BLTD48 | -0.1919 | -0.6818 | -0.17921 | -0.0093 | -0.06374 | 0.25295 | 0.14334 | 0.14739 | 0.15715 | 0.03686 | 0.00748 | -0.27620 |
| BLTA96 | -0.1919 | -0.6818 | -0.17930 | -0.0093 | -0.06368 | 0.25308 | 0.14336 | 0.14736 | 0.15706 | 0.03690 | 0.00747 | -0.27621 |
| nCp | 0.4157 | 0.1932 | 0.45266 | -0.0647 | 0.10492 | -0.19149 | 0.04369 | 0.35874 | 0.30796 | 0.16826 | -0.05475 | -0.05830 |
| nCs | 0.4737 | 0.2057 | -0.02197 | 0.0611 | 0.12302 | -0.03310 | 0.06904 | 0.43679 | 0.58584 | 0.07552 | -0.00350 | 0.01342 |
| nCt | 0.4271 | 0.2446 | 0.17876 | -0.0240 | 0.16131 | -0.15071 | 0.01801 | 0.16262 | 0.36650 | 0.48003 | -0.06546 | -0.00953 |
| nCq | 0.3323 | 0.2759 | 0.21706 | -0.0816 | 0.15029 | -0.21226 | 0.06629 | 0.05562 | 0.27265 | 0.50224 | -0.11795 | -0.02275 |
| nCrs | 0.4833 | 0.1147 | -0.03628 | 0.0127 | 0.15558 | 0.00477 | 0.03612 | 0.35384 | 0.49241 | 0.36707 | 0.05057 | -0.00821 |
| nCrt | 0.4243 | 0.1961 | 0.13358 | -0.0187 | 0.16780 | -0.13728 | 0.00728 | 0.08584 | 0.37164 | 0.54356 | -0.05935 | -0.00897 |
| nCrq | 0.3302 | 0.2631 | 0.18976 | -0.0724 | 0.15351 | -0.21109 | 0.06787 | 0.05740 | 0.27777 | 0.51578 | -0.12901 | -0.01424 |
| nCar | 0.2611 | 0.0689 | -0.19720 | 0.0552 | 0.03720 | 0.17727 | -0.03754 | 0.02821 | -0.72003 | -0.26748 | 0.17324 | 0.15097 |
| nCbH | 0.1485 | 0.2106 | -0.28063 | 0.0618 | 0.02005 | 0.13821 | 0.00360 | 0.00143 | -0.55407 | -0.32334 | 0.17511 | 0.17655 |
| nCb- | 0.2447 | -0.0644 | -0.02994 | -0.0002 | 0.19193 | 0.11863 | -0.07293 | 0.04066 | -0.62551 | -0.21031 | 0.19776 | -0.03066 |
| nCconj | 0.2450 | -0.0631 | 0.00449 | 0.0176 | 0.21644 | -0.16857 | 0.03747 | 0.13602 | -0.18325 | 0.01017 | -0.12872 | -0.04620 |
| nR=Cs | 0.1809 | 0.0978 | 0.08464 | -0.0142 | 0.21981 | -0.18364 | 0.04632 | 0.17054 | -0.03406 | -0.03201 | -0.09527 | -0.01150 |
| nR=Ct | 0.2021 | 0.2531 | 0.15784 | -0.0108 | 0.14380 | -0.20305 | 0.04611 | 0.11416 | 0.00292 | 0.01933 | -0.13957 | -0.04167 |
| nRCOOH | 0.0560 | -0.1146 | -0.13273 | 0.0139 | -0.00642 | -0.20777 | 0.06011 | 0.11763 | 0.10097 | -0.13435 | -0.24814 | -0.12473 |
| nRCOOR | 0.3298 | -0.0660 | 0.19635 | 0.0159 | 0.12569 | -0.16821 | 0.01818 | 0.09871 | 0.11506 | 0.22561 | -0.20363 | 0.01782 |
| nRCONHR | 0.1601 | -0.1054 | -0.00494 | 0.0645 | -0.13928 | -0.06239 | 0.05981 | 0.49407 | -0.01045 | -0.12352 | -0.07567 | 0.05056 |
| nRCONR2 | 0.1722 | -0.0878 | -0.01764 | 0.0839 | -0.14125 | -0.04400 | 0.08095 | 0.09923 | -0.00219 | -0.02565 | -0.04671 | 0.12786 |
| nRCO | 0.2222 | 0.0165 | 0.07736 | 0.0290 | 0.16480 | -0.14724 | -0.01585 | 0.00258 | 0.11919 | 0.27383 | -0.12075 | -0.05018 |
| nArCO | 0.1103 | -0.1189 | -0.03747 | -0.0131 | 0.25182 | 0.00050 | 0.05255 | -0.05005 | -0.30474 | -0.07403 | 0.11681 | -0.03046 |
| nRNH2 | -0.0183 | -0.2046 | -0.15945 | -0.0842 | -0.02120 | 0.14276 | 0.03030 | 0.17993 | 0.24471 | -0.08028 | -0.03407 | -0.02560 |
| nArNH2 | -0.0148 | -0.0589 | -0.14174 | -0.0304 | -0.20627 | 0.01136 | 0.11184 | -0.02942 | -0.08919 | -0.00363 | 0.03300 | 0.07668 |
| nRNHR | 0.0143 | -0.0488 | -0.03803 | 0.0427 | -0.05135 | 0.14591 | 0.01875 | 0.11377 | 0.13027 | -0.15005 | -0.02282 | 0.07246 |
| nRNR2 | 0.1766 | 0.1395 | 0.02855 | 0.0642 | -0.08375 | 0.36636 | -0.12898 | -0.05698 | 0.04957 | -0.06707 | 0.11304 | 0.12032 |
| nROH | 0.2962 | -0.2059 | -0.22932 | 0.0320 | 0.16781 | 0.08247 | -0.01407 | 0.42856 | 0.38020 | 0.06125 | 0.09231 | -0.26796 |
| nArOH | 0.0463 | -0.1441 | -0.07353 | -0.0931 | 0.31088 | -0.03738 | 0.04645 | 0.17724 | -0.30369 | -0.09539 | 0.16737 | -0.11943 |
| nOHp | 0.0914 | -0.1330 | -0.17550 | -0.0216 | 0.11829 | 0.21238 | -0.04722 | 0.17684 | 0.30472 | 0.00163 | 0.11185 | -0.15430 |
| nOHs | 0.2767 | -0.1673 | -0.17585 | 0.0447 | 0.17747 | 0.16261 | -0.05368 | 0.39771 | 0.37399 | 0.07829 | 0.15980 | -0.17637 |
| nOHt | 0.2494 | -0.0090 | 0.03362 | 0.0014 | 0.10461 | 0.02083 | 0.01280 | 0.07738 | 0.18043 | 0.25003 | 0.04374 | -0.09224 |
| nROR | 0.3460 | -0.1831 | -0.05179 | 0.0244 | 0.10871 | 0.16235 | -0.04995 | 0.42883 | 0.28925 | 0.14659 | 0.12381 | -0.07832 |
| nArOR | 0.1748 | -0.1956 | 0.19298 | 0.0641 | 0.25117 | 0.24268 | 0.04120 | -0.06977 | -0.30694 | -0.14184 | 0.13334 | -0.03836 |
| nRSR | 0.0968 | -0.0523 | 0.00534 | 0.1073 | -0.22263 | -0.09116 | -0.12607 | 0.01441 | -0.01431 | -0.07174 | -0.03420 | 0.05059 |
| nArX | 0.0408 | -0.0004 | -0.07335 | -0.1008 | -0.20595 | -0.03635 | -0.51719 | -0.04499 | -0.17880 | -0.04416 | -0.03150 | 0.11705 |
| nPyridines | -0.0244 | 0.0799 | -0.09477 | 0.0627 | -0.08621 | 0.17390 | -0.08989 | -0.00643 | -0.15817 | 0.01218 | 0.06884 | 0.06531 |
| nHDon | 0.2255 | -0.3416 | -0.29810 | -0.0316 | 0.01254 | 0.10107 | 0.07706 | 0.55772 | 0.18811 | -0.09090 | 0.03244 | -0.17979 |
| nHAcc | 0.5711 | -0.4288 | -0.10478 | 0.0532 | -0.06596 | 0.05757 | 0.06069 | 0.60024 | 0.05935 | -0.03269 | -0.04001 | -0.18553 |
| nHBonds | 0.2590 | -0.2706 | -0.14421 | 0.0053 | 0.10605 | 0.12205 | 0.01166 | 0.49109 | 0.10752 | -0.05129 | 0.11121 | -0.13401 |
| C-001 | 0.4105 | 0.2309 | 0.50365 | -0.0479 | 0.10373 | -0.23974 | 0.06660 | 0.31595 | 0.23861 | 0.18740 | -0.07227 | -0.02146 |
| C-002 | 0.3301 | 0.4698 | 0.04004 | 0.0532 | 0.09717 | -0.15775 | 0.11850 | 0.17896 | 0.53789 | 0.01110 | -0.09206 | 0.06962 |
| C-003 | 0.3784 | 0.3104 | 0.14376 | -0.0123 | 0.15488 | -0.15845 | 0.04971 | 0.16422 | 0.36550 | 0.40537 | -0.08705 | 0.00336 |
| C-004 | 0.3323 | 0.2759 | 0.21706 | -0.0816 | 0.15029 | -0.21226 | 0.06629 | 0.05562 | 0.27265 | 0.50224 | -0.11795 | -0.02275 |
| C-005 | 0.1841 | -0.1343 | 0.42919 | 0.0941 | -0.03987 | 0.41165 | 0.02123 | 0.03360 | -0.22395 | -0.03158 | 0.12434 | -0.04705 |
| C-006 | 0.1988 | -0.0068 | -0.08121 | 0.0608 | -0.21801 | 0.37598 | -0.08927 | 0.05213 | 0.29966 | -0.29720 | 0.12833 | 0.09256 |
| C-008 | 0.4152 | -0.2272 | -0.09249 | 0.0421 | 0.09858 | 0.14311 | -0.02184 | 0.55005 | 0.35253 | 0.10477 | 0.10653 | -0.06488 |
| C-009 | 0.2919 | -0.2347 | -0.15393 | 0.0774 | 0.01408 | 0.12012 | -0.06388 | 0.38939 | 0.23536 | 0.05127 | 0.11521 | -0.10564 |
| C-011 | 0.3238 | -0.0424 | 0.17107 | -0.0402 | 0.09321 | -0.05367 | -0.06845 | 0.07434 | 0.17869 | 0.41243 | 0.02103 | -0.03591 |
| C-016 | 0.1614 | 0.1625 | 0.11352 | -0.0275 | 0.22510 | -0.18705 | 0.04526 | 0.15525 | 0.01112 | -0.04002 | -0.11362 | -0.01739 |
| C-017 | 0.2021 | 0.2531 | 0.15784 | -0.0108 | 0.14380 | -0.20305 | 0.04611 | 0.11416 | 0.00292 | 0.01933 | -0.13957 | -0.04167 |
| C-019 | 0.1085 | -0.1511 | -0.05522 | 0.0327 | 0.05105 | -0.04549 | 0.01683 | 0.09371 | -0.13623 | 0.01276 | 0.02267 | 0.01298 |
| C-024 | 0.1555 | 0.2170 | -0.28742 | 0.0717 | 0.00650 | 0.15370 | -0.01019 | -0.00496 | -0.58116 | -0.31391 | 0.17098 | 0.19569 |
| C-025 | 0.2716 | 0.0692 | -0.03325 | 0.0515 | 0.21705 | 0.09763 | 0.02226 | 0.03764 | -0.55712 | -0.12964 | 0.13594 | 0.04364 |
| C-026 | 0.1945 | -0.1693 | 0.00564 | -0.0421 | 0.13122 | 0.09000 | -0.12534 | 0.04426 | -0.52611 | -0.20429 | 0.19267 | -0.07347 |
| C-027 | -0.0439 | 0.0174 | -0.11063 | 0.0347 | -0.14866 | 0.12286 | -0.04367 | -0.00629 | -0.11099 | -0.01257 | 0.00523 | 0.09788 |
| C-028 | 0.0846 | 0.0145 | -0.08746 | 0.0460 | -0.17094 | 0.11044 | -0.04294 | 0.07992 | -0.17640 | 0.00951 | -0.00514 | 0.07149 |
| C-029 | 0.0258 | -0.1271 | -0.07281 | 0.0097 | -0.21282 | 0.06522 | 0.06207 | -0.07260 | -0.06466 | -0.00707 | -0.03845 | 0.08291 |
| C-033 | 0.2097 | -0.0468 | 0.10315 | -0.0200 | -0.00240 | -0.05088 | 0.04323 | 0.06961 | -0.04908 | 0.24585 | -0.15650 | 0.13757 |
| C-034 | 0.0551 | -0.0016 | -0.06429 | 0.1045 | -0.07047 | 0.07117 | 0.00853 | -0.02505 | -0.18607 | 0.05383 | -0.06872 | 0.10474 |
| C-038 | 0.2302 | 0.0099 | 0.08017 | 0.0316 | 0.15701 | -0.14100 | -0.02306 | 0.00586 | 0.11513 | 0.26957 | -0.12627 | -0.05329 |
| C-039 | 0.1226 | -0.1263 | -0.04027 | -0.0024 | 0.23252 | -0.00249 | 0.04643 | -0.03695 | -0.31325 | -0.07234 | 0.10589 | -0.02102 |
| C-040 | 0.3392 | -0.2150 | 0.01376 | 0.0522 | -0.09545 | -0.22587 | 0.09791 | 0.46326 | -0.00231 | -0.03944 | -0.28496 | 0.00880 |
| C-041 | -0.0323 | -0.1835 | -0.00823 | -0.0143 | -0.28550 | 0.03642 | 0.00464 | -0.00019 | 0.05497 | -0.07885 | -0.26190 | 0.08391 |
| H-046 | 0.3286 | 0.4915 | 0.27851 | -0.0392 | 0.15316 | -0.27974 | 0.11896 | 0.22016 | 0.37984 | 0.13914 | -0.11605 | 0.00759 |
| H-047 | 0.4728 | -0.0105 | 0.01916 | 0.1285 | -0.02862 | 0.47527 | -0.03735 | 0.30466 | -0.11494 | -0.28902 | 0.23137 | 0.09708 |
| H-048 | 0.3072 | -0.2328 | -0.05436 | 0.0682 | -0.01894 | 0.07832 | -0.00217 | 0.31425 | 0.10512 | 0.14866 | 0.03032 | 0.00396 |
| H-049 | -0.0015 | -0.0799 | -0.10848 | -0.0076 | -0.20276 | 0.12844 | -0.01243 | -0.00813 | -0.11503 | 0.00544 | -0.05862 | 0.12219 |
| H-050 | 0.2205 | -0.3426 | -0.29826 | -0.0322 | 0.01110 | 0.09860 | 0.07565 | 0.56006 | 0.18959 | -0.09090 | 0.03239 | -0.18060 |
| H-051 | 0.2703 | -0.0125 | 0.10466 | 0.0860 | 0.03272 | -0.24386 | 0.07021 | 0.12466 | 0.16169 | 0.14478 | -0.26991 | 0.03339 |
| H-052 | 0.3378 | 0.0495 | 0.23293 | 0.0650 | -0.02135 | 0.07519 | 0.00823 | 0.21445 | 0.32284 | 0.03429 | 0.09567 | 0.05445 |
| H-053 | 0.2694 | -0.0357 | -0.01700 | 0.0066 | 0.01113 | 0.08402 | -0.05130 | 0.16085 | 0.21913 | 0.07397 | 0.06899 | -0.01217 |
| O-056 | 0.2948 | -0.1710 | -0.18328 | 0.0363 | 0.15808 | 0.16812 | -0.03474 | 0.41366 | 0.40037 | 0.11455 | 0.16950 | -0.22656 |
| O-057 | 0.0655 | -0.1924 | -0.14844 | -0.0853 | 0.29929 | -0.14975 | 0.06938 | 0.20983 | -0.26568 | -0.15560 | 0.04253 | -0.18495 |
| O-058 | 0.4120 | -0.2309 | -0.00278 | 0.0787 | -0.17617 | -0.31499 | 0.17879 | 0.43180 | -0.09251 | 0.02032 | -0.21157 | -0.12620 |
| O-059 | 0.3230 | -0.1809 | -0.02002 | 0.0220 | 0.03268 | 0.12581 | -0.06280 | 0.42454 | 0.28343 | 0.12344 | 0.16355 | -0.12206 |
| O-060 | 0.3667 | -0.2281 | 0.30030 | 0.0577 | 0.26145 | 0.10023 | 0.04701 | 0.04015 | -0.24357 | 0.01526 | -0.03546 | -0.02498 |
| N-066 | -0.0183 | -0.2046 | -0.15945 | -0.0842 | -0.02120 | 0.14276 | 0.03030 | 0.17993 | 0.24471 | -0.08028 | -0.03407 | -0.02560 |
| N-067 | -0.0096 | -0.0591 | -0.04803 | 0.0139 | -0.10566 | 0.14614 | 0.01719 | 0.10811 | 0.12233 | -0.14799 | -0.04002 | 0.06503 |
| N-068 | 0.1745 | 0.1252 | 0.02635 | 0.0668 | -0.14086 | 0.34297 | -0.10121 | -0.06355 | 0.04708 | -0.05779 | 0.13752 | 0.11130 |
| N-069 | -0.0271 | -0.0539 | -0.16157 | -0.0332 | -0.30043 | -0.02521 | 0.12671 | -0.03429 | -0.11282 | 0.00390 | 0.05640 | -0.00596 |
| N-072 | 0.1654 | -0.1946 | -0.03627 | 0.0817 | -0.29267 | -0.04591 | 0.10539 | 0.40024 | -0.02658 | -0.11225 | -0.16364 | 0.10464 |
| N-073 | 0.0827 | -0.0268 | -0.09380 | 0.0715 | -0.19509 | 0.13926 | -0.04178 | -0.03923 | -0.15397 | 0.02568 | -0.11160 | 0.14431 |
| N-074 | 0.0301 | -0.1102 | -0.06559 | -0.0498 | -0.23799 | 0.04815 | -0.04579 | 0.04943 | 0.01335 | -0.07445 | -0.22160 | 0.08579 |
| N-075 | 0.0762 | -0.1207 | -0.09909 | 0.0551 | -0.33516 | 0.11558 | 0.01002 | 0.00602 | -0.14201 | -0.01076 | -0.07241 | 0.16915 |
| Cl-089 | 0.0050 | -0.0181 | -0.04536 | -0.1057 | -0.20177 | -0.05029 | -0.53746 | -0.02513 | -0.15799 | 0.02549 | -0.02395 | 0.15262 |
| S-107 | 0.0478 | -0.0517 | 0.06195 | 0.0972 | -0.31258 | -0.18151 | -0.15936 | 0.09736 | -0.02263 | -0.09669 | 0.07514 | 0.06480 |
| S-110 | 0.0713 | 0.0075 | -0.10524 | 0.0441 | -0.43660 | -0.18709 | 0.18622 | 0.16713 | -0.14600 | -0.04336 | 0.13619 | -0.20767 |
| X0 | 0.7862 | 0.0064 | 0.07146 | 0.0600 | 0.02661 | -0.01177 | 0.02210 | 0.60134 | 0.06646 | -0.00084 | 0.00233 | -0.02043 |
| X1 | 0.8051 | 0.0307 | 0.03653 | 0.0762 | 0.03920 | 0.01900 | 0.02431 | 0.57742 | 0.04513 | -0.00332 | 0.01103 | 0.02026 |
| X2 | 0.8099 | 0.0289 | 0.05864 | 0.0515 | 0.03638 | -0.02757 | 0.03068 | 0.56064 | 0.05061 | 0.10222 | 0.00194 | -0.01409 |
| X3 | 0.8231 | 0.0203 | 0.03259 | 0.0583 | 0.09167 | 0.01106 | 0.01726 | 0.49972 | 0.05313 | 0.20336 | 0.03232 | 0.01707 |
| X4 | 0.8323 | 0.0303 | 0.03155 | 0.0670 | 0.10774 | 0.01036 | 0.02573 | 0.43949 | 0.02486 | 0.26113 | 0.02553 | 0.03386 |
| X5 | 0.8187 | 0.0448 | 0.01885 | 0.0647 | 0.12404 | 0.02888 | 0.01830 | 0.40657 | 0.01155 | 0.30092 | 0.02003 | 0.04015 |
| X0A | -0.4371 | -0.3050 | 0.36026 | -0.1092 | -0.17867 | -0.21676 | -0.03267 | 0.20989 | 0.26134 | -0.12703 | -0.10594 | -0.41041 |
| X1A | -0.6440 | -0.1303 | 0.12135 | -0.0127 | -0.14679 | 0.05239 | -0.03731 | 0.18322 | 0.17808 | -0.57582 | -0.10437 | -0.04204 |
| X2A | -0.6459 | -0.0924 | 0.10712 | -0.0645 | -0.21794 | -0.04781 | 0.01058 | 0.19973 | 0.17288 | -0.51578 | -0.17683 | -0.08617 |
| X3A | -0.6639 | -0.1098 | 0.05543 | -0.0535 | -0.14889 | 0.05328 | -0.02154 | 0.22790 | 0.19007 | -0.46716 | -0.09369 | 0.01749 |
| X4A | -0.5391 | -0.0259 | 0.02550 | -0.0290 | -0.13998 | -0.03454 | 0.00380 | 0.10726 | 0.12150 | -0.51238 | -0.00841 | -0.01101 |
| X5A | -0.4584 | -0.0132 | -0.05913 | -0.0226 | -0.08119 | -0.00871 | -0.02581 | 0.05652 | 0.10073 | -0.49925 | -0.04310 | 0.07488 |
| X0v | 0.7841 | 0.1057 | 0.14113 | 0.0612 | -0.01343 | -0.02825 | -0.00332 | 0.56710 | 0.11096 | 0.02024 | 0.02826 | 0.02731 |
| X1v | 0.7789 | 0.1531 | 0.09881 | 0.0731 | -0.05334 | -0.05304 | 0.01204 | 0.54793 | 0.15837 | 0.03572 | 0.06031 | 0.02835 |
| X2v | 0.7553 | 0.1919 | 0.15611 | 0.0370 | -0.05549 | -0.11248 | 0.00943 | 0.48901 | 0.20770 | 0.18296 | 0.06945 | 0.00452 |
| X3v | 0.7399 | 0.2121 | 0.12306 | 0.0395 | -0.02395 | -0.12520 | 0.00115 | 0.39752 | 0.24647 | 0.30870 | 0.07108 | 0.02436 |
| X4v | 0.7135 | 0.2339 | 0.11442 | 0.0359 | 0.00278 | -0.13758 | 0.00302 | 0.32609 | 0.26408 | 0.38146 | 0.05425 | 0.03078 |
| X5v | 0.6861 | 0.2445 | 0.09806 | 0.0338 | 0.02867 | -0.12550 | 0.00168 | 0.27807 | 0.26442 | 0.42614 | 0.03895 | 0.02859 |
| X0Av | -0.1207 | 0.3873 | 0.55972 | 0.0087 | -0.36653 | -0.17800 | -0.20475 | 0.04067 | 0.31376 | 0.02181 | 0.22035 | 0.16199 |
| X1Av | -0.1915 | 0.3189 | 0.31677 | 0.0125 | -0.45969 | -0.25829 | -0.07762 | 0.07872 | 0.41707 | -0.13863 | 0.27824 | 0.01465 |
| X2Av | -0.2052 | 0.2965 | 0.34129 | -0.0151 | -0.40286 | -0.26628 | -0.07756 | 0.09008 | 0.39971 | -0.13224 | 0.27003 | 0.02255 |
| X3Av | -0.2304 | 0.2291 | 0.26263 | -0.0149 | -0.37548 | -0.28394 | -0.06341 | 0.09486 | 0.37743 | -0.13904 | 0.26082 | 0.01318 |
| X4Av | -0.1785 | 0.2005 | 0.21878 | -0.0092 | -0.32432 | -0.29464 | -0.06262 | 0.06012 | 0.33468 | -0.15340 | 0.27247 | -0.02183 |
| X5Av | -0.1275 | 0.1685 | 0.18398 | -0.0049 | -0.26361 | -0.27863 | -0.06918 | 0.02526 | 0.29579 | -0.17098 | 0.27116 | 0.01793 |
| X0sol | 0.7798 | 0.0058 | 0.07783 | 0.0596 | 0.00024 | -0.02517 | 0.00142 | 0.60790 | 0.06142 | 0.00373 | 0.01262 | -0.00358 |
| X1sol | 0.7977 | 0.0288 | 0.04045 | 0.0777 | -0.01469 | -0.00733 | 0.00842 | 0.58825 | 0.03493 | -0.00541 | 0.03141 | 0.02146 |
| X2sol | 0.7955 | 0.0269 | 0.06653 | 0.0545 | -0.04574 | -0.06730 | 0.01044 | 0.57343 | 0.03560 | 0.09852 | 0.03649 | -0.01479 |
| X3sol | 0.8164 | 0.0145 | 0.04146 | 0.0587 | 0.00882 | -0.03023 | -0.01246 | 0.50851 | 0.03796 | 0.20327 | 0.06887 | 0.02267 |
| X4sol | 0.8297 | 0.0253 | 0.03540 | 0.0701 | 0.02411 | -0.03421 | -0.00512 | 0.44583 | 0.00824 | 0.25816 | 0.05930 | 0.03851 |
| X5sol | 0.8212 | 0.0402 | 0.02081 | 0.0693 | 0.04919 | -0.00692 | -0.00981 | 0.41283 | -0.00499 | 0.29715 | 0.04702 | 0.04701 |
| XMOD | 0.7937 | -0.0070 | 0.03151 | 0.0745 | -0.05108 | -0.01598 | -0.01081 | 0.59271 | 0.02449 | -0.00944 | 0.03857 | -0.00334 |
| RDCHI | 0.8520 | 0.1060 | -0.02603 | 0.1131 | 0.03455 | 0.03426 | 0.03265 | 0.44764 | 0.03844 | -0.13650 | 0.00700 | 0.07283 |
| RDSQ | 0.7266 | -0.0069 | 0.05697 | 0.0428 | 0.04692 | 0.01082 | 0.02214 | 0.65236 | 0.03749 | 0.14109 | 0.00933 | 0.00694 |
| ISIZ | 0.7042 | 0.1414 | 0.13590 | 0.0504 | 0.05830 | 0.01707 | 0.05664 | 0.61053 | 0.22747 | 0.01329 | 0.01444 | 0.01986 |
| IAC | 0.7601 | -0.0140 | 0.07265 | 0.0837 | -0.06595 | 0.02536 | 0.02007 | 0.59426 | 0.16223 | -0.02246 | 0.00097 | -0.02122 |
| AAC | -0.0325 | -0.5692 | -0.25020 | -0.0155 | -0.59607 | -0.07548 | -0.16724 | -0.02904 | -0.22202 | -0.02376 | -0.09914 | -0.24556 |
| IDE | 0.8164 | 0.1546 | -0.01041 | 0.1461 | 0.00927 | 0.00412 | 0.03686 | 0.21952 | 0.09401 | -0.38721 | -0.05469 | 0.02186 |
| IDM | 0.9526 | 0.0913 | 0.03291 | 0.1059 | 0.06984 | -0.00811 | 0.01984 | 0.23751 | 0.02639 | -0.01696 | 0.02458 | -0.00527 |
| IDDE | 0.8745 | 0.0761 | 0.01893 | 0.1774 | 0.12335 | 0.04550 | 0.03117 | 0.16957 | 0.03238 | 0.03580 | -0.07287 | -0.04885 |
| IDDM | 0.9506 | 0.0938 | 0.02973 | 0.1066 | 0.06804 | -0.00747 | 0.02179 | 0.24406 | 0.03066 | -0.03006 | 0.02075 | -0.00066 |
| IDET | 0.4865 | -0.0221 | 0.02772 | 0.0318 | -0.02284 | 0.01255 | 0.02031 | 0.83817 | 0.00386 | -0.01900 | 0.01186 | 0.00152 |
| IDMT | 0.3425 | -0.0234 | 0.01122 | 0.0209 | -0.04601 | 0.00904 | 0.01875 | 0.85831 | -0.03621 | -0.03916 | 0.01821 | -0.00497 |
| IVDE | 0.3728 | -0.0544 | 0.29790 | -0.0217 | 0.00898 | -0.28203 | 0.04563 | -0.03482 | 0.08313 | 0.46918 | -0.12514 | -0.35537 |
| IVDM | 0.9493 | 0.1103 | 0.00831 | 0.1151 | 0.07382 | 0.01108 | 0.02196 | 0.23646 | 0.01954 | -0.05304 | 0.02060 | 0.02670 |
| HVcpx | 0.8016 | 0.1476 | -0.02830 | 0.1461 | 0.02426 | 0.01238 | 0.03839 | 0.24982 | 0.09664 | -0.38434 | -0.05006 | 0.04941 |
| HDcpx | 0.9663 | 0.1089 | 0.02488 | 0.1126 | 0.07669 | -0.01358 | 0.01577 | 0.13660 | 0.00217 | -0.02066 | 0.03869 | -0.01770 |
| Uindex | 0.4538 | 0.0259 | 0.11092 | 0.0253 | -0.06451 | -0.08199 | 0.03846 | 0.59413 | 0.11628 | -0.29364 | -0.02383 | -0.03824 |
| Vindex | -0.8390 | -0.2274 | 0.17897 | -0.1307 | -0.15755 | -0.09758 | -0.02258 | 0.07516 | 0.15244 | -0.02176 | -0.01779 | -0.19197 |
| Xindex | -0.8227 | -0.2136 | 0.18800 | -0.1246 | -0.16528 | -0.10981 | -0.02159 | 0.05681 | 0.17328 | -0.11316 | -0.03075 | -0.19893 |
| Yindex | -0.8229 | -0.2352 | 0.16685 | -0.1355 | -0.14711 | -0.09284 | -0.02295 | 0.07881 | 0.11876 | 0.10186 | 0.01512 | -0.19779 |
| IC0 | -0.0325 | -0.5692 | -0.25020 | -0.0155 | -0.59607 | -0.07548 | -0.16724 | -0.02904 | -0.22202 | -0.02376 | -0.09914 | -0.24556 |
| TIC0 | 0.7601 | -0.0140 | 0.07265 | 0.0837 | -0.06595 | 0.02536 | 0.02007 | 0.59426 | 0.16223 | -0.02246 | 0.00097 | -0.02122 |
| SIC0 | -0.5311 | -0.4853 | -0.24661 | -0.1068 | -0.45277 | -0.09206 | -0.15352 | -0.07405 | -0.24527 | 0.06136 | -0.09227 | -0.18233 |
| CIC0 | 0.7571 | 0.3631 | 0.21010 | 0.1114 | 0.25691 | 0.05813 | 0.11323 | 0.22608 | 0.27490 | -0.04212 | 0.05226 | 0.08974 |
| BIC0 | -0.5486 | -0.5005 | -0.21067 | -0.1014 | -0.46610 | -0.08252 | -0.15437 | -0.05987 | -0.17687 | 0.04652 | -0.09389 | -0.20281 |
| IC1 | 0.3766 | -0.4126 | -0.19255 | 0.2438 | -0.11612 | 0.06898 | -0.06108 | -0.05670 | -0.43184 | 0.02900 | -0.23174 | -0.23696 |
| TIC1 | 0.7783 | 0.0094 | 0.08205 | 0.1218 | 0.02645 | 0.04445 | 0.03516 | 0.56592 | 0.09851 | 0.02120 | -0.03341 | -0.02102 |
| SIC1 | -0.3243 | -0.4797 | -0.27132 | 0.0880 | -0.16757 | 0.01260 | -0.10361 | -0.15026 | -0.49071 | 0.09026 | -0.22218 | -0.22472 |
| CIC1 | 0.5304 | 0.4428 | 0.25168 | -0.0341 | 0.15722 | -0.00296 | 0.10184 | 0.25099 | 0.46308 | -0.06558 | 0.15879 | 0.15826 |
| BIC1 | -0.3355 | -0.5029 | -0.23525 | 0.1047 | -0.17127 | 0.02814 | -0.10517 | -0.13875 | -0.42621 | 0.07798 | -0.23675 | -0.25681 |
| IC2 | 0.7180 | -0.1474 | -0.04446 | 0.2803 | 0.03991 | 0.09489 | 0.00087 | 0.02488 | -0.03288 | 0.14950 | -0.20912 | -0.16891 |
| TIC2 | 0.7537 | 0.0640 | 0.10849 | 0.1006 | 0.05733 | 0.03970 | 0.04126 | 0.55999 | 0.19102 | 0.06990 | -0.02569 | -0.00810 |
| SIC2 | -0.0911 | -0.4090 | -0.23750 | 0.1876 | -0.06898 | 0.05940 | -0.07781 | -0.18701 | -0.29465 | 0.23705 | -0.29977 | -0.24918 |
| CIC2 | 0.3194 | 0.4352 | 0.24141 | -0.1346 | 0.08546 | -0.04458 | 0.09221 | 0.28033 | 0.33166 | -0.22025 | 0.24677 | 0.20093 |
| BIC2 | -0.0769 | -0.4131 | -0.16898 | 0.2128 | -0.06163 | 0.08225 | -0.07399 | -0.16324 | -0.15860 | 0.22855 | -0.31607 | -0.28830 |
| IC3 | 0.8046 | 0.0346 | -0.11865 | 0.2171 | 0.13865 | 0.06666 | 0.01917 | 0.08705 | 0.07738 | 0.15989 | -0.14872 | -0.10531 |
| TIC3 | 0.7377 | 0.0972 | 0.09109 | 0.0829 | 0.07295 | 0.03200 | 0.04156 | 0.56940 | 0.21824 | 0.07780 | -0.01508 | -0.00050 |
| SIC3 | 0.0870 | -0.2312 | -0.41080 | 0.1560 | 0.07555 | 0.03939 | -0.06508 | -0.16831 | -0.22337 | 0.29649 | -0.28048 | -0.20916 |
| CIC3 | 0.1190 | 0.2976 | 0.40939 | -0.1294 | -0.04607 | -0.03278 | 0.08565 | 0.24662 | 0.25159 | -0.31309 | 0.25559 | 0.18715 |
| BIC3 | 0.1204 | -0.2193 | -0.33676 | 0.1838 | 0.09505 | 0.06546 | -0.05909 | -0.13517 | -0.04622 | 0.29099 | -0.29764 | -0.25351 |
| IC4 | 0.8253 | 0.0867 | -0.12614 | 0.1884 | 0.18347 | 0.05152 | 0.04097 | 0.12519 | 0.10351 | 0.13669 | -0.11864 | -0.07719 |
| TIC4 | 0.7281 | 0.1070 | 0.08819 | 0.0748 | 0.07943 | 0.02749 | 0.04585 | 0.58065 | 0.22338 | 0.07353 | -0.01079 | 0.00392 |
| SIC4 | 0.1997 | -0.1536 | -0.45631 | 0.1364 | 0.17010 | 0.02182 | -0.03167 | -0.13393 | -0.19757 | 0.27834 | -0.24954 | -0.17735 |
| CIC4 | -0.0052 | 0.2303 | 0.47029 | -0.1188 | -0.14225 | -0.01823 | 0.05375 | 0.19213 | 0.21886 | -0.31961 | 0.24446 | 0.16832 |
| BIC4 | 0.2430 | -0.1331 | -0.37973 | 0.1638 | 0.19535 | 0.04744 | -0.02317 | -0.09563 | -0.00851 | 0.26977 | -0.26371 | -0.22113 |
| IC5 | 0.8392 | 0.1010 | -0.12124 | 0.1802 | 0.20779 | 0.04881 | 0.04304 | 0.11286 | 0.08826 | 0.09339 | -0.08711 | -0.06161 |
| TIC5 | 0.7255 | 0.1080 | 0.08746 | 0.0733 | 0.08236 | 0.02754 | 0.04734 | 0.58670 | 0.22168 | 0.06257 | -0.00696 | 0.00514 |
| SIC5 | 0.3204 | -0.0963 | -0.42235 | 0.1335 | 0.22263 | 0.01690 | -0.02435 | -0.16019 | -0.20667 | 0.19504 | -0.17524 | -0.13701 |
| CIC5 | -0.1303 | 0.1904 | 0.46904 | -0.1251 | -0.20534 | -0.01937 | 0.04417 | 0.19546 | 0.22962 | -0.25409 | 0.19888 | 0.14725 |
| BIC5 | 0.3693 | -0.0722 | -0.35196 | 0.1578 | 0.24767 | 0.03781 | -0.01609 | -0.12905 | -0.03830 | 0.18115 | -0.18132 | -0.17453 |
| ATS1m | 0.9247 | 0.0338 | 0.01865 | 0.0958 | -0.14977 | -0.09652 | -0.04522 | 0.23173 | -0.01608 | 0.01498 | 0.11698 | -0.04170 |
| ATS2m | 0.9293 | 0.0195 | 0.02137 | 0.0712 | -0.09253 | -0.11882 | -0.08072 | 0.18032 | -0.02136 | 0.14260 | 0.10118 | -0.04382 |
| ATS3m | 0.9140 | 0.0073 | 0.02524 | 0.0669 | -0.01788 | -0.10187 | -0.10501 | 0.13109 | -0.02695 | 0.18151 | 0.12976 | -0.04840 |
| ATS4m | 0.9141 | 0.0216 | 0.04712 | 0.0818 | -0.01389 | -0.10628 | -0.08894 | 0.07586 | -0.04771 | 0.10235 | 0.08695 | -0.05992 |
| ATS5m | 0.9243 | 0.0530 | 0.02851 | 0.0948 | -0.00605 | -0.04433 | -0.08170 | 0.06607 | -0.02810 | -0.01781 | 0.02494 | -0.03897 |
| ATS6m | 0.9178 | 0.0839 | 0.04353 | 0.1057 | -0.01272 | -0.01063 | -0.04469 | 0.06651 | -0.00896 | -0.11682 | -0.00937 | -0.02674 |
| ATS7m | 0.8968 | 0.0825 | 0.00772 | 0.1230 | -0.00961 | 0.00721 | -0.02481 | 0.10077 | 0.00912 | -0.16930 | -0.04129 | 0.00151 |
| ATS8m | 0.8729 | 0.0717 | 0.01113 | 0.1216 | -0.00788 | 0.00240 | -0.01823 | 0.15126 | 0.05613 | -0.18840 | -0.04613 | 0.01326 |
| ATS1v | 0.9311 | 0.2238 | 0.00731 | 0.1050 | 0.10737 | -0.03949 | 0.00662 | 0.19917 | -0.00713 | 0.03515 | 0.05939 | 0.07764 |
| ATS2v | 0.9259 | 0.2223 | 0.03955 | 0.0901 | 0.12149 | -0.03314 | 0.00242 | 0.13546 | -0.03190 | 0.16153 | 0.07210 | 0.06745 |
| ATS3v | 0.9232 | 0.2100 | 0.06508 | 0.0958 | 0.12686 | -0.04608 | -0.01319 | 0.09257 | -0.02672 | 0.17146 | 0.07966 | 0.04206 |
| ATS4v | 0.9335 | 0.1992 | 0.09252 | 0.1066 | 0.10301 | -0.04061 | -0.01000 | 0.06793 | -0.01206 | 0.09666 | 0.04681 | 0.02293 |
| ATS5v | 0.9296 | 0.1906 | 0.07930 | 0.1188 | 0.08106 | 0.00332 | -0.00927 | 0.06510 | -0.01826 | -0.03070 | 0.01580 | 0.03614 |
| ATS6v | 0.9164 | 0.1861 | 0.07831 | 0.1241 | 0.05199 | 0.01528 | 0.01047 | 0.08822 | 0.01058 | -0.11501 | -0.00019 | 0.04919 |
| ATS7v | 0.8956 | 0.1509 | 0.04081 | 0.1312 | 0.02697 | 0.02111 | 0.02042 | 0.12891 | 0.03083 | -0.16365 | -0.01552 | 0.04988 |
| ATS8v | 0.8671 | 0.1355 | 0.04570 | 0.1318 | 0.01919 | 0.01447 | 0.02641 | 0.18440 | 0.07429 | -0.17520 | -0.02174 | 0.05136 |
| ATS1e | 0.9610 | 0.0476 | 0.00018 | 0.1061 | 0.06271 | 0.01302 | 0.02162 | 0.22080 | 0.00746 | 0.02603 | 0.02118 | -0.00314 |
| ATS2e | 0.9580 | 0.0307 | 0.00077 | 0.0841 | 0.06053 | -0.03477 | 0.02368 | 0.17422 | -0.00230 | 0.14290 | 0.03447 | -0.05705 |
| ATS3e | 0.9438 | 0.0206 | 0.01390 | 0.0826 | 0.12224 | -0.02479 | -0.00070 | 0.12797 | -0.00428 | 0.17106 | 0.07066 | -0.06838 |
| ATS4e | 0.9460 | 0.0316 | 0.04337 | 0.0974 | 0.10638 | -0.03808 | 0.01104 | 0.07880 | -0.02219 | 0.09221 | 0.04183 | -0.07817 |
| ATS5e | 0.9387 | 0.0605 | 0.02756 | 0.1078 | 0.08232 | 0.00002 | -0.00546 | 0.06967 | -0.00880 | -0.02749 | -0.00783 | -0.05262 |
| ATS6e | 0.9238 | 0.0864 | 0.04272 | 0.1156 | 0.05453 | 0.01598 | 0.01776 | 0.07414 | 0.00578 | -0.12060 | -0.02415 | -0.03673 |
| ATS7e | 0.9034 | 0.0807 | 0.00959 | 0.1296 | 0.03296 | 0.02338 | 0.01813 | 0.10996 | 0.02361 | -0.16265 | -0.04447 | -0.00399 |
| ATS8e | 0.8779 | 0.0702 | 0.01324 | 0.1270 | 0.02737 | 0.01281 | 0.02589 | 0.16128 | 0.06950 | -0.18139 | -0.04674 | 0.00856 |
| ATS1p | 0.9205 | 0.2391 | 0.02107 | 0.1047 | 0.05057 | -0.08788 | -0.01271 | 0.20395 | -0.01273 | 0.02946 | 0.10042 | 0.06431 |
| ATS2p | 0.9170 | 0.2384 | 0.05547 | 0.0892 | 0.07562 | -0.06904 | -0.02229 | 0.13558 | -0.03868 | 0.15963 | 0.10613 | 0.06852 |
| ATS3p | 0.9139 | 0.2249 | 0.07744 | 0.0952 | 0.08512 | -0.07850 | -0.03722 | 0.09195 | -0.03359 | 0.17037 | 0.10664 | 0.04825 |
| ATS4p | 0.9254 | 0.2122 | 0.10160 | 0.1057 | 0.06834 | -0.06538 | -0.03300 | 0.06649 | -0.01824 | 0.09705 | 0.06521 | 0.03024 |
| ATS5p | 0.9254 | 0.2008 | 0.08655 | 0.1182 | 0.05645 | -0.01285 | -0.02575 | 0.06406 | -0.02392 | -0.02933 | 0.03046 | 0.04403 |
| ATS6p | 0.9139 | 0.1940 | 0.08436 | 0.1238 | 0.03304 | 0.00396 | -0.00398 | 0.08764 | 0.00714 | -0.11376 | 0.00880 | 0.05717 |
| ATS7p | 0.8929 | 0.1577 | 0.04466 | 0.1312 | 0.01378 | 0.01289 | 0.01121 | 0.12846 | 0.02704 | -0.16530 | -0.00981 | 0.05422 |
| ATS8p | 0.8646 | 0.1420 | 0.04912 | 0.1323 | 0.00835 | 0.00912 | 0.01669 | 0.18448 | 0.07066 | -0.17546 | -0.01796 | 0.05561 |
| MATS1m | 0.0789 | 0.2345 | -0.08199 | -0.0091 | -0.03372 | -0.41656 | -0.11045 | -0.05110 | -0.07759 | -0.04248 | 0.20905 | 0.01103 |
| MATS2m | 0.1151 | 0.1188 | 0.07044 | -0.0238 | -0.03730 | -0.18302 | 0.11499 | -0.11000 | 0.03911 | -0.02595 | -0.31484 | 0.13342 |
| MATS3m | 0.2107 | 0.0687 | -0.00619 | 0.0192 | 0.10960 | 0.15077 | -0.02277 | 0.02743 | 0.20791 | -0.07038 | 0.06518 | 0.03272 |
| MATS4m | 0.1864 | -0.0412 | 0.07398 | 0.0556 | 0.09227 | -0.03998 | -0.03530 | -0.04079 | -0.12031 | 0.06089 | 0.06912 | 0.15566 |
| MATS5m | 0.0771 | -0.0868 | 0.01690 | 0.0112 | 0.02001 | -0.03369 | -0.08177 | 0.07719 | 0.08708 | -0.08764 | 0.12320 | 0.03092 |
| MATS6m | 0.0538 | -0.1838 | 0.05896 | 0.0148 | 0.10282 | -0.07770 | -0.00061 | 0.02321 | 0.05499 | -0.04899 | 0.07597 | -0.02600 |
| MATS7m | -0.0608 | -0.0592 | -0.01965 | -0.0001 | 0.02225 | -0.02733 | 0.00244 | 0.03304 | 0.00324 | -0.02132 | 0.12764 | -0.00465 |
| MATS8m | -0.0227 | -0.0738 | 0.01926 | -0.0408 | -0.01438 | -0.01260 | -0.02642 | 0.07607 | 0.02579 | 0.05484 | 0.01874 | 0.02346 |
| MATS1v | 0.0391 | 0.2106 | -0.12447 | -0.0542 | 0.25605 | -0.33154 | -0.18960 | -0.02570 | -0.03112 | 0.08518 | 0.05305 | 0.19550 |
| MATS2v | 0.0617 | 0.0319 | 0.03735 | -0.0540 | -0.44643 | -0.30990 | 0.00562 | -0.12966 | -0.04628 | 0.00239 | -0.20893 | 0.11583 |
| MATS3v | 0.2046 | 0.0539 | -0.00399 | 0.0198 | 0.11226 | 0.09721 | -0.15665 | 0.03448 | 0.18055 | -0.01259 | 0.09643 | 0.12304 |
| MATS4v | 0.1682 | -0.0017 | 0.07401 | 0.0230 | 0.14762 | 0.00308 | -0.03969 | -0.06620 | -0.09731 | 0.07472 | 0.11011 | 0.21572 |
| MATS5v | 0.0720 | -0.0770 | 0.05412 | 0.0012 | 0.08173 | -0.01973 | -0.07152 | 0.08356 | 0.12852 | -0.05389 | 0.12062 | 0.07614 |
| MATS6v | 0.0464 | -0.1808 | 0.03806 | -0.0175 | 0.03892 | -0.13090 | 0.01227 | 0.01331 | 0.03446 | -0.03897 | 0.14684 | -0.04120 |
| MATS7v | -0.0708 | -0.0635 | -0.01200 | 0.0048 | 0.01401 | -0.00098 | 0.02407 | 0.01864 | -0.00296 | -0.00045 | 0.13217 | -0.01184 |
| MATS8v | -0.0133 | -0.0721 | 0.04054 | -0.0193 | -0.00815 | -0.01353 | 0.00350 | 0.08757 | 0.02134 | 0.06469 | 0.02650 | 0.07591 |
| MATS1e | 0.1055 | 0.2324 | -0.22084 | -0.0235 | 0.22153 | -0.33871 | 0.01981 | -0.05719 | -0.07590 | 0.04295 | 0.03885 | 0.03425 |
| MATS2e | 0.0716 | 0.0608 | 0.08468 | -0.0118 | -0.40788 | -0.31719 | 0.13107 | -0.12404 | -0.01018 | -0.06188 | -0.20024 | 0.02109 |
| MATS3e | 0.2322 | 0.0786 | 0.00308 | 0.0556 | 0.23173 | 0.18386 | -0.06620 | 0.04408 | 0.22786 | -0.08206 | 0.02732 | 0.08049 |
| MATS4e | 0.2011 | -0.0065 | 0.08697 | 0.0472 | 0.22597 | 0.04208 | 0.02888 | -0.07058 | -0.09090 | 0.04307 | 0.07728 | 0.17812 |
| MATS5e | 0.0119 | -0.0715 | 0.04385 | 0.0074 | 0.05179 | -0.03005 | -0.16032 | 0.10288 | 0.08060 | -0.04778 | 0.12377 | 0.05473 |
| MATS6e | 0.0370 | -0.1737 | 0.03582 | -0.0273 | 0.04213 | -0.09914 | -0.01804 | 0.01699 | 0.03924 | -0.02969 | 0.10418 | -0.03970 |
| MATS7e | -0.0690 | -0.0536 | -0.02295 | -0.0086 | -0.00127 | -0.02092 | -0.04502 | 0.02145 | -0.03272 | 0.01119 | 0.11097 | 0.02474 |
| MATS8e | -0.0321 | -0.0680 | 0.02418 | -0.0266 | -0.00955 | -0.02122 | -0.05940 | 0.08117 | -0.00003 | 0.05973 | 0.00250 | 0.03315 |
| MATS1p | 0.0302 | 0.1986 | -0.03589 | -0.0224 | 0.41702 | -0.21594 | -0.27339 | -0.02079 | 0.02536 | 0.01991 | -0.04048 | 0.23514 |
| MATS2p | 0.0989 | 0.0658 | 0.02461 | -0.0340 | -0.34121 | -0.25146 | 0.06511 | -0.14271 | -0.04457 | -0.04983 | -0.30368 | 0.07020 |
| MATS3p | 0.2136 | 0.0579 | -0.00182 | 0.0334 | 0.07242 | 0.11216 | -0.07383 | 0.02893 | 0.16733 | -0.05823 | 0.09388 | 0.00147 |
| MATS4p | 0.1873 | 0.0065 | 0.05490 | 0.0348 | 0.12210 | 0.00866 | 0.00531 | -0.07598 | -0.11870 | 0.03431 | 0.08770 | 0.12013 |
| MATS5p | 0.0866 | -0.0601 | 0.04251 | 0.0124 | 0.09186 | 0.00941 | 0.01970 | 0.06773 | 0.12637 | -0.08805 | 0.08631 | 0.02467 |
| MATS6p | 0.0400 | -0.1801 | 0.05602 | 0.0136 | 0.06376 | -0.11093 | 0.04811 | 0.01799 | 0.04755 | -0.05286 | 0.13175 | -0.04300 |
| MATS7p | -0.0630 | -0.0651 | -0.00710 | 0.0118 | 0.02053 | -0.00312 | 0.06232 | 0.01659 | 0.00954 | -0.01126 | 0.13634 | -0.04119 |
| MATS8p | 0.0029 | -0.0712 | 0.03226 | -0.0097 | -0.01175 | -0.01534 | 0.05357 | 0.08260 | 0.03494 | 0.05629 | 0.03410 | 0.05272 |
| GATS1m | -0.0161 | -0.2418 | 0.03017 | 0.0774 | -0.40432 | 0.21570 | 0.31574 | 0.07670 | -0.01086 | -0.09514 | 0.11519 | -0.21049 |
| GATS2m | -0.0542 | -0.2122 | -0.02917 | 0.0587 | -0.04614 | 0.12321 | -0.04150 | 0.08265 | 0.03745 | -0.16066 | 0.31174 | -0.04565 |
| GATS3m | -0.1385 | -0.1884 | -0.00563 | 0.0155 | -0.11249 | -0.03295 | 0.05172 | -0.02518 | -0.17503 | -0.06632 | 0.14811 | 0.03423 |
| GATS4m | -0.1532 | -0.0315 | -0.05936 | -0.0236 | 0.02786 | 0.04135 | 0.10572 | -0.00660 | -0.02836 | -0.08800 | 0.10281 | -0.04260 |
| GATS5m | -0.0054 | 0.0801 | -0.08785 | 0.0113 | 0.06298 | 0.03596 | 0.03598 | -0.10508 | -0.07260 | 0.11365 | -0.03856 | -0.01438 |
| GATS6m | 0.0803 | 0.1648 | -0.05222 | 0.0004 | 0.01882 | 0.11319 | -0.06727 | -0.11721 | -0.08673 | 0.05846 | -0.10521 | 0.00440 |
| GATS7m | 0.2653 | 0.1524 | -0.02336 | 0.0637 | 0.05155 | 0.06403 | -0.08118 | -0.13060 | -0.07662 | -0.02126 | -0.18900 | -0.00205 |
| GATS8m | 0.3174 | 0.1426 | -0.02180 | 0.0639 | 0.03623 | 0.01024 | -0.10694 | -0.14838 | -0.03443 | -0.10055 | -0.10958 | -0.00722 |
| GATS1v | -0.0232 | -0.2472 | 0.14630 | 0.0586 | -0.35109 | 0.45343 | 0.04037 | 0.02902 | 0.04014 | -0.15919 | 0.07511 | -0.09744 |
| GATS2v | -0.0596 | -0.1565 | -0.00774 | 0.0675 | 0.21813 | 0.27916 | -0.06449 | 0.12255 | 0.12419 | -0.21515 | 0.29439 | -0.06371 |
| GATS3v | -0.1640 | -0.1838 | 0.00533 | -0.0023 | -0.08219 | 0.02900 | 0.07634 | -0.01138 | -0.16890 | -0.11986 | 0.11012 | 0.00970 |
| GATS4v | -0.1340 | -0.0319 | -0.07899 | -0.0046 | -0.06368 | 0.01121 | 0.04531 | 0.00851 | -0.06451 | -0.12755 | 0.06947 | -0.08351 |
| GATS5v | 0.0178 | 0.0820 | -0.12804 | 0.0211 | -0.00383 | 0.01636 | 0.00412 | -0.10847 | -0.13098 | 0.08098 | -0.05908 | -0.03578 |
| GATS6v | 0.1221 | 0.1802 | -0.05297 | 0.0335 | 0.05226 | 0.12697 | -0.00209 | -0.11571 | -0.09731 | 0.03535 | -0.12437 | -0.02310 |
| GATS7v | 0.3285 | 0.1684 | -0.03605 | 0.0942 | 0.08222 | 0.06243 | -0.02432 | -0.11713 | -0.08209 | -0.03261 | -0.20276 | -0.04868 |
| GATS8v | 0.4002 | 0.1564 | -0.06390 | 0.0917 | 0.08282 | -0.00663 | 0.02331 | -0.14422 | -0.03638 | -0.11813 | -0.12139 | -0.06542 |
| GATS1e | -0.0721 | -0.2617 | 0.20893 | 0.0289 | -0.25952 | 0.41429 | -0.08388 | 0.06174 | 0.06546 | -0.11442 | 0.08547 | -0.02483 |
| GATS2e | -0.0725 | -0.1821 | -0.05127 | 0.0174 | 0.21094 | 0.27270 | -0.16236 | 0.13220 | 0.08618 | -0.15213 | 0.30020 | -0.02430 |
| GATS3e | -0.1845 | -0.2008 | 0.00533 | -0.0377 | -0.15071 | -0.02765 | 0.02481 | -0.01039 | -0.19460 | -0.05734 | 0.17530 | 0.02766 |
| GATS4e | -0.1666 | -0.0379 | -0.08016 | -0.0144 | -0.11472 | -0.00538 | 0.04542 | 0.01265 | -0.05625 | -0.09799 | 0.10548 | -0.05764 |
| GATS5e | 0.0444 | 0.0821 | -0.13035 | 0.0322 | 0.01296 | 0.03599 | 0.10726 | -0.13538 | -0.09799 | 0.08985 | -0.06579 | -0.02101 |
| GATS6e | 0.1189 | 0.1789 | -0.04350 | 0.0578 | 0.04812 | 0.12421 | 0.02526 | -0.12410 | -0.08938 | 0.02414 | -0.11232 | -0.01946 |
| GATS7e | 0.3200 | 0.1587 | -0.02827 | 0.1058 | 0.06811 | 0.07176 | -0.00057 | -0.12774 | -0.07153 | -0.03519 | -0.19288 | -0.06167 |
| GATS8e | 0.4039 | 0.1560 | -0.05092 | 0.0884 | 0.06048 | 0.00715 | 0.01556 | -0.15385 | -0.02733 | -0.12031 | -0.10729 | -0.03009 |
| GATS1p | -0.0070 | -0.2162 | 0.06300 | 0.0499 | -0.53437 | 0.26215 | 0.20626 | 0.01870 | -0.01273 | -0.12549 | 0.17590 | -0.22010 |
| GATS2p | -0.0663 | -0.1866 | 0.00762 | 0.0642 | 0.11931 | 0.22663 | -0.08909 | 0.11187 | 0.12268 | -0.18104 | 0.34943 | 0.00164 |
| GATS3p | -0.1496 | -0.1901 | -0.00596 | 0.0007 | -0.06788 | 0.01172 | 0.01730 | -0.02122 | -0.16369 | -0.09248 | 0.11610 | 0.10872 |
| GATS4p | -0.1456 | -0.0484 | -0.06644 | -0.0134 | -0.02939 | 0.00293 | 0.01015 | 0.01441 | -0.05533 | -0.09725 | 0.07967 | -0.00531 |
| GATS5p | 0.0014 | 0.0716 | -0.11097 | 0.0059 | -0.00486 | -0.00804 | -0.06162 | -0.09023 | -0.12725 | 0.10349 | -0.03194 | 0.00242 |
| GATS6p | 0.1122 | 0.1804 | -0.06304 | 0.0079 | 0.04839 | 0.12145 | -0.05192 | -0.10896 | -0.09951 | 0.04950 | -0.12609 | 0.00084 |
| GATS7p | 0.3113 | 0.1706 | -0.03465 | 0.0739 | 0.08545 | 0.05695 | -0.06726 | -0.11187 | -0.08386 | -0.02870 | -0.20976 | -0.00641 |
| GATS8p | 0.3706 | 0.1598 | -0.05304 | 0.0772 | 0.08178 | -0.00031 | -0.05094 | -0.14344 | -0.04316 | -0.10872 | -0.12439 | -0.03985 |
| EPS0 | 0.8093 | 0.0358 | 0.02932 | 0.0822 | 0.04412 | 0.03160 | 0.02429 | 0.56788 | 0.04069 | -0.00484 | 0.01594 | 0.03169 |
| EPS1 | 0.8209 | 0.0393 | 0.02694 | 0.0721 | 0.05107 | 0.01231 | 0.02757 | 0.55046 | 0.03586 | 0.06583 | 0.01137 | 0.02703 |
| EEig01x | 0.6388 | 0.0978 | 0.07565 | 0.0285 | -0.04522 | -0.16531 | 0.05513 | -0.14154 | -0.17295 | 0.56542 | 0.13190 | -0.19748 |
| EEig02x | 0.8394 | 0.1039 | 0.02229 | 0.0425 | 0.09836 | -0.13969 | 0.02125 | -0.10554 | -0.15927 | 0.26874 | 0.02252 | -0.09491 |
| EEig03x | 0.8797 | 0.0674 | -0.02408 | 0.0700 | 0.11241 | -0.08876 | -0.00068 | -0.06593 | -0.19034 | 0.15283 | 0.02869 | 0.00209 |
| EEig04x | 0.9061 | 0.1081 | -0.04522 | 0.0908 | 0.11945 | -0.05445 | 0.00627 | -0.05360 | -0.19678 | 0.06014 | 0.00455 | 0.00678 |
| EEig05x | 0.9280 | 0.1216 | -0.04430 | 0.0984 | 0.09138 | -0.02976 | 0.00573 | -0.03838 | -0.16344 | 0.02260 | -0.00160 | 0.04191 |
| EEig06x | 0.9354 | 0.1298 | -0.05594 | 0.1034 | 0.10655 | -0.01258 | 0.02708 | -0.00749 | -0.12931 | 0.00665 | 0.00298 | 0.05715 |
| EEig07x | 0.9304 | 0.1457 | -0.05141 | 0.1230 | 0.09759 | 0.00037 | 0.03858 | 0.03048 | -0.10158 | -0.00961 | 0.01042 | 0.06227 |
| EEig08x | 0.9213 | 0.1408 | -0.04713 | 0.1237 | 0.09696 | 0.01084 | 0.03336 | 0.07823 | -0.07969 | -0.01990 | -0.00422 | 0.06128 |
| EEig09x | 0.9132 | 0.1305 | -0.03463 | 0.1319 | 0.06075 | 0.00585 | 0.02178 | 0.13896 | -0.02731 | -0.00656 | -0.01149 | 0.06093 |
| EEig10x | 0.8960 | 0.1020 | -0.02780 | 0.1282 | 0.05379 | 0.00277 | 0.03370 | 0.19413 | -0.00414 | 0.00280 | -0.01250 | 0.05477 |
| EEig11x | 0.8727 | 0.0767 | -0.01269 | 0.1176 | 0.05710 | 0.00860 | 0.03816 | 0.25399 | 0.01095 | 0.01274 | -0.01138 | 0.05514 |
| EEig12x | 0.8428 | 0.0542 | -0.00213 | 0.1021 | 0.05960 | 0.01048 | 0.04252 | 0.31305 | 0.02852 | 0.02938 | -0.02521 | 0.05789 |
| EEig13x | 0.7995 | 0.0378 | 0.00748 | 0.0931 | 0.06543 | 0.00340 | 0.04392 | 0.38249 | 0.03469 | 0.04333 | -0.02213 | 0.04687 |
| EEig14x | 0.7546 | 0.0134 | 0.02150 | 0.0926 | 0.05580 | 0.01859 | 0.04369 | 0.44406 | 0.04981 | 0.06795 | -0.01738 | 0.03346 |
| EEig15x | 0.7066 | -0.0150 | 0.02983 | 0.0667 | 0.04627 | 0.02133 | 0.03013 | 0.50039 | 0.05707 | 0.08087 | -0.02357 | 0.02014 |
| EEig01d | 0.4473 | -0.0823 | 0.04085 | 0.0707 | -0.37011 | -0.37645 | 0.05892 | -0.03148 | -0.00006 | 0.26586 | -0.00691 | -0.25279 |
| EEig02d | 0.7079 | -0.1078 | 0.07662 | 0.0645 | -0.11287 | -0.31833 | -0.02713 | -0.00652 | 0.04583 | 0.19916 | -0.11024 | -0.23784 |
| EEig03d | 0.8556 | -0.0614 | 0.05375 | 0.0989 | -0.00187 | -0.21594 | -0.04041 | -0.01902 | 0.00249 | 0.13791 | -0.05798 | -0.11823 |
| EEig04d | 0.9089 | -0.0420 | 0.03976 | 0.1017 | 0.05534 | -0.14290 | -0.05660 | 0.00255 | -0.03090 | 0.07657 | -0.06317 | -0.04715 |
| EEig05d | 0.9325 | -0.0167 | 0.01977 | 0.1009 | 0.06385 | -0.09377 | -0.03821 | 0.03459 | -0.02228 | 0.06573 | -0.06388 | -0.02144 |
| EEig06d | 0.9364 | -0.0032 | 0.02335 | 0.0998 | 0.06781 | -0.07992 | -0.01748 | 0.06165 | -0.01002 | 0.04884 | -0.04913 | -0.00533 |
| EEig07d | 0.9297 | 0.0040 | 0.00885 | 0.1141 | 0.06606 | -0.05023 | -0.00367 | 0.09729 | 0.00835 | 0.03533 | -0.05544 | 0.00144 |
| EEig08d | 0.9139 | 0.0108 | 0.01494 | 0.1175 | 0.05742 | -0.02965 | 0.00144 | 0.13762 | 0.02239 | 0.01734 | -0.05028 | 0.00530 |
| EEig09d | 0.8884 | 0.0123 | 0.01671 | 0.1197 | 0.05257 | -0.01381 | 0.01348 | 0.18780 | 0.04178 | 0.01239 | -0.04400 | 0.02646 |
| EEig10d | 0.8493 | -0.0004 | 0.01217 | 0.1246 | 0.04420 | -0.01697 | 0.01264 | 0.24407 | 0.07668 | 0.01412 | -0.04493 | 0.01718 |
| EEig11d | 0.8005 | -0.0149 | 0.02083 | 0.1124 | 0.04202 | -0.00690 | 0.01420 | 0.30307 | 0.09665 | 0.03305 | -0.04943 | 0.01822 |
| EEig12d | 0.7319 | -0.0415 | 0.03802 | 0.0970 | 0.04067 | -0.00194 | 0.02108 | 0.36666 | 0.11943 | 0.04973 | -0.07182 | 0.02131 |
| EEig13d | 0.6396 | -0.0694 | 0.04990 | 0.0758 | 0.05470 | -0.01446 | 0.01632 | 0.44293 | 0.13704 | 0.07613 | -0.06127 | -0.00600 |
| EEig14d | 0.5468 | -0.1000 | 0.05651 | 0.0490 | 0.03822 | 0.00554 | 0.01684 | 0.50637 | 0.16042 | 0.10673 | -0.04576 | -0.03071 |
| EEig15d | 0.4520 | -0.1417 | 0.04720 | 0.0200 | 0.02323 | -0.00032 | -0.00606 | 0.55564 | 0.16675 | 0.12325 | -0.07226 | -0.06590 |
| EEig01r | 0.6489 | 0.1393 | 0.13722 | 0.0111 | 0.05810 | -0.16659 | 0.06194 | -0.09821 | 0.07981 | 0.60737 | 0.08976 | -0.16187 |
| EEig02r | 0.8412 | 0.1261 | 0.06181 | 0.0387 | 0.14379 | -0.12603 | 0.04488 | -0.07154 | 0.03354 | 0.32429 | 0.01632 | -0.09287 |
| EEig03r | 0.8976 | 0.0956 | 0.02145 | 0.0738 | 0.14506 | -0.06470 | 0.02020 | -0.04576 | -0.02943 | 0.18859 | 0.03558 | -0.00023 |
| EEig04r | 0.9241 | 0.1316 | -0.00749 | 0.0939 | 0.14569 | -0.03186 | 0.02364 | -0.03475 | -0.06381 | 0.08290 | 0.01080 | 0.00704 |
| EEig05r | 0.9383 | 0.1423 | -0.01214 | 0.0978 | 0.11681 | -0.01497 | 0.01943 | -0.01355 | -0.05528 | 0.04402 | 0.01041 | 0.03651 |
| EEig06r | 0.9392 | 0.1466 | -0.02644 | 0.1077 | 0.12329 | 0.00102 | 0.03918 | 0.01568 | -0.03022 | 0.02788 | 0.00991 | 0.04964 |
| EEig07r | 0.9297 | 0.1534 | -0.02247 | 0.1245 | 0.11540 | 0.00679 | 0.04817 | 0.05479 | -0.00604 | 0.01101 | 0.01029 | 0.05689 |
| EEig08r | 0.9156 | 0.1517 | -0.02000 | 0.1251 | 0.11299 | 0.01810 | 0.04872 | 0.10638 | 0.01452 | 0.00079 | -0.00060 | 0.05724 |
| EEig09r | 0.8990 | 0.1447 | -0.00819 | 0.1364 | 0.08173 | 0.01984 | 0.03585 | 0.16351 | 0.05564 | 0.00911 | -0.00121 | 0.05631 |
| EEig10r | 0.8728 | 0.1152 | 0.00322 | 0.1334 | 0.08001 | 0.01527 | 0.04364 | 0.22218 | 0.08334 | 0.01688 | -0.01068 | 0.05264 |
| EEig11r | 0.8417 | 0.0907 | 0.01359 | 0.1114 | 0.08048 | 0.02019 | 0.05006 | 0.28233 | 0.09797 | 0.03174 | -0.01216 | 0.04971 |
| EEig12r | 0.8007 | 0.0653 | 0.02602 | 0.0960 | 0.07891 | 0.01774 | 0.05803 | 0.34936 | 0.11299 | 0.04895 | -0.02578 | 0.05033 |
| EEig13r | 0.7432 | 0.0436 | 0.03216 | 0.0822 | 0.08460 | 0.01023 | 0.05418 | 0.42380 | 0.12713 | 0.06889 | -0.02599 | 0.03621 |
| EEig14r | 0.6839 | 0.0144 | 0.04579 | 0.0702 | 0.07646 | 0.02626 | 0.05548 | 0.48854 | 0.14180 | 0.09758 | -0.02250 | 0.01550 |
| EEig15r | 0.6210 | -0.0152 | 0.05346 | 0.0495 | 0.06414 | 0.02666 | 0.03789 | 0.54464 | 0.15044 | 0.11699 | -0.03266 | -0.00637 |
| ESpm02u | 0.9542 | 0.1016 | 0.01402 | 0.0879 | 0.09217 | -0.02826 | 0.02754 | 0.15543 | -0.00846 | 0.15702 | 0.04724 | -0.01028 |
| ESpm03u | 0.8505 | 0.0427 | 0.11625 | 0.0254 | 0.03272 | -0.12016 | 0.04207 | 0.08987 | 0.01500 | 0.37757 | 0.05337 | -0.18605 |
| ESpm04u | 0.9150 | 0.1008 | 0.06148 | 0.0592 | 0.07073 | -0.07658 | 0.03740 | 0.09440 | 0.01071 | 0.30404 | 0.07715 | -0.07776 |
| ESpm05u | 0.8130 | 0.0756 | 0.09893 | 0.0267 | 0.02439 | -0.11065 | 0.04907 | 0.02446 | 0.00533 | 0.42787 | 0.07393 | -0.20080 |
| ESpm06u | 0.8525 | 0.1054 | 0.09485 | 0.0365 | 0.04535 | -0.11535 | 0.04656 | 0.03936 | 0.03234 | 0.41549 | 0.09754 | -0.12779 |
| ESpm07u | 0.7732 | 0.0960 | 0.09116 | 0.0245 | 0.01795 | -0.11206 | 0.05317 | -0.01762 | 0.00400 | 0.46416 | 0.08274 | -0.21070 |
| ESpm08u | 0.8003 | 0.1091 | 0.11026 | 0.0245 | 0.03002 | -0.13602 | 0.05142 | -0.00123 | 0.04315 | 0.47912 | 0.10855 | -0.15412 |
| ESpm09u | 0.7416 | 0.1073 | 0.08832 | 0.0223 | 0.01369 | -0.11574 | 0.05524 | -0.04600 | 0.00509 | 0.48892 | 0.08684 | -0.21724 |
| ESpm10u | 0.7645 | 0.1115 | 0.11651 | 0.0190 | 0.02183 | -0.14577 | 0.05356 | -0.02935 | 0.04659 | 0.51391 | 0.11456 | -0.16728 |
| ESpm11u | 0.7184 | 0.1136 | 0.08738 | 0.0209 | 0.01097 | -0.11890 | 0.05620 | -0.06565 | 0.00618 | 0.50550 | 0.08892 | -0.22139 |
| ESpm12u | 0.7406 | 0.1131 | 0.11899 | 0.0164 | 0.01739 | -0.15040 | 0.05436 | -0.04906 | 0.04694 | 0.53395 | 0.11797 | -0.17417 |
| ESpm13u | 0.7018 | 0.1171 | 0.08710 | 0.0201 | 0.00924 | -0.12111 | 0.05662 | -0.07971 | 0.00669 | 0.51667 | 0.09010 | -0.22396 |
| ESpm14u | 0.7242 | 0.1143 | 0.11998 | 0.0153 | 0.01487 | -0.15266 | 0.05460 | -0.06329 | 0.04623 | 0.54629 | 0.11994 | -0.17801 |
| ESpm15u | 0.6897 | 0.1192 | 0.08703 | 0.0197 | 0.00811 | -0.12252 | 0.05677 | -0.09006 | 0.00675 | 0.52434 | 0.09079 | -0.22556 |
| ESpm01x | 0.9474 | 0.0960 | -0.04821 | 0.1127 | 0.06608 | -0.01328 | 0.02525 | 0.19621 | -0.15397 | -0.01005 | 0.01602 | 0.03390 |
| ESpm02x | 0.9573 | 0.0962 | -0.02209 | 0.0974 | 0.07691 | -0.03825 | 0.02838 | 0.16252 | -0.10396 | 0.09760 | 0.02763 | 0.00146 |
| ESpm03x | 0.9454 | 0.0848 | -0.00773 | 0.0827 | 0.06393 | -0.06773 | 0.02960 | 0.13597 | -0.14996 | 0.15652 | 0.03793 | -0.03975 |
| ESpm04x | 0.9304 | 0.0796 | 0.00887 | 0.0710 | 0.04987 | -0.09026 | 0.03325 | 0.11040 | -0.15911 | 0.21525 | 0.05033 | -0.07496 |
| ESpm05x | 0.9104 | 0.0763 | 0.02300 | 0.0610 | 0.03491 | -0.10844 | 0.03639 | 0.08526 | -0.17035 | 0.26541 | 0.06301 | -0.10539 |
| ESpm06x | 0.8892 | 0.0760 | 0.03391 | 0.0532 | 0.02118 | -0.12224 | 0.03946 | 0.06151 | -0.17602 | 0.30821 | 0.07482 | -0.12922 |
| ESpm07x | 0.8676 | 0.0772 | 0.04197 | 0.0471 | 0.00917 | -0.13266 | 0.04213 | 0.03950 | -0.18004 | 0.34408 | 0.08520 | -0.14787 |
| ESpm08x | 0.8469 | 0.0794 | 0.04772 | 0.0425 | -0.00092 | -0.14046 | 0.04437 | 0.01963 | -0.18263 | 0.37388 | 0.09410 | -0.16222 |
| ESpm09x | 0.8274 | 0.0818 | 0.05180 | 0.0389 | -0.00925 | -0.14627 | 0.04618 | 0.00189 | -0.18448 | 0.39849 | 0.10163 | -0.17328 |
| ESpm10x | 0.8096 | 0.0843 | 0.05467 | 0.0362 | -0.01603 | -0.15058 | 0.04767 | -0.01378 | -0.18582 | 0.41883 | 0.10786 | -0.18183 |
| ESpm11x | 0.7935 | 0.0865 | 0.05670 | 0.0342 | -0.02152 | -0.15375 | 0.04883 | -0.02757 | -0.18685 | 0.43565 | 0.11307 | -0.18845 |
| ESpm12x | 0.7790 | 0.0886 | 0.05817 | 0.0326 | -0.02594 | -0.15608 | 0.04972 | -0.03966 | -0.18771 | 0.44960 | 0.11734 | -0.19364 |
| ESpm13x | 0.7661 | 0.0903 | 0.05923 | 0.0315 | -0.02951 | -0.15781 | 0.05042 | -0.05026 | -0.18843 | 0.46121 | 0.12089 | -0.19772 |
| ESpm14x | 0.7547 | 0.0918 | 0.06002 | 0.0306 | -0.03243 | -0.15906 | 0.05098 | -0.05958 | -0.18906 | 0.47090 | 0.12383 | -0.20096 |
| ESpm15x | 0.7445 | 0.0930 | 0.06061 | 0.0300 | -0.03478 | -0.15997 | 0.05140 | -0.06779 | -0.18965 | 0.47907 | 0.12628 | -0.20354 |
| ESpm01d | 0.6777 | -0.3745 | 0.08134 | 0.0994 | -0.08014 | -0.19088 | -0.08899 | 0.23567 | 0.02762 | -0.02792 | -0.18399 | -0.20172 |
| ESpm02d | 0.9412 | 0.0063 | 0.02395 | 0.0969 | 0.00946 | -0.12655 | 0.00449 | 0.19256 | 0.00190 | 0.12972 | -0.00655 | -0.06266 |
| ESpm03d | 0.7866 | -0.1782 | 0.10376 | 0.0754 | -0.12592 | -0.27247 | -0.01211 | 0.16614 | 0.04122 | 0.16903 | -0.09015 | -0.21685 |
| ESpm04d | 0.8298 | -0.0880 | 0.05870 | 0.0814 | -0.13178 | -0.27030 | 0.01332 | 0.15068 | 0.02056 | 0.20026 | -0.04749 | -0.18194 |
| ESpm05d | 0.7312 | -0.1403 | 0.08416 | 0.0693 | -0.19759 | -0.32402 | 0.01745 | 0.11680 | 0.03342 | 0.21748 | -0.07221 | -0.24220 |
| ESpm06d | 0.7193 | -0.1123 | 0.06532 | 0.0713 | -0.22311 | -0.33531 | 0.03003 | 0.10298 | 0.02612 | 0.22982 | -0.05532 | -0.23838 |
| ESpm07d | 0.6674 | -0.1233 | 0.07035 | 0.0665 | -0.25227 | -0.35316 | 0.03492 | 0.07958 | 0.02730 | 0.23646 | -0.05990 | -0.25958 |
| ESpm08d | 0.6458 | -0.1120 | 0.06175 | 0.0671 | -0.27203 | -0.36102 | 0.04162 | 0.06752 | 0.02408 | 0.24144 | -0.05122 | -0.26065 |
| ESpm09d | 0.6166 | -0.1128 | 0.06136 | 0.0652 | -0.28682 | -0.36765 | 0.04477 | 0.05220 | 0.02202 | 0.24435 | -0.05011 | -0.26865 |
| ESpm10d | 0.5982 | -0.1071 | 0.05688 | 0.0655 | -0.29923 | -0.37182 | 0.04836 | 0.04268 | 0.01989 | 0.24659 | -0.04486 | -0.26967 |
| ESpm11d | 0.5800 | -0.1057 | 0.05544 | 0.0649 | -0.30769 | -0.37458 | 0.05020 | 0.03232 | 0.01742 | 0.24807 | -0.04253 | -0.27285 |
| ESpm12d | 0.5664 | -0.1025 | 0.05285 | 0.0650 | -0.31524 | -0.37664 | 0.05214 | 0.02503 | 0.01566 | 0.24930 | -0.03901 | -0.27331 |
| ESpm13d | 0.5542 | -0.1009 | 0.05147 | 0.0649 | -0.32045 | -0.37785 | 0.05320 | 0.01773 | 0.01350 | 0.25022 | -0.03675 | -0.27457 |
| ESpm14d | 0.5443 | -0.0988 | 0.04984 | 0.0651 | -0.32511 | -0.37883 | 0.05428 | 0.01216 | 0.01198 | 0.25106 | -0.03422 | -0.27465 |
| ESpm15d | 0.5357 | -0.0975 | 0.04875 | 0.0651 | -0.32844 | -0.37936 | 0.05488 | 0.00683 | 0.01023 | 0.25175 | -0.03232 | -0.27507 |
| ESpm01r | 0.9497 | 0.1335 | -0.00171 | 0.1122 | 0.11324 | -0.00531 | 0.05698 | 0.20974 | 0.00929 | 0.02948 | 0.01380 | 0.02298 |
| ESpm02r | 0.9554 | 0.1140 | 0.00901 | 0.0945 | 0.10126 | -0.02724 | 0.03788 | 0.16448 | -0.00437 | 0.12815 | 0.03702 | -0.00255 |
| ESpm03r | 0.9403 | 0.1103 | 0.03755 | 0.0771 | 0.09953 | -0.06075 | 0.04929 | 0.14229 | 0.00843 | 0.20544 | 0.04353 | -0.05054 |
| ESpm04r | 0.9218 | 0.1071 | 0.05739 | 0.0647 | 0.09233 | -0.08083 | 0.05243 | 0.11627 | 0.01466 | 0.26913 | 0.05520 | -0.08007 |
| ESpm05r | 0.8980 | 0.1070 | 0.07508 | 0.0535 | 0.08564 | -0.09869 | 0.05685 | 0.09242 | 0.02331 | 0.32491 | 0.06465 | -0.10644 |
| ESpm06r | 0.8744 | 0.1091 | 0.08749 | 0.0450 | 0.07901 | -0.11181 | 0.05990 | 0.07020 | 0.03002 | 0.36970 | 0.07295 | -0.12494 |
| ESpm07r | 0.8515 | 0.1120 | 0.09654 | 0.0382 | 0.07332 | -0.12202 | 0.06235 | 0.05014 | 0.03553 | 0.40613 | 0.07966 | -0.13893 |
| ESpm08r | 0.8306 | 0.1152 | 0.10297 | 0.0330 | 0.06860 | -0.12981 | 0.06406 | 0.03240 | 0.03980 | 0.43536 | 0.08507 | -0.14913 |
| ESpm09r | 0.8118 | 0.1183 | 0.10757 | 0.0289 | 0.06482 | -0.13582 | 0.06528 | 0.01688 | 0.04307 | 0.45888 | 0.08936 | -0.15675 |
| ESpm10r | 0.7952 | 0.1211 | 0.11093 | 0.0257 | 0.06182 | -0.14047 | 0.06612 | 0.00338 | 0.04553 | 0.47782 | 0.09277 | -0.16245 |
| ESpm11r | 0.7806 | 0.1235 | 0.11338 | 0.0233 | 0.05944 | -0.14407 | 0.06664 | -0.00835 | 0.04734 | 0.49317 | 0.09549 | -0.16676 |
| ESpm12r | 0.7680 | 0.1256 | 0.11521 | 0.0214 | 0.05758 | -0.14686 | 0.06697 | -0.01854 | 0.04867 | 0.50570 | 0.09766 | -0.17007 |
| ESpm13r | 0.7570 | 0.1273 | 0.11661 | 0.0200 | 0.05609 | -0.14906 | 0.06717 | -0.02741 | 0.04961 | 0.51598 | 0.09942 | -0.17263 |
| ESpm14r | 0.7474 | 0.1287 | 0.11768 | 0.0189 | 0.05492 | -0.15078 | 0.06726 | -0.03517 | 0.05027 | 0.52450 | 0.10085 | -0.17463 |
| ESpm15r | 0.7390 | 0.1299 | 0.11853 | 0.0180 | 0.05398 | -0.15213 | 0.06727 | -0.04198 | 0.05070 | 0.53159 | 0.10201 | -0.17622 |
| MWC01 | 0.8205 | 0.0349 | 0.03149 | 0.0714 | 0.05283 | 0.01333 | 0.02627 | 0.55111 | 0.03874 | 0.06859 | 0.01369 | 0.02300 |
| MWC02 | 0.9587 | 0.1070 | 0.00927 | 0.0959 | 0.09072 | -0.01699 | 0.02609 | 0.17359 | -0.00288 | 0.10706 | 0.04128 | 0.00452 |
| MWC03 | 0.9526 | 0.1050 | 0.00271 | 0.0923 | 0.10682 | -0.01912 | 0.02311 | 0.13577 | -0.01727 | 0.17055 | 0.05312 | 0.00613 |
| MWC04 | 0.9435 | 0.1021 | 0.01021 | 0.0853 | 0.10863 | -0.03010 | 0.02487 | 0.10833 | -0.02356 | 0.22445 | 0.05948 | -0.00775 |
| MWC05 | 0.9318 | 0.0998 | 0.00708 | 0.0822 | 0.11847 | -0.03196 | 0.02194 | 0.08047 | -0.03248 | 0.26832 | 0.06529 | -0.00801 |
| MWC06 | 0.9204 | 0.0982 | 0.01332 | 0.0773 | 0.11857 | -0.03918 | 0.02347 | 0.05997 | -0.03610 | 0.30500 | 0.06929 | -0.01786 |
| MWC07 | 0.9086 | 0.0966 | 0.01128 | 0.0752 | 0.12496 | -0.04049 | 0.02099 | 0.03947 | -0.04195 | 0.33458 | 0.07186 | -0.01788 |
| MWC08 | 0.8979 | 0.0957 | 0.01636 | 0.0717 | 0.12420 | -0.04564 | 0.02240 | 0.02431 | -0.04387 | 0.36002 | 0.07445 | -0.02519 |
| MWC09 | 0.8876 | 0.0946 | 0.01474 | 0.0703 | 0.12865 | -0.04655 | 0.02035 | 0.00911 | -0.04781 | 0.38035 | 0.07541 | -0.02489 |
| MWC10 | 0.8783 | 0.0942 | 0.01893 | 0.0677 | 0.12753 | -0.05044 | 0.02163 | -0.00223 | -0.04874 | 0.39858 | 0.07716 | -0.03057 |
| TWC | 0.8636 | 0.0456 | 0.03528 | 0.0743 | 0.06252 | -0.00080 | 0.02563 | 0.47574 | 0.02914 | 0.09969 | 0.02363 | 0.00815 |
| SRW01 | 0.8019 | 0.0234 | 0.04909 | 0.0686 | 0.03573 | 0.00457 | 0.02431 | 0.58341 | 0.05232 | 0.01076 | 0.00771 | 0.00458 |
| SRW02 | 0.8205 | 0.0349 | 0.03149 | 0.0714 | 0.05283 | 0.01333 | 0.02627 | 0.55111 | 0.03874 | 0.06859 | 0.01369 | 0.02300 |
| SRW04 | 0.8253 | 0.0284 | 0.04819 | 0.0575 | 0.06546 | -0.01095 | 0.02714 | 0.51622 | 0.04893 | 0.17605 | 0.01360 | 0.00616 |
| SRW05 | 0.2745 | -0.0282 | 0.07663 | -0.0075 | -0.01099 | -0.07035 | 0.04858 | 0.01370 | 0.11915 | 0.45566 | -0.10496 | 0.13112 |
| SRW06 | 0.8176 | 0.0275 | 0.06339 | 0.0450 | 0.08474 | -0.02984 | 0.02577 | 0.46168 | 0.06279 | 0.28758 | 0.01691 | -0.00458 |
| SRW07 | 0.3134 | -0.0225 | 0.06873 | 0.0007 | -0.00715 | -0.07429 | 0.03878 | 0.00746 | 0.13629 | 0.51509 | -0.11085 | 0.12951 |
| SRW08 | 0.7933 | 0.0291 | 0.07996 | 0.0329 | 0.10404 | -0.04811 | 0.02339 | 0.39890 | 0.08136 | 0.38757 | 0.01495 | -0.01113 |
| SRW09 | 0.3258 | -0.0190 | 0.07252 | -0.0016 | 0.00394 | -0.07834 | 0.02635 | 0.00427 | 0.14502 | 0.54169 | -0.10209 | 0.11823 |
| SRW10 | 0.7558 | 0.0310 | 0.09700 | 0.0211 | 0.11849 | -0.06519 | 0.02079 | 0.33714 | 0.10147 | 0.46739 | 0.00715 | -0.01390 |
| MPC01 | 0.8205 | 0.0349 | 0.03149 | 0.0714 | 0.05283 | 0.01333 | 0.02627 | 0.55111 | 0.03874 | 0.06859 | 0.01369 | 0.02300 |
| MPC02 | 0.8233 | 0.0279 | 0.05331 | 0.0515 | 0.07104 | -0.01644 | 0.02704 | 0.50462 | 0.05166 | 0.20817 | 0.01444 | -0.00004 |
| MPC03 | 0.8164 | 0.0237 | 0.04814 | 0.0518 | 0.10988 | -0.00605 | 0.01990 | 0.42915 | 0.05590 | 0.32140 | 0.02053 | 0.01532 |
| MPC04 | 0.7960 | 0.0257 | 0.05408 | 0.0499 | 0.12478 | -0.00749 | 0.02185 | 0.36628 | 0.04836 | 0.39439 | 0.00867 | 0.02453 |
| MPC05 | 0.7634 | 0.0300 | 0.05685 | 0.0420 | 0.13960 | 0.00068 | 0.01794 | 0.33087 | 0.04679 | 0.43127 | 0.00510 | 0.02184 |
| MPC06 | 0.7220 | 0.0249 | 0.07785 | 0.0341 | 0.14023 | -0.00798 | 0.02260 | 0.30425 | 0.06760 | 0.46177 | -0.00952 | 0.01180 |
| MPC07 | 0.6829 | 0.0296 | 0.07985 | 0.0315 | 0.13698 | -0.00317 | 0.02429 | 0.28495 | 0.07804 | 0.47342 | -0.02035 | 0.01630 |
| MPC08 | 0.6428 | 0.0266 | 0.08381 | 0.0250 | 0.13775 | -0.00087 | 0.02555 | 0.27827 | 0.08790 | 0.47801 | -0.02681 | 0.01837 |
| MPC09 | 0.6084 | 0.0164 | 0.08772 | 0.0162 | 0.13573 | -0.00027 | 0.02576 | 0.28351 | 0.09519 | 0.47241 | -0.02850 | 0.01555 |
| MPC10 | 0.5723 | 0.0036 | 0.09224 | 0.0097 | 0.12516 | 0.00301 | 0.02550 | 0.29564 | 0.09802 | 0.45975 | -0.03007 | 0.01616 |
| piPC01 | 0.9474 | 0.0960 | -0.04821 | 0.1127 | 0.06608 | -0.01328 | 0.02525 | 0.19621 | -0.15397 | -0.01005 | 0.01602 | 0.03390 |
| piPC02 | 0.9169 | 0.0838 | -0.06644 | 0.0964 | 0.05987 | -0.02161 | 0.01707 | 0.12230 | -0.30010 | 0.07519 | 0.05104 | 0.00205 |
| piPC03 | 0.8437 | 0.0880 | -0.10560 | 0.0991 | 0.12382 | 0.02800 | -0.01575 | 0.02103 | -0.41679 | 0.12525 | 0.08848 | 0.05018 |
| piPC04 | 0.7766 | 0.0984 | -0.12455 | 0.1009 | 0.13548 | 0.05330 | -0.02422 | -0.05175 | -0.47982 | 0.12596 | 0.08734 | 0.07829 |
| piPC05 | 0.7324 | 0.1163 | -0.13814 | 0.0962 | 0.16482 | 0.07721 | -0.03964 | -0.08716 | -0.51285 | 0.09880 | 0.09119 | 0.08102 |
| piPC06 | 0.7491 | 0.1204 | -0.10350 | 0.0928 | 0.16102 | 0.06456 | -0.02903 | -0.11099 | -0.47509 | 0.10850 | 0.07687 | 0.06043 |
| piPC07 | 0.7572 | 0.1484 | -0.11629 | 0.1009 | 0.14238 | 0.08004 | -0.01180 | -0.12631 | -0.44695 | 0.08754 | 0.05065 | 0.07302 |
| piPC08 | 0.7654 | 0.1603 | -0.11161 | 0.1149 | 0.12962 | 0.08834 | -0.00799 | -0.12723 | -0.41229 | 0.06787 | 0.02801 | 0.09977 |
| piPC09 | 0.7830 | 0.1605 | -0.12897 | 0.1031 | 0.11575 | 0.07602 | -0.00629 | -0.11736 | -0.35069 | 0.04207 | 0.02492 | 0.11894 |
| piPC10 | 0.7920 | 0.1626 | -0.12115 | 0.1048 | 0.12002 | 0.05593 | 0.01661 | -0.09361 | -0.30155 | 0.02193 | 0.02999 | 0.13257 |
| TPC | 0.7886 | 0.0617 | 0.00935 | 0.0778 | 0.08095 | 0.02891 | 0.03259 | 0.52163 | 0.04300 | 0.15727 | 0.00983 | 0.04891 |
| piID | 0.7654 | 0.0706 | -0.02014 | 0.0873 | 0.06695 | 0.04904 | 0.03485 | 0.49251 | -0.11633 | 0.06716 | 0.01877 | 0.07447 |
| PCR | 0.0514 | 0.0860 | -0.21518 | 0.0415 | 0.03176 | 0.09704 | -0.02625 | -0.10273 | -0.78496 | -0.27654 | 0.04142 | 0.13655 |
| PCD | 0.4672 | 0.0700 | -0.08620 | 0.0831 | 0.01149 | 0.08302 | 0.02942 | 0.26593 | -0.47091 | -0.17521 | 0.03482 | 0.11377 |
| CID | 0.8122 | 0.0328 | 0.03611 | 0.0727 | 0.04484 | 0.01381 | 0.02533 | 0.56744 | 0.04279 | 0.03061 | 0.01170 | 0.01774 |
| BID | 0.7995 | 0.0223 | 0.04911 | 0.0677 | 0.03590 | 0.00464 | 0.02430 | 0.58664 | 0.05202 | 0.01478 | 0.00816 | 0.00490 |
| BEHm1 | 0.0990 | 0.0561 | -0.01111 | 0.0024 | -0.34964 | -0.17086 | -0.12734 | -0.03100 | -0.19110 | 0.07655 | 0.18949 | -0.04647 |
| BEHm2 | 0.3817 | 0.0986 | -0.03770 | 0.0393 | -0.21137 | -0.16799 | -0.11817 | -0.02625 | -0.15672 | -0.03398 | 0.12737 | 0.01413 |
| BEHm3 | 0.5954 | 0.1208 | -0.00569 | 0.0873 | -0.16467 | -0.13710 | -0.10947 | 0.02143 | -0.07286 | -0.07205 | 0.08524 | 0.04908 |
| BEHm4 | 0.8905 | 0.1849 | 0.02322 | 0.1238 | -0.08046 | -0.05768 | -0.11161 | 0.05043 | -0.02853 | -0.09771 | 0.05596 | 0.06124 |
| BEHm5 | 0.9053 | 0.1828 | 0.05153 | 0.1301 | -0.03156 | -0.04613 | -0.08278 | 0.02773 | -0.00835 | -0.08824 | 0.05220 | 0.03302 |
| BEHm6 | 0.9167 | 0.1773 | 0.07340 | 0.1154 | 0.00878 | -0.04248 | -0.04014 | 0.01460 | 0.01275 | -0.08565 | 0.05905 | 0.04122 |
| BEHm7 | 0.9187 | 0.1780 | 0.07352 | 0.1259 | 0.03563 | -0.02955 | -0.01562 | -0.00905 | 0.01646 | -0.10434 | 0.06288 | 0.04608 |
| BEHm8 | 0.9242 | 0.1743 | 0.07704 | 0.1376 | 0.04310 | -0.01026 | -0.01508 | 0.00679 | 0.02895 | -0.10865 | 0.04573 | 0.04753 |
| BELm1 | 0.5496 | 0.4785 | -0.00061 | 0.0838 | 0.38232 | 0.04433 | 0.07780 | -0.09585 | -0.09021 | 0.17083 | 0.03422 | 0.11130 |
| BELm2 | 0.7378 | 0.3547 | 0.07883 | 0.1440 | 0.20916 | 0.06381 | 0.11307 | -0.08421 | 0.00363 | -0.17023 | 0.06943 | 0.18232 |
| BELm3 | 0.7790 | 0.3395 | 0.15718 | 0.2085 | 0.11797 | 0.12646 | 0.07302 | -0.00713 | 0.17674 | -0.13152 | 0.03233 | 0.10796 |
| BELm4 | 0.7963 | 0.3248 | 0.19380 | 0.2024 | 0.12883 | 0.11882 | 0.08549 | 0.03379 | 0.17479 | -0.11403 | 0.02556 | 0.08243 |
| BELm5 | 0.8195 | 0.3205 | 0.18717 | 0.1698 | 0.15612 | 0.10534 | 0.10061 | 0.03816 | 0.17151 | -0.11386 | 0.01052 | 0.08170 |
| BELm6 | 0.8322 | 0.3100 | 0.17970 | 0.1475 | 0.16131 | 0.07807 | 0.10719 | 0.05554 | 0.17869 | -0.10895 | 0.03525 | 0.08973 |
| BELm7 | 0.8409 | 0.3021 | 0.15490 | 0.1464 | 0.16161 | 0.06413 | 0.10740 | 0.07654 | 0.18244 | -0.08834 | 0.02052 | 0.06797 |
| BELm8 | 0.8309 | 0.2962 | 0.15224 | 0.1466 | 0.15029 | 0.06230 | 0.09398 | 0.12545 | 0.20801 | -0.06520 | 0.01191 | 0.06082 |
| BEHv1 | 0.6164 | 0.3412 | -0.06761 | 0.0868 | 0.20547 | 0.00675 | 0.00368 | -0.14518 | -0.38680 | 0.33127 | 0.11298 | 0.05509 |
| BEHv2 | 0.8361 | 0.2831 | -0.05077 | 0.1117 | 0.11782 | -0.03883 | 0.06687 | -0.10744 | -0.16540 | -0.08168 | 0.08297 | 0.13923 |
| BEHv3 | 0.8770 | 0.2812 | 0.03474 | 0.1405 | 0.06730 | 0.00965 | 0.05723 | -0.00084 | 0.01396 | -0.10951 | 0.07889 | 0.11254 |
| BEHv4 | 0.8752 | 0.2889 | 0.06302 | 0.1584 | 0.07916 | 0.01916 | 0.04296 | 0.02657 | 0.03306 | -0.11004 | 0.04017 | 0.05625 |
| BEHv5 | 0.8885 | 0.2732 | 0.09574 | 0.1571 | 0.08645 | 0.01671 | 0.04765 | 0.00347 | 0.03192 | -0.10141 | 0.03128 | 0.04831 |
| BEHv6 | 0.8936 | 0.2633 | 0.11126 | 0.1410 | 0.10928 | 0.01528 | 0.06470 | -0.00057 | 0.04012 | -0.10446 | 0.05434 | 0.07959 |
| BEHv7 | 0.8970 | 0.2573 | 0.10150 | 0.1532 | 0.11313 | 0.01990 | 0.07370 | -0.00317 | 0.04644 | -0.12094 | 0.04349 | 0.07206 |
| BEHv8 | 0.8982 | 0.2510 | 0.09269 | 0.1690 | 0.11277 | 0.03329 | 0.06539 | 0.02865 | 0.06387 | -0.11464 | 0.03380 | 0.06185 |
| BELv1 | 0.6310 | 0.3125 | -0.05773 | 0.0753 | 0.19753 | 0.07970 | 0.10099 | -0.11896 | -0.20060 | 0.22270 | 0.09532 | -0.02460 |
| BELv2 | 0.8220 | 0.2459 | -0.00982 | 0.1173 | 0.08830 | 0.06864 | 0.12275 | -0.07788 | -0.04739 | -0.16155 | 0.05478 | 0.09618 |
| BELv3 | 0.8501 | 0.2547 | 0.07207 | 0.1815 | 0.01981 | 0.11113 | 0.08664 | -0.00897 | 0.12747 | -0.14692 | 0.03234 | 0.03582 |
| BELv4 | 0.8618 | 0.2374 | 0.12504 | 0.1953 | 0.04413 | 0.08071 | 0.08582 | 0.02904 | 0.14491 | -0.12788 | 0.01725 | 0.00327 |
| BELv5 | 0.8712 | 0.2395 | 0.14056 | 0.1727 | 0.09083 | 0.08281 | 0.10093 | 0.04081 | 0.14491 | -0.12407 | 0.00068 | 0.01423 |
| BELv6 | 0.8774 | 0.2283 | 0.15594 | 0.1563 | 0.11880 | 0.05908 | 0.11052 | 0.05876 | 0.15868 | -0.11221 | 0.02140 | 0.03165 |
| BELv7 | 0.8790 | 0.2214 | 0.13824 | 0.1469 | 0.12652 | 0.03810 | 0.11113 | 0.08095 | 0.17031 | -0.09916 | 0.01766 | 0.02861 |
| BELv8 | 0.8707 | 0.2166 | 0.12884 | 0.1492 | 0.12069 | 0.03415 | 0.09834 | 0.12671 | 0.17854 | -0.08417 | 0.00993 | 0.02495 |
| BEHe1 | 0.6749 | 0.2226 | -0.08111 | 0.0823 | 0.11269 | 0.05677 | 0.06716 | -0.12596 | -0.32493 | 0.40251 | 0.07943 | 0.02799 |
| BEHe2 | 0.8717 | 0.2182 | -0.05566 | 0.1105 | 0.06267 | -0.00185 | 0.10064 | -0.10582 | -0.11825 | -0.09004 | 0.03907 | 0.08706 |
| BEHe3 | 0.8931 | 0.2216 | 0.03259 | 0.1512 | 0.02410 | 0.04356 | 0.06609 | 0.00074 | 0.04549 | -0.12961 | 0.04386 | 0.05307 |
| BEHe4 | 0.8943 | 0.2248 | 0.07325 | 0.1733 | 0.06337 | 0.03879 | 0.06712 | 0.04224 | 0.07411 | -0.13100 | 0.01435 | 0.00597 |
| BEHe5 | 0.9016 | 0.2176 | 0.09672 | 0.1601 | 0.09786 | 0.03662 | 0.06711 | 0.03035 | 0.07580 | -0.10943 | 0.00918 | -0.00410 |
| BEHe6 | 0.9045 | 0.2146 | 0.11837 | 0.1442 | 0.12006 | 0.02880 | 0.08275 | 0.02997 | 0.08266 | -0.10533 | 0.03085 | 0.03076 |
| BEHe7 | 0.9029 | 0.2133 | 0.10271 | 0.1437 | 0.13121 | 0.02905 | 0.09443 | 0.01470 | 0.08352 | -0.12537 | 0.02895 | 0.02321 |
| BEHe8 | 0.9053 | 0.2148 | 0.09457 | 0.1568 | 0.13378 | 0.03863 | 0.08317 | 0.03742 | 0.08866 | -0.12433 | 0.02779 | 0.01656 |
| BELe1 | 0.5747 | 0.3921 | -0.05775 | 0.0869 | 0.27776 | 0.01837 | 0.02434 | -0.13656 | -0.33295 | 0.18482 | 0.11829 | 0.02833 |
| BELe2 | 0.7959 | 0.3038 | -0.01862 | 0.1098 | 0.14714 | 0.00690 | 0.08416 | -0.09243 | -0.13409 | -0.12988 | 0.09351 | 0.15872 |
| BELe3 | 0.8565 | 0.3021 | 0.05732 | 0.1677 | 0.05960 | 0.06037 | 0.06688 | -0.01746 | 0.08246 | -0.10965 | 0.05864 | 0.10277 |
| BELe4 | 0.8618 | 0.3008 | 0.10356 | 0.1777 | 0.04760 | 0.04985 | 0.06443 | 0.04447 | 0.10420 | -0.09685 | 0.03611 | 0.06140 |
| BELe5 | 0.8694 | 0.2887 | 0.14018 | 0.1698 | 0.07297 | 0.04751 | 0.07882 | 0.06979 | 0.11395 | -0.09793 | 0.00900 | 0.07220 |
| BELe6 | 0.8671 | 0.2695 | 0.15138 | 0.1503 | 0.09780 | 0.03056 | 0.08966 | 0.10586 | 0.12988 | -0.08327 | 0.03128 | 0.08416 |
| BELe7 | 0.8587 | 0.2525 | 0.13400 | 0.1481 | 0.09954 | 0.01492 | 0.08364 | 0.15059 | 0.13556 | -0.05874 | 0.02002 | 0.07973 |
| BELe8 | 0.8312 | 0.2383 | 0.13900 | 0.1567 | 0.09081 | 0.03636 | 0.06038 | 0.21911 | 0.15471 | -0.02119 | -0.00235 | 0.05932 |
| BEHp1 | 0.5753 | 0.3520 | -0.02531 | 0.0865 | 0.00677 | -0.11231 | -0.00865 | -0.13486 | -0.38412 | 0.30375 | 0.24024 | -0.03835 |
| BEHp2 | 0.8264 | 0.2885 | -0.04024 | 0.1124 | 0.04670 | -0.08284 | 0.05112 | -0.10411 | -0.17600 | -0.08693 | 0.12383 | 0.11190 |
| BEHp3 | 0.8670 | 0.2877 | 0.03805 | 0.1436 | 0.01900 | -0.02812 | 0.05206 | 0.00480 | 0.00588 | -0.11420 | 0.10644 | 0.10727 |
| BEHp4 | 0.8688 | 0.2964 | 0.06854 | 0.1586 | 0.04995 | -0.00170 | 0.03276 | 0.03274 | 0.03081 | -0.11079 | 0.05868 | 0.05909 |
| BEHp5 | 0.8852 | 0.2812 | 0.10666 | 0.1594 | 0.06657 | 0.00109 | 0.03401 | 0.01238 | 0.03347 | -0.10108 | 0.04492 | 0.05293 |
| BEHp6 | 0.8913 | 0.2694 | 0.11801 | 0.1422 | 0.09259 | 0.00283 | 0.05078 | 0.00450 | 0.03983 | -0.10454 | 0.06403 | 0.08420 |
| BEHp7 | 0.8952 | 0.2631 | 0.10944 | 0.1537 | 0.09875 | 0.00928 | 0.06128 | 0.00223 | 0.04654 | -0.12068 | 0.05259 | 0.07884 |
| BEHp8 | 0.8973 | 0.2565 | 0.09930 | 0.1697 | 0.09978 | 0.02424 | 0.05450 | 0.03284 | 0.06628 | -0.11298 | 0.04124 | 0.06757 |
| BELp1 | 0.6302 | 0.2623 | -0.07024 | 0.0705 | 0.27335 | 0.14625 | 0.08076 | -0.12071 | -0.20896 | 0.21473 | 0.02728 | 0.02207 |
| BELp2 | 0.8225 | 0.2209 | -0.02514 | 0.1162 | 0.11933 | 0.09832 | 0.12178 | -0.07752 | -0.05298 | -0.15522 | 0.01991 | 0.10978 |
| BELp3 | 0.8559 | 0.2397 | 0.05665 | 0.1763 | 0.04573 | 0.12797 | 0.08119 | -0.00895 | 0.12003 | -0.14514 | 0.01164 | 0.03779 |
| BELp4 | 0.8692 | 0.2213 | 0.10913 | 0.1907 | 0.06473 | 0.08878 | 0.08887 | 0.02730 | 0.13724 | -0.12542 | 0.00073 | 0.00585 |
| BELp5 | 0.8773 | 0.2246 | 0.12381 | 0.1718 | 0.10449 | 0.09106 | 0.10487 | 0.04072 | 0.13794 | -0.11937 | -0.01459 | 0.01409 |
| BELp6 | 0.8813 | 0.2149 | 0.14536 | 0.1564 | 0.12796 | 0.06473 | 0.11526 | 0.06237 | 0.15464 | -0.10893 | 0.00967 | 0.03016 |
| BELp7 | 0.8818 | 0.2063 | 0.13215 | 0.1461 | 0.13394 | 0.04146 | 0.11433 | 0.08482 | 0.16639 | -0.09616 | 0.00859 | 0.02766 |
| BELp8 | 0.8727 | 0.2022 | 0.12442 | 0.1479 | 0.12410 | 0.03632 | 0.09816 | 0.13165 | 0.17494 | -0.08079 | 0.00396 | 0.02558 |
| LP1 | 0.7267 | 0.0865 | 0.04444 | 0.0482 | 0.13224 | -0.08209 | 0.02108 | -0.12218 | -0.03328 | 0.58988 | 0.06509 | -0.04681 |
| Eig1Z | 0.5953 | 0.0388 | 0.03387 | 0.0516 | 0.00667 | 0.00090 | 0.03137 | 0.76535 | 0.09724 | -0.07907 | -0.00862 | 0.01521 |
| Eig1m | 0.5953 | 0.0387 | 0.03389 | 0.0516 | 0.00683 | 0.00098 | 0.03149 | 0.76539 | 0.09727 | -0.07902 | -0.00864 | 0.01525 |
| Eig1v | 0.5713 | -0.0399 | 0.01702 | 0.0578 | -0.01601 | 0.03486 | 0.01509 | 0.77971 | 0.08716 | -0.08583 | 0.01832 | -0.00960 |
| Eig1e | 0.5959 | 0.0374 | 0.03380 | 0.0530 | -0.01287 | -0.00834 | 0.02906 | 0.76562 | 0.09347 | -0.08318 | -0.00193 | 0.01169 |
| Eig1p | 0.5670 | -0.0505 | 0.01447 | 0.0578 | -0.00978 | 0.04550 | 0.01343 | 0.77985 | 0.08698 | -0.08589 | 0.01604 | -0.00870 |
| SEigZ | 0.4972 | -0.3867 | -0.06993 | 0.0151 | -0.32913 | -0.12487 | -0.19748 | 0.53693 | -0.02276 | -0.00130 | 0.07470 | -0.16437 |
| SEigm | 0.4957 | -0.3852 | -0.07119 | 0.0129 | -0.33252 | -0.12610 | -0.20386 | 0.53216 | -0.02423 | -0.00171 | 0.07482 | -0.16505 |
| SEigv | -0.5883 | 0.4123 | 0.08708 | -0.0423 | -0.04142 | -0.03070 | -0.05183 | -0.57617 | -0.05661 | -0.00563 | 0.02424 | 0.25429 |
| SEige | 0.5882 | -0.4279 | -0.09291 | 0.0326 | -0.05969 | 0.00874 | -0.02870 | 0.58926 | 0.03682 | 0.01053 | -0.01521 | -0.20798 |
| SEigp | -0.5883 | 0.4095 | 0.09376 | -0.0415 | -0.06056 | -0.04907 | -0.06518 | -0.56537 | -0.06045 | -0.00195 | 0.03804 | 0.25578 |
| AEigZ | 0.5947 | 0.0406 | 0.03434 | 0.0517 | 0.00704 | 0.00111 | 0.03229 | 0.76558 | 0.09729 | -0.07899 | -0.00849 | 0.01585 |
| AEigm | 0.5947 | 0.0405 | 0.03436 | 0.0518 | 0.00720 | 0.00118 | 0.03243 | 0.76563 | 0.09733 | -0.07894 | -0.00849 | 0.01589 |
| AEigv | 0.5727 | -0.0441 | 0.01607 | 0.0578 | -0.01511 | 0.03496 | 0.01555 | 0.77918 | 0.08696 | -0.08457 | 0.01795 | -0.01208 |
| AEige | 0.5954 | 0.0391 | 0.03429 | 0.0530 | -0.01266 | -0.00820 | 0.02922 | 0.76585 | 0.09355 | -0.08301 | -0.00186 | 0.01267 |
| AEigp | 0.5689 | -0.0555 | 0.01340 | 0.0577 | -0.00851 | 0.04568 | 0.01420 | 0.77902 | 0.08666 | -0.08434 | 0.01551 | -0.01166 |
| VEA1 | 0.8602 | 0.0560 | 0.01134 | 0.0522 | 0.11258 | 0.00356 | 0.01374 | 0.26224 | -0.03503 | 0.05964 | 0.06392 | 0.00244 |
| VEA2 | -0.9494 | -0.1329 | -0.03776 | -0.1406 | -0.04473 | 0.01645 | -0.02256 | -0.08570 | -0.04401 | 0.10415 | -0.00557 | 0.02764 |
| VRA1 | 0.8195 | 0.0347 | 0.03147 | 0.0714 | 0.05241 | 0.01359 | 0.02627 | 0.55273 | 0.03901 | 0.06689 | 0.01347 | 0.02317 |
| VRA2 | 0.7155 | 0.2081 | -0.20466 | 0.0807 | 0.18747 | 0.06890 | 0.03260 | -0.17767 | -0.19209 | 0.38174 | 0.10583 | 0.20643 |
| VED1 | 0.8947 | 0.0578 | 0.04127 | 0.0885 | 0.05440 | -0.00127 | 0.02446 | 0.41684 | 0.04915 | -0.00650 | 0.01283 | 0.00496 |
| VED2 | -0.9676 | -0.1269 | -0.01750 | -0.1209 | -0.07555 | 0.01395 | -0.01771 | -0.08600 | -0.00271 | 0.05963 | -0.03056 | 0.01449 |
| VRD1 | 0.8197 | 0.0341 | 0.03241 | 0.0712 | 0.05244 | 0.01303 | 0.02614 | 0.55242 | 0.03934 | 0.06824 | 0.01343 | 0.02210 |
| VRD2 | 0.7017 | 0.2029 | -0.20080 | 0.0780 | 0.18794 | 0.06586 | 0.03235 | -0.18362 | -0.19420 | 0.40740 | 0.10857 | 0.19871 |
| VEZ1 | 0.8950 | 0.0582 | 0.03902 | 0.0875 | 0.05734 | -0.00151 | 0.02364 | 0.41605 | 0.05000 | -0.00311 | 0.00980 | 0.00648 |
| VEZ2 | -0.9673 | -0.1268 | -0.02089 | -0.1224 | -0.07185 | 0.01413 | -0.01845 | -0.08656 | -0.00192 | 0.06260 | -0.03463 | 0.01705 |
| VRZ1 | 0.8196 | 0.0342 | 0.03252 | 0.0713 | 0.05254 | 0.01314 | 0.02617 | 0.55245 | 0.03930 | 0.06812 | 0.01351 | 0.02213 |
| VRZ2 | 0.7021 | 0.2037 | -0.19913 | 0.0787 | 0.18935 | 0.06716 | 0.03243 | -0.18332 | -0.19454 | 0.40565 | 0.10932 | 0.19920 |
| VEm1 | 0.8950 | 0.0582 | 0.03900 | 0.0875 | 0.05733 | -0.00150 | 0.02362 | 0.41604 | 0.04998 | -0.00311 | 0.00978 | 0.00649 |
| VEm2 | -0.9673 | -0.1268 | -0.02092 | -0.1224 | -0.07184 | 0.01415 | -0.01852 | -0.08656 | -0.00193 | 0.06263 | -0.03466 | 0.01706 |
| VRm1 | 0.8196 | 0.0342 | 0.03252 | 0.0713 | 0.05254 | 0.01315 | 0.02618 | 0.55245 | 0.03930 | 0.06812 | 0.01350 | 0.02213 |
| VRm2 | 0.7021 | 0.2038 | -0.19913 | 0.0787 | 0.18941 | 0.06722 | 0.03251 | -0.18332 | -0.19455 | 0.40555 | 0.10924 | 0.19918 |
| VEv1 | 0.8938 | 0.0602 | 0.03774 | 0.0882 | 0.04879 | -0.00400 | 0.02488 | 0.41831 | 0.05611 | -0.00894 | 0.01197 | 0.01013 |
| VEv2 | -0.9668 | -0.1247 | -0.02007 | -0.1217 | -0.08272 | 0.01029 | -0.01674 | -0.08523 | 0.00574 | 0.05719 | -0.03254 | 0.02146 |
| VRv1 | 0.8197 | 0.0341 | 0.03238 | 0.0712 | 0.05275 | 0.01315 | 0.02614 | 0.55240 | 0.03904 | 0.06829 | 0.01355 | 0.02190 |
| VRv2 | 0.6996 | 0.2023 | -0.20173 | 0.0773 | 0.19199 | 0.06741 | 0.03196 | -0.18423 | -0.19765 | 0.40816 | 0.11007 | 0.19628 |
| VEe1 | 0.8950 | 0.0582 | 0.03981 | 0.0878 | 0.05540 | -0.00278 | 0.02466 | 0.41607 | 0.05049 | -0.00402 | 0.01153 | 0.00540 |
| VEe2 | -0.9673 | -0.1269 | -0.01964 | -0.1221 | -0.07464 | 0.01204 | -0.01712 | -0.08640 | -0.00093 | 0.06133 | -0.03211 | 0.01536 |
| VRe1 | 0.8197 | 0.0342 | 0.03249 | 0.0713 | 0.05249 | 0.01313 | 0.02609 | 0.55245 | 0.03926 | 0.06818 | 0.01350 | 0.02215 |
| VRe2 | 0.7019 | 0.2037 | -0.19966 | 0.0783 | 0.18872 | 0.06704 | 0.03147 | -0.18336 | -0.19509 | 0.40656 | 0.10929 | 0.19928 |
| VEp1 | 0.8934 | 0.0605 | 0.03704 | 0.0883 | 0.04863 | -0.00357 | 0.02471 | 0.41896 | 0.05704 | -0.00964 | 0.01130 | 0.01154 |
| VEp2 | -0.9667 | -0.1243 | -0.02068 | -0.1217 | -0.08212 | 0.01106 | -0.01700 | -0.08514 | 0.00690 | 0.05690 | -0.03357 | 0.02361 |
| VRp1 | 0.8197 | 0.0341 | 0.03238 | 0.0712 | 0.05282 | 0.01316 | 0.02616 | 0.55238 | 0.03902 | 0.06829 | 0.01355 | 0.02186 |
| VRp2 | 0.6992 | 0.2020 | -0.20202 | 0.0773 | 0.19251 | 0.06748 | 0.03231 | -0.18439 | -0.19793 | 0.40823 | 0.11028 | 0.19591 |
| GGI1 | 0.7199 | 0.0003 | 0.17761 | -0.0182 | 0.01867 | -0.13257 | 0.02829 | 0.52470 | 0.13348 | 0.25233 | -0.02584 | -0.12554 |
| GGI2 | 0.7274 | 0.0347 | 0.12349 | -0.0086 | 0.09724 | -0.10375 | 0.00468 | 0.42755 | 0.16349 | 0.39916 | 0.00274 | -0.08020 |
| GGI3 | 0.7286 | -0.0245 | 0.14665 | 0.0025 | 0.09742 | -0.12143 | 0.01360 | 0.36442 | 0.13381 | 0.44399 | -0.00689 | -0.06270 |
| GGI4 | 0.7335 | -0.0484 | 0.13535 | 0.0126 | 0.14107 | -0.05545 | 0.01266 | 0.41320 | 0.07474 | 0.38930 | -0.01031 | -0.06775 |
| GGI5 | 0.7284 | -0.0455 | 0.15301 | 0.0053 | 0.07762 | -0.05901 | 0.03410 | 0.44673 | 0.05995 | 0.30366 | -0.03725 | -0.06287 |
| GGI6 | 0.7082 | -0.0473 | 0.12291 | 0.0157 | 0.07331 | -0.01491 | 0.01180 | 0.52078 | 0.08705 | 0.24871 | -0.03360 | -0.03300 |
| GGI7 | 0.6678 | -0.0705 | 0.09590 | 0.0174 | 0.04503 | 0.01662 | 0.01038 | 0.61846 | 0.07947 | 0.16582 | -0.02037 | -0.02947 |
| GGI8 | 0.6411 | -0.0731 | 0.08603 | 0.0221 | 0.03361 | 0.01843 | 0.02262 | 0.66444 | 0.07151 | 0.12804 | -0.01300 | -0.02345 |
| GGI9 | 0.5952 | -0.0684 | 0.07633 | 0.0136 | 0.01513 | 0.01770 | 0.01990 | 0.73481 | 0.05297 | 0.05711 | -0.00869 | -0.01207 |
| GGI10 | 0.5268 | -0.0593 | 0.06183 | 0.0125 | -0.01001 | 0.02249 | 0.02211 | 0.78330 | 0.04536 | 0.01979 | -0.00091 | -0.00201 |
| JGI1 | -0.0200 | -0.1286 | 0.37643 | -0.1471 | -0.16302 | -0.31155 | 0.03612 | 0.08267 | 0.21939 | 0.35731 | -0.05033 | -0.44703 |
| JGI2 | 0.1381 | -0.0589 | 0.21784 | -0.1027 | 0.03405 | -0.23970 | -0.04329 | 0.02133 | 0.22922 | 0.55801 | 0.03756 | -0.29869 |
| JGI3 | 0.0829 | -0.1582 | 0.20931 | -0.0557 | -0.05090 | -0.30299 | 0.04383 | -0.03092 | 0.09030 | 0.53518 | -0.02617 | -0.18813 |
| JGI4 | 0.1642 | -0.1571 | 0.02931 | -0.0525 | 0.24760 | -0.13352 | -0.07972 | -0.06701 | -0.10996 | 0.46012 | 0.02927 | -0.15722 |
| JGI5 | 0.1881 | -0.0487 | 0.12054 | -0.0675 | 0.10233 | -0.15004 | 0.07173 | -0.12933 | -0.10263 | 0.23101 | -0.02946 | -0.25128 |
| JGI6 | 0.4155 | 0.0703 | 0.04148 | 0.0606 | 0.12364 | -0.04857 | -0.04014 | -0.14763 | -0.07727 | 0.16359 | -0.09408 | -0.08664 |
| JGI7 | 0.5343 | 0.0738 | 0.04840 | 0.0841 | 0.04099 | -0.00700 | -0.01608 | -0.14816 | -0.05436 | 0.00486 | -0.10629 | -0.11428 |
| JGI8 | 0.6325 | 0.1026 | -0.00528 | 0.1025 | 0.05228 | -0.01909 | 0.03951 | -0.12025 | -0.00121 | -0.03148 | -0.07891 | -0.04390 |
| JGI9 | 0.6216 | 0.1401 | 0.00278 | 0.0861 | 0.05567 | -0.02943 | 0.02305 | -0.04032 | -0.01413 | -0.09988 | -0.06087 | 0.00545 |
| JGI10 | 0.6153 | 0.0811 | 0.00733 | 0.1323 | 0.06434 | -0.05738 | 0.07758 | 0.03463 | 0.02440 | -0.05468 | -0.05749 | 0.01610 |
| JGT | 0.2311 | -0.1150 | 0.33169 | -0.1083 | -0.03631 | -0.33056 | 0.01856 | -0.00057 | 0.16309 | 0.51126 | -0.04982 | -0.42788 |

## S8. Details for the 0-2D Dragon MDs merely explained in a exclusive way into Factor 10.

| **Second Mohar index** (TI2) are calculated from the eigenvalues of the Laplacian matrix as follows:   |
| --- |
| **Path/walk Randic shape indices** (PW*k*) are calculated by summing the ratios of the atomic path count over the atomic walk count of the same order *k* and then dividing by the number of non-H atoms (nSK) [M.Randic, J. Chem. Inf. Comput. Sci. 2001, 41, 607-613. Since path/walk count ratio is independent of molecular size, these descriptors can be considered as shape descriptors. DRAGON calculates path/walk shape indices from order 2 up to 5; the index of first order is not provided as the counts of the paths and walks of length one are equal and, therefore, the corresponding molecular index always equals one for all molecules. |
| The **rotatable bond fraction** (RBF) is the number of rotatable bonds (RBN) divided by the number of bonds (nBT) in a molecule. |
| **Eigenvalues from the three weighted edge adjacency matrices** are also provided by DRAGON (EEig*k*x, EEig*k*d, EEig*k*r). For each molecular matrix the first 15 eigenvalues are retained. These molecular descriptors have not yet been proposed in the literature and therefore their meaning and role in QSAR modelling need to be a little more studied. |

## S9. Names of structures for Cramer’s steroid database and their corresponding values for the binding affinity to the corticosteroid-binding globulin (CBG).

| **ID** | **Name** | **Y Exp.** |  | **ID** | **Name** | **Y Exp.** |
| --- | --- | --- | --- | --- | --- | --- |
| 1 | aldosterone | -6.28 |  | 17 | pregnenolone | -5.23 |
| 2 | androstanediol | -5.00 |  | 18 | 17a-hydroxypregnenolone | -5.00 |
| 3 | 5-androstenediol | -5.00 |  | 19 | progesterone | -7.38 |
| 4 | 4-androstenedione | -5.76 |  | 20 | 17a-hydroxyprogesterone | -7.74 |
| 5 | androsterone | -5.61 |  | 21 | testosterone | -6.72 |
| 6 | corticosterone | -7.88 |  | 22 | prednisolone | -7.51 |
| 7 | cortisol | -7.88 |  | 23 | cortisolacetat | -7.55 |
| 8 | cortisone | -6.89 |  | 24 | 4-pregnene-3,11,20-trione | -6.78 |
| 9 | dehydroepiandrosterone | -5.00 |  | 25 | epicorticosterone | -7.20 |
| 10 | 11-deoxycorticosterone | -7.65 |  | 26 | 19-nortestosterone | -6.14 |
| 11 | 11-deoxycortisol | -7.88 |  | 27 | 16a,17a-dihydroxyprogesterone | -6.25 |
| 12 | dihydrotestosterone | -5.92 |  | 28 | 16a-methylprogesterone | -7.12 |
| 13 | estradiol | -5.00 |  | 29 | 19-norprogesterone | -6.82 |
| 14 | estriol | -5.00 |  | 30 | 2a-methylcortisol | -7.69 |
| 15 | estrone | -5.00 |  | 31 | 2a-methyl-9a-fluoro-cortisol | -5.80 |
| 16 | etiocholanolone | -5.23 |  |  |  |  |
